# Sehiridae stat. nov., the Highly-Supported Monophylum Within the ‘Cydnoid’ Complex of Pentatomoid Families (Hemiptera: Heteroptera: Pentatomoidea) †

**DOI:** 10.3390/insects17080835

**Published:** 2026-08-12

**Authors:** Jerzy A. Lis, Paweł J. Domagała, Barbara Lis, Anna Zielińska

**Affiliations:** Institute of Biology, University of Opole, Oleska 22, 45-052 Opole, Poland; pdomagala@uni.opole.pl (P.J.D.); canta@uni.opole.pl (B.L.); anna.zielinska2@uni.opole.pl (A.Z.)

**Keywords:** Cydnidae, Sehiridae, Parastrachiinae, Sehirinae, Dismegistini, Ochetostethini, new status, new tribe, tribal classification, molecular phylogeny

## Abstract

Previous molecular and morphological analyses have suggested that the family Parastrachiidae and the subfamily Sehirinae of the family Cydnidae may be more closely related than previously thought. However, no conclusive study has yet been conducted to support their recognition as a monophyletic group within the superfamily Pentatomoidea. Our analyses identified a strongly supported clade comprising species of the subfamily Sehirinae and the family Parastrachiidae. Based on the results of the present study, together with previous research on mitochondrial and nuclear DNA sequences, secondary and tertiary structures of 18S rRNA, and morphological characters, the subfamily Sehirinae is elevated to family status (Sehiridae stat. nov.), with two subfamilies (Sehirinae and Parastrachiinae). Furthermore, the subfamily Sehirinae is divided into two well-defined monophyletic tribes, Sehirini and Ochetostethini, and the subfamily Parastrachiinae is divided into Parastrachiini and Dismegistini trib. nov.

## Simple Summary

Numerous studies of the taxa included in the ‘cydnoid’ complex of pentatomoid families have examined the group’s morphology, biology, and molecular characteristics. In many of those analyses, consistent similarities between the two groups within the ‘cydnoid’ complex, namely the family Parastrachiidae and the subfamily Sehirinae of the family Cydnidae, have been observed. Both groups were also consistently identified as the clade with the highest reliability coefficients. In this study, we confirmed the existence of such a clade by considering the broadest possible range of species across the family Cydnidae and other families within the ’cydnoid’ complex. As a result, we upgraded the subfamily Sehirinae to family rank, with two subfamilies: Sehirinae and Parastrachiinae.

## 1. Introduction

The history of research on the family Cydnidae and its classification is among the most intricate within the superfamily Pentatomoidea [1]. Numerous studies have examined the group’s morphology, biology, and molecular characteristics. These aspects are comprehensively summarised in a work that reviews the entire history of research on the classification of the family Cydnidae and related families [1].

This summary [1] also highlights issues concerning this family that require immediate attention. One of these concerns is the systematic position of the family Parastrachiidae and its possible relationship with the subfamily Sehirinae within the broader concept of the family Cydnidae. Similarities between these two groups have been consistently observed, as clades comprising Parastrachiidae species and Sehirinae representatives consistently show the highest reliability coefficients (Table 1).

Therefore, in this study, we aimed to confirm the existence of such a clade by considering the broadest possible range of species across the family Cydnidae and other families within the ’cydnoid’ complex.

## 2. Materials and Methods

### 2.1. Taxa

In the present study, 100 terminal taxa were included in the analysis, comprising 53 terminal ingroup taxa of the family Cydnidae, 32 terminal ingroup taxa from other Pentatomoidea, and 15 terminal outgroup taxa (Tables S1–S3).

### 2.2. DNA Markers Selected for Analyses

We used three molecular markers, which have repeatedly been shown to be the most useful for resolving phylogenetic relationships among taxa within the ‘cydnoid’ complex of pentatomoid families [2,3,4,5,6,7,8]. These markers were the genes encoding 16S rRNA, 18S rRNA and 28S rRNA. The first two genes were sequenced and analysed in full. For the third gene, however, only the D3 region was sequenced and analysed; this is the region of the 28S rDNA most commonly analysed in Heteroptera. Newly obtained sequences for 41 species (38 in Cydnidae, 1 in Scutelleridae, 1 in Lygaeidae, and 1 in Nabidae) were deposited in GenBank (Tables S1–S3).

### 2.3. DNA Extraction, PCR Amplification, Purification, and Sequencing

DNA was extracted from specimens, either dried or preserved in ethanol, and stored in the first author’s collection (JAL). Total genomic DNA from each sample was extracted from thoracic muscle tissue using a Sherlock AX DNA Extraction Kit (A&A Biotechnology, Gdańsk, Poland) following the manufacturer’s protocol. PCR reactions for the 28S and 18S rDNA genes were performed according to the detailed protocol of Lis et al. [2], and the 16S rDNA protocol was the same as that used in the study of the origin of the coxal combs in Cydnidae [6]. PCR products were bidirectionally sequenced (Genomed S.A., Warszawa, Poland). The resulting sequences were verified by using NCBI BLAST (BLASTDBv5) searches and deposited in the NCBI GenBank under accession numbers PZ639145-PZ639148 (16S rDNA), PZ612341-PZ612381 (28S rDNA), and PZ621189-PZ621229 (18S rDNA) (Tables S1–S3).

After DNA extraction, the specimens’ remains were placed in tubes containing 96% ethanol and stored in a deep freezer at the International Research and Development Centre at the University of Opole, Poland (MCBR UO). All sequenced specimens were identified to species by the first author (JAL).

### 2.4. Photographic Documentation

Dorsal-view images of specimens were captured with a DLT-Cam PRO 20MP digital camera (Delta Optical, Poland) mounted on an Olympus SZX10 microscope, using DLT-Cam Viewer software 4.7 (Delta Optical, Nowe Osiny, Poland). The same software was used to merge multiple focal planes. The SEM micrographs were obtained using Hitachi S-3000N (Hitachi High-Tech Corporation, Tokyo, Japan) and Jeol JMS-IT200 InTouchScope™ (JEOL Europe SAS, Croissy-sur-Seine, France) microscope.

### 2.5. Morphological Nomenclature

To define and morphologically diagnose certain monophyla identified in our analyses, we have used only those morphological characters considered well-established apomorphies and already described in detail for taxa representing a particular clade within the ‘cydnoid’ complex.

The terminology for morphological characters has been adapted from Schaefer (stridulitrum and plectrum [9]); Lis and Heyna (metathoracic wing venation [10]; metathoracic wing stridulitrum [11]); Lis (mesothoracic wing venation [12]); Lis and Pluot-Sigwalt (cephalic chaetotaxy [13]; Lis and Hohol-Kilinkiewicz (abdominal trichobothria [14,15,16]); Lis and Hohol-Kilinkiewicz (adult dorso-abdominal scent glands [17]); Lis and Schaefer (tibial combs [18]); Grazia et al. (female external genitalia [8]); Pluot-Sigwalt and Lis (spermathecae [19]); Lis (coxal combs [20]); Lis (pretarsal structures [21]); Lis and Ziaja (pretarsal structures [22]); Gapon (male genitalia [23]); and Kment and Vilímová (thoracic efferent system [24]).

The nomenclature for the subregions within the secondary and tertiary structures of the Length-Variable Regions (LVRs) of the 18S rRNA follows Lis and Domagała [5] and Lis [25].

### 2.6. Phylogenetic Analysis

The input alignment dataset used for the phylogenetic analyses was generated with MAFFT [26], and Maximum likelihood (ML) and Bayesian (BI) analyses were then performed on it (File S1).

To identify the best-fitting substitution model, ModelFinder in IQ-TREE [27] was used for ML analysis, and jModelTest2 [28] was used for BI analysis. The selected models (GTR + I + G for ML, TVM + I + G for BI) were used for phylogenetic analyses.

The ML tree was inferred using IQ-TREE [27] on the web server [29] with 10,000 replicates and the ultrafast bootstrap method [30]. Bayesian inference was performed with MrBayes 3.2.6 [31] on the CIPRES server [32]. As in the previous study of the origin of coxal combs in the ‘cydnoid’ complex [6], two independent runs with four chains were conducted for 10 million generations. The Markov chain Monte Carlo analysis was sampled every 1000 generations, and 25% of the samples were discarded as burn-in. Convergence was assessed by checking that the final mean standard deviation of split frequencies was <0.05 and that each parameter’s potential scale reduction factor was close to 1.

The consensus trees from both analyses were visualised and edited using the online tool iTOL v5 [33]. Clades were considered well supported if they had bootstrap values > 70% and posterior probabilities > 0.95. The strict consensus tree resulting from the combined ML and BI analyses was produced and visualised using Mesquite v.4.03 [34]. All trees were prepared for publication in CorelDRAW 21.

## 3. Results of the Phylogenetic Analyses

### 3.1. Sequence Alignment

The final alignment (File S1) comprised 100 sequences and 2871 sites, of which 1277 were conserved and 1474 variable. A total of 914 sites were parsimony-informative, and 541 were singletons. The average nucleotide frequencies were 25.8% (T), 25.8% (A), 26.2% (G), and 22.2% (C).

### 3.2. Tree Topology

**Figure 1:**
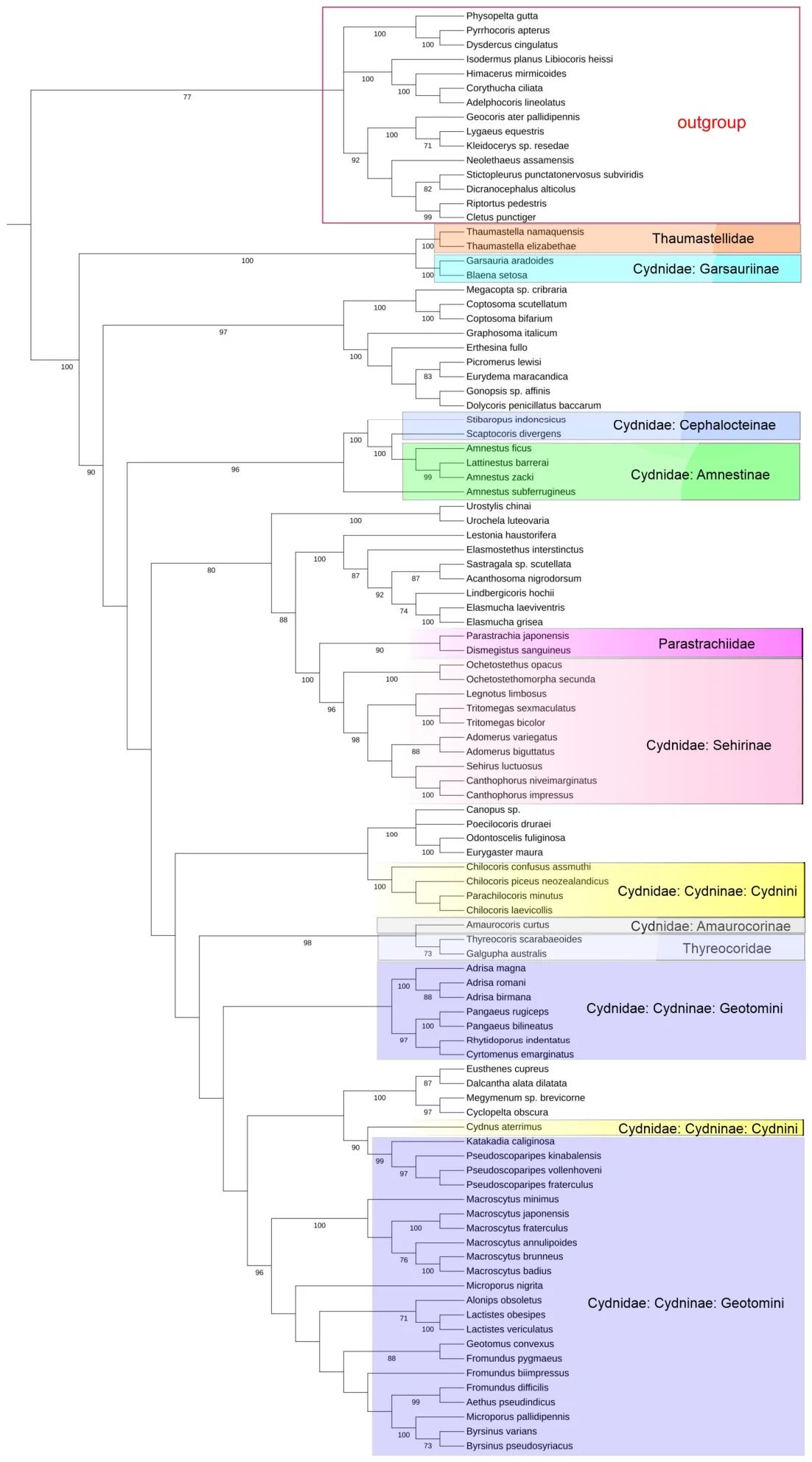
Phylogenetic consensus tree based on a Maximum likelihood analysis. Bootstrap values ≥ 70% are shown next to the branches.

**Figure 2:**
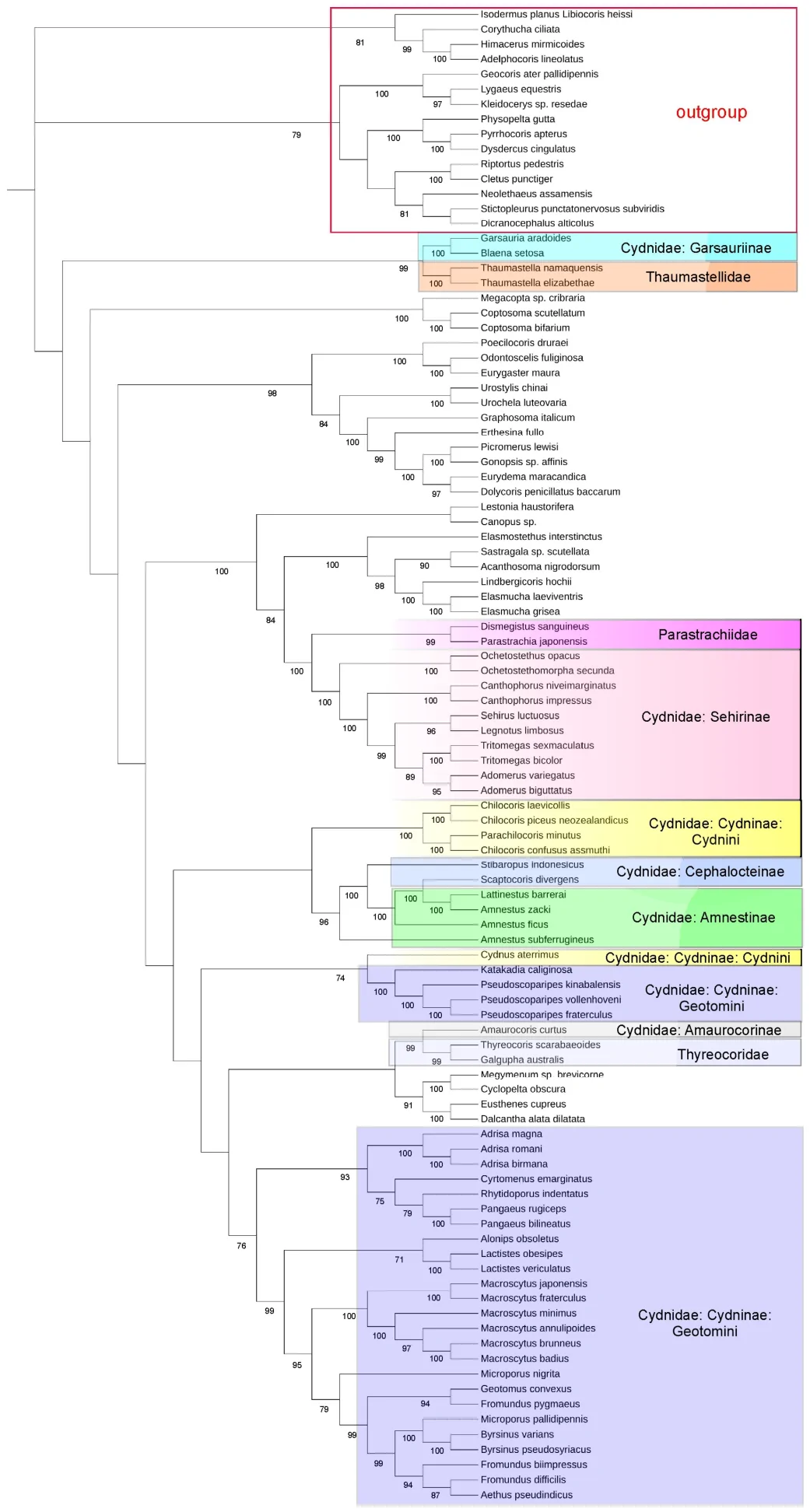
Phylogenetic consensus tree based on a Bayesian inference analysis. Posterior probability values ≥ 70% are shown next to the branches.

**Figure 3:**
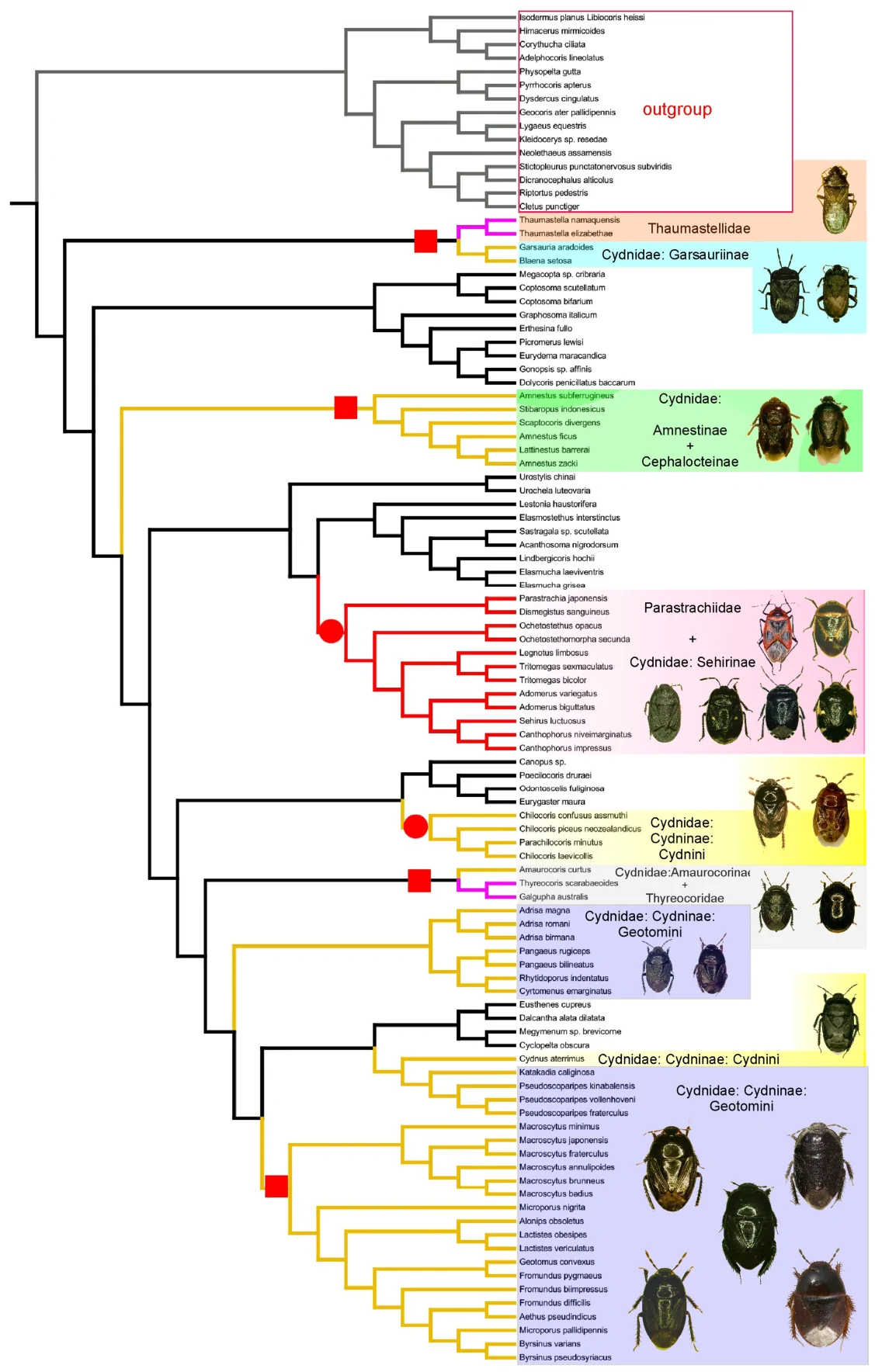
The strict consensus tree from the combined ML and BI analyses. Branches in red—a monophylum comprising Parastrachiidae and Sehirinae; branches in yellow—all other taxa within the family Cydnidae; branches in purple—the other families of the ‘cydnoid’ complex (Thaumastellidae, Thyreocoridae); branches in black—other pentatomoid taxa; branches in grey—the outgroup. Red squares on branches indicate a support value greater than 95%; red circles indicate a support value of 100%.

Both analyses (ML and BI) produced consensus trees with comparable topologies (Figure 1—the ML tree, and Figure 2—the BI tree). Differences in clade placement and the associated reliability values (ML bootstrap and BI posterior probability) were not significant. The strict consensus tree from the combined ML and BI analyses, generated and visualised in Mesquite v.4.03 [34], is shown in Figure 3 and exhibits a topology similar to those of the ML and BI trees.

The results of our study once again confirmed the non-monophyletic origin of the ‘cydnoid’ complex (Cydnidae *sensu* Schuh and Weirauch [35]), the polyphyly of the family Cydnidae (*sensu* Pluot-Sigwalt and Lis, 2008 [19]), and the polyphyly of two tribes within the subfamily Cydninae, namely Cydnini and Geotomini.

Across all our analyses, Thaumastellidae were identified as the sister group to Garsauriinae (a subfamily of the Cydnidae *sensu stricto*), together forming a highly supported monophylum (ML bootstrap value = 100 and BI posterior probability = 99). This systematic placement of Thaumastellidae within the ‘cydnoid’ complex is demonstrated for the first time in studies of this family, as Garsauriinae have never been included in any molecular analyses.

A clade comprising three groups—the subfamily Amaurocorinae (Cydnidae) and two subfamilies (Corimelaeninae and Thyreocorinae) of the Thyreocoridae—was first identified in a study of the evolutionary origin of coxal combs [6] and was also recovered in our analyses. This clade was unequivocally supported by high reliability values (ML bootstrap value = 98 and BI posterior probability = 99).

A somewhat surprising result was a monophyletic group, supported by high ML bootstrap values and BI posterior probabilities (99 in both analyses), comprising representatives of two distinct subfamilies within the family Cydnidae. These include species of the tribe Scaptocorini (subfamily Scaptocorinae) and representatives of the subfamily Amnestinae. Never before has a clade of this type been identified in any molecular study.

However, the most significant finding from our analyses was the confirmation of the monophyly of the group comprising the subfamily Sehirinae (Cydnidae) and the family Parastrachiidae. As this group has repeatedly been identified as a well-documented monophylum (see Table 1), we decided to discuss this issue in detail and to take specific steps regarding its systematic position within the ‘cydnoid’ complex.

## 4. Discussion

In all molecular analyses conducted to date that have included species of the subfamily Sehirinae and the family Parastrachiidae [2,3,4,5,6,7], these taxa have consistently formed a highly supported clade distinct from all other representatives of the family Cydnidae, irrespective of the internal classification used.

It is also crucial to note that species representing all Parastrachiidae genera, as well as species from one to eight Sehirinae genera, were included in these analyses (Table 1). Support values for the Parastrachiidae + Sehirinae clade ranged from 0.8 to 1.00 (BI = 0.98 to 1.0, ML = 0.97 to 1.0, and MP = 0.8) (Table 1). Our study also identified a well-supported clade comprising two Parastrachiidae species from two genera and ten Sehirinae species from seven genera (Figure 1, Figure 2 and Figure 3, Tables S1 and S2).

Furthermore, a recent analysis of 18S ribosomal RNA in species of the broadly conceived Cydnidae [25] revealed that the predicted secondary and tertiary structures of the Length-Variable Region L (LVR L) confirmed the close relationship between the family Parastrachiidae and the subfamily Sehirinae (Cydnidae). This was particularly evident in the numerous morpho-molecular synapomorphies shared between their LVR L subregions, especially in the tertiary structure [25].

Therefore, it is reasonable to regard the group comprising Sehirinae and Parastrachiidae species as thoroughly documented, monophyletic, and supported by molecular evidence.

In addition, the most significant results of the non-molecular studies, including external and internal morphological analyses [8,11,18,19,20,21,36,37,38,39,40,41,42], also suggest that the species of *Parastrachia* and *Dismegistus* are related to representatives of the subfamily Sehirinae. This is true not only of their morphological features but also of their shared maternal care [38,39,40,41,42,43,44,45,46,47,48,49,50].

## 5. Proposed Changes in the ‘Cydnoid’ Complex Classification

### 5.1. Family Sehiridae Amyot et Audinet-Serville, **New Status**

Séhirides Amyot et Audinet-Serville, 1843: XXI and 96 [51].
Type genus: *Sehirus* Amyot et Audinet-Serville, 1843 [51].
Zoobank: urn:lsid:zoobank.org:act:F476959A-8BD7-4E5D-87AC-45978CC6C5C2

### 5.2. Description

**Figure 4:**
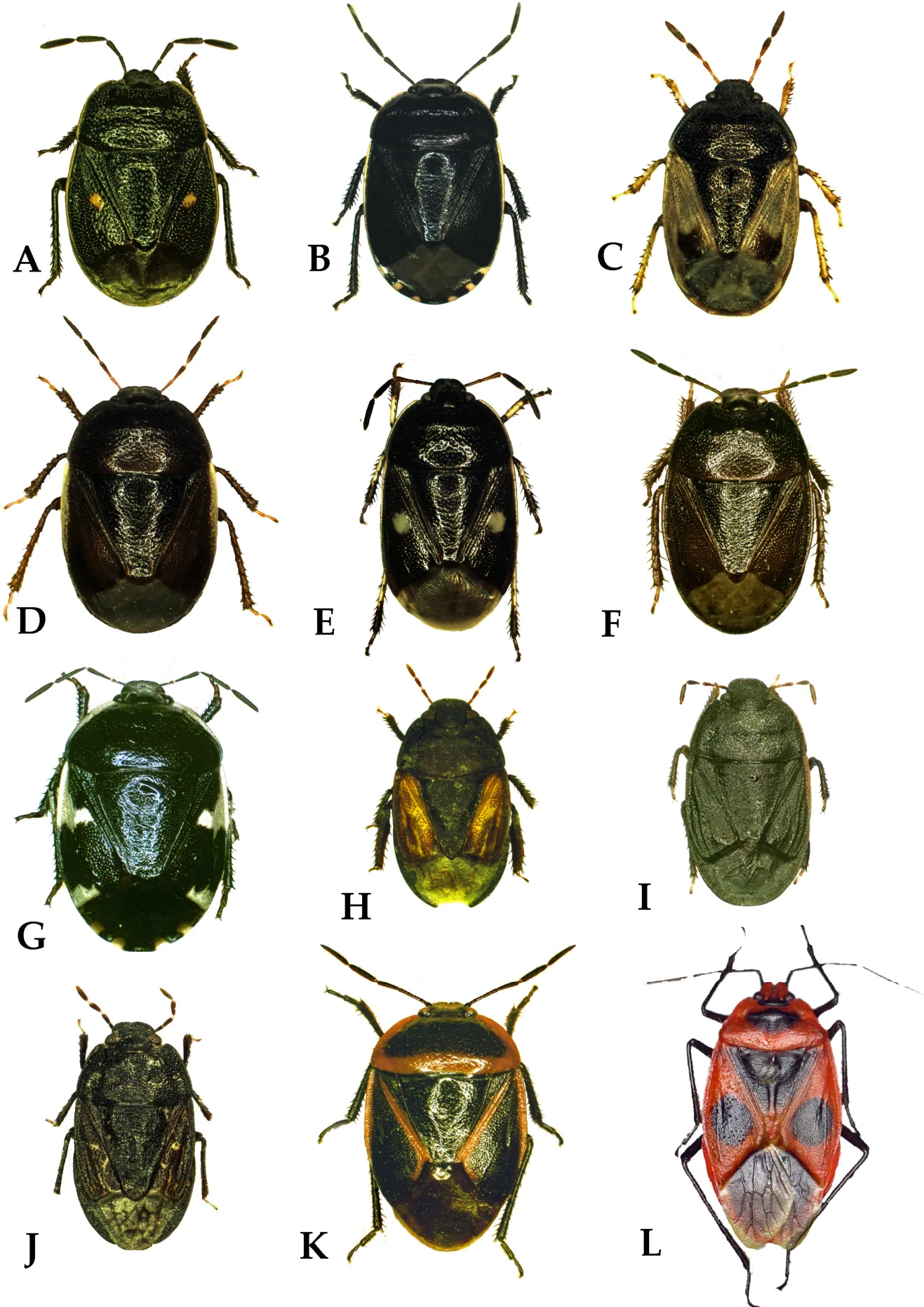
Representatives of the family Sehiridae. (**A**–**J**) Sehirinae. (**K**,**L**) Parastrachiinae. (**A**) *Adomerus biguttatus* (Linnaeus, 1758). (**B**) *Canthophorus impressus* (Horváth, 1881). (**C**) *Crocistethus waltlianus* (Fieber, 1837). (**D**) *Exosehirus marginatus* (Signoret, 1881). (**E**) *Lalervis expansa* (Signoret, 1881). (**F**) *Sehirus luctuosus* Mulsant et Rey, 1866. (**G**) *Tritomegas sexmaculatus* (Rambur, 1839). (**H**) *Ochetostethus brachyscytus* Reuter, 1891. (**I**) *O. opacus* (Scholtz, 1847). (**J**) *O. orientalis* (Distant, 1901). (**K**) *Dismegistus fimbriatus* (Thunberg, 1873). (**L**) *Parastrachia japonensis* (Scott, 1880).

Body from small (about 2.5 mm) to large (almost 20 mm); its colouration is black, dark brown or bluish, usually with white or yellow markings and body margins (Sehirinae, Figure 4A,B,D–F); sometimes also with paler corium (Figure 4C,H). In Parastrachiinae, body distinctly bicoloured, brightly red and black, and sometimes with red colour predominating (Figure 4K,L). Dorsal body side more or less convex, though sometimes flattened (Figure 4H–J).

Head semicircular or more or less subtriangular; clypeus and paraclypei without setigerous punctures; antennae five-segmented.

Pronotum wider than long, with lateral margins either carinated to varying degrees or lacking carinae. The anterior submarginal impressed line absent. The lateral margins of the pronotum are without setigerous punctures bearing long setae, although occasionally with a few very short, barely visible hair-like setae. Scutellum longer than broad.

**Figure 5:**
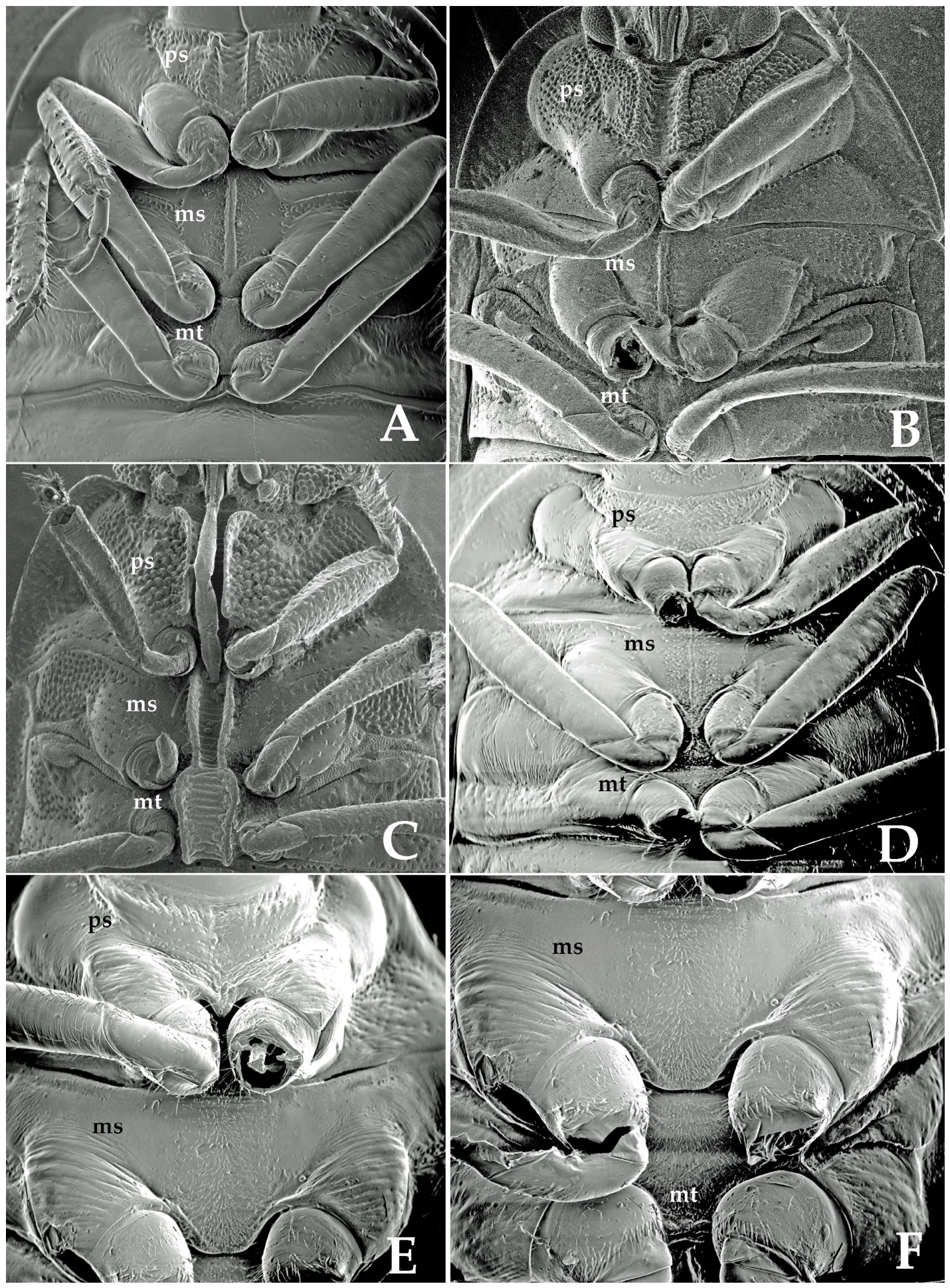
Pro-, meso-, and metasternum. (**A**–**C**) Sehirinae. (**D**–**F**) Parastrachiinae. (**A**) *Canthophorus impressus*. (**B**) Sehirus *luctuosus*. (**C**) *Ochetostethus crassicornis* Horváth, 1919. (**D**) *Dismegistus fimbriatus*. (**E**,**F**) *Parastrachia japonensis* (ps—prosternum, ms—mesosternum, mt—metasternum).

Prosternal median sulcus deep and well-developed, with high longitudinal ridges on its lateral sides (Sehirinae) (Figure 5A–C), or entirely absent or unnoticeable, without longitudinal ridges (Parastrachiinae) (Figure 5D–F). Mesosternum is flat, without a median carina (Parastrachiinae: Parastrachiini) (Figure 5E,F), with more or less percurrent median carinae (Sehirinae: Sehirini; Parastrachiinae: Dismegistini) (Figure 5A,B,D), or with a verse-ribbed, deep, median groove (Sehirinae: Ochetostethini) (Figure 5C). Metasternum without a median carina or groove (Parastrachiinae: Parastrachiini) (Figure 5F), with a low, barely visible median carina (Sehirinae: Sehirini; Parastrachiinae: Dismegistini) (Figure 5A,B,D), or with a broad, deep median groove bearing transverse ribs (Sehirinae: Ochetostethini) (Figure 5C).

Evaporatorium on metapleuron well-developed or rudimentary [24,37,52,53,54,55].

Mesothoracic wing with the costal vein (C) entirely coalescent with the subcosta (Sc); radial vein (R) entirely, almost entirely, or only in its basal part is coalescent with the median vein (M); cubital vein (Cu) widely separated from R + M; first anal vein (1A) present; the second anal vein (2A) absent [12]. Costal margins without setigerous punctures.

Metathoracic wing with the vannus I and its interclaval veins (Icl) present; vannus II and III also well-differentiated; subcostal vein (Sc) coalescent with the basal part of the radial vein (R); apical branches of the radial (R) and median (M) veins arise close to each other; cross vein r-m absent; posterior cubital vein (pCu) present and connected to the posterior branch of the anterior cubital vein (aCu); first (1A), second (2A), and third (3A) anal veins present; hamus in radial cell present (Sehirinae: Sehirini; Parastrachiinae) or absent (Sehirinae: Ochetostethini) [10]. Stridulitrum on the first anal vein (1A) is present (Sehirinae; Parastrachiinae: Parastrachiini) or absent (Parastrachiinae: Dismegistini) [10,11].

Legs gracile [2,52,54,56], with anterior tibiae terete, narrow and only slightly expanded, their marginal tibial spines short and slender, similar to those on the other tibiae (Parastrachiinae: Parastrachiini) [2] (Figure 4L), or marginal spines stouter (Parastrachiinae: Dismegistini) [2,56] (Figure 4K), or anterior tibiae apically more or less broadened, and armed with marginal setae stouter than those on the other tibiae (Sehirinae) [52,54] (Figure 4A–J); tarsi always inserted at the apices of the tibiae.

**Figure 6:**
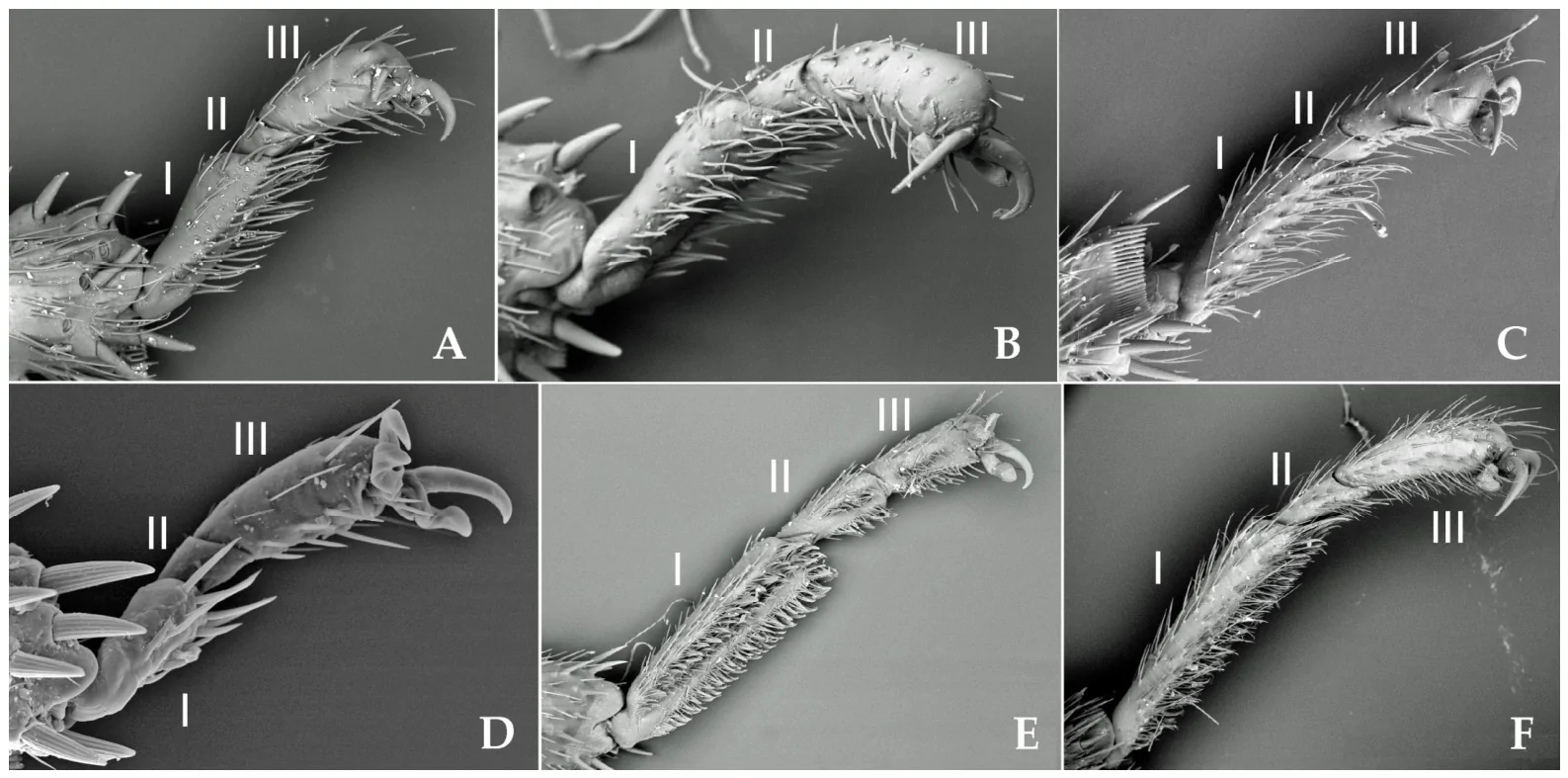
Tarsus. (**A**–**D**) Sehirinae. (**E**,**F**) Parastrachiinae. (**A**) *Adomerus biguttatus*. (**B**) *Canthophorus impressus*. (**C**) *Lalervis expansa*. (**D**) *Ochetostethus tarsalis* (Mulsant et Rey, 1852). (**E**) *Dismegistus fimbriatus*. (**F**) *Parastrachia japonensis* (I—basitarsus, II—tarsomere 2, III—tarsomere 3).

Tarsus with a second segment (tarsomere 2) very short, more or less thinner than the first or third, the undersurface of the basitarsus with stout, long setae usually arranged in two rows (Figure 6).

Pretarsal structures with pulvilli divided into basi- and distipulvilli; the claws, parempodia (parempodial setae), and the empodium are typically pentatomoid [21,22].

The tibial comb complex is characterised by numerous setae along the lateral margins of the tibial fossula [18]. The median concavity of the tibial fossula is covered with setae of varying lengths (particularly short in *Dismegistus*, short in all Sehirinae, and long in *Parastrachia*) [18]. The coxal comb consists of a more or less regular row of scale-like or gutter-like setae [20].

Abdominal trichobothria in pairs (2 + 2) on lateral parts of ventrites III to VII arranged in oblique or transverse pairs posterior to the spiracle; sometimes (Sehirinae: Sehirini) trichobothria of sternum III arranged in longitudinal pairs, with one trichobothrium distinctly anterior to the spiracle [14,15,16].

Male genital capsule with its opening dorsally, sometimes nearly horizontal; aedeagus bears two pairs of conjunctival appendages; paramere elongated with the hypophysis digitate, more or less recurved apically [23,55].

Female eighth ventral laterotergites in contact with each other usually not entirely fused, bearing a more or less visible furrow or rim [8,57,58].

Spermathecae of two types [19]. The first (recovered in the tribe Sehirini of the subfamily Sehirinae and the subfamily Parastrachiinae) can be differentiated by the elongate or ovoid apical receptacle, a long, twisted neck, and the intermediate part of the spermatheca rather straight, with two more or less expanded, thin flanges [19]. The second, restricted to the tribe Ochetostethini of the subfamily Sehirinae, is very simple, with the apical receptacle spherical and lacking a neck, and the flanges in its intermediate part [19].

### 5.3. Parental Care

It has been observed that females of species in the genus Parastrachia (Parastrachiinae) and in the Sehirinae exhibit distinctive egg-guarding behaviour, e.g., [38,39,40,41,42,43,44,45,46,47,48,49,50]. This maternal care, in which the female protects the eggs, is widespread across the entire Pentatomoidea, e.g., [44,59,60,61,62,63,64,65,66,67], and has also been observed in other heteropteran families outside the pentatomoid bugs, e.g., [44,61,63,67,68,69,70,71].

Nevertheless, until recently, no published data on parental care in species of the genus *Dismegistus* (Parastrachiidae) were available. New reports of females in *Dismegistus* species carrying and guarding egg batches [50] provide further evidence of the close relationship between *Parastrachia* and *Dismegistus*, and between the Parastrachiidae and the Sehirinae. This evidence confirms that the entire family Sehiridae is characterised by parental care.

### 5.4. Differential Diagnoses

Because many morphological characters in Pentatomoidea remain poorly studied and have yet to be analysed across all families within the superfamily, we have focused on those that have been extensively studied at all supraspecific taxonomic levels for the Cydnidae *sensu* Schuh and Weirauch [35] to define diagnostic features of the family Sehiridae.

The characters defining the family Sehiridae (including Sehirinae and Parastrachiinae), which are not found in the taxa of the broadly defined Cydnidae or in any other family of the Pentatomoidea, are as follows.

(1) The cephalic primary setae I-V in the nymphal stage 5 and adults are absent [13,72]. However, only the absence of primary setae III and IV in the nymphal stage 5 and adults can be considered the family autapomorphy [13,72].

(2) The pronotal and costal margins of the hemelytra lack setigerous punctures bearing secondary setae [37,38,53,73,74,75]. By contrast, all other Cydnidae taxa (Amaurocorinae, Amnestinae, Cephalocteinae, Cydninae, Garsauriinae) show setigerous punctures on these margins, though these can be secondarily lost [8,53,75,76,77]. This character is similar to that exhibited by representatives of the family Thyreocoridae [8,74,75,76,77,78,79]. Nevertheless, the Sehiridae can be readily distinguished from the Thyreocoridae by the shape of the scutellum. In the former family, the scutellum is triangular and typically developed, whereas in the latter it is greatly enlarged and covers almost the entire mesothoracic wings [8,74,75,76,77,78,79].

(3) The tibial combs complex is characterised by the tibial fossula with numerous setae along its lateral margins [18]; on the contrary, only a few setae along each lateral margin, or a few setae along the outer lateral margin and numerous setae along the inner lateral margin, are typical of other taxa within the broadly defined Cydnidae [18].

(4) The median concavity of the tibial combs complex is covered with setae of various lengths (very short setae in the tribe Dismegistini (Parastrachiinae), short in Sehirinae, and long setae in the tribe Parastrachiini (Parastrachiinae) [18]. The smooth median concavity, lacking pilosity, was recovered across all other subfamilies of the broadly defined Cydnidae [18].

(5) The adult dorso-abdominal scent glands are between the abdominal terga III and IV, IV and V, V and VI, unpaired and located in the midline of the abdominal segments [17,80]. In all other Cydnidae *sensu* Schuh and Weirauch [35] and other Pentatomoidea, these glands follow a different pattern [17,80].

Moreover, we identified molecular characters that can serve as synapomorphies for the family Sehiridae.

(1) The subregion LA (A1 + A2) within the Length-Variable Region L (LVR L) of the 18S rRNA is characterised by the fewest nucleotides, namely 12 (6 + 6), whereas all other families of the Pentatomoidea, including the broadly defined Cydnidae, exhibit 14 (7 + 7) to 18 (9 + 9) nucleotides in this subregion [5,25]. Only the Amnestinae show fewer nucleotides in this subregion, namely 11, but their numbers in LA1 and LA2 are highly asymmetric, i.e., 10 + 1, respectively [5].

(2) The secondary and tertiary structures of the LA subregion within the LVR L of the 18S rRNA are unique to the Sehiridae (including Sehirinae and Parastrachiinae). These structures differ from those reported for the superfamily Pentatomoidea [5,25].

### 5.5. Key to Subfamilies of the Family Sehiridae

1. Body small, medium-sized to large, from about 2.0 mm to almost 12.0 mm; its colouration uniform (black, dark brown or bluish) or bicoloured, but never bright red and black (Figure 4A–J); coxal combs composed of a regular row of elongated scale-like or gutter-like setae [6, Figure 1H,I]; prosternal median sulcus deep, and well-developed, with high longitudinal ridges on its lateral sides (Figure 5A–C) . . . . . . . . . . . . . Sehirinae
- Body always large, significantly above 12.0 mm, sometimes reaching 21.0 mm, brightly coloured red and black (Figure 4K,L); coxal combs composed of an irregular row of long, apically sharpened, stout setae [6, Figure 1J]; prosternal median sulcus absent or almost unnoticeable, without longitudinal ridges (Figure 5D–E) . . . . Parastrachiinae

### 5.6. Subfamily Sehirinae

Subfamily Sehirinae Amyot et Audinet-Serville, 1843 [51]
Séhirides Amyot et Audinet-Serville, 1843: XXI and 96. Type genus: *Sehirus* Amyot et Audinet-Serville, 1843 [51].

*Diagnosis.* Body size from small (about 2.0–2.5 mm) to large (up to about 12.0 mm), uniformly black, dark brown, or bluish, sometimes bicoloured (with distinct pale markings); dorsal side more or less convex, though sometimes flattened. Head, pronotal and costal margins without setigerous punctures. Prosternal median sulcus deep and well-developed, with high longitudinal ridges along its lateral sides. Metathoracic wing with stridulitrum on the first anal vein (1A). Legs gracile, anterior tibiae apically more or less broadened, and armed with marginal setae stouter than those on the other tibiae. Coxal combs composed of a regular row of elongated, scale-like or gutter-like setae.

*Key to tribes of the subfamily Sehirinae*

1. Body more or less flat, opaque, its dorsal side densely punctate (Figure 4H–J); mesosternum medially with a deep, transversely ribbed groove (Figure 5C); metasternum with a broad, deep median groove bearing transverse ribs (Figure 5C) . . . . . . . . . . . . . *Ochetostethini*
- Body more or less convex, shining, its dorsal side impunctate or almost impunctate (Figure 4A–G); mesosternum flat, with more or less percurrent median carinae (Figure 5A,B); metasternum with a low, sometimes hardly visible median carina (Figure 5A,B) . . . . . . . . . . . . . . . . . . . . . . . . . . . . . . . . . . . . . . . . . . . . . . . . . . . . . . . . . . . . . . . . . . . . . *Sehirini*

#### 5.6.1. Tribe *Ochetostethini* Mulsant et Rey, 1866, ***Restored Status***

Ochétostéthaires Mulsant et Rey, 1866: 7, 74. Type genus: *Ochetostethus* Fieber, 1860 [81].

*Diagnosis.* Body usually small or medium-sized, about 2.0–4.2 mm long, more or less flat, with the dorsal side opaque and densely punctate (Figure H–J). Mesosternum with a deep midline groove, the surface of which is distinctly transversely ribbed (Figure 5C); metasternal median groove broad and deep, also bearing transverse ribs (Figure 5C). Abdominal trichobothria on the lateral parts of ventrites III–VII, arranged in oblique pairs (Figure 1A in [16]). Metathoracic wing without a hamus in the radial cell (Figures 144–147 in [10]). Spermathecae simple, with the apical receptacle spherical, without a neck and flanges in its intermediate part (Figure 62 in [19]).

*Genera included* [82,83,84]: *Ochetostethomorpha* Schumacher, 1913 (two species); *Ochetostethus* Fieber, 1860 (16 species).

*Comments.* The *Ochetostethus* species-group was already recognised by Linnavuori [84] on the basis of the general geotomine-like habitus of the species (see, for example, *Geotomus granulosus* J.A. Lis & B. Lis, 2022, which represents the tribe Geotomini of the Cydnidae [85]), the absence of a hamus in the radial cell of the metathoracic wing, a sulcate mesosternum, and a geotomine-like pygophore.

#### 5.6.2. Tribe *Sehirini* Amyot et Audinet-Serville, 1843

*Diagnosis.* Body from small to large (2.5 to 12.0 mm), uniformly black, dark brown, or bluish, sometimes with distinct pale markings; dorsal side more or less convex, impunctate or almost impunctate (Figure 4A–G). Mesosternum flat, with more or less percurrent median carinae (Figure 5A–B); metasternum with a low, sometimes barely visible median carina (Figure 5A–B). Abdominal trichobothria on the lateral parts of ventrites III to VII arranged in transverse pairs posterior to the spiracle (Figure 1B in [16]; sometimes trichobothria of sternum III arranged in longitudinal pairs, with one trichobothrium distinctly anterior to the spiracle (Figure 1C in [16]). Hamus in the radial cell of the metathoracic wing present (Figures 131–143 in [10]). Spermatheca with the apical receptacle elongate or ovoid, with a long, twisted neck, and two more or less expanded, thin flanges (Figures 51–61 in [19]).

*Genera included* [23,52,55,74,81,82,84]: *Adomerus* Mulsant et Rey, 1866 (seven species); *Canthophorus* Mulsant et Rey, 1866 (eight species); *Crocistethus* Fieber, 1860 (four species); *Exosehirus* Wagner, 1963 (five species); *Lalervis* Signoret, 1881 (three species); *Legnotus* Schiødte, 1848 (six species); *Sehirus* Amyot et Audinet-Serville, 1843 (12 species); *Singeria* Wagner, 1955 (one species); *Tacolus* Schouteden, 1910 (one species); *Tritomegas* Amyot et Audinet-Serville, 1843 (6 species).

### 5.7. Subfamily Parastrachiinae

Subfamily Parastrachiinae, ***Reviewed Status***
Parastrachiinae Oshanin, 1922: 145 (Parastrachiaria). Type genus: *Parastrachia* Distant, 1883 [86].
Parastrachiinae: Schaefer et al., 1988: 307 [37].
Parastrachiidae: Sweet and Schaefer, 2002: 446 [38].

The monophyly of the group, comprising species of *Parastrachia* and *Dismegistus*, was first proposed by Grazia et al. [8] based on morphological and molecular analyses. This hypothesis has been repeatedly recovered and argued for in numerous subsequent papers (e.g., [3,4,5,87,88,89]).

All taxa of the subfamily Parastrachiinae display the typical morphological attributes of the family Sehiridae, as previously discussed (see above). Nevertheless, both genera of Parastrachiinae (*Dismegistus* and *Parastrachia*) exhibit two autapomorphies in their pretarsal structures [21]. The first of these autapomorphies is the presence of stout pulvilli, which differentiates the subfamily from all other taxa within Pentatomoidea [21,22]. The second autapomorphy is the absence of the peculiar membrane that surrounds the pretarsal structures (i.e., the membrane with microtrichia). This character is specific to all taxa included in the broadly conceived Cydnidae (*sensu* Schuh and Weirauch, 2020 [35]), but was not recovered in the Parastrachiinae [21,22].

*Genera included* [2,8,35,50,56,90]: *Dismegistus* Amyot and Audinet-Serville, 1843 (seven species); *Parastrachia* Distant, 1883 (two species).

*Key to tribes of the subfamily Parastrachiinae*

1. Dorsal body side with black colour predominating (Figure 4K); uradenia present [91]; male eighth abdominal segment not exposed posteriorly [91]; dorsal side of the abdominal segment VIII unsclerotised [91]; spiracles on the abdominal segment VIII present [91]; mesosternum flat with more or less percurrent median carinae (Figure 5D) . . . . *Dismegistini*
- Dorsal body side with red colour predominating (Figure 4L); uradenia absent [91]; male eighth abdominal segment exposed posteriorly [91]; dorsal side of the abdominal segment VIII well-sclerotised [91]; spiracles on the abdominal segment VIII atrophied [91]; meso-sternum flat, without a median carina (Figure 5E,F) . . . . . . . . . . . . . . . . . . . . . . . . . . . *Parastrachiini*

#### 5.7.1. Tribe *Dismegistini* Lis J.A. and Lis B., ***New Tribe***

Type genus: *Dismegistus* Amyot and Audinet-Serville, 1843, monotypic, based on *Cydnus circumcinctus* Hahn, 1834 (=*Cimex fimbriatus* Thunberg, 1783) [51]
**Zoobank number (new tribe):**
urn:lsid:zoobank.org:act:E3B0756A-CB5C-42FB-833A-AD994D555DC4

Taxa of this tribe exhibit the general characteristics of the family Sehiridae and the subfamily Parastrachiinae (see above). The diagnostic characters that may be considered autapomorphies for this tribe are listed below.

(1) Body brightly coloured red and black, with black predominating (Figure 4L).

All other species of the Sehiridae are almost uniformly dark-coloured or bicoloured, but none have bright red markings. The only exception is the genus *Parastrachia*, in which the body is also brightly coloured red and black, with red predominating (Figure 4L).

(2) Uradenia (the voluminous paired paragenital exocrine glands) present [91].

These glands are situated on the intersegmental membrane between segments VIII and IX in males of *Dismegistus* only [91]. None of the genera of Sehiridae, nor any other genus of the superfamily Pentatomoidea, possesses these structures [91]. They have been previously documented in other pentatomomorphan families, including the Coreoidea, Lygaeoidea, and Pyrrhocoroidea [91]. However, the uradenia of *Dismegistus* differ from those of other pentatomomorphan bugs in two ways. Firstly, they are located on the dorsal lateral side of the body rather than the ventral side. Secondly, they are paired rather than unpaired and median [91]. Moreover, the gland is glove-like, and its digitate shape is specific only to *Dismegistus*. However, it is somewhat similar to multi-digitate or lobate glands found in males of the superfamily Coreidae [91].

(3) Metathoracic stridulitrum on the first anal vein (1A) absent [10,11].

In Pentatomoidea, the metathoracic stridulitrum is present on the first anal vein (1A) in many families, including Canopidae, Cyrtocoridae, Megarididae, Plataspidae, Scutelleridae, Tessaratomidae and Urostylididae [8,9,75,77]. This structure is also known in all families (and subfamilies) classified within the broadly defined Cydnidae, except the *Garsauriinae* [8,11]. However, in species of the latter, the basal part of 1A is slightly wrinkled [11], which may suggest secondary reduction in the stridulitrum. Conversely, in species of *Dismegistus*, the surface of the basal part of 1A is devoid of wrinkles [11], which suggests that two independent evolutionary events occurred (one in *Garsauriinae*, the second in *Dismegistini* of *Parastrachiidae*). Notably, the well-developed metathoracic stridulitrum is present in the second tribe of the Parastrachiinae, the Parastrachiini, and has a similar shape and organisation to that found in the other Sehiridae [11].

*Other characters separating Dismegistini and Parastrachiini*

As previously documented by Pluot-Sigwalt and Lis [91], several characteristics of the male abdominal segment VIII distinguish *Dismegistus* from *Parastrachia*. In *Dismegistus*, it is completely inserted into the seventh abdominal segment and is not exposed posteriorly, as it is in *Parastrachia*. In addition to *Parastrachia*, the male abdominal segment VIII is exposed ventrally only in the Acanthosomatidae [92], Urostylididae [93], Lestoniidae and some Tessaratomidae [8].

Furthermore, in *Dismegistus*, the dorsal side of abdominal segment VIII is unsclerotised [91]. It is similarly membranous in some other taxa within the superfamily Pentatomoidea (Pentatomidae, Acanthosomatidae, Tessaratomidae, Dinidoridae) [94,95,96,97,98]. In contrast, in *Parastrachia*, the dorsum of abdominal segment VIII is well-sclerotised [19].

A further significant distinction between *Dismegistus* and *Parastrachia* is the presence of functional spiracles on abdominal segment VIII in the former and non-functional, atrophied spiracles in the latter [91]. The presence of functional spiracles is associated with the occurrence of uradenia in *Dismegistus* [91]. These glands are always accompanied by a network of tracheae, which supply the oxygen necessary for their activity [91]. Within Pentatomoidea, functional spiracles on segment VIII have been described in some Phloeidae and Tessaratomidae [96,98] and in a single species of Pentatomidae, *Rhaphigaster nebulosa* (Poda, 1761) [99].

Other characters, particularly the egg structure and the male and female genitalia, were previously proposed as autapomorphies of the genus *Dismegistus* [73,75]. However, these characters cannot be considered autapomorphies because they have not been comprehensively studied across all families of the superfamily Pentatomoidea. Consequently, they currently lack sufficient phylogenetic context.

*Genus included* [56]: *Dismegistus* Amyot and Audinet-Serville, 1843 (seven species).

#### 5.7.2. Tribe *Parastrachiini* Oshanin, 1922

*Parastrachiini* Oshanin, 1922: 145 (Parastrachiaria). Type genus: *Parastrachia* Distant, 1883 [86].
Parastrachiinae: Schaefer et al., 1988: 307 [37].
Parastrachiidae: Sweet and Schaefer, 2002:446 [38]; Lis, 2006: 118 [100].

All diagnostic characters of *Parastrachia*, the only genus currently representing the tribe Parastrachiini, have been discussed in detail by Schaefer et al. [37] and Sweet and Schaefer [38]. Therefore, they are not repeated here. Furthermore, all characters that distinguish *Parastrachiini* from the newly established tribe *Dismegistini* have already been delineated above. Additionally, the characters that distinguish the two tribes have been included in the key to the tribes of Parastrachiinae.

*Genus included* [37,38,100]: *Parastrachia* Distant, 1883 (two species).

## 6. Conclusions

1. Our study is the first to compile comprehensive morphological, biological, and molecular evidence supporting a close relationship between Sehirinae and Parastrachiidae.
2. It presents the most extensive molecular testing of this hypothesis so far and offers a clear systematic resolution by elevating the subfamily Sehirinae to a distinct family, which includes Sehirinae and Parastrachiinae.
3. Our findings verify that the ‘cydnoid’ complex (Cydnidae as defined by Schuh and Weirauch [35]) does not stem from a single common ancestor, thus confirming its non-monophyly.
4. Additionally, the family Cydnidae (as defined by Pluot-Sigwalt and Lis, 2008 [19]) is polyphyletic, comprising species with distinct evolutionary origins.
5. Furthermore, two tribes within the subfamily Cydninae—Cydnini and Geotomini—are polyphyletic.
6. A notable result of our analyses is the identification of Thaumastellidae as the sister group to Garsauriinae, a subfamily within Cydnidae *sensu stricto*, which was used in this type of analysis for the first time.
7. A clade including three groups—the subfamily Amaurocorinae (Cydnidae) and two subfamilies of the Thyreocoridae (Corimelaeninae and Thyreocorinae)—was initially identified in a study on the evolutionary origin of coxal combs [6] and was also found in our analyses.
8. A somewhat unexpected outcome is the discovery of a monophyletic group that includes members of the tribe Scaptocorini (subfamily Scaptocorinae) and the subfamily Amnestinae. This is the first time a clade of this nature has been detected in any molecular research.
9. Although many relationships found in our study were not the main focus here, they offer useful insights and suggest promising directions for future research.

## Figures and Tables

**Table 1:** The values supporting the Parastrachiidae + Sehirinae clade across various studies.

Genes Analysed	Support Value for the Parastrachiidae + Sehirinae Clade	Species of Parastrachiidae Included	Species of Sehirinae Included	References
18S rDNA 28S rDNA	Bayesian Inference (100)	*Dismegistus sanguineus* *Parastrachia japonensis*	*Adomerus biguttatus*, *Canthophorus niveimarginatus*, *Ochetostethomorpha secunda*, *Sehirus luctuosus*, *Tritomegas sexmaculatus*	[2]
16S rDNA COI 18S rDNA 28S rDNA	Maximum Likelihood (98) Maximum Parsimony (80)	*Dismegistus sanguineus* *Parastrachia japonensis*	*Adomerus biguttatus*, *Canthophorus niveimarginatus*, *Ochetostethomorpha secunda*, *Sehirus luctuosus*, *Tritomegas sexmaculatus*	[3]
16S rDNA COI 18S rDNA 28S rDNA	Maximum Likelihood (98) Bayesian Inference (100)	*Dismegistus sanguineus* *Parastrachia japonensis*	*Adomerus biguttatus*, *Sehirus luctuosus*	[4]
18S rDNA	Maximum Likelihood (99)	*Dismegistus sanguineus* *Parastrachia japonensis*	*Adomerus biguttatus*, *Canthophorus niveimarginatus*, *Ochetostethomorpha secunda*, *Ochetostethus opacus*, *Sehirus luctuosus*, *Tritomegas bicolor*, *T. sexmaculatus*	[5]
16S rDNA	Maximum Likelihood (97) Bayesian Inference (98)	*Dismegistus sanguineus* *Parastrachia japonensis*	*Adomerus biguttatus*, *A. rotundus*, *A. triguttulus*, *A. variegatus*, *Canthophorus impressus*, *C. niveimarginatus*, *Crocistethus waltlianus*, *Legnotus limbosus*, *Ochetostethomorpha secunda*, *Ochetostethus brachyscytus*, *O. heissi, O. nanus*, *O. opacus*, *Sehirus luctuosus*, *Tritomegas bicolor*, *T. sexmaculatus*	[6]
12S rDNA 16S rDNA 18S rDNA 28S rDNA	Maximum Likelihood (98/100/99/100)	*Dismegistus sanguineus* *Parastrachia japonensis*	*Canthophorus melanopterus*	[7]

## Data Availability

The original contributions presented in the study are included in the article and Supplementary Materials; further inquiries can be directed to the corresponding author.

## Supplementary Materials

The following supporting information can be downloaded at: https://www.mdpi.com/article/10.3390/insects17080835/s1, File S1. The input alignment dataset for the phylogenetic analyses generated using MAFFT. Table S1. List of Cydnidae specimens used in the phylogenetic analyses with GenBank accession numbers. Table S2. List of Pentatomoidea specimens (other than Cydnidae) used in the phylogenetic analyses with GenBank accession numbers. Table S3. List of outgroup specimens used in the phylogenetic analyses with GenBank accession numbers.

## References

[1] Lis J.A. (2025). Classification of the Burrower Bugs (Hemiptera: Heteroptera: Cydnidae): A Never-Ending Story?. Insects.

[2] Lis J.A., Ziaja D., Lis B., Gradowska P.A. (2017). Non-monophyly of the “cydnoid” complex within Pentatomoidea (Hemiptera: Hete-roptera) revealed by Bayesian phylogenetic analysis of nuclear rDNA sequences. Arthropod Syst. Phylogeny.

[3] Bianchi F.M., Barão K.R., Grassi A., Ferrari A. (2021). A milestone for Pentatomoidea: Grazia et al. 2008-What do we know and where can we go?. Zootaxa.

[4] Roca-Cusachs M., Schwertner C.F., Kim J., Eger J., Grazia J., Jung S. (2022). Opening Pandora’s box: Molecular phylogeny of the stink bugs (Hemiptera: Heteroptera: Pentatomidae) reveals great incongruences in the current classification. Syst. Entomol..

[5] Lis J.A., Domagała P.J. (2024). Inconsistencies in the Classification of the Family Cydnidae (Hemiptera: Heteroptera: Pentatomoidea) Revealed by Molecular Apomorphies in the Secondary and Tertiary Structures of 18S rRNA Length-Variable Region L (LVR L). Int. J. Mol. Sci..

[6] Lis J.A., Domagała P.J., Lis B. (2024). New Molecular Phylogenetic Evidence Confirms Independent Origin of Coxal Combs in the Families of the ‘Cydnoid’ Complex (Hemiptera: Heteroptera: Pentatomoidea). Insects.

[7] Luo J.-Y., Wu Y.-Z., Kment P., Salomão A.T., Damken C., Wang Y.-H., Xie Q. (2025). Origin of the only myrmecomorphic stink bug, *Pentamyrmex spinosus* (Hemiptera: Pentatomidae), in the radiation era of ants (Hymenoptera: Formicidae). Syst. Entomol..

[8] Grazia J., Schuh R.T., Wheeler W.C. (2008). Phylogenetic relationships of family groups in Pentatomoidea based on morphology and DNA sequences (Insecta: Heteroptera). Cladistics.

[9] Schaefer C.W. (1981). The sound-producing structures of some primitive Pentatomoidea (Hemiptera: Heteroptera). J. N. Y. Entomol. Soc..

[10] Lis J.A., Heyna J. (2001). Metathoracic wing venation in Cydnidae (Hemiptera: Heteroptera) and its bearing on the classification of the family. Ann. Zool..

[11] Lis J.A., Heyna J. (2001). The metathoracic wing stridulitrum of the Cydnidae (Hemiptera: Heteroptera). Pol. J. Entomol..

[12] Lis J.A. (2002). The mesothoracic wing and its phylogenetic significance in Cydnidae (Hemiptera: Heteroptera: Pentatomoidea). Pol. J. Entomol..

[13] Lis J.A., Pluot-Sigwalt D. (2002). Nymphal and adult cephalic chaetotaxy of the Cydnidae (Hemiptera: Heteroptera), and its adaptive, taxonomic and phylogenetic significance. Eur. J. Entomol..

[14] Lis J.A., Hohol-Kilinkiewicz A. (2001). Abdominal trichobothrial pattern and its taxonomic and phylogenetic significance in Cephalocteinae (Hemiptera: Heteroptera: Cydnidae). Ann. Zool..

[15] Lis J.A., Hohol-Kilinkiewicz A. (2001). Abdominal trichobothrial pattern and its taxonomic and phylogenetic significance in Cydnidae (Hemiptera: Heteroptera). II. Amnestinae and Garsauriinae. Ann. Zool..

[16] Lis J.A., Hohol-Kilinkiewicz A. (2002). Abdominal trichobothrial pattern and its taxonomic and phylogenetic significance in Cydnidae (Hemiptera: Heteroptera). III. Sehirinae. Ann. Zool..

[17] Lis J.A., Hohol-Kilinkiewicz A. (2002). Adult dorso-abdominal scent glands in the burrower bugs (Hemiptera: Heteroptera: Cydnidae). Pol. J. Entomol..

[18] Lis J.A., Schaefer C.W. (2005). Tibial combs in the Cydnidae (Hemiptera: Heteroptera) and their functional, taxonomic and phylogenetic significance. J. Zool. Syst. Evol. Res..

[19] Pluot-Sigwalt D., Lis J.A. (2008). Morphology of the spermatheca in the Cydnidae (Hemiptera: Heteroptera): Bearing of its diversity on classification and phylogeny. Eur. J. Entomol..

[20] Lis J.A. (2010). Coxal combs in the Cydnidae *sensu lato* and three other related “cydnoid” families—Parastrachiidae, Thaumastellidae, Thyreocoridae (Hemiptera: Heteroptera): Functional, taxonomic, and phylogenetic significance. Zootaxa.

[21] Lis J.A. (2010). Pretarsal structures in the family Parastrachiidae (Hemiptera: Heteroptera: Pentatomoidea). Zootaxa.

[22] Lis J.A., Ziaja D.J. (2010). Pretarsal structures in the family Cydnidae sensu lato (Hemiptera: Heteroptera: Pentatomoidea). Zootaxa.

[23] Gapon D.A. (2018). Morphology of male and female terminalia and taxonomic revision of the burrower bugs genus Canthophorus (Heteroptera: Cydnidae). Ann. Entomol. Soc. Fr..

[24] Kment P., Vilímová J. (2010). Thoracic scent efferent system of Pentatomoidea (Hemiptera: Heteroptera): A review of terminology. Zootaxa.

[25] Lis J.A. (2023). Molecular Apomorphies in the Secondary and Tertiary Structures of Length-Variable Regions (LVRs) of *18S rRNA* Shed Light on the Systematic Position of the Family Thaumastellidae (Hemiptera: Heteroptera: Pentatomoidea). Int. J. Mol. Sci..

[26] Katoh K., Toh H. (2008). Recent developments in the MAFFT multiple sequence alignment program. Brief. Bioinform..

[27] Nguyen L.-T., Schmidt H.A., von Haeseler A., Minh B.Q. (2015). IQ-TREE: A fast and effective stochastic algorithm for estimating maximum likelihood phylogenies. Mol. Biol. Evol..

[28] Darriba D., Taboada G.L., Doallo R., Posada D. (2012). jModelTest 2: More models, new heuristics and parallel computing. Nat. Methods.

[29] Trifinopoulos J., Nguyen L.-T., von Haeseler A., Minh B.Q. (2016). W-IQ-TREE: A fast online phylogenetic tool for maximum likelihood analysis. Nucleic Acids Res..

[30] Hoang D.T., Chernomor O., von Haeseler A., Minh B.Q., Vinh L.S. (2018). UFBoot2: Improving the Ultrafast Bootstrap Approximation. Mol. Biol. Evol..

[31] Ronquist F., Teslenko M., van der Mark P., Ayres D.L., Darling A., Höhna S., Larget B., Liu L., Suchard M.A., Huelsenbeck J.P. (2012). MrBayes 3.2: Efficient bayesian phylogenetic inference and model choice across a large model space. Syst. Biol..

[32] Miller M.A., Pfeiffer W., Schwartz T. (2010). Creating the CIPRES science gateway for inference of large phylogenetic trees. Proceedings of the Gateway Computing Environments Workshop (GCE), New Orleans, LA, USA, 14 November 2010.

[33] Letunic I., Bork P. (2021). Interactive Tree of Life (iTOL) v5: An online tool for phylogenetic tree display and annotation. Nucleic Acids Res..

[34] Maddison W.P., Maddison D.R. (2026). Mesquite: A Modular System for Evolutionary Analysis.

[35] Schuh R.T., Weirauch C. (2020). True Bugs of the World (Hemiptera: Heteroptera). Classification and Natural History.

[36] Tachikawa S., Schaefer C.W. (1985). The biology of *Parastrachia japonensis* (Hemiptera: Pentatomoidea: ?-dae). Ann. Entomol. Soc. Am..

[37] Schaefer C.W., Dolling W.R., Tachikawa S. (1988). The shieldbug genus *Parastrachia* and its position within the Pentatomoidea (Insecta: Hemiptera). Zool. J. Linn. Soc..

[38] Sweet M.H., Schaefer C.W. (2002). Parastrachiinae (Hemiptera: Cydnidae) raised to family level. Ann. Entomol. Soc. Am..

[39] Filippi L., Hironaka M., Nomakuchi S. (2001). A review of the ecological parameters and implications of subsociality in *Parastrachia japonensis* (Hemiptera: Cydnidae), a semelparous species that specializes on a poor resource. Popul. Ecol..

[40] Filippi L., Baba N., Inadomi K., Yanagi T., Hironaka M., Nomakuchi S. (2009). Pre and posthatch trophic egg production in the subsocial burrower bug, *Canthophorus niveimarginatus* (Heteroptera: Cydnidae). Naturwissensch.

[41] Inadomi K., Wakiyama M., Hironaka M., Mukai H., Filippi L., Nomakuchi S. (2014). Postovipositional maternal care in the burrower bug, *Adomerus rotundus* (Hemiptera: Cydnidae). Can. Entomol..

[42] Filippi L., Nomakuchi S. (2022). The Life History of the Parental Shield Bug, Parastrachia japonensis.

[43] Miyamoto S. (1956). Discovery of *Parastrachia japonensis* eggs. Kôntyú.

[44] Southwood T.R.E. (1956). The structure of eggs of the terrestrial Heteroptera and its relationship to the classification of the group. Trans. R. Entomol. Soc. Lond..

[45] Filippi-Tsukamoto L., Nomakuchi S., Kuki K., Tojo S. (1995). Adaptiveness of Parental Care in Parastrachia japonensis (Hemiptera: Cydnidae). Ann. Entomol. Soc. Am..

[46] Kight S.L. (1997). Factors influencing maternal behaviour in a burrower bug, *Sehirus cinctus* (Heteroptera: Cydnidae). Anim. Behav..

[47] Agrawal A.F., Combs N., Brodie E.D. (2005). Insights into the costs of complex maternal care behavior in the burrower bug (*Sehirus cinctus*). Behav. Ecol. Sociobiol..

[48] Hironaka M., Nomakuchi S., Iwakuma S., Filippi L. (2005). Trophic egg production in a subsocial shield bug, *Parastrachia japonensis* Scott (Heteroptera: Parastrachiidae), and its functional value. Ethology.

[49] Mukai H., Hironaka M., Narumi B., Yanagi T., Inadomi K., Filippi L., Nomakuchi S. (2010). Maternal-care behaviour in *Adomerus variegatus* (Hemiptera: Cydnidae). Can. Entomol..

[50] Lis J.A., Domagała P.J. (2024). First reports of parental care in species of the genus *Dismegistus* (Hemiptera: Heteroptera: Parastrachiidae). Eur. J. Entomol..

[51] Amyot C.J.B., Audinet-Serville J.G.A. (1843). Histoire Naturelle des Insectes. Hémiptères.

[52] Froeschner R.C. (1960). Cydnidae of the Western Hemisphere. Proc. U. S. Natl. Mus..

[53] Lis J.A. (1994). A Revision of Oriental Burrower Bugs.

[54] Signoret V. (1884). Révision du Groupe des Cydnides de la Famille des Pentatomides. 13e et derniére partie (1). Ann. Soc. Entomol. Fr..

[55] Gapon D.A. (2021). Revision of the genus *Exosehirus* (Heteroptera: Cydnidae), with the description of two new species. Acta Entomol. Musei Natl. Pragae.

[56] Kment P., Lis J.A. (2025). *Dismegistus madagascariensis* sp. nov., the first representative of the family Parastrachiidae (Hemiptera, Heteroptera) in Madagascar. Deut. Entomol. Zeitschr..

[57] Wagner E. (1963). Untersuchungen über den taxonomischen Wert des Baues der Genitalien bei den Cydnidae (Hem. Het.). Acta Entomol. Musei Natl. Pragae.

[58] Schaefer C.W. (1968). The homologies of the female genitalia in the Pentatomoidea (Hemiptera-Heteroptera). J. N. Y. Entomol. Soc..

[59] Melber A., Hölscher L., Schmidt G.H. (1980). Further studies on the social behaviour and its ecological significance in *Elasmucha grisea* L. (Hem.-Het.: Acanthosomatidae). Zool. Anz..

[60] Kudo S., Sato M., Ohara M. (1989). Prolonged maternal care in *Elasmucha dorsalis* (Heteroptera: Acanthosomatidae). J. Ethol..

[61] Kudo S., Nakahira T. (1993). Brooding behavior in the bug *Elasmucha signoreti* (Heteroptera: Acanthosomatidae). Psyche.

[62] Kaitala A., Mappes J. (1997). Parental care and reproductive investment in shield bugs (Acanthosomatidae, Heteroptera). Oikos.

[63] Gogala M., Yong H., Brühl C. (1998). Maternal care in *Pygoplatys bugs* (Heteroptera: Tessaratomidae). Eur. J. Entomol..

[64] Kudo S. (2000). The guarding posture of females in the subsocial bug *Elasmucha dorsalis* (Heteroptera: Acanthosomatidae). Eur. J. Entomol..

[65] Tsai J.F., Kudo S., Yoshizawa K. (2015). Maternal care in Acanthosomatinae (Insecta: Heteroptera: Acanthosomatidae)—Correlated evolution with morphological change. BMC Evol. Biol..

[66] Kudo S., Ogasa W. (2017). Notes on maternal care in *Elasmucha japonica* and *Acanthosoma forcula* (Heteroptera: Acanthosomatidae). Jpn. J. Entomol..

[67] Kudo S.I., Harano T., Tsai J.F., Yoshizawa K., Kutsukake N. (2024). Maternal Care under Large Clutches with Small Eggs: The Evolution of Life History Traits in Shield Bugs. Am. Nat..

[68] Kearns R.S., Yamamoto R.T. (1981). Maternal behavior and alarm response in the eggplant lace bug, *Gargaphia solani* Heidemann (Tingidae: Heteroptera). Psyche.

[69] Tallamy D.W., Denno R.F. (1981). Maternal care in *Gargaphia solani* (Hemiptera: Tingidae). Anim. Behav..

[70] Tallamy D.W., Schaefer C., Choe J.C., Crespi B.J. (1997). Maternal care in the Hemiptera: Ancestry, alternatives, and current adaptive value. The Evolution of Social Behavior in Insects and Arachnids.

[71] Guidoti M., Tallamy D.W., Marsaro A.L. (2015). Maternal care in *Gargaphia decoris* (Heteroptera, Tingidae), with comments on this behavior within the genus and family. Rev. Bras. Entomol..

[72] Lis J.A., Domagała P.J. (2026). Nymphal and adult cephalic chaetotaxy in two genera of Parastrachiinae (*Parastrachia* and *Dismegistus*).

[73] Leston D. (1956). The Ethiopian Pentatomoidea (Hemiptera): XXII, On *Dismegistus* Amyot and Serville (Cydnidae). Proc. R. Entomol. Soc. Lond. Ser. A Gen. Entomol..

[74] Wagner E. (1966). Die Tierwelt Deutschlands Teil 54. Wanzen Oder Heteropteren. I. Pentatomorpha.

[75] Dolling W.L. (1981). A rationalised classification of the burrower bugs (Cydnidae). Syst. Entomol..

[76] Matesco V.C., Grazia J. (2013). Revision of the genus *Alkindus* Distant (Hemiptera: Heteroptera: Thyreocoridae: Corimelaeninae). Zootaxa.

[77] Schwertner C.F., Nardi C., Panizzi A.R., Grazia J. (2015). Burrower Bugs (Cydnidae). True Bugs (Heteroptera) of the Neotropics.

[78] McAtee W., Malloch J. (1933). Revision of the subfamily Thyreocorinae of the Pentatomidae (Hemiptera-Heteroptera). Ann. Carnegie Mus..

[79] Štys P., Davidová J. (1979). Taxonomy of *Thyreocoris* (Heteroptera, Thyreocoridae). Annot. Zool. Bot..

[80] Davidová-Vilímová J., Podoubsky M. (1999). Larval and adult dorso-abdominal scent glands and androconia of central European Pentatomoidea (Heteroptera). Acta Soc. Zool. Bohem..

[81] Mulsant E., Rey C. (1866). Histoire Naturelle des Punaises de France. II Tribu: Pentatomides.

[82] Lis J.A. (1999). Burrower bugs of the Old World—A catalogue (Hemiptera: Heteroptera: Cydnidae). Genus.

[83] Lis J.A., Lis B., Ziaja D., Dobosz R. (2014). Description and DNA barcoding of *Ochetostethomorpha secunda*, a new species of the South African endemic burrower bug genus (Hemiptera: Heteroptera: Cydnidae) from Namibia. Zootaxa.

[84] Linnavuori R.E. (1993). Cydnidae of West, Central and North-East Africa (Heteroptera). Acta Zool. Fenn..

[85] Lis J.A., Lis B. (2022). *Geotomus granulosus*, a peculiar sehirine-like new species of the subfamily Cydninae (Hemiptera: Heteroptera: Cydnidae) from Burundi. Zootaxa.

[86] Oshanin B. (1922). Sur les genres de la tribu des Strachiaria Put. (Heteroptera, Pentatomidae). Ezhegodnik Zool. Muzeya Ross. Akad. Nauk..

[87] Lis J.A., Lis P., Ziaja D.J., Kocorek A. (2012). Systematic position of Dinidoridae within the superfamily Pentatomoidea (Hemiptera: Heteroptera) revealed by the Bayesian phylogenetic analysis of the mitochondrial 12S and 16S rDNA sequences. Zootaxa.

[88] Lis J.A., Kocorek A., Ziaja D.J., Lis P. (2015). New insight into the systematic position of the endemic Madagascan genus *Amberiana* (Hemiptera: Heteroptera: Dinidoridae) using 12S rDNA sequences. Turk. J. Zool..

[89] Wu Y.Z., Yu S.S., Wang Y.H., Wu Y.H., Li X.R., Men X.Y., Zhang Y.W., Rédei D., Xie Q., Bu W.J. (2016). The evolutionary position of Lestoniidae revealed by molecular autapomorphies in the secondary structure of rRNA besides phylogenetic reconstruction (Insecta: Hemiptera: Heteroptera). Zool. J. Linn. Soc..

[90] Robertson I.A.D. (2009). The Pentatomoidea (Hemiptera: Heteroptera) of Sub-Saharan Africa. A Database.

[91] Pluot-Sigwalt D., Lis J.A. (2018). Presence of uradenia in male adults of the genus *Dismegistus* (Hemiptera: Heteroptera: Parastrachiidae). Acta Entomol. Musei Natl. Pragae.

[92] Kumar R. (1974). A revision of World Acanthosomatidae (Heteroptera: Pentatomoidea): Keys to and descriptions of subfamilies, tribes and genera with designation of types. Aust. J. Zool..

[93] Gapud V.P. (1991). A generic revision of the subfamily Asopinae, with consideration of its phylogenetic position in the family Pentatomidae and superfamily Pentatomoidea (Hemiptera-Heteroptera). Philipp. Entomol..

[94] Leston D. (1953). Notes on the Ethiopian Pentatomoidea (Hem.). XVI, An Acanthosomatid from Angola, with remarks upon the status and morphology of Acanthosomatidae Stål. Publ. Cult. Comp. Diam. Angola.

[95] Leston D. (1953). On the wing-venation, male genitalia and spermatheca of *Podops inuncta* (F.), with a note on the diagnosis of the subfamily Podopinae Dallas (Hem., Pentatomidae). J. Soc. Br. Entomol..

[96] Leston D. (1953). Phloeidae Dallas (Hem. Pentatomoidea): Systematics and morphology, with remarks on the phylogeny of Pentatomoidea Leach and upon the position of *Serbana* Distant. Rev. Bras. Biol..

[97] Leston D. (1954). Notes on the Ethiopian Pentatomoidea (Hem.). XII, On some specimens from Southern Rhodesia, with an investigation of certain features in the morphology of *Afrius figuratus* (Germar) and remarks upon the male genitalia in Amyotinae. Occas. Pap. Natl. Mus. South. Rhod..

[98] Leston D. (1954). Wing venation and male genitalia of *Tessaratoma* Berthold, with remarks on Tessaratominae Stål (Hemiptera: Pentatomidae). Proc. R. Entomol. Soc. Lond. Ser. A Gen. Entomol..

[99] Ribaut H. (1923). L’urite IX des mâles chez les Pentatomides. Bull. Soc. Hist. Nat. Toulouse.

[100] Lis J.A., Aukema B., Rieger C. (2006). Parastrachiidae Oshanin, 1922. Catalogue of the Heteroptera of the Palaearctic Region, Volume 5, Pentatomomorpha II.

