# Supplementary material for: Sehiridae stat. nov., the Highly-Supported Monophylum Within the ‘Cydnoid’ Complex of Pentatomoid Families (Hemiptera: Heteroptera: Pentatomoidea)
Source: Insects. 2026 Aug 12;17(8):835. doi: 10.3390/insects17080835 (PMC13512964; doi:10.3390/insects17080835)
Supplement: Supplementary file 1 [file insects-17-00835-s001.zip › File S1.nexus.pdf]

```
#NEXUS
[ TITLE 18S+28S+16S ]
begin data;
   dimensions ntax=100 nchar=2772;
   format missing=? gap=- matchchar=. datatype=dna;
   matrix

[!Domain=Data;]
Acanthosoma_nigrodorsum
ATGCAAGTCTCAGTACGAGCTGACT-
TAAAGTGAAACCGCAAAAGGCTCAGTAAACCGGTCATAGTTTACCGGA-TATATG-CCACTC-
ACGTGGACAACTGTGGTAATTCTAGAGCTAATACATGCAACC-AAGTCCCGACCAGCG---
ATGGAAAGGACGCTTTTATTGGATCAAAACCATTCGCCCGGCCCCGCCCGGGC------
GTCTGCTTGATGACTCT-AGATAAC-ACCACA-GATCGCACGGTCATAGTACCGGC-
GACGTATCCGTCAAATGTCTGACTTATCAGCTGTCGATGGTAGACTCTGTGTCTACCATGGCGCTCACGGGTA
ACGGGGAATCAGGGTTCGATTCCGGAGAGGGAGCCTGAGAAACGGCTACCACATCCAAGGAAGGCAGCAGGCA
CGCAAATTACCCACTCCCGGCACGGGGAGGTAGTGACAAAAAATAACGATACGGGACTCATCCGAGGCCCCGT
AATCGGAATGAGAACACCCTAAATCCTTTAACGAGGATCTATTGGAGGGCAAGTCTGGTGCCAGCAGCCGCGG
TAATTCCAGCTCCAATAGCGTATATTAAAGTTGTTGCGGTTAAAAA-
GCTCGTAGTTGGTTCTGCGTGCCACGCTGTCGGTTCACCGCCTGTCGGTGCAACTGGCATGCC-
GTGGCATGTCCTGTCGGTGGCTTCACAGGCTTCGTG-GCGCGCCCTATG-------CGTT---AACACGT-G-
TAGGTAGTTGC--GCCGC-GTTGTTC-TTGTAGG-CCGGCT-CAATCCTGCCGCGGTGCT-
CTTTACCGAGTGTCGAGGTAGGCCGACACT-
GTTTACTTTGAACAAATTAGAGTGCTTCAAGCAGGCTCTGAAAGC-
AGCCTGAATAGTGGTGCATGGAATAATGGAACAGGACCTTGGTTCTATTTTGTT-GGTTTTAGGA-
ATCCAAGGTAATGATCAATAAGGA-CAGGCGGGGGCATTCGTATTGCGACGTTAGA-----------------
-GGTGAAATTCTTGG-
ATCGTCGCAAGACGCACTAGAGCGAAAGCATTTGCCAAGTATGTCTTAATTGATCAAGAACGAAAGTTAGAGG
TTCGAAGGCGATCAGATACCGCCCTAGTTCTAACCATAAACGATGCCAGCCAGCGATCCGCCGATGTTCCTCC
GATGACTCGGCGGGGAGCTT-
TCCCCGGGAAACCAAAGCTTTTGGGTTCCGGGGGAAGTATGGTTGCAAAGCTGAAACTTAAAGGAATTGACGG
AAGGGCACCACCAGGAGTGGAGCCTGCGGCTTAATTTGACTCAACACGGGAAACCTCACCAGGCCCGGACATT
GGAA-GGATTGACAGAT-CGATAGCTCTTT-CTCGATT-CAGTGGGT-
AGTGGTGCATGGCCGTTCTTAGTTGGTGGACTGATTTGTCTGGTTAATTCCGATAACGAACGAGACTCTGACC
TATTAACTAGGCGCTC-CCGGTATAC-ATA-CCTACCGGCGAGAT-
TTATTCTTCTTAAAGGGACAGGCGGCTCTTAGCCGCACGAGATTGAGCAATAACAGGTCTGTGATGCCCTTAG
ATGTTCTGGGCCGCACGCGCGCTACACTGAAGGAATCAGCGTGTGC--
TCCCCTGTCCGAAAGGACCGGGCAACCCGCTGAACCTCCTTCGTGCTAGGAATTGGGGCTTGCAATTGTTCCC
CATGAACGAGGAATTCCCAGTAAGCGCGAGTCATAAGCTCGCGTTGATTACGTCCCTGCCCTTTGTACACACC
GCCCGTCGCTACTACCGATTGAATGATTTAGTGAGGTCTTCGGACC-GGCGGTA--AC-CGGC-ACC--
GGCA-----CGTTCGG---TATCGGT-TTCGCTGGAAA-
GATGACCGAACTTGATCATTTAGAGGAAGTAAAAGTCGTAACAAGG-TTTCCAGTCA--TTGGGTTCT--
AAAACCTAAAGGCGAAATGAAAGT-GTC-------------AGGGGGGCCTCCT-------------
CAGGGAGG-----TCTCCCCACAGGGAGGATCGGCTTCC----GTACAATTA-C--------TGT--
CGGAGCCCCGCACTCCCGGGGCGTCTC--GTTCTC-ATCACG-AG----
TAGAGACGCACCAGGAGCGCACACGCTGGTACCCGAAAGATGGTGAACTATGCCTGGCCAGGACGAAGCCAGG
GGAAACCCTGGTGGAGGTCC--GAAGCG-ATTCTG--ACGTGCAAATCG-
ATCGTATGAGCTGGGTATAGGGGCGAAAGACCAATCGAACCATCT----GTTTATTT--
ATATTAAGTCTTGCCTGCTCAATGATATTTATT-----
ATATTAAATAGCCGCAGTATATTGACTGTGCAAAGGTAGCATAATCATTTGTCTTTTAATTGGAGACTTGTAT
GAATGGTTTGATGAGATATTGACTTTCTTTA---TATAAAATTAA--
TTAACTTAATATTTTAGTTAAAAAGCTAAAATACTT-TTAAGGGACGAGAAGACCCTATAGAA--
TTTTATACTTTT-GAA---------TT-TAATTTATATTTGGATATTTTTG----A-ATTTTATCTTTAAA--
--AGTATTTTGTTGGGGTGACAGGGAAA-TTTTCTTAACTTTCTTTATTTATACT---
TTATTAATATATATAT-----TTATGATCCTA-----
TTTTATGGATATTTAAAGATTAAATTACCTTAGGATAAC
Adelphocoris_lineolatus
```

ATGCAAGTCTCAGTACAAACTGTAT-
TAAAGTGAAACCGCAAAAGGCTCAGTAAACCAGTCATGATTCACTCGAGTATACA-
TGACTGTCTTTGGATAACTGTGGTAATCCTAGAGCTAATACATGCAACC-
AAGTCCCGACCAGTCTGACTGGGAAGGACGCTTTTATTAGATCAAGACCAATCGCCCGG-CTCGTCCGGGC--
----GTTGCCTTGGTGACTCT-AGGTAAC-ACATCA-GATCGCATGGTCTCAGCACCGGC-
GACTTATCCTTCAAATGCCTGACTTATCAACTGTCGATGGTAGACTCCATGTCTACCATGGTTGTTACGGGTA
ACGGGGAATCAGGGTTCGATTCCGGAGAGGGAGCCTGAGAAACGGCTACCACATCCAAGGAAGGCAGCAGGCA
CGCAAATTACCCACTCCCGGCACGGGGAGGTAGTGACAAAAAATAACGATACGGGACTCTTATGAGGCCCCGT
AATCGGAATGAGTACACTTTAAATCCTTTAACAAGGATCCATTGAAGGGCAAGTCTGGTGCCAGCAGCCGCGG
TAATTCCAGCTTCAATAGCGTATATTAAAGTTGTTGCGGTTAAAAA-
GCTCGTAGTTGGTTCTGCGTGCTACGCTGTCGGTTCGCCGCCTGCCGGTGTAACTGGCATGCC-
GTGGCATGTCCTGCCGGTGGCGTGCGGGA-GCCTTGCCAGGTGACGGC----------CCGGGTTCG-CT---
CGGTGCCGTAGG-CCGGAG-GTCCAACCCGTGCG-CCTACT-CTATCCTACCTAGGTGCT-
CTTCACCGAGTGTCGAGGTAGGCCGGTAC--
GTTTACTTTGAACAAATTAGATTGCTCAAAGCAGGCTGAAATCTC-
TGCCTGAATAGTGGTGCATGGAATAATAAAACAGGACCTTGGTTCTATTTTGTTGGTTCTTAGGA-
ACACAAGGTAATGATCAATACGGA-CAGGCGGGGGCATTCGTATTGCGACGTTAGA-----------------
-GGTGAAATTCTTGG-
ATCGTCGCAAGACGCACAAGAGCGAAAGCATTTGCCAAGTATGTCTTGATTGATCAAGAACGAAAGTTAGAGG
TTCGAAGGCGATCAGATACCGCCCTAGTTCTAACCATAAACGATGCCAGCCAGCGATCCGCCGATGTTCCTCC
GATGACTCGGCGGGGAGCTTCTTCCCGGGAAACCAAAGCTTTTGGGTTCCGGGGGAAGTATGGTTGCAAAGCT
GAAACTTAAAGGAATTGACGGAAGGGCACCACCAGGAGTGGAGCCTGCGGCTTAATTTGACTCAACACGGGAA
ACCTCACCAGGCCAGGACATTGGAA-GGATTGACAGAT-TGATAGCTCTTT-CTTGATT-CAGTGGGT-
AGTGGTGCATGGCCGTTCTTAGTTGGTGGATCGATTTGTCTGGTTAATTCCGATAACGAACGAGACTCTAGCC
TATTAACTAGGCGTTTTCCGGTGTCC---CCGCCACCGGCGACAA-
TCATTCTTCTTAAGGGGACAGGCGGCTTATAGCCGCACGAGATTGAGCAATAACAGGTCTGTGATGCCCTTAG
ATGTCCTGGGCCGCACGCGCGCTACACTGAAGGAATCAGCGTGTGC-CTCCCAGA-
CCGAAAGGCCCGGGTAATCCGCTGAACCTCCTGCGTGCTAGGAATTGGGGCTTGCAATTGTTCCCCATGAACG
AGGAATTCCCAGTAAGCGCGAGTCATAAGCTCGCGTTGATTACGTCCCTGCCCTTTGTACACACCGCCCGTCG
CTACTACCGATTGAATGATTTAGTGAGGTCTTCGGACT-GGCGGTAATCCCTATCGACT----------TGT-
-CGG---TATCGATGAACGCTGGAAA-
GATGACCAAACTTGATCATTTAGAGGAAGTAAAAGTCGTAACAAGG-TTTCCAGTCA--TTGGGTGT--
CAAAACCTAAAGGCGAAATGAAAGT-----------------GAAGGAAACTTTGT-----------------
------------TTTCGAGGGAGGATGGGTTAG----------GTCCCTCTCCAGGGCCGG------
CCTCGCACTCCCGGGGCGTCTC--TATCTC-ATCACG-AG----
AGGAGGCGCACCTAGAGCGTACACGCTGATACCCGAAAGATGGTGAACTATGCCTGGCCAGGACGAAGCCAGG
GGAAACCCTGGTGGAGGTCC--GCAGCG-ATTCTG--ACGTGCAAATCG-
ATCGTCGGAGCTGGGTTTAGGGGCGAAAGACCAATCGAACCATCTG-------
TTTTAAATAAAAGATTTAACCTGCTCTATGATT---------A----
TTAAATGGCTGCAGTATTTTGACTGTACAAAGGTAGCATAATAATTTGTCTTTTAATTGAAGACTGGTATGAA
TGGTTGTATGAGAAATATACTTTCTTTATTTTA--TATAAAA----
AAATTGATTTTTTAGTTAAAAAGCTAAAATATTT-TTATAGGACGAGAAGACCCTATAGAA--
CTTTACTTTAA--------------TATAAATATACTATTTATATATTTTT---AAAATTTTA-
TATTTTTATTA-AAGTTTTGTTGGGGTGACAAAAAAAATT-ATATAACTT-TTTTTAT--TAAA---
TCATTAATTTATGTTTT--TTT---GATCCTT-----TATTATGGAAA--
ATAAGATTAAGTTACCTTAGGGATAAC
Adomerus_biguttatus
ATGCAAGTCTCAGTACGAGCTTACT-
TAAAGTGAAACCGCAAAAGGCTCAGTAAACCGGTCATAGTTTACCGAA-TATATG-CCACTC-
ACGTGGATAACTGTGGTAATTCTAGAGCTAATACATGCAACA-AAGTCCCGACCAGCG---
ATGGAAAGGACGCTTTTATTGGATCAAAACCATTCGCCCGGTTC-GCCCGGGC------
GTCTGCTTGATGACTCT-AGATAACCACAACA-GATCGCACGGTCACTGTACCGGC-
GACGTATCCGTCAAATGTCTGACTTATCAGCTGTCGATGGTAGACTCTGTGTCTACCATGGCGCTCACGGGTA
ACGGGGAATCAGGGTTCGATTCCGGAGAGGGAGCCTGAGAAACGGCTACCACATCCAAGGAAGGCAGCAGGCA
CGCAAATTACCCACTCCCGGCACGGGGAGGTAGTGACAAAAAATAACGATACGGGACTCATCCGAGGCCCCGT
AATCGGAATGAGAACACCCTAAATCCTTTAACGAGGATCTATTGGAGGGCAAGTCTGGTGCCAGCAGCCGCGG
TAATTCCAGCTCCAATAGCGTATATTAAAGTTGTTGCGGTTAAAAA-
GCTCGTAGTTGGTTCTGCGTGCCACGCTGTCGGTTCACCGCCTGTCGGTGCAACTGGCATGCC-

GTGGCATGTCCTGTCGGTGGCCTACTGGT-TTCGCG-GCGCACCCTATG-------CGTT---CACTCGT-G-
TAGGTCGTTGC--GCTGC-GTTGTCC-TCGTAGG-CCGGCT-CAATCCTACCGCGGTGCT-
CTTTACCGAGTGTCGAGGTAGGCCGACACT-
GTTTACTTTGAACAAATTAGAGTGCTTCAAGCAGGCTCTGAAAGC-
AGCCTGAATAGTGGTGCATGGAATAATGGAACAGGACCTTGGTTCTATTTTGTT-GGTTTTCGGA-
ATCCAAGGTAATGATCAATAAAGA-CAGGCGGGGGCATTCGTATTGCGACGTTAGA-----------------
-GGTGAAATTCTTGG-
ATCGTCGCAAGACGCACTAGAGCGAAAGCATTTGCCAAGTATGTCTTAATTGATCAAGAACGAAAGTTAGAGG
TTCGAAGGCGATCAGATACCGCCCTAGTTCTAACCATAAACGATGCCAGCCAGCGATCCGCCGATGTTCCTCC
GATGACTCGGCGGGGAGCTT-
CCCCCGGGAAACCAAAGCTTTTGGGTTCCGGGGGAAGTATGGTTGCAAAGCTGAAACTTAAAGGAATTGACGG
AAGGGCACCACCAGGAGTGGAGCCTGCGGCTTAATTTGACTCAACACGGGAAACCTCACCAGGCCCGGACATT
GGAA-GGATTGACAGAT-CGATAGCTCTTT-CTCGATT-CAGTGGGT-
AGTGGTGCATGGCCGTTCTTAGTTGGTGGACTGATTTGTCTGGTTAATTCCGATAACGAACGAGACTCTGACC
TATTAACTAGGCGCTC-CCGGTATAC-ATA-CCTACCGGCGAGAC-
TTATTCTTCTTAAAGGGACAGGCGGCTCTTAGCCGCACGAGATTGAGCAATAACAGGTCTGTGATGCCCTTAG
ATGTTCTGGGCCGCACGCGCGCTACACTGAAGGAATCAGCGTGTTC--
TCCCCTGTCCGAAAGGACCGGGCAACCCGCTGAACCTCCTTCGTGCTAGGAATTGGGGCTTGCAATTGTTGCC
CATGAACGAGGAATTCCCAGTAAGCGCGAGTCATAAGCTCGCGTTGATTACGTCCCTGCCCTTTGTACACACC
GCCCGTCGCTACTACCGATTGAATGATTTAGTGAGGTCTTCGGACC-GGCGGTA--AC-AGGC-ATA--
GGTT-----CGCCCTT---TATCTGT-TCCGCTGGAAA-
GATGACCGAACTTGATCATTTAGAGGAAGTAAAAGTCGTAACAAGG-TTTCCAGTCA--TTGGGTGT---
AAA-CCTAAAGGCGCAATGAAAGT-GTC-------------AGGGGGTCCTCCT-------------
AAGGGAGG----ATCTCCCCGCTGGGAAGATGGGCCACC----GTACAATTACT--------TGT--
CGGAGCCCCGCACTCCCGGGGCGTCTC--GTTCTC-ATTGCG-AG----
TAGAGACGCACCAGGAGCGCACACGCTGGTACCCGAAAGATGGTGAACTATGCCTGGCCAGGACGAAGCCAGG
GGAAACCCTGGTGGAGGTCC--GAAGCG-ATTCTG--ACGTGCAAATCG-
ATCGTCTGAGCTGGGTATAGGGGCGAAAGACCAATCGAACCATCT-GTTAT--TT----
ATTTTAAGTCAAACCTGCTCAATGATAT--TA------
TTATTAAATAGCCGCAGTATTTTGACTGTGCAAAGGTAGCATAATCATTTGTCTATTAATTGTAGGCTGGTAT
GAATGGTTGGATGAAATATTGACTTTCTTTATTTT-TATAATTAA--
TGAATTTAATATTTAAGTTAAAAAGCTTAAATAATT-TTGTAGGACGAGAAGACCCTGTAGAA--
TTTTATTTAAAT--TT--------TAATTAATTGTAATTTTATT-A--ATT----ATATT-TATTAATTCT--
--TAAATTTTGTTGGGGTGACAGGGAAA-ATCTAATAACTTTCTTTATT-ATTACTT-
TTATTAATTAATATAT-----TAAAAATCCTT-----TATTAAAGAA---
GTAAGATTAAATTACCTTAGGGATAAC
Adomerus_variegatus
ATGCAAGTCTCAGTACGAGCTTACT-
TAAAGTGAAACCGCAAAAGGCTCAGTAAACCGGTCATAGTTTACCGGA-TATAAG-CCACTC-
ACGTGGATAACTGTGGTAATTCTAGAGCTAATACATGCAACC-AAGTCCCGACCAGCG---
ATGGAAAGGACGCTTTTATTGGATCAAAACCATTCGCCCGGTTC-GCCCGGGC------
GTCTGCTTGATGACTCT-AGATAAC-ACCACA-GATCGCACGGTCATTGTACCGGC-
GACGTATCCGTCAAATGTCTGACTTATCAGCTGTCGATGGTAGACTCTGTGTCTACCATGGCGCTCACGGGTA
ACGGGGAATCAGGGTTCGATTCCGGAGAGGGAGCCTGAGAAACGGCTACCACATCCAAGGAAGGCAGCAGGCA
CGCAAATTACCCACTCCCGGCACGGGGAGGTAGTGACAAAAAATAACGATACGGGACTCATCCGAGGCCCCGT
AATCGGAATGAGAACACCCTAAATCCTTTAACGAGGATCTATTGGAGGGCAAGTCTGGTGCCAGCAGCCGCGG
TAATTCCAGCTCCAATAGCGTATATTAAAGTTGTTGCGGTTAAAAA-
GCTCGTAGTTGGTTCTGCGTGCCACGCTGTCGGTTCACCGCCTGTCGGTGCAACTGGCATGCC-
GTGGCATGTCCTGTCGGTGGCCTACTGGT-TTCGCG-GCGCACCCTATG-------CGTT---CACTCGT-G-
TAGGTCGTTGC--GCTGC-GTTGTCC-TCGTAGG-CCGGCT-CAATCCTACCGCGGTGCT-
CTTTACCGAGTGTCGAGGTAGGCCGACACT-
GTTTACTTTGAACAAATTAGAGTGCTTCAAGCAGGCTCTGAAAGC-
AGCCTGAATAGTGGTGCATGGAATAATGGAACAGGACCTTGGTTCTATTTTGTT-GGTTTTCGGA-
ATCCAAGGTAATGATCAATAAAGA-CAGGCGGGGGCATTCGTATTGCGACGTTAGA-----------------
-GGTGAAATTCTTGG-
ATCGTCGCAAGACGCACTAGAGCGAAAGCATTTGCCAAGTATGTCTTAATTGATCAAGAACGAAAGTTAGAGG
TTCGAAGGCGATCAGATACCGCCCTAGTTCTAACCATAAACGATGCCAGCCAGCGATCCGCCGATGTTCCTCC
GATGACTCGGCGGGGAGCTT-

CCCCCGGGAAACCAAAGCTTTTGGGTTCCGGGGGAAGTATGGTTGCAAAGCTGAAACTTAAAGGAATTGACGG
AAGGGCACCACCAGGAGTGGAGCCTGCGGCTTAATTTGACTCAACACGGGAAACCTCACCAGGCCAGGACATT
GGAA-GGATTGACAGAT-CGATAGCTCTTT-CTCGATT-CAGTGGGT-
AGTGGTGCATGGCCGTTCTTAGTTGGTGGACTGATTTGTCTGGTTAATTCCGATAACGAACGAGACTCTGACC
TATTAACTAGGCGCTC-CCGGTATAC-ATA-CCTACCGGCGAGAC-
TTATTCTTCTTAAAGGGACAGGCGGCTCTTAGCCGCACGAGATTGAGCAATAACAGGTCTGTGATGCCCTTAG
ATGTTCTGGGCCGCACGCGCGCTACACTGAAGGAATCAGCGTGTTC--
TCCCCTGTCCGAAAGGACCGGGCAACCCGCTGAACCTCCTTCGTGCTAGGAATTGGGGCTTGCAATTGTTGCC
CATGAACGAGGAATTCCCAGTAAGCGCGAGTCATAAGCTCGCGTTGATTACGTCCCTGCCCTTTGTACACACC
GCCCGTCGCTACTACCGATTGAATGATTTAGTGAGGTCTTCGGACC-GGCGGTA--AC-AGGC-ATC--
GGCT-----CGCCCTT---TATCTGT-TCCGCTGGAAA-
GATGACCGAACTTGATCATTTAGAGGAAGTAAAAGTCGTAACAAGG-TTTCCAGTCA--TTGGGTGT---
AAA-CCTAAAGGCGCAATGAAAGT-GTC-------------AGGGGGTCCTCCC-------------
ACGGGAGG----ATCTCCCCGCTGGGAAGATGGGCCACC----GTACAATTATT--------TGT--
CGGAGCCCCGCACTCCCGGGGCGTCTC--GTTCTC-ATTGCG-AG----
TAGAGACGCACCAGGAGCGCACACGCTGGTACCCGAAAGATGGTGAACTATGCCTGGCCAGGACGAAGCCAGG
GGAAACCCTGGTGGAGGTCC--GAAGCG-ATTCTG--ACGTGCAAATCG-
ATCGTCTGAGCTGGGTATAGGGGCGAAAGACCAATCGAACCATCT-GTTATATTT----
ATTTTAGGTCAAGCCTGCTCAATGAT----TA------
TTATTAAATAGCCGCAGTATTTTGACTGTGCAAAGGTAGCATAATCATTTGTCTATTAATTGTAGGCTGGTAT
GAATGGTTAGATGAAATATTGACTTTCTTTGCTCTATATAATT-----
GAATTTAATATTTAAGTTAAAAAGCTTAAATGGTT-TTGTAGGACGAGAAGACCCTATAGAA--
TTTTATTTAATT--AT--------TAATTTGTTATGATTTAGTT-ATTATT----GTATA-AATTGGTGAT--
--TAAATTTTGTTGGGGTGACAGGGAAA-ATTTAATAACTTTCTTTATTTATTA--T-
TTATTAATTGGTATAT-----TAAAGATCCTT-----TATTTAAGGATT-
ATAAGATTAAATTACCTTAGGGATAAC
Adrisa_birmana
ATGCAAGTCTCAGTACGAGCTGACT-
TAAAGTGAAACCGCAAAAGGCTCAGTAAACCGGTCATAGTTTACCGGA-TATATG-CCACTC-
ACGTGGATAACTGTGGTAATTCTAGAGCTAATACATGCAACC-AAGTCCCGACCAGCG---
ATGGAAAGGACGCTTTTATTGGATCAAAACCATTCGCCCGGCTC-GCCCGGGC------
GTCTGCTTGATGACTCT-AGATAAC-ACCACA-GATCGCACGGTCACAGTACCGGC-
GACGTATCCGTCAAATGTCTGACTTATCAGCTGTCGATGGTAGACTCTGTGTCTACCATGGCGCTCACGGGTA
ACGGGGAATCAGGGTTCGATTCCGGAGAGGGAGCCTGAGAAACGGCTACCACATCCAAGGAAGGCAGCAGGCA
CGCAAATTACCCACTCCCGGCACGGGGAGGTAGTGACAAAAAATAACGATACGGGACTCATCCGAGGCCCCGT
AATCGGAATGAGAACACCCTAAATCCTTTAACGAGGATCTATTGGAGGGCAAGTCTGGTGCCAGCAGCCGCGG
TAATTCCAGCTCCAATAGCGTATATTAAAGTTGTTGCGGTTAAAAA-
GCTCGTAGTTGGTTCTGCGTGCCACGCTGTCGGTTCACCGCCTGTCGGTGCAACTGGCATGCC-
GTGGCATGTCCTGTCGGTGGCTGACAGGC-TTCGCG-GCGCGCCCTATG-------CGTTAAC-GCGTGT---
-AGGTCGTTGC--GCCG-CGTTGTCC-TTGTAGG-CCGGCT-CAATCCTACCGCGGTGCT-
CTTTGCCGAGTGTCGAGGTAGGCCGACACT-
GTTTACTTTGAACAAATTAGAGTGCTTAAAGCAGGCTCTGAAAGC-
AGCCTGAATAGTGGTGCATGGAATAATGGAACAGGACCTTGGTTCTATTTTGTT-GGTTTTCTGA-
ATCCAAGGTAATGATCAATAAAGA-CAGGCGGGGGCATTCGTATTGCGACGTTAGA-----------------
-GGTGAAATTCTTGG-
ATCGTCGCAAGACGCACTAGAGCGAAAGCATTTGCCAAGTATGTGTTAATTGATCAAGAACGAAAGTTAGAGG
TTCGAAGGCGATCAGATACCGCCCTAGTTCTAACCATAAACGATGCCAGCCAGCGATCCGCCGATGTTCCTCC
GATGACTCGGCGGGGAGCTT-
TCCCCGGGAAACCAAAGCTTTTGGGTTCCGGGGGAAGTATGGTTGCAAAGCTGAAACTTAAAGGAATTGACGG
AAGGGCACCACCAGGAGTGGAGCCTGCGGCTTAATTTGACTCAACACGGGAAACCTCACCAGGCCCGGACATT
GGAA-GGATTGACAGAT-CGATAGCTCTTT-CTCGATT-CAGTGGGT-
AGTGGTGCATGGCCGTTTTTAGTTGGTGGACTGATTTGTCTGGTTAATTCCGATAACGAACGAGACTCTGGCC
TATTAACTAGGCGCTC-CCGGTATAA-ATA-CCTACCGGCGAGAT-
TTATTCTTCTTAAGGGGACAGGCGGCTCTCAGCCGCACGAGATTGAGCAATAACAGGTCTGTGATGCCCTTAG
ATGTTTTGGGCCGCACGCGCGCTACACTGAAGGAATCAGCGTGTGC--
TCCCCTGTCCGAAAGGACCGGGCAACCCGCTGAACCTCCTTCGTGCTAGGAATTGGGGCTTGCAATTGTTCCC
CATGAACGAGGAATTCCCAGTAAGCGCGAGTCATAAGCTCGCGTTGATTACGTCCCTGCCCTTTGTACACACC
GCCCGTCGCTACTACTGATTGGATGATTTAGTGAGGTCTTCGGACC-GGCGGCA--AC-CGGC-ACC--GGC-

----TCGTCCGG---TATCGGT-TTCGCGGGAAA-
GATGACCGAACTTGATCATCTAGAAGAAGTAAAAGTCGTAACGAGG-TTTCCAGTCA--TTGGGTGC---
AAA-CCTAAAGGCGCAATGAAAGT-GTCAGA-----------GGGGGGCCTCCTC-------------
CGGGAGG-----TCTCTCCGCAGGGAGGATGGGCCTCC----GTGCAATTATT--------TGC--
CGGAGCCCCGCACTCCCGGGGCGTCTC--GTTCTC-ATTACG-AG----
TAGAGACGCACCAGGAGCGCACACGCTGGTATCCGAAAGATGGTGAACTATGCCTGGCCAGGACGAAGCCAGG
GGAAACCCTGGTGGAGGTCC--GAAGCG-ATTCTG--ACGTGCAAATCG-
ATCGTCTGAGCTGGGTATAGGGGCGAAAGACCAATCGAACCATCTTG-TTAAAGTTT--
ATATCAAGTCTGGCCTGCTCAATGATTTT-------
GGTGATTTAATAGCCGCAGTATTTTGACTGTGCAAAGGTAGCATAATCATTTGTCTTTTAATTGTAGGCTTGT
ATGAATGGTTTGATGAGATACTAACTTTCTTTATATTAATATTTA----
AGAATTTAATATCTGAGTCAAAAAGCTTAGATTTAC-TCGTAGGACGAGAAGACCCTATAGAA--
TTTTATTA------TATT-AATTTATTGT---TGGTTTTTGGT---TATT----
ATTAGTCTGATAATATTTTGTGTAATTGTGTTGGGGTGACAGGGAAA-TTGTTTTAACTTTCTTTATT--
TATTTA-TTAC-GAATTGTATA-----TTTGAGATCCTATGAA-TT---TGGATA--
GTAAGATTAAATTACCTTAGGGATAAC
Adrisa_magna
ATGCAAGTCTCAGTACGAGCTGACT-
TAAAGTGAAACCGCAAAAGGCTCAGTAAACCGGTCATAGTTTACCGGA-TATATG-CCACTC-
ACGTGGATAACTGTGGTAATTCTAGAGCTAATACATGCAACC-AAGTCCCGACCAGCG---
ATGGAAAGGACGCTTTTATTGGATCAAAACCATTCGCCCGGCTC-GCCCGGGC------
GTCTGCTTGATGACTCT-AGATAAC-ACCACA-GATCGCACGGTCACAGTACCGGC-
GACGTATCCGTCAAATGTCTGACTTATCAGCTGTCGATGGTAGACTCTGTGTCTACCATGGCGCTCACGGGTA
ACGGGGAATCAGGGTTCGATTCCGGAGAGGGAGCCTGAGAAACGGCTACCACATCCAAGGAAGGCAGCAGGCA
CGCAAATTACCCACTCCCGGCACGGGGAGGTAGTGACAAAAAATAACGATACGGGACTCATCCGAGGCCCCGT
AATCGGAATGAGAACACCCTAAATCCTTTAACGAGGATCTATTGGAGGGCAAGTCTGGTGCCAGCAGCCGCGG
TAATTCCAGCTCCAATAGCGTATATTAAAGTTGTTGCGGTTAAAAA-
GCTCGTAGTTGGTTCTGCGTGCCACGCTGTCGGTTCACCGCCTGTCGGTGCAACTGGCATGCC-
GTGGCATGTCCTGTCGGTGGCTGACAGGC-TTCGCG-GCGCGCCCTATG-------CGTTCAC-GCGTGT---
-AGGTCGTTGC--GCCG-CGTTGTCC-TTGTAGG-CCGGCT-CAATCCTACCGCGGTGCT-
CTTTGCCGAGTGTCGAGGTAGGCCGACACT-
GTTTACTTTGAACAAATTAGAGTGCTTAAAGCAGGCTCTGAAAGC-
AGCCTGAATAGTGGTGCATGGAATAATGGAACAGGACCTTGGTTCTATTTTGTT-GGTTTTCTGA-
ATCCAAGGTAATGATCAATAAAGA-CAGGCGGGGGCATTCGTATTGCGACGTTAGA-----------------
-GGTGAAATTCTTGG-
ATCGTCGCAAGACGCACTAGAGCGAAAGCATTTGCCAAGTATGTCTTAATTGATCAAGAACGAAAGTTAGAGG
TTCGAAGGCGATCAGATACCGCCCTAGTTCTAACCATAAACGATGCCAGCCAGCGATCCGCCGATGTTCCTCC
GATGACTCGGCGGGGAGCTT-
TCCCCGGGAAACCAAAGCTTTTGGGTTCCGGGGGAAGTATGGTTGCAAAGCTGAAACTTAAAGGAATTGACGG
AAGGGCACCACCAGGAGTGGAGCCTGCGGCTTAATTTGACTCAACACGGGAAAGCTCACCAGGCCCGGACATT
GGAA-GGATTGACAGAT-CGATAGCTCTTT-CTCGATT-CAGTGGGT-
AGTGGTGCATGGCCGTTCTTAGTTGGTGGACTGATTTGTCTGGTTAATTCCGATAACGAACGAGACTCTGACC
TATTAACTAGGCGCTC-CCGGTATAA-ATA-CCTACCGGCGAAAT-
TTATTCTTCTTAAGGGGACAGGCGGCTCTCAGCCGCACGAGATTGAGCAATAACAGGTCAGTGATGCCCTTAG
ATGTTCTGGGCCGCACGCGCGCTACACTGAAGGAATCAGCGTGTGC--
TCCCCTGTCCGAAAGGACCGGGCAACCCGCTGAACCTCCTTCGTGCTAGGAATTGGGGCTTGCAATTGTTCCC
CATGAACGAGGAATTCCCAGTAAGCGCGAGTCATAAGCTCGCGTTGATTACGTCCCTGCCCTTTGTACACACC
GCCCGTCGCTACTACTGATTGAATGATTTAGTGAGGTCTTCGGACC-GGCGGTA--AC-CGGC-ACC--GGC-
----TCGCACTT---TATCGGT-TTCGCTGGAAA-
GATGACCGAACTTGATCATTTAGAGGAAGTAAAAGTCGTAACAAGG-TTTCCAGTCA--TTGGGTGC---
AAA-CCTAAAGGCGCAATGAAAGT-GTCAGA-----------GGGGAGCCTCCTC-------------
CGGGAGC-----GCTCTCCGCAGGGAGGATGGGCCGCC----GTGCAATTATT--------TGC--
CGGAGCCCCGCACTCCCGGGGCGTCTC--GTTCTC-ATTACG-AG----
TAGAGACGCACCAGGAGCGCACACGCTGGTATCCGAAAGATGGTGAACTATGCCTGGCCAGGACGAAGCCAGG
GGAAACCCTGGTGGAGGTCC--GAAGCG-ATTCTG--ACGTGCAAATCG-
ATCGTCTGAGCTGGGTATAGGGGCGAAAGACCAATCGAACCATCTGG-T-AAAGTTT--
ATATCAAGTCTGACCTGCTCAATGATTTATATTT-
AAATGATTAAATAGCCGCAGTATTTTGACTGTGCAAAGGTAGCATAATCATTTGTCTTTTAATTGTAGGCTTG

TATGAATGGTTTGATGAAATATCAGCTTTCTTTATACTATTATTAAGTTTAGAATTTAATATTTGAGTTAAAA
AGCTTAAATGGTT-TCGGAGGACGAGAAGACCCTATAGAA--
TTTTATTACTAAGATATTTAATGTGATGTATTTTACTTTTGGT---TTTT----
ATTGATTAATTTACATCTTTTGTAATTTTGTTGGGGTGACAGGGAAA-TTTATTTAACTTTCTTTATA--
TATTAT-TTACAGATTTGTATA-----TTTATGATCCTTTAT--TT---TGGATA--
TTAAGATTAAATTACCTTAGGGATAAC
Adrisa_romani
ATGCAAGTCTCAGTACGAGCTGACT-
TAAAGTGAAACCGCAAAAGGCTCAGTAAACCGGTCATAGTTTACCGGA-TATATG-CCACTC-
ACGTGGATAACTGTGGTAATTCTAGAGCTAATACATGCAACC-AAGTCCCGACCAGCG---
ATGGAAAGGACGCTTTTATTGGATCAAAACCATTCGCCCGGCTC-GCCCGGGC------
GTCTGCTTGATGACTCT-AGATAAC-ACCACA-GATCGCACGGTCACAGTACCGGC-
GACGTATCCGTCAAATGTCTGACTTATCAGCTGTCGATGGTAGACTCTGTGTCTACCATGGCCCTCACGGGTA
ACGGGGAATCAGGGTTCGATTCCGGAGAGGGAGCCTGAGAAACGGCTACCACATCCAAGGAAGGCAGCAGGCA
CGCAAATTACCCACTCCCGGCACGGGGAGGTAGTGACAAAAAATAACGATACGGGACTCATCCGAGGCCCCGT
AATCGGAATGAGAACACCCTAAATCCTTTAACGAGGATCTATTGGAGGGCAAGTCTGGTGCCAGCAGCCGCGG
TAATTCCAGCTCCAATAGCGTATATTAAAGTTGTTGCGGTTAAAAA-
GCTCGTAGTTGGTTCTGCGTGCCACGCTGTCGGTTCACCGCCTGTCGGTGCAACTGGCATGCC-
GTGGCATGTCCTGTCGGTGGCTGACAGGC-TTCGCG-GCGCGCCCTATG-------CGTTCAC-GCGTGT---
-AGGTCGTCGC--GCCG-CGTTGTCC-TTGTAGG-CCGGCT-CAATCCTACCGCGGTGCT-
CTTTGCCGAGTGTCGAGGTAGGCCGACACT-
GTTTACTTTGAACAAATTAGAGTGCTTAAAGCAGGCTCTGAAAGC-
AGCCTGAATAGTGGTGCATGGAATAATGGAACAGGACCTTGGTTCTATTTTGTT-GGTTTTCTGA-
ATCCAAGGTAATGATCAATAAGGA-CAGGCGGGGGCATTCGTATTGCGACGTTAGA-----------------
-GGTGAAATTCTTGG-
ATCGTCGCAAGACGCACTAAAGCGAAAGCATTTGCCAAGTGTGTCTTAATTGATCAAGAACGAAAGTTAGAGG
TTCGAAGGCGATCAGATACCGCCCTAGTTCTAACCATAAACGATGCCAGCCAGCGATCCGCCGATGTTCCTCC
GATGACTCGGCGGGGAGCTT-
TCCCCGGGAAACCAAAGCTTTTGGGTTCCGGGGGAAGTATGGTTGCAAAGCTGAAACTTAAAGGAATTGACGG
AAGGGCACCACCAGGAGTGGAGCCTGCGGCTTAATTTGACTCAACACGGGAAACCTCACCAGGCCCGGACATT
GGAA-GGATTGACAGAT-CGATAGCTCTTT-CTCGATT-CAGTGGGT-
AGTGGTGCATGGCCGTTCTTAGTTGGTGGACTGATTTGTCTGGTTAATTCCGATAACGAACGAGACTCTGGCC
TATTAACTAGGCGCTC-CCGGTATAA-ATA-CCTACCGGCGAGAT-
TTATTCTTCTTAAGGGGACAGGCGGCTCTCAGCCGCACGAGATTGAGCAATAACAGGTCTGTGATGCCCTTAG
ATGTTCTGGGCCGCACGCGCGCTACACTGAAGGAATCAGCGTGTGC--
TCCCCTGTCCGAAAGGACCGGGCAACCCGCTGAACCTCCTTCGTGCTAGGAATTGGGGCTTGCAATTGTTCCC
CATGAACGAGGAATTCCCAGTAAGCGCGAGTCATAAGCTCGCGTTGATTACGTCCCTGCCCTTTGTACACACC
GCCCGTCGCTACTACTGATTGAGTGATTTAGTGAGGTCTTCGGACC-GGCGTTA--AC-CGGC-ACC--GGC-
----TCGTCCGG---TATCGGT-TTCGCTGGAAA-
GATGACCGAACTTGATCATTTAGAAGAAGTAAAAGTCGTAACAAGG-TTTCCAGTCA--TTGGGTGC---
AAA-CCTAAAGGCGCAATGAAAGT-GTCAGA-----------GGGGGGCCTCCTC-------------
CGGGAGA-----CCTCTCCGCTGGGAGGATGGGCCGCC----GTGCAATTATT--------TGC--
CGGAGCCCCGCACTCCCGGGGCGTCTC--GTTCTC-ATTACG-AG----
TAGAGACGCACCAGGAGCGCACACGCTGGTATCCGAAAGATGGTGAACTATGCCTGGCCAGGACGAAGCCAGG
GGAAACCCTGGTGGAGGTCC--GAAGCG-ATTCTG--ACGTGCAAATCG-
ATCGTCTGAGCTGGGTATAGGGGCGAAAGACCAATCGAACCATCTCG-TTGAAGTTT--
ATGTCAAGTCGGGCCTGCTCAATGATTATTTGT---
AATAATTTAATAGCCGCAGTATTTTGACTGTGCAAAGGTAGCATAATCATTTGTCTTTTAATTGTAGGCTTGT
ATGAATGGTTTGATGAGATATCATCTTTCTTTATATTATCATG-A----
AGAATTTAATTTTTAAGTTAAAAAGCTTAAATACTT-TCGTAGGACGAGAAGACCCTATAGAA--
TTTTATCA------TATTTAATTCGTTCT---TATTTTTTGGT---TGTT----ATTAAATAAATA-
CGGGGTATATGATTATGTTGGGGTGACAGGGAAA-TTAATTTAACTTTCTTTATT--TTATTA-
TTACTGATTTGTATT-----TTTATGATCCTATTT--TT---TGGATA--
GTAAGATTAAATTACCTTAGGGATAAC
Aethus_pseudindicus
ATGCAAGTCTCAGTACGAGCTGACT-
TAAAGTGAAACCGCAAAAGGCTCAGTAAACCGGTCATAGTTTACCGGA-TATATG-CCACTC-
ACGTGGATAACTGTGGTAATTCTAGAGCTAATACATGCAACC-AAGTCCCGACCAGCG---

ATGGAAAGGACGCTTTTATTGGATCAAAACCATTCGCCCGGCTC-GCCCGGGC------
GTCTGCTTGATGACTCT-AGATAAC-ACCACA-GATCGCACGGTCACAGTACCGGC-
GACGTATCCGTCAAATGTCTGACTTATCAGCTGTCGATGGTAGACTCTGTGTCTACCATGGCGCTCACGGGTA
ACGGGGAATCAGGGTTCGATTCCGGAGAGGGAGCCTGAGAAACGGCTACCACATCCAAGGAAGGCAGCAGGCA
CGCAAATTACCCACTCCCGGCACGGGGAGGTAGTGACAAAAAATAACGATACGGGACTCATCCGAGGCCCCGT
AATCGGAATGAGAACACCCTAAATCCTTTAACGAGGATCTATTGGAGGGCAAGTCTGGTGCCAGCAGCCGCGG
TAATTCCAGCTCCAATAGCGTATATTAAAGTTGTTGCGGTTAAAAA-
GCTCGTAGTTGGTTCTGCGTGCCACGCTGTCGGTTCACCGCCTGTCGGTGCAACTGGCATGCC-
GTGGCATGTCCTGTCGGTGGCTGACAGGC-TTCGCG-GCGCGCCCTATG-------CGTTTAC-GCGTGT---
-AGGTCGTCGC--GCCGACGTTGTCC-TTGTAGG-CCGGCT-CAATCCTACCGCGGTGCT-
CTTTGCCGAGTGTCGAGGTAGGCCGACACT-
GTTTACTTTGAACAAATTAGAGTGCTTCAAGCAGGCTCTGAAAGC-
AGCCTGAATAGTGGTGCATGGAATAATGGAACAGGACCTTGGTTCTATTTTGTT-GGTTTTCGGA-
ATCCAAGGTAATGATCAATAAGGA-CAGGCGGGGGCATTCGTATTGCGACGTTAGA-----------------
-GGTGAAATTCTTGG-
ATCGTCGCAAGACGCACTAGAGCGAGAGCATTTTCCAAGTATGTCTTAATTGATCAAGAACGAAAGTTAGAGG
TTCGAAGGCGATCAGATACCGCCCTAGTTCTAACCATAAACGATGCCAGCCAGCGATCCGCCGATGTTCCTCC
GATGACTCGGCGGGGAGCTT-
TCCCCGGGAAACCAAAGCTTTTGGGTTCCGGGGGAAGTATGGTTGCAAAGCTGAAACTTAAAGGAATTGACGG
AAGGGCACCACCAGGAGTGGAGCCTGCGGCTTAATTTGACTCAACACGGGAAACCTCACCAGGCCCGGACATT
GGAA-GGATTGACAGAT-CGATAGCTCTTT-CTCGATT-CAGTGGGT-
AGTGGTGCATGGCCGTTCTTAGTTGGTGGACTGATTTGTCTGGTTAATTCCGATAACGAACGAGACTCTGACC
TATTAACTAGGCGCTC-CCGGTATAA-ATA-CCTACCGGCGAGAT-
TTATTCTTCTTAAGGGGACAGGCGGCTCTTAGCCGCACGAGATTGAGCAATAACAGGTCTGTGATGCCCTTAG
ATGTTCTGGGCCGCACGCGCGCTACACTGAAGGAATCAGCGTGTGC--
TCCCCTGGCCGAAAGGTCCGGGCAACCCGCTGAACCTCCTTCATGCTAGGAATTGGGGCTTGCAATTGTTCCC
CATGAACGATGAATTCCTAGTAAGCGCGAGTCATAAGCTCGCGTTGATTACATCCCTGCCCTTTGTACACACC
GCCCGTCGCTACTACCGATTGAATGATTTAGTGAGGTCTTCGGACC-GGCGCTA--AC-CGGC-ACC--GGC-
----TCGCTCGG---TATCGGT-TTCTCTGGAAA-
GATGACCGAACTTGATCTTTTAGAGGAAGTAAAAATAAAAAAAAGT-TTTCCAGTCA--TTGGGTGC---
AAA-CCTAAAGGCGCAATGAAAGT-GTCAG-------------GAGGGGCTCCTT-------------
TAGGAGT-----CCTCTCCGCAGGGAGGATGGGCCACC----GTACAATTACT--------TGT--
CGGAGCCCCGCACTCCCGGGGCGTCTC--GTTCTC-ATCACG-AG----
TAGAGACGCACCAGGAGCGCACACGCTGGTACCCGAAAGATGGTGAACTATGCCTGGCCAGGACGAAGCCAGG
GGAAACCCTGGTGGAGGTCC--GAAGCG-ATTCTG--ACGTGCAAATCG-
ATCGTCTGAGCTGGGTATAGGGGCGAAAGACCAATCGAACCATCGGT-GGGGATT----
AAATCATGTCGGGCCTGCTCA-TGATTTT-------AAT-
ATTTAATAGCCGCAGTATCTTGACTGTGCGAAGGTAGCATAATCATTTGTCTTTTAATTGTAGGCTTGTATGA
AGGGTTTGATGAAATTCTAACTTTCTTTATTTTATATTTAA-----
TAATTTAATGTTTGGGTCAAAAAGCTTAGATGATT-CCATAGGACGAGAAGACCCTATAGAG--TTTTATT--
------ATGGGATT-TGTGATTTGTATTATGGTA--AATTT----GTTTC-ATTTTATAGATTCT-
ATAATTGTGTTGGGGCGACAGGGAAA-TTTTTTTAACTTTCTTTGAT-AATTTAT-TTATTAATGAGTAT---
--GTTTATGATCCCATA---TT--AGGGATA--GTAAGATTAAATTACCTTAGGGATAAC
Alonips_obsoletus
ATGCAAGTCTCAGTACGAGCTGACT-
TAAAGTGAAACCGCAAAAGGCTCAGTAAACCGGTCATAGTTTACCGGA-TATATG-CCACTT-
ACGTGGATAACTGTGGTAATTCTAGAGCTAATACATGCAACC-AAGTCCCGACCAGCG---
ATGGAAAGGACGCTTTTATTGGATCAAAACCATTCGCCCGGCTC-GCCCGGGC------
GTCTGCTTGATGACTCT-AGATAAC-ACCACA-GATCGCACGGTCACAGTACCGGC-
GACGTATCCGTCAAATGTCTGACTTATCAGCTGTCGATGGTAGACTCTGTGTCTACCATGGCGCTCACGGGTA
ACGGGGAATCAGGGTTCGATTCCGGAGAGGGAGCCTGAGAAACGGCTACCACATCCAAGGAAGGCAGCAGGCA
CGCAAATTACCCACTCCCGGCACGGGGAGGTAGTGACAAAAAATAACGATACGGGACTCATCCGAGGCCCCGT
AATCGGAATGAGAACACCCTAAATCCTTTAACGAGGATCTATTGGAGGGCAAGTCTGGTGCCAGCAGCCGCGG
TAATTCCAGCTCCAATAGCGTATATTAAAGTTGTTGCGGTTAAAAA-
GCTCGTAGTTGGTTCTGCGTGCCACGCTGTCGGTTCACCGCCTGTCGGTGCAACTGGCATGCC-
GTGGCATGTCCTGTCGGTGGCTGACAGGC-TTCGCG-GCGCGCCCTATG-------CGTTTAC-GCGTGT---
-AGGTCGTCGC--GCCGACGTTGTCC-TTGTAGG-CCGGCT-CAATCCTACCGCGGTGCT-
CTTTGCCGAGTGTCGAGGTAGGCCGACACT-

GTTTACTTTGAACAAATTAGAGTGCTTCAAGCAGGCTCTGAAAGC-
AGCCTGAATAGTGGTGCATGGAATAATGGAACAGGACCTTGGTTCTATTTTGTT-GGTTTTCGGA-
ATCCAAGGTAATGATCAATAAGGA-CAGGCGGGGGCATTCGTATTGCGACGTTAGA-----------------
-GGTGAAATTCTTGG-
ATCGTCGCAAGACGCACTAGAGCGAAAGCATTTGCCAAGTATGTCTTAATTGATCAAGAACGAAAGTTAGAGG
TTCGAAGGCGATCAGATACCGCCCTAGTTCTAACCATAAACGATGCCAGCCAGCGATCCGCCGATGTTCCTCC
GATGACTCGGCGGGGAGCTT-
TCCCCGGGAAACCAAAGCTTTTGGGTTCCGGGGGAAGTATGGTTGCAAAGCTGAAACTTAAAGGAATTGACGG
AAGGGCACCACCAGGAGTGGAGCCTGCGGCTTAATTTGACTCAACACGGGAAACCTCACCAGGCCCGGACATT
GGAA-GGATTGACAGAT-CGATAGCTCTTT-CTCGATT-CAGTGGGT-
AGTGGTGCATGGCCGTTCTTAGTTGGTGGACTGATTTGTCTGGTTAATTCCGATAACGAACGAGACTCTGACC
TATTAACTAGGCGCTC-CCGGTATAA-ATA-CCTACCGGCGAGAT-
TTATTCTTCTTAAGGGGACAGGCGGCTCTTAGCCGCACGAGATTGAGCAATAACAGGTCTGTGATGCCCTTAG
ATGTTCTGGGCCGCACGCGCGCTACACTGAAGGAATCAGCGTGTGC--
TCCCCTGGCCGAAAGGTCCGGGCAACCCGCTGAACCTCCTTCGTGCTAGGAATTGGGGCTTGCAATTGTTCCC
CATGAACGAGGAATTCCCAGTAAGCGCGAGTCATAAGCTCGCGTTGATTACGTCCCTGCCCTTTGTACACACC
GCCCGTCGCTACTACCGATTGAATGATTTAGTGAGGTCTTCGGACC-GGCGCTA--AC-CGGC-ACC--GGC-
----TCGTTCGG---TATCGGT-TTCGCTGGAAA-
GATGACCGAACTTGATCATTTAGAGGAAGTAAAAGTCGTAACAAGG-TTTCCAGTCA--TTGGGTGC---
AAA-CCTAAAGGCGCAATGAAAGT-GTCAG-------------GTGGGACTCCTT-------------
TAGGAGT-----TCCCTCCGCAGGGAGGATGGGTTACC----GTACTATTAT----------GT--
CGGGGCCCCGCACTCCCGGGGCGTCTC--GTTCTC-ATCACG-AG----
TGGAGACGCACCAGGAGCGCACACGCTGGTACCCGAAAGATGGTGAACTATGCCTGGCCAGGACGAAGCCAGG
GGAAACCCTGGTGGAGGTCC--GAAGCG-ATTCTG--ACGTGCAAATCG-
ATCGTCTGAGCTGGGTATAGGGGCGAAAGACCAATCGAACCATCTAG-AGTGATT-T--
ATTTTAGGTCGGGCCTGCTCAGTGATT--------
ATTTGATTAAATAGCCGCAGTATTTTGACTGTGCGAAGGTAGCATAATCATTTGTCTTTTAATTGTAGGCTTG
TATGAAGGGTTTGATGAAATATTGACTTTCTTTGTATTAAATATAT------
AACTTAATGATTTAGTTAAAAGGCTAAGATTTTT-TCAGAGGACGAGAAGACCCTATAGAA--TTTTATT---
-----ATTGCTATAATTTTATTTATTTAG-GTA--CTGTT----
GGTATAATTGAAGTTTTTAGGATAATTTTGTTGGGGCGACAGGGAAA-TTTGATTAACTTTCTTT--A-
TTTTTAT-TTAATAATTGTTATT---GT-TAGAGATCCTTTA---TT--AGGGATT--
ATAAGATTAATTTACCTTAGGGATAAC
Amaurocoris_curtus
ATGCAAGTCTCAGTACAAGCTGAAT-
TAAAGTGAAACCGCAAAAGGCTCAGTAAACCGGTCATAGTTTACCGGA-TATATG-CCACTC-
ACGTGGATAACTGTGGTAATTCTAGAGCTAATACATGCAAAC-AAGTCCCGACCAGCG---
ATGGAAAGGACGCTTTTATTGGATCAAAACCATTCGCCCGGCTC-GCCCGGGC------
GTCTGCTTGATGACTCT-AGATAAC-ACCACA-GATCGCACGGTCACAGTACCGGC-
GACGTATCCGTCAAATGTCTGACTTATCAGCTGTCGATGGTAGACTCTGTGTCTACCATGGCGCTCACGGGTA
ACGGGGAATCAGGGTTCGATTCCGGAGAGGGAGCCTGAGAAACGGCTACCACATCCAAGGAAGGCAGCAGGCA
CGCAAATTACCCACTCCCGGCACGGGGAGGTAGTGACAAAAAATAACGATACGGGACTCATCCGAGGCCCCGT
AATCGGAATGAGAACACCCTAAATCCTTTAACGAGGATCTATTGGAGGGCAAGTCTGGTGCCAGCAGCCGCGG
TAATTCCAGCTCCAATAGCGTATATTAAAGTTGTTGCGGTTAAAAA-
GCTCGTAGTTGGTTCTGCGTGCCACGCTGTCGGTTCACCGCCTGTCGGTGCAACTGGCATGCC-
GTGGCATGTCCTGTCGGTGGCTGACAGGT-TTCGAG-GCGCGCCCTATG-------CGTTTAC-TCGTGT---
-AGGTCGTGGC--GCCT-CGTTGTCC-TTGTTGG-CCGGCT-CAATCCTACCGCGGTGCT-
CTTTACCGAGTGTCGAGGTAGGCCGACACT-
GTTTACTTTGAACAAATTAGAGTGCTTCAAGCAGGCTCTGAAAGC-
AGCCTGAATAGTGGTGCATGGAATAATGGAACAGGACCTTGGTTCTATTTTGTT-GGTTTTCGGA-
ATCCAAGGTAATGATCAATAAGGA-CAGGCGGGGGCATTCGTATTGCGACGTTAGA-----------------
-GGTGAAATTCTTGG-
ATCGTCGCCAGGGTCACTAAAGCGAACATTTTCAGAAGGCGTATCTTGCTTGATCAAGAACGAAAGTTAGAGG
TTCGAAGGCGATCAGATACCGCCCTAGTTCTGACCATAAACGATGCCCGCCAGCGATCCGCCAATGTTCCTCC
GATGACTCGGCGGGGAGCTT-
TCCCCGGGAAACCAAAGCTTTTGGGTTCCGGGGGAAGTATGGTTGCAAAGCTGAAACTTAAAGGAATTGACGG
AAGGGCACCACCAGAAGTGGAGCCGGCGGCTTAATTTGACTCAACACGGGAAACCTCACCAGGCCCGGACCTT
GGAA-GGATTGACAGAAACAATAGCTCTTT-CTCGATT-CGATGGGT-

AGTGGGGCATGGCCGTTCTTAGTTGGTGGACTGATTTGTCTGGTTAATTCCGATAACGAACGAGACTCTGACC
TATTAACTAGGCGCTC-CCGGTATAG-ACA-CCTACCGGCGAGAT-
TTATTCTTCTTAAGGGGACAGGCGGCTCTTAACCCCACAAAATTGAGCAATAACAGGTCTGTGATGCCCTTAG
ATGTTCTGGGCCGCACGCCCGCTACACTGAAAGAATAAACGTGTGC--
TCCCCTGTCCGAAAGGGCCCGGCGGCCCGCTTAACCTCCTTCTTGCTAAGAATTGGGGCTTGCAATTGTTCCC
CCAGAACGAGGAGATCCCAGTAAGCGCGAGTCATAAGCTCGCGTTGATTACCTCCCCGCCCCCTGTACACACC
ACCCGTCGCTTCTACCGATTGAATGATTTAGTGAAGTCGTCGGACCCAGCGGTG--AC-AAGC-ACC--GGC-
----TCGTTCGG---TATCTGT-TTT-CTGGAGA-
GATGACCGAACTTGATCATTTATAAGAAGTAAAAATCGTTACAAGT-GTTCAGTCA---TTGGGTGT---
AAA-CCTAAAGGCGCAATGAAAGT-GTCTG-------------GGGTTCCTCCTT-------------
TTGGAGG-----TTTCCCCGGAGGGAGGATGGGCCACC----CTGCATTTTTAG-------TGC--
CGGAGCCCCGCACTCCCGGGGCGTCTC--GTTCTC-ATTGCG-AG----
TAGAGACGCACCAGGAGCGCACACGCTGGTACCCGAAAGATGGTGAACTATGCCTGGCCAGGACGAAGCCAGG
GGAAACCCTGGTGGAGGTCC--GAAGCG-ATTCTG--ACGTGCAAATCG-
ATCGTCTGAGCTGGGTATAGGGGCGAAAGACCAATCGAACCATCAG---TTATATTT--
GTTTTAAGTCTGTCCTGCTCAATGATTTAT----TT---
AATTAAATGGCCGCAGTATTTTGACTGTGCAAAGGTAGCATAATCATTTGTCTTTTAATTGTAGGCTTGTATG
AATGGTTTGATGAAATACCAGCTTTCTTTATATTATATAGTG------
AATTTAATTTTTGAGTTAAAAAGCTTAAATTTAT-TTATGGGACGAGAAGACCCTATAGAA--TTTT-AT---
------TTATATTTAATTTATTTAATTATATGGTTATTT------TAATT-ATTAGTT-
TAAATATAAATTATGTTGGGGCGACAGGAAAA-TTTAATTAACTTTTCTTATT--TATATT-
TTATATATTTATATA-----TTAAAGATCCTAGAA--TT---AGGATT--
ATAAGATTAAATTACCTTAGGGATAAC
Amnestus_ficus
ATGCAAGTCTCAGTACGAGCTGTCT-
TAAAGCGAAACCGCAAATGGCTCAGTAAACCGGTCATAGTTTACCGGA-TATATG-CCACTT-
ACGTGGATAACTGTGGTAATTCTAGAGCTAATACATGCAACC-AAGTCCCGACCAGCG---
ATGGAAAGGACGCTTTTATTGGATCAAAACCATTCGCCCGGCTC-GCCCGGGC------
GTCTGCTTGATGACTCT-AGATAAC-CTAACA-GATCGCACGGTCACAGTACCGGC-
GACGTATCCGTCAAATGTCTGACTTATCAGCTGTCGATGGTAGACTCTGTGTCTACCATGGCGCTCACGGGTA
ACGGGGAATCAGGGTTCGATTCCGGAGAGGGAGCCTGAGAAACGGCTACCACATCCAAGGAAGGCAGCAGGCA
CGCAAATTACCCACTCCCGGCACGGGGAGGTAGTGACAAAAAAATAACGATACGGGACTCATCCAAGGCCCCGT
AATCGGAATGAGAACACCCTAAATCCTTTAACGAGGATCTATCGGAGGGCAAGTCTGGTGCCAGCAGCCGCGG
TAATTCCAGCTCCGATAGCGTATATTAAAGTTGTTGCGGTTAAAAA-
GCTCGTAATTGGTTCTGCGTGCCACGCTGTCGGTTCACCGCCTGTCGGTGCAACTGGCATGCC-
GTGGCATGTCCTGTCGGTGGCTGACAGGC-TTCGCG-GTCCGCCCTACGCGGTCCCGGGTAAACGGGTGT---
-AGGTCGTTGC--GCCGCGTT-GTCC-TTGTAGG-CCGGCT-CAATCCTACCGCGGTGCT-
CTTTGCCGAGTGTCGAGGTAGGCCGACACT-
GTTTACTTTGAACAAATTAAAGTGCTTAAAGCAGGCTCTGAAAGC-
AGCCTGAATAGTGGTGCATGGAATAATGAAACAGGACCTTGGTTCTATTTTGTT-GGTTTTCTGA-
ATCCAAGGTAATGATCAATAAGGA-ACGGCGGGGGCATTCGTATTCCAACGTTGAA-----------------
-GGTGAAATTCTTGG-
ATTGTCGCAAGAACGACTAAAACCAAAGCATTTGCCAGGTGGGTCTTAATTGATCAAGAACGAAAATTAGAGG
CTAGAAGGAGATCAGATACCGCCCTAGTTATAACCATAAACGATGCCAGCCAGCGATCCGCCGATGTTCCTCC
GATGACTTGGGGGGGAGCTC-
TACCCGGGAAACCAAAGCTTTTGGGTTCCGGGGGAAGTATGGTTGCAAAGCTGAAACTTAAAGGAATTGAAGG
AAGGGCACAACCCGGAATGGAGCCTGCGGGTTAATTTGACTCAACACGGGAAAGCTCACCAGGCCCGGACATT
GGGA-GGATTGACAGAT-TGATAGCTGTTT-GTTGATT-CAGTGGGT-
AGTGGTGCATGGCCGTTTTTAGTTGGTGGATTGATTTGTGTGGGTAATTCCGTTAACGAAAGAGACTATGGCC
TATTAAATAGGCGCTT-CCGGTATGG-ATA-CCTACCGGCGAGAT-
TTATTTTTCTTAAGGGGACCGGCGGCTTCAAGCCGCACGAGATTGAGCAACGACAGGTCAGTGATGCCCTTAG
ATGTTTTGGGCCGCACGCGCGCTACACTGAAGGAATCAGAGTGTGC--
TCCCCTGTCCGAAAGGACCGGGTAACCCGCTGAAACTCCTTAGTGCTAGGAATCGGGGCTTGCAATTTTTCCC
CGTGAATGAGGAATGCCCAGTAAGTGCGAATCATAAGCTCGCGCTGATTACGTCCCTGCCCCTTGTCCACACC
GCCCGTCGCTACTACCGATTGAATGATTTAGTGAGGTATTCGGACC-GGCGGCG--ACGCGGC-ATA--GGC-
----TCGTCCCG---TGCTGGT-
ACCGCTGAAAAAGATGACCGAACTTGATCATTAGGAGGAAGTACAGTGTACAGCCAAG-GTTTCAGTCT--
CTGGGTTT---GAAACCTTTAGGCGCAATGAAAGT-GTCT--------------AATGTGTACGCC-------

------AGGGGACG---GGCGTAAAACCGG-AGGGTGGACTGAA----GCTTAAACT-------------
GGGGGGGCGCCTCACTCCCGGGGCATTTCGAGACCTC-CTCGTGAGG---
GGAGAGACGCACCAGGAGCGCGCGCGCTGGTACCCGAAAGATGGTGAACTATGCCTGATTAGGAGGAAGCCAG
GGGAAACCCTGGTGGAGGTCTCGGAAGCGGATTCTGGAACGTGCAAATCGGATCGTACGTAATTGGTATAGAG
GCGAAAGACTAATCAAACCATCTAG-------TCTTTATTATAGGTTTGACCTGCTCAATGATTATA------
---
AATTAAATAGCTGCAGTATTTTGACTGTACAAAGGTAGCATAATCATTTGTCTTTTAATTGAAGGCTGGTATG
AAGGGTCGGACGAGATACCTGCTTTCTTAA---TAATAATTT---
ATGAATTTAAAATTTAGGTTAAAATGCTTAAATGTTT-TTAAGGGACGAGAAGACCCTATAGAA--
TTTAAATATAAT------------TTTATATTTTATTTCTGGTTA-TT-AT---TGTAT-TTAATT-
TTAAAATAATATTTTTATTGGGGTGATAGGAAAA-TTTGTTTAACTTTTTTT--ATTTATTTT-
TCATGAATTAATGTA----TTTAAAGATCCCTTAATAA-----GGATT--
ATAAGATTAAATTACCTTAGGGATAAC
Amnestus_subferrugineus
ATGCAAGTCTCAGTACGAGCTACCT-
TAAAGCGAAACCGCAAAAGGCTCAGTAAACCGGTCATAGTTTACCGGA-GATATG-CCACTC-
ACGTGGATAACTGTGGTAATTCTAGAGCTAATACATGCAAAC-AAGTCCCGACCAGCG---
ATGGAAAGGACGCTTTTATTGGATCAAAACCATTCGCCCGGCTC-GCCCGGGC------
GTCAGCTTGATGACTCT-AGATAAC-CTAACA-GATCGCACGGTCACAGTACCGGC-
GACGTATCTGTCAAATGTCTGACTTATCAGCTGTCGATGGTAGACTCTGTGTCTACCATGGCGCTCACGGGTA
ACGGGGAATCAGGGTTCGATTCCGGAGAGGGAGCCTGAGAAACGGCTACCACATCCAAGGAAGGCAGCAGGCA
CGCAAATTACCCACTCCCGGCACGGGGAGGTAGTGACAAGAAATAACGATACGGGACTCATCCGAGGCCCCGT
AATCGGAATGAGAACACCATAAATCCTTTAACGAGGATCTATCGGAGGGCAAGTCTGGTGCCAGCAGCCGCGG
TAATTCCAGCTCCGATAGCGTATATTAAAGTTGTTGCGGTTAAAAA-
GCTCGTAGTTGGTTCTGCGTGCCACGCTGTCGGTTCACCGCCTGTCGGTGCAACTGGCATGCC-
GTGGCATGTCCTGTCGGTGGCCGACAGGT-TTCGCG-GAGCACCCTATGCGCTCCCGGGTAACGCGGTGT---
-AGGCTGTTGC--GCCCGCTGTTCCC-TTGTAGG-CCGGCT-CAATCCTACCGCGGTGCT-
CTTTACCGAGTGTCGAGGTAGGCCGACACT-
GTTTACTTTGAACAAATTAGAGTGCTTCAAGCAGGCTCTGAAAGC-
AGCCTGAATAGTGGTGCATGGAATAATGGAACAGGACCTTGGTTCTATTTTGTT-GGTTTGCGGA-
ATCCAAGGTAATGATCAATAAGGA-CAGGCGGGGGCATTCGTATTGCGACGTTAGA-----------------
-GGTGAAATTCTTGG-
ATCGTCGCAAGACGCACTAGAGCGAAAGCATTTGCCAAGTGTGTCTTAATTGATCAAGAACGAAAGTTAGAGG
TTCGAAGGCGATCAGATACCGCCCTAGTTCTAACCATAAACGATGCCAGCCAGCGATCCGCCGATGCTCCTCC
GATGACTCGGCGGGGAGCTT-
TCCCCGGGAAACCAAAGCTTTTGGGTTCCGGGGGAAGTATGGTTGCAAAGCTGAAACTTAAAGGAATTGACGG
AAGGGCACCACCAGGAGTGGAGCCTGCGGCTTAATTTGACTCAACACGGGAAAGCTCACCAGGCCCGGACATT
GGAA-GGATTGACAGAT-CGATAGCTCTTT-CTCGATT-CAGTGGGT-
AGTGGTGCATGGCCGTTCTTATTTGGTGGACTGATTTGTCTGGTTAATTCCGATAACGAACGAGACTCTGACC
TATTAACTAGGCGCTC-CCGGTATAA-ATA-CCTACCGGCGAGAA-
TTATTCTTCTTAAGGGGACAGGCGGCTCTAAGCCGCACGAGATTGAGCAATAACAGGTCAGTGATGCCCTTAG
ATGTTCTGGGCCGCACGCGCGCTACACTGAAGGAATCAGCGTGTGC--
TCCCCTGTCCGAAAGGTCCGGGCAACCCGCTGAACCTCCTTCGTGCTAGGAATTGGGGCTTGCAATTGTTCCC
CATGAACGAGGAATGCCCAGTAAGCGCGAGTCATAAGCTCGCGCTGATTACGTCCCTGCCCTTTGTCCACACC
GCCCGTCGCTACTACCGATTGAATGATTTAGTGAGGTCTTCGGACC-GGCGTTA--ACGAGGC-ACC--GGC-
----TCGTTCGG---TATCGGT-TTCTCTGGAAA-
GATGACCGAACTTGATCATTTAGAGGAAGTAAAAGTCGTAACAAGG-TTTCCAATCT--CTGGGTGT---
GAAACCTAAAGGCGCAATGAAAGT-GTCTGG------------AGGGTCTGCCTT-------------
TGAGGAGC---GCTACTCCGTAGGGAGGATGGGCAGCC----GTACTC-----------
TTACTGTCGGAGCCCCGCACTCCCGGGGCGTCTC--GGTCTC-TTTACGAGC-----
GGAGACGCACCAGGAGCGCACACGCTGGTACCCGAAAGATGGTGAACTATGCCTGGCCAGGACGAAGCCAGGG
GAAACCCTGGTGGAGGTCC--GAAGCG-ATTCTG--ACGTGCAAATCG-
ATCGTCTGAGCTGGGTATAGGGGCGAAAGACCAATCGAACCATCTA--------------
TATTCGGTTCGCCCTGCCCAATGATCAGTTAT----A-
GGTTGAATGGCTGCAGTATCTTGACTGTACAAAGGTAGCATAATCATTTGTCTTTTAATTGAAGGCTTGTATG
AATGGGTTGATGAGGTGATAACTTTCTTTA---TGCTATTGT-G-
TTGAACTTGATTTTTAAGTTAAAAGGCTTAAATTTAT-TTGGGGGACGAGAAGACCATGTAGAA--TTTA-
ACTTACC------------TAAATAGTTCCATG----TCTCTCCCA---AATG--

TGAATCTATGTTAGTTAAGTTGGGTTGGGGCGACAAAGGGT-CTTTTTAAACACCCTTTAATTATTTTAA-
TTACTTGTTTGTATGA---TTAATTGATCCCGTTATTAT----GGATA--
AAAAGATTAAATTACCTCAGGGATAAC
Amnestus_zacki
ATGCAAGTCTCAGTACGAGCTACCT-
TAATGCGAAACCGCAAAAGGCTCAGTAAACCGGTCATTGTTTACAGGG-TATATG-CCACTC-
ACGTGGATAACTGTGGTAATTCTAGAGCTAATACATGCATAC-AAGTCCCGGCCAGTG---
ATGGCGAGGACGCTTTTATTGGAACAAAACCATTCGCCCGGCCC-GCCCGGGC------
GTCTCTTTGGTGACTCT-AGATAAC-CTAACA-GATCGCACGGTCCAAGTACCGGC-
GACGTATCTTTCAAATGTCTGACTTATCAGCTATCGATGGTAGACTCTGTGTCTACCATGGCTCTCACGGGTA
ACGGGGAATCAGGGTTCGATTCCGGAGAGGGAGCCTGAGAGACGGCTACCACATCCAAGGAAGGCAGCAGGCA
CGCAAATTACCCACTCCCGGCACGGGGAGGTAGTGACAAAAAATAACGATACGGGACTCATCCGAGGCCCCGT
AATCGGAATGAGAACACTCTAAATCCTTTAACGAGGATCTATCGGAGGGCAAGTCTGGTGCCAGCAGCCGCGG
TAATTCCAGCTCCGATAGCGTATATTAAAGTTGTTGCGGTTAAAAA-
GCTCGTAGTTGGTTCTGCGTGCCACGCTGTCGGTTCACCGCCTGCCGGTGCAACTGGCATGCC-
GTGGCATGTCCTGTCGGTGGCCGAAGGGG-TTCCGG-TCGCTCGCTACGGCGGCCCGGGTCAACCGGAGC---
-CG-TCGCCGGA-GCCCGGTCCGCCC-TTGTAGG-CCGTCT-CAATCCTACCTAGGTGCT-
CTTTGCCGAGTGTCGAGGTGGGCCGACACT-
GTTTACTTTGAACAAATTAGAGTGCTTAAAGCAGGCTCTTGAAGC-
CGCCTGAATAGTGGTGCATGGAATAATGGAACAGGACCTTGGTTCTATTTTGTT-GGTTTTCGGA-
ATCCAAGGTAATGATCAATAAGGA-CAGGCGGGGGCATTCGTATTGCGACGTTAGA-----------------
-GGTGAAATTCTTGG-
ATCGTCGCAAGACGCACTAGAGCGGCAGCATTTGCCAAGTGTGTCTTAATTGATCAAGAACGAAAGTTAGAGG
TTCGAAGGCGATCAGATACCGCCCTAGTTCTAACCATAAACGATGCCAGCCAGCGATCCGCCGACGTTCCTCC
GATGACTCGGCGGGGAGCTT-
TCCCCGGGAAACCAAAGCTTTTGGGTTCCGGGGGAAGTATGGTTGCAAAGCTGAAACTTAAAGGAATTGACGG
AAGGGCACCACCAGGCGTGGAGCCTGCGGCTTAATTTGACTCAACACGGGAAAGCTCACCAGGCCCGGACATT
GGGA-GGATTGACAGAT-CGATAGCTCTTT-CTCGATT-CAGTGGGT-
AGTGGTGCATGGCCGTTTTTAGTTGGTGGACTGATTTGTCTGGTTAATTCCGTTAACGAACGAGACTCTGGCC
TATTAAATAGGCGCTT-CCGGTATGG-ATA-CCTACCGGCGAGAT-
TTATTCTTCTTAAGGGGACCGGCGGCTTTAAGCCGCACGAGATTGAGCAACAACAGGTCAGTGATGCCCTTAG
ATGTTCTGGGCCGCACGCGCGCTACACTGAAGGAATCAGCGTGTGC--
TCCCCTGTCCGAAAGGACCGGGTAACCCGCTGAAACTCCTTCGTGCTAGGAATCGGGGCTTGCAATTCTTCCC
CGTGAACGAGGAATGCCCAGTAAGCGCGAGTCATAAGCTCGCGCTGATTACGTCCCTGCCCTTTGTCCACACC
GCCCGTCGCTACTACCGATTGAATGGTTTAGTGAGGTCTTCGGACC-GGCGGCA--ACGCGGC-ATC--GGC-
----TCGTCCGG---TGTCGGT-TCCGCTGTAAA-
GATGACCGAACTTGATCATTTAGAGGAAGTAAAAGTCGTAATGAGG-TTTCCAGTCT--CTGGGTGT---
GAAACCTATGGGCGCAATGAAAGT-GTCAGTC----------CGGTCGGCCCCTC-------------
CGGGGTCC---GGCCGGCGGAGGG-AGGATGGGCCTCC----GCCTGAACC-------------
GGCGTGGCCCCGCACTCCCGGGGCGTTTCGAGCCCTC-CTCGCGAGG---
GCTGAGACGCACCAGGAGCGCGCGCGCTGGTACCCGAAAGATGGTGAACTATGCCTGGTTAGGACGAAGCCAG
GGGAAACCCTGGTGGAGGTCC--GAAGCG-ATTCTG--ACGTGCAAATCG-
ATCGTACAAATTGGGTATAGGGGCGAAAGACCAATCGAACTATCTAG-------
AATTTATTATAGGTTTAACCTGCTCAATGATTAAT---------
AATTAAATAGCTGCAGTATTTTGACTGTACAAAGGTAGCATAATCATTTGTCTTTTAATTGAAGGCTGGTATG
AAGGGTTGGACGAAATACCTACTTTCTTAA---TAATAATTT---
AAGAATTTAAAATTTAGGTTAAAATGCTTAAATTTTT-TTAAGGGACGAGAAGACCCTATAGAA--
TTTTAATTTTAT------------TTTATGATTTGTTT----TTAGTT-TT---TATATTTTATTT-
TTAAAATAATATTTTTGTTGGGGTGATAGGAAAA-TTTTTATAACTTTTTTT--ATTAATTTA-
TCATTAATTAATGTG----TTTAATGATCCTTTATTAA-----GGATT--
ATAAGATTAAATTACCTTAGGGATAAC
Blaena_setosa
ATGCAAGTCTCAGTACAGATTACTT-
TAATGTGAAACCGCAAACGGCTCAGTAAACCGGTCATTGTTTACAGGG-TATATG-CCACTC-
ACGTGGATAACTGTGGTAATTCTAGAGTTAATACATGCATCC-AAGTCCGGACCAGAG---
ATGGCGAGGACGCTTTTATTGGAACAAAACCATTCGCCCGGCCC-GCCCGGGC------
GTCTGCTTGATGACTCT-AGATAAC-ACCACA-GATCGCACGGTCACAGTACCGGC-
GACGTATCTTTCAAATGTCTGACTTATCAGCTGTCGATGGTAGACTCTGTGTCTACCATGGCTCTCACGGGTA

ATGGGGAATCAGGGTTCTATTCCGGAGAGGGAGCCTGAGACACGGCTACCACATCCAAGGAAGGCAGCAGGCA
CGCAAATTACCCACTCCCGGCACGGGGAGGTAGTGACAAAAAATAACGGTACGGGACTCATCCGAGGCCCCGT
AATCGGAATGAGAACACCCTAAATCCTTTAACGAGGATCTATTGGAGGGCAAGTCTGGTGCCAGCAGCCGCGG
TAATTCCAGCTCCAATAGCGTATATTAAAGTTGTTGCGGTTAAAAA-
GCTCGTAGTTGGTTCTGCGTGCCACGCTGTCGGTTCACCGCCTGTCGGTGCAACTGGCATGCC-
GTGGCATGTCCTGTCGGTGGCTGACAGGC-TTCGCG-GCGCGCCCTATG-------CGTTTAC-GCGTGT---
-AGGTCGTCGC--GCCGACGTTGTCC-TTGTAGG-CCGGCT-CAATCCTACCGCGGTGCT-
CTTTGCCGAGTGTCGAGGTAGGCCGACACT-
GTTTACTTTGAACAAATTAGAGTGCTTCAAGCAGGCTCTGAAAGC-
AGCCTGAATAGTGGTGCATGGAATAATGGAACAGGACCTTGGTTCTATTTTGTT-GGTTTTCGGA-
ATCCAAGGTAATGATCAATAAGGA-CAGGCGGGGGCATTCGTATTGCGACGTTAGA-----------------
-GGTGAAATTCTTGG-
ATCGTCGCAAGACGCACTAGAGCGAAAGCATTTGCCAAGTATGTCTTAATTGATCAAGAACGAAAGTTAGAGG
TTCGAAGGCGATCAGATACCGCCCTAGTTCTAACCATAAACGATGCCAGCCAGCGATCCGCCGATGTTCCTCC
GATGACTCGGCGGGGAGCTT-
TCCCCGGGAAACCAAAGCTTTTGGGTTCCGGGGGAAGTATGGTTGCAAAGCTGAAACTTAAAGGAATTGACGG
AAGGGCACCACCAGGAGTGGAGCCTGCGGCTTAATTTGACTCAACACGGGAAACCTCACCAGGCCCGGACATT
GGAAGG-ATTGACAGAT-CGATAGCTCTTT-CTCGATT-CAGTGGGT-
AGTGGTGCATGGCCGTTCTTAGTTGGTGGACTGATTTGTCTGGTTAATTTCGTTGACGAACGAGAATCTAACC
TATTAACTAGGCGCTC-CCGGTATAT-ATA-CCTACCGGCGAGAT-
TTATTCTTCTTAAGGGGACAGGCGGGTTTTATCCGCTCGAGGTTGAGCAGTAACAGGTCAGTACTGCCATTAG
ATGTGTTGGGTCGCACGCACCCTACACTGAAGGTATTAGCGTGTGC--
TCCCCTGTCCGAAAGTACCGGGTCACCCGGTGAACCTCCTTCGTGCTAGGAATTGCCGCTTGCAATGCTACCC
CATGAACGAGGAATTCCCAGTAAGAATAAATCATAAGCTCGCGCTGATTACGTCCCTGCCCTTTGTCCACACC
GCCCGTCAGTACTACCGATTGAATGATTTAGTGAGGTGATCGGACC-GGCGAGCGTATCCTCC-ACC--
GAAG---ATCAAACGTC--CTTCCAT-TTCGAAAAAAT-TAAAAGACAG-
TTAATACTTTAGAGGAAGTAAAAGTCGTACCATTG-CCCCAAGTCA--TTGGGTGT--
AAAAACCTAAAGGCGCAATGAAAGT-GTCTGC------------GGTCCTCTCCCCCC-----------
CGGGGGAG---GGGCCGCGTCGGGGAGGATGGGCTCCC----GTCCCTCCCGG---------GT--
CGGGGCCCCGCACTCCCGGGGCGCCTC--GCCGCTCGTCACG-AG----
CCGAGGCGCACCAGGAGCGCACACGCTGGTACCCGAAAGATGGTGAACTATGCCTGGCCAGGACGAAGCCAGG
GGAAACCCGGGTGGAGGTCC--GAAGCG-ATTCTG--ACGTGCAAATCG-
ATCGTCTGACCTGGGTATAGGGGCGAAAGACCAATCCAACCAGCTTG----CAATTT--
ATATCACGTTTGGCCTGCTCAATGAATA--------ATT-
TTTAAATAGCCGCAGTACTTTGACTGTGCGAAGGTAGCATAATCATTTGTCTTTTAATTGAAGGCTTGTATGA
ATGGTTGGATGAAATATATGCTTTCTTTATTTTATATATTA-----
GAATTTAATTTTTGAGTTAAAAAGCTTAAATTTGTT-TAGAGGACGAGAAGACCCTATAGAG--
TTTTATATATTTT-TT----------ATTAAGT-ACTATTTGTTATTAGTA---------
CATTATAATTTATT--ATATTTTGTTGGGGCAATGGGGAAA-TTTTAATAATTTTTCTTATTTA-TTTT--
TTATTTATTTGTA-AT----TTAAGGATCCTT-----AATTTTGGATT--
ATAAGATTAAATTACCTTAGGGATAAC
Byrsinus_pseudosyriacus
ATGCAAGTCTCAGTACGAGCTGACT-
TAAAGTGAAACCGCAAAAGGCTCAGTAAACCGGTCATAGTTTACCGGA-TATATG-CCACTC-
ACGTGGATAACTGTGGTAATTCTAGAGCTAATACATGCAACC-AAGTCCCGACCAGCG---
ATGGAAAGGACGCTTTTATTGGATCAAAACCATTCGCCCGGCTC-GCCCGGGC------
GTCTGCTTGATGACTCT-AGATAAC-ACCACA-GATCGCACGGTCACAGTACCGGC-
GACGTATCCGTCAAATGTCTGACTTATCAGCTGTCGATGGTAGACTCTGTGTCTACCATGGCGCTCACGGGTA
ACGGGGAATCAGGGTTCGATTCCGGAGAGGGAGCCTGAGAAACGGCTACCACATCCAAGGAAGGCAGCAGGCA
CGCAAATTACCCACTCCCGGCACGGGGAGGTAGTGACAAAAAATAACGATACGGGACTCATCCGAGGCCCCGT
AATCGGAATGAGAACACCCTAAATCCTTTAACGAGGATCTATTGGAGGGCAAGTCTGGTGCCAGCAGCCGCGG
TAATTCCAGCTCCAATAGCGTATATTAAAGTTGTTGCGGTTAAAAA-
GCTCGTAGTTGGTTCTGCGTGCCACGCTGTCGGTTCACCGCCTGTCGGTGCAACTGGCATGCC-
GTGGCATGTCCTGTCGGTGGCTGACAGGC-TTCGCG-GCGCGCCCTATG-------CGTTTAC-GCGTGT---
-AGGTTGTCGC--GCCGACGTTGTCC-TTGTAGG-CCGGCT-CAATCCTACCGCGGTGCT-
CTTTGCCGAGTGTCGAGGTAGGCCGACACT-
GTTTACTTTGAACAAATTAGAGTGCTTCAAGCAGGCTCTGAAAAT-
AGCCTGAATAGTGGTGCATGGAATAATGGAACAGGACCTTGGTTCTATTTTGTT-GGTTTCCGGA-

ATCCAAGGTAATGATCAATAAGGA-CAGGCGGGGGCATTCGTATTGCGACGTTAGA-----------------
-GGTGAAATTCTTGG-
ATCGTCGCAAGACGCACTAGAGCGAGAGCATTTGCCAAGTATGTCTTAATTGATCAAGAACGAAAGTTAGAGG
TTCGAAGGCGATCAGATACCGCCCTAGTTCTAACCATAAACGATGCCCGCCAGCGATCCGCCGATGTTCCTCC
GATGACTCGGCGGGGAGCTT-
TCCCCGGGAAACCAAAGCTTTTGGGTTCCGGGGGAAGTATGGTTGCAAAGCTGAAACTTAAAGGAATTGACGG
AAGGGCACCACCAGGAGTGGAGCCTGCGGCTTAATTTGACTCAACACGGGAAACCTCACCAGGCCCGGACATT
GGAA-GGATTGACAGAT-CGATAGCTCTTT-CTCGATT-CAGTGGGT-
AGTGGTGCATGGCCGTTCTTAGTTGGTGGACTGATTTGTCTGGTTAATTCCGATAACGAACGAGACTCTGACC
TATTAACTAGGCGCTC-CCGGTATAA-ATA-CCTACCGGCGAGAT-
TTATTCTTCTTAAGGGGACAGGCGGCTCTTAGCCGCACGAGATTGAGCAATAACAGGTCTGTGATGCCCTTAG
ATGTTCTGGGCCGCACGCGCGCTACACTGAAGGAATCAGCGTGTGC--
TCCCCTGGCCGAAAGGTCCGGGCAACCCGCTGAACCTCCTTCGTGCTAGGAATTGGGGCTTGCAATTGTTCCC
CATGAACGAGGAATTCCCAGTAAGCGCGAGTCATAAGCTCGCGTTGATTACGTCCCTGCCCTTTGTACACACC
GCCCGTCGCTACTACCGATTGAATGATTTAGTGAGGTCTTCGGACC-GGCGGTA--AC-CGGC-ACC--GGC-
----TCGTTCGG---TATCGGT-TTCGCTGGAAA-
GATGACCGAACTTGATCATTTGGAGGAAGTAAAAGTCGTAACAAGG-TTTCCAGTCA--TTGGGTGC---
AAA-CCTAAAGGCGCAATGAAAGT-GTCAG-------------GCGGGGCTCCTT-------------
TAGGAGT-----CCCCGCCGCAGGGAGGATGGGCCGCC----GTACAATTATT--------TGT--
CGGAGCCCCGCACTCCCGGGGCGTCTC--GTTCTC-ATCACG-AG----
TGGAGACGCACCAGGAGCGCACACGCTGGTACCCGAAAGATGGTGAACTATGCCTGGCCAGGACGAAGCCAGG
GGAAACCCTGGTGGAGGTCC--GAAGCG-ATTCTG--ACGTGCAAATCG-
ATCGTCTGAGCTGGGTATAGGGGCGAAAGACCAATCGAACCATCT-G-TGAAATT----
TTATTAAGTCAGGCCTGCTCAATGATTA-------
TATTGATTAAATAGCCGCAGTATTTTGACTGTGCGAAGGTAGCATAATCATTTGTCTTTTAATTGTAGGCTAG
AATGAATGGTTTGATGAAATCTTGACTTTCTTTATTTTAATATTAA-----
TAATTTAATGTTTGAGTCAAAAGGCTTAAATGATT-TTGGAGGACGAGAAGACCCTATAGAA--TTTTATT--
------GTAATTTA-GTAGTG--TTATTTTAGGG--AAATG----GTTAATGTCATTTATAATTT-
ACAATTGTGTTGGGGTGACAGGGAAA-TTAGTTTAACTTTCTCTATA-AGTAT-T-
AATATGTTAATTGGCATATATTT-TGATCCTTTA---TT--AAAGATT--
ATAAGATTAAATTACCTTAGGGATAAC
Byrsinus_varians
ATGCAAGTCTCAGTACGAGCTGACT-
TAAAGTGAAACCGCAAAAGGCTCAGTAAACCGGTCATAGTTTACCGGA-TATATG-CCACTC-
ACGTGGATAACTGTGGTAATTCTAGAGCTAATACATGCAACC-AAGTCCCGACCAGCG---
ATGGAAAGGACGCTTTTATTGGATCAAAACCATTCGCCCGGCTC-GCCCGGGC------
GTCTGCTTGATGACTCT-AGATAAC-ACCACA-GATCGCACGGTCACAGTACCGGC-
GACGTATCCGTCAAATGTCTGACTTATCAGCTGTCGATGGTAGACTCTGTGTCTACCATGGCGCTCACGGGTA
ACGGGGAATCAGGGTTCGATTCCGGAGAGGGAGCCTGAGAAACGGCTACCACATCCAAGGAAGGCAGCAGGCA
CGCAAATTACCCACTCCCGGCACGGGGAGGTAGTGACAAAAAATAACGATACGGGACTCATCCGAGGCCCCGT
AATCGGAATGAGAACACCCTAAATCCTTTAACGAGGATCTATTGGAGGGCAAGTCTGGTGCCAGCAGCCGCGG
TAATTCCAGCTCCAATAGCGTATATTAAAGTTGTTGCGGTTAAAAA-
GCTCGTAGTTGGTTCTGCGTGCCACGCTGTCGGTTCACCGCCTGTCGGTGCAACTGGCATGCC-
GTGGCATGTCCTGTCGGTGGCTGACAGGC-TTCGCG-GCGCGCCCTATG-------CGTTTAC-GCGTGT---
-AGGTTGTCGC--GCCGACGTTGTCC-TTGTAGG-CCGGCT-CAATCCTACCGCGGTGCT-
CTTTGCCGAGTGTCGAGGTAGGCCGACACT-
GTTTACTTTGAACAAATTAGAGTGCTTCAAGCAGGCTCTGAAAAT-
AGCCTGAATAGTGGTGCATGGAATAATGGAACAGGACCTTGGTTCTATTTTGTT-GGTTTCCGGA-
ATCCAAGGTAATGATCAATAACGA-CAGGCGGGGGCATTCGTATTGCGACGTTAGA-----------------
-GGTGAAATTCTTGG-
ATCGTCGCAAGACGCACTAGAGCGAAAGCATTTGCCAAGTATGTCTTAATTGATCAAGAACGAAAGTTAGAGG
TTCGAAGGCGATCAGATACCGCCCTAGTTCTAACCATAAACGATGCCAGCCAGCGATCCGCCGATGTTCCTCC
GATGACTCGGCGGGGAGCTT-
TCCCCGGGAAACCAAAGCTTTTGGGTTCCGGGGGAAGTATGGTTGCAAAGCTGAAACTTAAAGGAATTGACGG
AAGGGCACCACCAGGAGTGGAGCCTGCGGCTTAATTTGACTCAACACGGGAAACCTCACCAGGCCCGGACATT
GGAA-GGATTGACAGAT-CGATAGCTCTTT-CTCGATT-CAGTGGGT-
AGTGGTGCATGGCCGTTCTTAGTTGGTGGACTGATTTGTCTGGTTAATTCCGATAACGAACGAGACTCTGACC
TATTAACTAGGCGCTC-CCGGTATAA-ATA-CCTACCGGCGAGAT-

TTATTCTTCTTAAGGGGACAGGCGGCTCTTAGCCGCACGAGATTGAGCAATAACAGGTCTGTGATGCCCTTAG
ATGTTCTGGGCCGCACGCGCGCTACACTGAAGGAATCAGCGTGTGC--
TCCCCTGGCCGAAAGGTCCGGGCAACCCGCTGAACCTCCTTCGTGCTAGGAATTGGGGCTTGCAATTGTTCCC
CATGAACGAGGAATTCCCAGTAAGCGCGAGTCATAAGCTCGCGTTGATTACGTCCCTGCCCTTTGTACACACC
GCCCGTCGCTACTACCGATTGAATGATTTAGTGAGGTCTTCGGACC-GGCGGTA--AC-CGGC-ACC--GGC-
----TCGTTCGG---TATCGGT-TTCGCTGGAAA-
GATGACCGAACTTGATCATTTAGAGGAAGTAAAAGTCGTAACAAGG-TTTCCAGTCA--TTGGGTGC---
AAA-CCTAAAGGCGCAATGAAAGT-GTCAG-------------GTGAGGCTCCTT-------------
TAGGAGT-----CCCCACCGCAGGGAGGATGGGCCGCC----GTACAATTATT--------TGT--
CGGATCCCCGCACTCCCGGGGCGTCTC--GTTCTC-ATCACG-AG----
TGGAGACGCACCAGGAGCGCACACGCTGGTACCCGAAAGATGGTGAACTATGCCTGGCCAGGACGAAGCCAGG
GGAAACCCTGGTGGAGGTCC--GAAGCG-ATTCTG--ACGTGCAAATCG-
ATCGTCTGAGCTGGGTATAGGGGCGAAAGACCAATCGAACCATCTTG-TAGAATT----
TTATTAAGTCGGGCCTGCTCAATGATT--------
TATTTATTAAATAGCCGCAGTATTTTGACTGTGCGAAGGTAGCATAATCATTTGTCTTTTAATTGAAGGCTAG
TATGAATGGTTTGATGAAATTTAGACTTTCTTTATTTTAATATTAA-----
TAATTTGATGTTTAAGTTAAAAAGCTAAGATTATT-TCAGAGGACGAGAAGACCCTATAGAA--TTTTATT--
------ATTATTTG-ATAAAGATTTGTTTTTGGG--AGTTA----ATTTA-ATTTTTTATTGATA-
ATAATTTTGTTGGGGTGACGGGGAAA-TTGTTTTAACTTTCTTTATA-AATGTAT-
AATATGTTAATTAGCATATATAAATGATCCTTTA---TT--ATAGATT--
ATAAGATTAAATTACCTTAGGGATAAC
Canopus_sp.
ATGCAAGTCTCAGTACGAGCTGACT-
TAAAGTGAAACCGCAAAAAGCTCAGTAAACCGGTCATAGTTTACCGGA-TATATG-CCACTC-
ACGTGGATAACTGTGGTAATTCTAGAGCTAATACATGCAACA-AAGTCCCGACCAGTG---
ATGGAAAGGACGCTTTTATTGGATCAAAACCATTCGCCCGTCTCGCAC-GGGC------
GTTTGCTTGATGACTCT-AGATAAC-ACCACA-GATCGCACGGTCACAGTACCGGC-
GACGTATCTGTCAAATGTCTGACTTATCAGCTGTCGATGGTAGACTCTGTGTCTACCATGGCGCTCACGGGTA
ACGGGGAATCAGGGTTCGATTCCGGAGAGGGAGCCTGAGAAACGGCTACCACATCCAAGGAAGGCAGCAGGCA
CGCAAATTACCCACTCCCGGCACGGGGAGGTAGTGACAAAAAATAACGATACGGGACTCATCCGAGGCCCCGT
AATCGGAATGAGAACACCCTAAATCCTTTAACGAGGATCTATTGGAGGGCAAGTCTGGTGCCAGCAGCCGCGG
TAATTCCAGCTCCAATAGCGTATATTAAAGTTGTTGCGGTTAAAAA-
GCTCGTAGTTGGTTCTGCGTGTCACGCTGTCGGTTCACCGCCTGTCGGTGCAACTGGCATGCC-
GTGGCATGTCCTGTCGGTGGCCGACAGGC-TTCGCG-GCGCGCCCTATG-------CGTT---CACACGT-G-
TAGGTCGTTGC--GCC-GCGTTGTCC-TTGTAGG-CCGGCT-CAATCCTACCGCGGTGCT-
CTTTGCCGAGTGTCGAGGTAGGCCGACACT-
GTTTACTTTGAACAAATTAGAGTGCTTAAAGCAGGCTCTGAAAGC-
AGCCTGAATAATGGTGCATGGAATAATGGAACAGGACCTTGGTTCTATTTTGTT-GGTTTAGGA-
AACCAAGGTAATGATCAATAAGGA-CAGGCGGGGGCATTCGTATTGCGACGTTAGA-----------------
-GGTGAAATTCTTGG-
ATCGTCGCAAGACGCACTAGAGCGAAAGCATTTGCCAAGTATGTCTTAATTGATCAAGAACGAAAGTTAGAGG
TTCGAAGGCGATCAGATACCGCCCTAGTTCTAACCATAAACGATGCCAGCCAGCGATCCGCCGATGTTCCTCC
GATGACTCGGCGGGGAGCTT-
TCCCCGGGAAACCAAAGCTTTTGGGTTCCGGGGGAAGTATGGTTGCAAAGCTGAAACTTAAAGGAATTGACGG
AAGGGCACCACCAGGAGTGGAGCCTGCGGCTTAATTTGACTCAACACGGGAAACCTCACCAGGCCCGGACATT
GGAA-GGATTGACAGAT-CGATAGCTCTTT-CTCGATT-CAGTGGGT-
AGTGGTGCATGGCCGTTCTTAGTTGGTGAAGTGATTTGTCTGGTTAATTCCGATAACGAACGAGACTCTGACC
TATTAACTAGGCGCTC-CCGGTATAC-AAA-CCTACCGGCGACAT-
TTATTCTTCTTAAAGGGACAGGCGGCTCTTAGCCGCACGAGATTGAGCAATAACAGGTCTGTGATGCCCTTAG
ATGTTCTGGGCCGCACGCGCGCTACACTGAAGGAATCAGCGTGTGC--
TCCCCTGGCCGAAAGGTCCGGGCAACCCGCTGAACCTCCTTCGTGCTAGGAATTGGGGCTTGCAATTGTTCCC
CATGAACGAGGAATTCCCAGTAAGCGCGAGTCATAAGCTCGCGTTGATTACGTCCCTGCCCTTTGTACACACC
GCCCGTCGCTACTACCGATTGAATGATTTAGTGAGGTCTTCGGACC-GGCGGTA--AT-TGGC-ACC--GGC-
----CTGTCCGG---TATCGGT-TTCGCTGGAAA-
GATGACCGAACTTGGTCATTTAGAGGAAGTAAAAGTCGTAACAAGT-GCGCGAGTCA--TTGGGTGT---
AAAACCTAAAGGCGCAATGAAAGT-GTC-------------AGGAGGGGCTCCT-------------
TTAGGAGC-----GCTCTCCGTAGGGAGGATGTGGTTCC----GCACACGTA-TATA--
TTTATGGTCGGATCACCGCACTCCCGGGGCGTCTC--GTTCTC-GTTACG-AG----

TGGAGACGCACCAGGAGCGCACACGCTGGTACCCGAAAGATGGTGAACTATGCCTGGCCAGGACGAAGCCAGG
GGAAACCCTGGTGGAGGTCC--GAAGCG-ATTCTG--ACGTGCAAATCG-
ATCGTCTGAGCTGGGTATAGGGGCGAAAGACCAATCGAACCATCTAG--TTAAATTT------
TAGCTGGTTCCCTCTGAATGATA-TTTAA-----TTA--
AAATAGCCGCAGTATACTGACTGTGCGAAGGTAGCATAATCATTTGTCTATTAATTGTAGGCTAGTATGAAGG
GTTTGATGAAATATTTGCTTTCTTTAATTATACTATTGA---
TGAATTTAATATTTGGGTTAAAAAGCTTAAATTCAT-AATTGGGACGAGAAGACCCTATAGAG--
TTTATATTCATGTTTG----------TTATATTAGTTTATTATGG-------TATAA-TTAATTAATA--
TAAGGTGAATTTTGTTGGGGTGACAAGGGGA-TTTATTTAACTC-CTCTTATAATTAA---
TTATTATTGATTTGTAT--ATATATAATCCTG-----TTATTATGGAA--
TAAAGGTTAAATTACCTTAGGGATAAC
Canthophorus_impressus
ATGCAAGTCTCAGTACGAGCTTACA-
TAAAGTGAAACCGCAAAAGGCTCAGTAAACCGGTCATAGTTTACCGGA-TATATG-CCACTC-
ACGTGGATAACTGTGGTAATTCTAGAGCTAATACATGCAATC-AAGTCCCGACCAGTG---
ATGGAAAGGACGCTTTTATTGGATCAAAACCTTTCGCCCGGCTT-GCCCGGGC------
GTCGGCTTGATGACTCT-AGATAAC-ACCACA-GATCGCACGGTCATTGTACCGGC-
GACGTATCCGTCAAATGTCTGACTTATCAGCTGTCGATGGTAGACTCTGTGTCTACCATGGCGCTCACGGGTA
ACGGGGAATCAGGGTTCGATTCCGGAGAGGGAGCCTGAGAAACGGCTACCACATCCAAGGAAGGCAGCAGGCA
CGCAAATTACCCACTCCCGGCACGGGGAGGTAGTGACAAAAAATAACGATACGGGACTCATCCGAGGCCCCGT
AATCGGAATGAGAACACCCTAAATCCTTTAACAAGGATCTATTGGAGGGCAAGTCTGGTGCCAGCAGCCGCGG
TAACTCCAGCTCCAATAGCATATATTAAAGTTGTTGCGGTTAAAAA-
GCTCGTAGTTGGTTCTGCGTGCCGCGCTGTCGGTTCACCGCCTGTCGGTGCAACTGGCATGCC-
GCGGCATGTCCTGTCGGTGGCCTACTGGTCTTCGAG-GCCCACCCTATG-------CGTT---CACACGT-G-
TAGGTCGTTGG--GCTGC-GTTGTAC-TCGTAGG-CCGGCT-CAATCCTACCGCGGTGCT-
CTTTACCGAGTGTCGAGGTAGGCCGACACT-
GTTTACTTTGAACAAATTAGAGTGCTTCAAGCAGGCTCTGAAAGC-
AGCCTGAATAGTGGTGCATGGAATAATGGAACAGGACCTTGGTTCTATTTTGTT-GGTTTTCGGA-
ATCCAAGGTAATGATCAATAAGGA-CAGGCGGGGGCATTCGTATTGCGACGTTAGA-----------------
-GGTGAAATTCTTGG-
ATCGTCGCAAGACGCACTAGAGCGAAAGCATTTGCCAAGTATGTCTTAATTGATCAAGAACGAAAGTTAGAGG
TTCGAAGGCGATCAGATACCGCCCTAGTTCTAACCATAAACGATGCCAGCCAGCGATCCGCCGATGTTCCTCC
GATGACTCGGCGGGGAGCTT-
CCCCCGGGAAACCAAAGCTTTTGGGTTCCGGGGGAAGTATGGTTGCAAAGCTGAAACTTAAAGGAATTGACGG
AAGGGCACCACCAGGAGTGGAGCCTGCGGCTTAATTTGACTCAACACGGGAAACCTCACCAGGCCCGGACATT
GGAA-GGATTGACAGAT-CGATAGCTCTTT-CTCGATT-CAGTGGGT-
AGTGGTGCATGGCCGTTCTTAGTTGGTGGACTGATTTGTCTGGTTAATTCCGATAACGAACGAGACTCTAACC
TGTTAACTAGGCGTCC-CCGGTATAC-ATA-CCTACCGGCGAGAC-
TTATTCTTCTTAAAGGGACAGGCGGCTCTTAGCCGCACGAGATTGAGCAATAACAGGTCTGTGATGCCCTTAG
ATGTTCTGGGCCGCACGCGCGCTACACTGAAGGAATCAGCGTGTTC--
TCCCCTGTCCGAAAGGACCGGGCAACCCGCTGAACCTCCTTCGTGCTAGGAATTGGGGCTTGCAATTGTTCCC
CATGAACGAGGAATTCCCAGTAAGCGCGAGTCATAAGCTCGCGTTGATTACGTCCCTGCCCTTTGTACACACC
GCCCGTCGCTACTACCGATTGAATGATTTAGTGAGGTCTTCGGACC-GGCGGTA--AC-AGGC-ATA--
GGCT-----CGCCCTA---TATCTGT-TCCGCTGGAAA-
GATGACCGAACTTGATCATTTAGAGGAAGTAAAAGTCGTAACAAGG-TTTCCAGTC---TTGGGTG-----
AGACCTAAAGGCGCAATGAAAGT-GTC-------------AGGGGGGCCCTCCT------------
CCGGGAGG----GTCCCCCCGCTGGGAAGATGGGCCCACC---GTACAATTCCT--------TGT--
CGGAGCCCCGCACTCCCGGGGCGTCTC--GTTCTC-ATTGCG-AG----
TAGAGACGCACCAGGAGCGCACACGCTGGTACCCGAAAGATGGTGAACTATGCCTGGCCAGGACGAAGCCAGG
GGAAACCCTGGTGGAGGTCC--GAAGCG-ATTCTG--ACGTGCAAATCG-
ATCGTCTGAGCTGGGTATAGGGGCGAAAGACCAATCGAACCATCTGATAAAATATTG--
ATTTTAGGTCAGGCCTGCTCAGTGATTATAAA------
AAATTAAATAGCCGCAGTATTTTGACTGTGCAAAGGTAGCATAATCATTTGTCTATTAATTGTAGGCTAGTAT
GAATGGTTTGATGAGATATTAACTTTCTTTA---TATATTTTTT---ATAAATTAATATATTAAATATCC--
TTTAATTATGG-AAATAAGATTAAATTACCTTAGGGAT--AACAGCGTAATTCTTT--------
TGGAGAGTTCATATCGATAA-AAGATT----AAAAA-ATTATGTAAT----
TAAATTTTGTTGGGGTGATAGGGAAA-ATTTCATAACTTTCTTTATATATTT---GTTATAAATTAATATAT-
----TAAATATCCTT-----TATTAAGGAA---ATAAGATTAAATTACCTTAGGGATAAC

Canthophorus_niveimarginatus
ATGCAAGTCTCAGTACGAGCTTACT-
TAAAGTGAAACCGCAAAAGGCTCAGTAAACCGGTCATAGTTTACCGGA-TATATG-CCACTC-
ACGTGGATAACTGTGGTAATTCTAGAGCTAATACATGCAATC-AAGTCCCGACCAGCG---
ATGGAAAGGACGCTTTTATTGGATCAAAACCATTCGCCCGGCTT-GCCCGGGC------
GTCTGCTTGATGACTCT-AGATAAC-ACCACA-GATCGCACGGTCATTGTACCGGC-
GACGTATCCGTCAAATGTCTGACTTATCAGCTGTCGATGGTAGACTCTGTGTCTACCATGGCGCTCACGGGTA
ACGGGGAATCAGGGTTCGATTCCGGAGAGGGAGCCTGAGAAACGGCTACCACATCCAAGGAAGGCAGCAGGCA
CGCAAATTACCCACTCCCGGCACGGGGAGGTAGTGACAAAAAATAACGATACGGGACTCATCCGAGGCCCCGT
AATCGGAATGAGAACACCCTAAATCCTTTAACGAGGATCCATTGGAGGGCAAGTCTGGTGCCAGCAGCCGCGG
TAACTCCAGCTCCAATAGCATATATTAAAGTTGTTGCGGTTAAAAA-
GCTCGTAGTTGGTTCTGCGTGCCGCGCTGTCGGTTCACCGCCTGTCGGTGCAACTGGCATGCC-
GCGGCATGTCCTGTCGGTGGCCTACTGGTCTTCGAG-GCCCACCCTATG-------CGTT---CACTCGT-G-
TAGGTCGTTGG--GCTGC-GTTGTAC-TCGTAGG-CCGGCT-CAATCCTACCGCGGTGCT-
CTTTACCGAGTGTCGAGGTAGGCCGACACT-
GTTTACTTTGAACAAATTAGAGTGCTTCAAGCAGGCTCTGAAAGC-
AGCCTGAATAGTGGTGCATGGAATAATGGAACAGGACCTTGGTTCTATTTTGTT-GGTTTTCGGA-
ATCCAAGGTAATGATCAATAAAGA-CAGGCGGGGGCATTCGTATTGCGACGTTAGA-----------------
-GGTGAAATTCTTGG-
ATCGTCGCAAGACGCACTAGAGCGAAGGCTTTTGCCAAGTATGTCTTAATTGATCAAGAACGAAAGTTAGAGG
TTCGAAGGCGATCAGATACCGCCCTAGTTCTAACCATAAACGATGCCAGCCAGCGATCCGCCGATGTTCCTCC
GATGACTCGGCGGGGAGCTT-
CCCCCGGGAAACCAAAGCTTTTGGGTTCCGGGGGAAGTATGGTTGCAAAGCTGAAACTTAAAGGAATTGACGG
AAGGGCACCACCAGGAGTGGAGCCTGCGGCTTAATTTGACTCAACACGGGAAACCTCACCAGGCCCGGACATT
GGAA-GGATTGACAGAT-CGATAGCTCTTT-CTCGATT-CAGTGGGT-
AGTGGTGCATGGCCGTTCTTAGTTGGTGGATTGATTTGTCTGGTTAATTCCGATAACGAACGAGACTCTAACC
TGTTAACTAGGCGTCC-CCGGTATAC-ATA-CCTACCGGCGAGAC-
TTATTCTTCTTAAAGGGACAGGCGGCTCTTAGCCGCACGAGATTGAGCAATAACAGGTCTGTGATGCCCTTAG
ATGTTCTGGGCCGCACGCGCGCTACACTGAAGGAATCAGCGTGTTC--
TCCCCTGTCCGAAAGGACCGGGCAACCCGCTGAACCTCCTTCGTGCTAGGAATTGGGGCTTGCAATTGTTCCC
CATGAACGAGGAATTCCCAGTAAGCGCGAGTCATAAGCTCGCGTTGATTACGTCCCTGCCCTTTGTACACACC
GCCCGTCGCTACTACCGATTGAATGATTTAGTGAGGTCTTCGGACC-GGCGGTA--AC-AGGC-ATA--
GGCT-----CGCCCTG---TATCTGT-TCCGCTGGAAA-
GATGACCGAACTTGATCATTTAGAGGAAGTAAAAGTCGTAACAAGG-TTTCCAGTCA--TTGGGTG-----
AAACCTAAAGGCGCAATGAAAGT-GTC-------------AGGGGGGCACTCCT------------
CCGGGAGG----GCCCCCCCGCTGGGAAGATGGGCCCACC---GTACAATTCCT--------TGT--
CGGAGCCCCGCACTCCCGGGGCGTCTC--GTTCTC-ATTGCG-AG----
TTGAGACGCACCAGGAGCGCACACGCTGGTACCCGAAAGATGGTGAACTATGCCTGGCCAGGACGAAGCCAGG
GGAAACCCTGGTGGAGGTCC--GAAGCG-ATTCTG--ACGTGCAAATCG-
ATCGTCTGAGCTGGGTATAGGGGCGAAAGACCAATCGAACCATCTGA---GATATTG--
ATTATAGGTCAGGCCTGCTCAGTGATTATTAA------
AAATTAAATAGCCGCAGTATTTTGACTGTGCAAAGGTAGCATAATCATTTGTCTATTAATTGTAGGCTAGTAT
GAATGGTTTAATGAGATATTAACTTTCTTTACTTTACATTTATT---
TGAATTTAATATTTAAGTTAAAAAGCTTAGATGTTT-TTGTGGGACGAGAAGACCCTATAGAA--
TTTTATTTAATTATTG---------ATTTATTTTTAATTTATTA-ATCATT----AAAAA-ATTATGTAAT--
--TAAATTTTGTTGGGGTGATAGGGAAA-ATTTCATAACTTTCTTTATATATTT---
GTTATAAATTAATATAT-----TAAATATCCTT-----TATTAAGGAA---
ATAAGATTAAATTACCTTAGGGATAAC
Chilocoris_confusus_assmuthi
ATGCAAGTCTCAGTACGAGCTCATT-
TAAAGTGAAACCGCAAAAGGCTCAGTAAACCGGTCATAGTTTACCGGA-TATATG-CCACTA-
ACGTGGATAACTGTGGTAATTCTAGAGCTAATACATGCAAAC-AAGTCCCGACCAGCG---
ATGGAAAGGACGCTTTTATTGGATCAAAACCATTCGCCCGGCTC-GCCCGGGC------
GTCTGCTTGATGACTCT-AGATAAC-ACCACA-GATCGCACGGTCGCAGTACCGGC-
GACGTATCCGTCAAATGTCTGACTTATCAGCTGTCGATGGTAGACTCTGTGTCTACCATGGCGCTCACGGGTA
ACGGGGAATCAGGGTTCGATTCCGGAGAGGGAGCCTGAGAAACGGCTACCACATCCAAGGAAGGCAGCAGGCA
CGCAAATTACCCACTCCCGGCACGGGGAGGTAGTGACAAAATATAACGATACGGGACTCATCCGAGGCCCCGT
AATCGGAATGAGAACACCCTAAATCCTTTAACGAGGATCTATTGGAGGGAAAGTCTGGTGCCAGCAGCCGCGG

TAATTCCAGCTCCAATAGCGTATATTAAAGTTGTTGCGGTTAAAAA-
GCTCGTAGTTGGTTCTGCGTGCCACGCTGTCGGTTCACCGCCTGTCGGTGCAACTGGCATGCCCGTGGCATGT
CCTGCCGGTGGCCGACGGGC-TTCTGG-CGGCGCCCCGGTCGCA---CGTTAACCACACGTCC-
CGGTGTGCTGCC-GCCCA-GTCGTAC-CCGTGGG-CCGGCT-CAATCCTACCGCGGTGCT-
CTTTGCCGAGTGTCGAGGTAGGCCGGCACT-
GTTTACTTTGAACAAATTAGAGTGCTTCAAGCAGGCTATGAAAGC-
AGCCTGAATAGTGGTGCATGGAATAATGGAACAGGACCTCGGTTCTATTTTGTT-GGTTTCCGGA-
ATCCGAGGTAATGATCAATAAGGA-CAGGCGGGGGCATTCGTATTGCGACGTTAGA-----------------
-GGTGAAATTCTTGG-
ATCGTCGCAAGACGCACTAGAGCGAAAGCATTTGCCAAGTATGTCTTAATTGATCAAGAACGAAAGTTAGAGG
CTCGAAGGCGATCAAATACCACCCTAGTTCTAACCATAAACGATGCCAGCCAGCGATCCGCCGATGTTCCTCC
GATGACTCGGCGGGGAGCTT-
TCCCCGGGAAACCAAAGCTTTTGGGTTCCGGGGGAAGTATGGTTGCAAAGCTGAAACTTAAAGGAATTGACGG
AAGGGCACCACCAGAAGTGGAGCCTGCGGCTTAATTTGACTCAACACGGGCAAGCTCACCAGGCCCGGACATT
GGAA-GGATTGACAGAT-CGATAGCTCTTT-CTCGATT-CAGTGGGT-
AGTGGTGCATGGCCGTTCTTAGTTGGTGGACTGATTTGTCTGGTTAATTCCGATAACGAACGAGACTCTGACC
TATTAACTAGGCGTTT-CCGGTATAA-ATATACTACCGGCGAAAT-
TTATTCTTCTTAAGGGGACAGGCGGCTCTCAGCCGCACGAGATTGAGCAATAACAGGTCAGTGATGCCCTTAG
ATGTTCTGGGCCGCACGCGCGCTACACTGAAGGAATCAGCGTGTGC--
TCCCCTGTCCGAAAGGTCCGGGCAACCCGCTGAACCTCCTTCGTGCTAGGAATTGGGGCTTGCAATTGTTCCC
CATGAACGAGGAATTCCCAGTAAGCGCGAGTCATAAGCTCGCGTTGATTACGTCCCTGCCCTTTGTACACACC
GCCCGTCGCTAATACCGATTGAATGATTTAGTGAGGTCTTCGGACC-GGCGGTT--AC-CGGC-ACC--
GGCGTTCCACGCTCGG---TATCGGT-TCCGCTGGAAA-
GATGACCGAACTTGATTATTTAGAGGAATTAAAAGTCGTAACAAGG-TTTCCAGTCA--TTGGGTGT----
AAACCTAAAGGCGCAATGAAAGT-GTC-------------AGAG------------------------
GGGAGCCTAGCGCTCCCCGCAGGGAGGATGGGCTT-CC---GTACAATTTTT--------TGT--
CGGGGCCCCGCACTCCCGGGGCGTCTC--GTTCTC-ATCACG-AG----
TAGAGACGCACCAGGAGCGCACACGCTGATACCCGAAAGATGGTGAACTATGCCTGGCCAGGACGAAGCCAGG
GGAAACCCTGGTGGAGGTCC--GAAGCG-ATTCTG--ACGTGCAAATCG-
ATCGTCTGAGCTGGGTATAGGGGCGAAAGACCAATCGAACCATCTGA-GTA--TTT--
AATTTTAAGTCTTGCCTGCTCAATGATAAAAT--------
AATTAAATAGCCGCAGTATTTTGACTGTGCAAAGGTAGCATAATCATTTGTCCTTTAATTGGGGGCTAGTATG
AATGGTTTGATGAAATATAAACTTTCTTTATTATA-ATGGGTAT--
TGAATTTAATTTTTTAGTTAAAAGGCTGAAATAGTT-TTATGGGACGAGAAGACCCTATAGAA--
TTTTATTTATTTTTAT---------TTTTTACTTACTATTGGGTTAGTAA-----
TTATTTTATAATAAATAGTAA--ATTTTGTTGGGGTGACAGGGAAA-TTTTAATAACTTTCTTT-
AAAATTAG--TTTACTAATTAGTATAA-----TAATGATCTTT-----CATTGGTGATT--
AAAAGACTAAATTACCTTAGGGATAAC
Chilocoris_laevicollis
ATGCAAGTCTCAGTACGAGCTCATT-
TAAAGTGAAACCGCAAAAGGCTCAGTAAACCGGTCATAGTTTACCGGA-TATATG-CCACTA-
ACGTGGATAACTGTGGTAATTCTAGAGCTAATACATGCAAAC-AAGTCCCTACCAGCG---
ATGGAAAGGACGCTTTTATTGGATCAAAACCATTCGCCCGGCTC-GCCCGGGC------
GTCTGCTTGATGACTCT-AGATAAC-ACCACA-GATCGCACGGTCATAGTACCGGC-
GACGTATCCGTCAAATGTCTGACTTATCAGCTGTCGATGGTAGACTCTGTGTCTACCATGGCGCTCACGGGTA
ACGGGGAATCAGGGTTCGATTCCGGAGAGGGAGCCTGAGAAACGGCTACCACATCCAAGGAAGGCAGCAGGCA
CGCAAATTACCCACTCCCGGCACGGGGAGGTAGTGACAAAATATAACGATACGGGACTCATCCGAGGCCCCGT
AATCGGAATGAGAACACCCTAAATCCTTTAACGAGGATCTATTGGAGGGAAAGTCTGGTGCCAGCAGCCGCGG
TAATTCCAGCTCCAATAGCGTATATTAAAGTTGTTGCGGTTAAAAA-
GCTCGTAGTTGGTTCTGCGTGCCACGCTGTCGGTTCGCCGCCTGTCGGCGCAACTGGCATGCCCGTGGCATGT
CCTGCCGGTGGCCGACGGGC-TTCTGGACGGCGGCCCCGCCCCG---TGTTAACAGCAC-
TGGACGGGTAGTCGCC-GCTC--GTTGTAC-CCGTAGG-CCGGCT-CAATCCTACCGCGGTGCT-
CTTTGCCGAGTGTCGAGGTAGGCCGGCACT-
GTTTACTTTGAACAAATTAGAGTGCTTCAAGCAGGCTATGAAAGC-
AGCCTGAATAGTGGTGCATGGAATAATGGAAAAGGACCTTGGTTCTATTTTGTT-GGTTTCCGGA-
ATCCAAGGTAATGATCAATAAGGA-CAGGCGGGGGCATTCGTATTGCGACGTTAGA-----------------
-GGTGAAATTCTTGG-
ATCGTCGCAAGACTCACTAGAGCGAAAGCATTTGCCAAGTATGGCTTAATTGATCAAGAACGAAAGTTAGAGG

CTCGAAGGCGATCAGATACCGCCCTAGTTCTAACCATAAACGATGCCAGCCAGCGATCCGCCGATGTTCCTCC
GATGACTCGGCGGGGAGCTT-
TCCCCGGGAAACCAAAGCTTTTGGGTTCCGGGGGAAGTATGGTTGCAAAGCTGAAACTTAAAGGAATTGACGG
AAGGGCACCACCAGCAGTGGAGCCTGCGGCTTAATTTGACTCAACACGGGCAAGCTCACCAGGCCCGGACATT
GGAA-GGATTGACAGAT-CGATAGCTCTTT-CTCGATT-CAGTGGGT-
AGTGGTGCATGGCCGTTCTTAGTTTGTGGACTGATTTGTCTGTATAATTCCGATAACGAACGAGACTCTGACC
TATTAACTAGGCGTTT-CCGGTATAA-TTTTACTACCGGCGAAAT-
TTATTCTTCTTAAGGGGACAGGCGGCTCTCAGCCGCACGAGATTGAGCAATAACAGGTCAGTGATGCCCTTAG
ATGTTCTGGGCCGCACGCGCGCTACACTGAAGGAATCAGCGTGTGC--
TCCCCTGTCCGAAAGGTCCGGGCAACCCGCTGAACCTCCTTCGTGCTAGGAATTGGGGCTTGCAATTGTTCCC
CATGAACGAGGAATTCCCAGTAAGCGCGAGTCATAAGCTCGCGTTGATTACGTCCCTGCCCTTTGTACACACC
GCCCGTCGCTAATACCGATTGAATGATTTAGTGAGGTCTTCGGACC-GGCGGTT--AC-CGGC-ACC--
GGCGTTCCGCGCTCGG---TATCGGT-TCCGCTGGAAA-
GATGACCGAACTTGATTATTTAGAGGAATTAAAAGTCGTAACAAGG-TTTCGAGTCA--TTGGGTGA----
AAACCTATAGGCGCAATGAAAGT-GTC-------------AGGGTGCCCCCC-------------
GTTCGCGGGTGAGGGC--CCCGCAGGGAGGATGGGCCG-CC---GCACACGTCAAAA------TGT--
CGGCGCCCCGCACTCCCGGGGCGTCTC--GTACCA-CGTTCGCGG----
TAGAGACGCACCAGGAGCGCACACGCTGGTATCCGAAAGATGGTGAACTATGCCTGGCCAGGACGAAGCCAGG
GGAAACCCTGGTGGAGGTCC--GAAGCG-GTTCTG--ACGTGCAAATCG-
ATCGTCTGAGCTGGGTATAGGGGCGAAAGACCAATCGAACCATCTGATAA---ATT--
AATATTAAGTCGGACTCGCTCCGTGACAAAGG--------
TAGTATATAGCCGCAGTATTTTGACAGTGCAAAGGTAGCATAATCATTTGTCGGTTAATTGTGGTCTAGATTG
AATGGTTTGATAAAGACTAGTCTGTCTATAGGTTG-AAAATTAT--
AGAATTTAATATAAGAGTTAAAAGCCTGAAATATTT-TTAAGGGACGAGAAGACCCTATAGAA--
TACAGGAGTTAATATT---------AA--GAATATAAGA-ATGTTAAAA-------
TAGGAATTGGTGATATATGATTAAAA-GTTGGGGTGACAGGGAAA-TATATTGAACTTTCTTT-ATAAATTA-
-ATTGTTAATTATCCTAT-----TATTGATCTTC-----CAATAGTGATT--
TTTTAAATAAATTACCTTAGGGATAGC
Chilocoris_piceus_neozealandicus
ATGCAAGTCTCAGTACGAGCTCATT-
TAAAGTGAAACCGCAAAAGGCTCAGTAAACCGGTCATAGTTTACCGGA-TATATG-CCACTA-
ACGTGGATAACTGTGGTAATTCTAGAGCTAATACATGCAAGC-AAGTCCCGACCAGCG---
ATGGAAAGGACGCTTTTATTGGACCAAAACCATTCGCCCGGCTC-GCCCGGGC------
GTCTATTTGATGACTCT-AGATAAC-ACCACA-GATCGCACGGTCGCAGTACCGGC-
GACGTATCCGTCAAATGTCTGACTTATCAGCTGTCGATGGTAGACTCTGTGTCTACCATGGCGCTCACGGGTA
ACGGGGAATCAGGGTTCGATTCCGGAGAGGGAGCCTGAGAAACGGCTACCACATCCAAGGAAGGCAGCAGGCA
CGCAAATTACCCACTCCCGGCACGGGGAGGTAGTGACAAAATATAACGATACGGGACTCATCCGAGGCCCCGT
AATCGGAATGAGAACACCCTAAATCCTTTAACGAGGATCTATTGGAGGGAAAGTCTGGTGCCAGCAGCCGCGG
TAATTCCAGCTCCAATAGCGTATATTAAAGTTGTTGCGGTTAAAAA-
GCTCGTAGTTGGTTCTGCGTGCCACGCTGTCGGTTCACCGCCTGTCGGTGCAACTGGCATGCCCGTGGCATGT
CCTGCCGGTGGCCGACGGGC-TCCTGG-CGGCGCCCCGGTCGCA---CGTTAACCACACGTCC-
CGGTCGGTCGCC-CGCCA-GTCGTGC-CCGTGGG-CCGGCT-CAATCCTACCGCGGTGCT-
CTTTGCCGAGTGTCGAGGTAGGCCGGCACT-
GTTTACTTTGAACAAATTAGAGTGCTTCAAGCAGGCTAAGAAATC-
AGCCTGAATAGTGGTGCATGGAATAATGGAACAGGACCTCGGTTCTATTTTGTT-GGTTTCCGGA-
ATCCGAGGTAATGATCAATAAGGA-CAGGCGGGGGCATTCGTATTGCGACGTTAGA-----------------
-GGTGAAATTCTTGG-
ATCGTCGCAAGACGCACTAGAGCGAAAGCATTTGCCAAGTATGTCTTAATTGATCAAGAACGAAAGTTAGAGG
CTCGAAGGCGATCAGATACCGCCCTAGTTCTAACCATAAACGATGCCAGCCAGCGATCCGCCGATGTTCCTCC
GATGACTCGGCGGGGAGCTT-
TCCCCGGGAAACCAAAGCTTTTGGGTTCCGGGGGAAGTATGGTTGCAAAGCTGAAACTTAAAGGAATTGACGG
AAGGGCACCACCAGAAGTGGAGCCTGCGGCTTAATTTGACTCAACACGGGCAAGCTCACCAGGCCCGGACATT
GGAA-GGATTGACAGAT-CGATAGCTCTTT-CTCGATT-CAGTGGGT-
AGTGGTGCATGGCCGTTCTTAGTTGGTGGACTGATTTGTCTGGTTAATTCCGATAACGATCGAGACTCTGACC
TATTAACTAGGCGTTT-CCGGTATAA-ATATACTACCGGCGAAAT-
TTATTTTTCTTAAGGGGACAGGCGGCTTTCATCCGCGCGTCATTGATCATTAACAGATCACAGATGCCCTTAG
ATGTTATGGGCTGCCGGCGGGGCACACTGAAGGAATCAGCGTGTAC--
TCCCCTGTCCGAAAGGTCCGGGCAACCCGCTGAACCTCCTTCGTGCTAGGAATTGGGGCTTGCAATTGTTCCC

CATGAACGAGGAATTCCCAGTAAGCGCGAGTCAAAAGCTCGCATTGATTACGTCCCTGCCCTTTGTACACACC
GCCCGTCGAGAATACCGATTGAATGATTTAGTGAGGTCTTCGGACC-GGCGGTT--AC-CGGC-ACC--
GGCTCTCCGCGTTCGG---TATCGGA-TGCGCTGGAAA-
GATGAGCGAACTTGATTATTTAGAGGAATTAAAAGTCGTAACAAGG-TTTCGAGTCA--TTGGGCGA----
AAACCCATAGGCGCAATGAAAGT-GTC-------------AGGGCGATTCCCCCGCGCCGCC--
TCCCGGCGGCCGGGGGGCGCCCCCGGGGAGGATGGGCCTGCC---GTACACGTAACAG------CGT--
CGGCGCCCCGCACTCCCGGGGCGTCTC--GCGCCA-CGCCCGTGG----
CGGAGACGCACCAGGAGCGCACACGCTGGTACCCGAAAGATGGTGATCTATGCCTGGCCAGGACGAAGCCAGG
GGAAACCCTGGTGGAGGTCC--GAAGCG-GTTCTG--ACGTGCAAATCG-
ATCGTCTGAGCTGGGTATAGGGGCGAAAGACCAATCGAACCATCTGATATT--ATT--
GATATTAAGTCTGACCTGCTCAATGATTGATTT-------
AATTAAATAGCCGCAGTATTTTGACTGTGCAAAGGTAGCATAATCATTTGTCCTTTAATTGTGGGCTAGAATG
AATGGTTTGATGAAATATAAACTTTCTTTATTATA-ATTATCAT--
AGAATTTAATATTTTAGTTAAAAAGCTAAAATGGTT-TTATGGGACGAGAAGACCCTATAGAA--
TTTTATATGTTATAAT---------AATTAATTTGTT--TAAGTGAAAAT-----
ATATGAAATTAATTATTATGATTATTATGTTGGGGTGACAGGGAAA-TTTAATAAACTTTCTTT-
ATTTAATT--TTTATTGATTAATATAT-----TAAAGATCCTT-----TACGTAGGATT--
ATAAGATTAAATTACCTTAGGGATAAC
Cletus_punctiger
ATGCAAGTCTCAGTACGAGCTGCAT-
TAAAGTGAAACCGCAAAAGGCTCAGTAAACCGGTCATAGTTTACCGGA-CATATA-CCACTT-
ACGTGGATAACTGTGGTAATTCTAGAGCTAATACATGCAAAC-AAGTCCCGACCAGCG---
ATGGAGAGGACGCTTTTATTTGATCAAAACCATTCGCCCGGCTT-GCCCGGGC------
GTCGGCTTGATGACTCT-AGATAAC-ACCACA-GATCGCACGGTCACAGTACCGGC-
GACGTATCCTTCAAATGTCTGACTTATCAGCTGTCGATGGTAGACTCTGTGTCTACCATGGCGCTCACGGGTG
ACGGGGAATCGGGGTTCGATTCCGGAGAGGGAGCCTGAGAAACGGCTACCACATCCAAGGAAGGCAGCAGGCA
CGCAAATTACCCACTCCCGGCACGGGGAGGTAGTGACAAAAAATAACGATACGGGACTCATCCGAGGCCCCGT
AATCGGAATGAGAACACCCTAAATCCTTTAACGAGGATCTATTGGAGGGCAAGTCTGGTGCCAGCAGCCGCGG
TAATTCCAGCTCCAATAGCGTATATTAAAGTTGTTGCGGTTAAAAA-
GCTCGTAGTTGGTTCTGCGTGCCACGCTGTCGGTTCACCGCCTGCCGGTGCAACTGGCATGCC-
GTGGCATGTCCTGTCGGTGGCGGACAGGC-TCCTCG-GCGTGCCCT-TGCGT----CTT-AC-GGCGTTT---
-AGGTCGCTGC--GCCGCGT-TGTCC-TTGTTCG-CCGGCT-CAATCCTACCGCGGTGCT-
CTTTACCGAGTGTCGAGGTAGGCCGACACT-
GTTTACTTTGAACAAATTAGAGTGCTTAAAGCAGGCTCTTAAATC-
AGCCTGAATAGTGGTGCATGGAATAATGGAAAAGGACCTTGGTTCTATTTTGTT-GGTTTACGGA-
ATCCAAGGTAATGATCAATAAGGA-CAGGCGGGGGCATTCGTATTGCGGCGTTAGA-----------------
-GGTGAAATTCTTGG-
ATCGTCGCAAGACGCACTAGAGCGAAAGCATTTGCCAAGTATGTCTTAATTGATCAAGAACGAAAGTTAGAGG
CTCGAAGGCGATCAGATACCGCCCTAGTTCTAACCATAAACGATGCCAGCCAGCGATCCGCCGATGTTCCTCC
GATGACACGGCGGGGAGCTT-
TCCTCGGGAAACCAAAGCTTTTGGGTTCCGGGGGAAGTATGGTTGCAAAGCTGAAACTTAAAGGAATTGACGG
AAGGGCACCACCAGGAGTGGAGCCTGCGGCTTAATTTGACTCAACACGGGAAACCTCACCAGGCCCGGACATT
GGAA-GGATTGACAGAT-CGATAGCTCTTT-CTCGATT-CAGTGGGT-
AGTGGTGCATGGCCGTTCTTAGTTGGTGGACTGATTTGTCTGGTTAATTCCGATAACGAACGAGACTCTGACC
TATTAACTAGGCGTTG-CCGGTATAAATCA-GCTACCGGCGAGAT-
TTTTTCTTCTTAAGGGGACAGGCGGCTCTCAGCCGCACGAGATTGAGCAATAACAGGTCTGTGATGCCCTTAG
ATGTTCTGGGCCGCACGCGCGCTACACTGAAGGAATCAGCGTGTGC--
TCCCCTGGCCGAAAGGTCCGGGTAACCCGCTGAACCTCCTTCGTGCTAGGAATTGGGGCTTGCAATTGTTCCC
CATGAACGAGGAATTCCCAGTAAGCGCGAGTCATAAGCTCGCGTTGATTACGTCCCTGCCCTTTGTACACACC
GCCCGTCGCTACTACTGATTGAATGATTTAGTGAGGTCTTCGGACC-GGCGATA--AC-CGGCCACC--
GACTC-----GTTCGG---TACCGAT-TTCGCTGGAA--
GATGACCGAACTTGATTATTTAGAAGAAGTAAAAGTCGTAACAAGG-TTTCCAGTCA--CTGGGTGT---
AAAACCTAAAGGCGTAATGAAAGT-GTCTG---------------AGGGCGCTC-----------------
GCGT--------CCTCGGCGGGAGGATGGGCTC-CC---GGCTTTT-AGC-------------
CGGTGCCCCGCACTCCCGGGGCGTTTC--ATTCTCGATTCCG-AG----
TCGAGACGCACCAGGAGCGCACACGCTGGTACCCGAAAGATGGTGAACTATGCCTGGCCAGGACGAAGCCAGG
GGAAACCCTGGTGGAGGTCC--GAAGCG-ATTCTG--ACGTGCAAATCG-
ATCGTCTGAGCTGGGTATAGGGGCGAAAGACCAATCGAACCATCTAG----ATTTT-

TAATTTTAAGTCTAATCTGCCCGGTGATTAA------------
TTAAACGGCCGCAGTATTCTGACTGTGCGAAGGTAGCATAATCGTTTGTCTCTTAATTGGGGGCTTGTATGAA
TGATTGGACGAGGGACTGACTTTTTTTATTTAA-TTTTTA----
TGAATTTGATTTTTGAGTTAAAAAGCTTAAATTTTT-TTGCAAGACGAGAAGACCCTATAGAA--TTTTATT-
----------TTTAATTAATTACTTACTATT---TGGTTTATT---AAC-ATTAGGTAGTTTTTTAAGAA-
TTTTGTGGGGGCGATATTGAAA-TTAAAATAACTTTCATTTATAATTTT---TCATTATTTAATGTATT----
TTTTGATCCTTTA---TTAT--GGATT--ATTAGATTAAATTACCTTAGGGATAAC
Coptosoma_bifarium
ATGCAAGTCTCAGTACGAGCCGACT-
TAAAGTGAAACCGCAAATGGCTCAGTAAATCGGTCATAGTTTACTGGA-TATATG-CCACTC-
ACGTGGATAACTGTGGTAATTCTAGAACTAATACATGCAACC-AAGTCCCGACCAGCG---
ATGGAAAGGACGCTTTTATTGGATCAAAACCATTCGCCCGG-TCCGCCCGGGC------
GTCTGCTTGATGACCCT-AGATAAC-ACCACA-GATCGCACGGTCGCAGTACCGGT-
GACGTATCCGTCAAATGTCTGACTTATCAGCTGTCGATGGTAGACTCTGTGTCTACCATGGCACTCACGGGTA
ACGGGGAATCAGGGTTCGATTCCGGAGAGGGAGCCTGAGGAACGGCCACCACATCCAAGGAAGGCAGCAGGCA
CGCAAATTACCCACTCCCGGCACGGGGAGGTAGTGACAAAAAATAACGATACGGGACTCATCCGAGGCCCCGT
AATCGGAATGAGAACACCCTAAATCCTTTAACGAAGATCTATTGGAGGGCAA--
CTGGCCCCAGCAACCGCGGTAATTCCAGCTCCAATAGCGTATATTAAAGTTGTTGCGGTTAAAAAGCTCGTA
GTTGGTTCTGCGTGCCACGCTGTCGGTTCACCGCCTGTCGGTGCAACTGGCATGCC-
GTGGCATGTCCTGTCGGTGGCCGACAGGC-TGCGTGGCGGCGACCTATG-------CGTTAACAACGCGT---
-GGGTGGCCGC--GCCGTGGTGGTCC-CCGCTCGGCCGGCT-CAATCCTACCGCGGTGCT-
CGTTACCGAGTGTCGAGGTAGGCCGACACT-
GTTTACTTTGAACAAATTAGAGTGCTTAAAGCAGGCTCTGAAAGC-
AGCCTGAACAGTGGTGCATGGAATAATGGAAAAGGACCTCGGTTCTATTTTGTT-GGTTTTCTGA-
ATCCGAGGTAATGCTCAATAAGGA-CAGGCGGGGGCATTCGTATTGCGACGTTAGA-----------------
-GGTGAAATTCTTGG-
ATCGTCACAAGACGCACTAGAGCGAAAGCATTTGCCAAGTATGTCTTAATTGATCAAGAACTAAAGTTAGAGG
CTCGAAGGCGATCAGATACCGCCCTAGTTCTAACCATAAACGATGCCAGCCAGCGATCCGCCGATGTTCCTCC
GATGACTCGGCGGGGAGCTT-
TCCCCGGGAAACCAAAGCTTTTGGGTTCCGGGGGAAGTATGGTTGCAAAGCTGAAACTTAAAGGAATTGACGG
AAGGGCACCACCAGGAGTGGAGCCTGCGGCTTAATTTGACTCAACACGGGAAACCTCACCAGGCCCGGACATT
GGAA-GGATTGACAGAT-CGATAGCTCTTT-CTCGATT-CAGTGGGT-
AGTGGTGCATGGCCGTTCTTAGTTCGTGGACTGATTTGTCTGGTTAATTCCGATAACGAACGAGACTCTGACC
TATTAACTAGGCGACC-CCGGTATAA-ATA-CCTACCGGCGAGAT-
TTATTCTTCTTAAGGGGACAGGCGGCTCTCAGCCGCACGAGATTGAGCAATAACAGGTCTGTGATGCCCTTAG
ATGTTCTGGGCCGCACGCGCGCTACACTGAAGGAATCAGCGTGTTC--
TCCCCTGTCCGAAAGGTCCGGGCAACCCGCTGAACCTCCTTCGTGCTAGGAATTGGGGCTTGCAATTGTTCCC
CATGAACGAGGAATTCCCAGTAAGCGCGAGTCATAAGCTCGCGTTGATTACGTCCCTGCCCTTTGTACACACC
GCCCGTCGCTACTACCGATTGAATGATTTAGTGAGGTCTTCGGACC-GGCGGCG--AC-CGGC-ACT--
CGGG--CCACGCCCGG---TATCGGC-CCCGCTGGAAA-
GATGACCGAACTTGATCATTTAGAGGAAGTAAAAGTCGTAACAAGG-TTTCCAGTCA--CTGGGT--G-
AAAAACCTAAAGGCGCAATGAAAGT-GTCAG------------GTGGGGTCTCCTC------------CGGG-
-CG----GCCCCCCCGCTGGGAGGATGGGCTCTCC---GCACGTTTCTC-A------CGT--
CGGGGCCCCGCACTCCCGGGGCGTCCC--GTCCTCTCTCACG-AG----
CCGGGACGCACCAGGAGCGCACACGCTGGTACCCGAAAGATGGTGAACTATGCCTGGCCAGGACGAAGCCAGG
GGAAACCCTGGTGGAGGTCC--GAAGCG-ATTCTG--ACGTGCAAATCG-
ATCGTCTGAGCTGGGTATAGGGGCGAAAGACCAATCGAACCATCT----GTTA-TTT--
ATTTCAGGTTTGGCCTGCTCAATGATTTAATTAT--------
TAAATAGCCGCGGTATTTTGACCGTGCAAAGGTAGCATAATCAATTGTCTCTTAATTAGAGACTTGTATGAAT
GGTTTGATGAGAAACTAACTTTCTTTA---TTATAAGTTAA--
TGAACTTAATTTTTAGGTTAAAAAGCTTAAATGTTT-CTGTGGGACGAGAAGACCCTATAGAA--ATTTA-
CTATCCCTGT----------GACACTTATTTATATTT--ATTTAA----AATAAATGTTAGTAGTGAT---
AGTTTTGTTGGGGCGACATGGAGA-TTTTACAAACTCTCTACTTTTAATTT---TTATAAGTGTGTATAT---
--TATTGATCCTT-----TATTAAGGATA--TTGAGATTAATTTACCTTAGGGATAAC
Coptosoma_scutellatum
ATGCAAGTCTCAGTACGAGCCGACT-
TAAAGTGAAACCGCAAATGGCTCAGTAAATCGGTCATAGTTTACTGGA-TATATG-CCACTC-
ACGTGGATAACTGTGGTAATTCTAGAACTAATACATGCAACC-AAGTCCCGACCAGCG---

ATGGAAAGGACGCTTTTATTGGATCAAAACCATTCGCCCGG-TCCGCCCGGGC------
GTCTGCTTGATGACCCT-AGATAAC-ACCACA-GATCGCACGGTCGCAGTACCGGT-
GACGTATCCGTCAAATGTCTGACTTATCAGCTGTCGATGGTAGACTCTGTGTCTACCATGGCACTCACGGGTA
ACGGGGAATCAGGGTTCGATTCCGGAGAGGGAGCCTGAGGAACGGCCACCACATCCAAGGAAGGCAGCAGGCA
CGCAAATTACCCACTCCCGGCACGGGGAGGTAGTGACAAAAAATAACGATACGGGACTCATCCGAGGCCCCGT
AATCGGAATGAGAACACCCTAAATCCTTTAACGAAGATCTATTGGAGGGCAAGTCTGGCCCCAGCAACCGCGG
TAATTCCAGCTCCAATAGCGTATATTAAAGTTGTTGCGGTTAAAAAGCTCGTAGTTGGTTCTGCGTGCCACG
CTGTCGGTTCACCGCCTGTCGGTGCAACTGGCATGCC-GTGGCATGTCCTGTCGGTGGCCGACAGGC-
TGCGTGGCGGCGACCTATG-------CGTTAACAACGCGT----GGGTGGCCGC--GCCGTGGTGGTCC-
CCGCTCGGCCGGCT-CAATCCTACCGCGGTGCT-CGTTACCGAGTGTCGAGGTAGGCCGACACT-
GTTTACTTTGAACAAATTAGAGTGCTTAAAGCAGGCTCTGAAAGC-
AGCCTGAACAGTGGTGCATGGAATAATGGAAAAGGACCTCGGTTCTATTTTGTT-GGTTTTCTGA-
ATCCGAGGTAATGCTCAATAAGGA-CAGGCGGGGGCATTCGTATTGCGACGTTAGA-----------------
-GGTGAAATTCTTGG-
ATCGTCACAAGACGCACTAGAGCGAAAGCATTTGCCAAGTATGTCTTAATTGATCAAGAACTAAAGTTAGAGG
CTCGAAGGCGATCAGATACCGCCCTAGTTCTAACCATAAACGATGCCAGCCAGCGATCCGCCGATGTTCCTCC
GATGACTCGGCGGGGAGCTT-
TCCCCGGGAAACCAAAGCTTTTGGGTTCCGGGGGAAGTATGGTTGCAAAGCTGAAACTTAAAGGAATTGACGG
AAGGGCACCACCAGGAGTGGAGCCTGCGGCTTAATTTGACTCAACACGGGAAACCTCACCAGGCCCGGACATT
GGAA-GGATTGACAGAT-CGATAGCTCTTT-CTCGATT-CAGTGGGT-
AGTGGTGCATGGCCGTTCTTAGTTCGTGGACTGATTTGTCTGGTTAATTCCGATAACGAACGAGACTCTGACC
TATTAACTAGGCGACC-CCGGTATAA-ATA-CCTACCGGCGAGAT-
TTATTCTTCTTAAGGGGACAGGCGGCTCTCAGCCGCACGAGATTGAGCAATAACAGGTCTGTGATGCCCTTAG
ATGTTCTGGGCCGCACGCGCGCTACACTGAAGGAATCAGCGTGTTC--
TCCCCTGTCCGAAAGGTCCGGGCAACCCGCTGAACCTCCTTCGTGCTAGGAATTGGGGCTTGCAATTGTTCCC
CATGAACGAGGAATTCCCAGTAAGCGCGAGTCATAAGCTCGCGTTGATTACGTCCCTGCCCTTTGTACACACC
GCCCGTCGCTACTACCGATTGAATGATTTAGTGAGGTCTTCGGACC-GGCGGCG--AC-CGGC-ACT--
CGGG--CCACGCCCGG---TATCGGC-CCCGCTGGAAA-
GATGACCGAACTTGATCATTTAGAGGAAGTAAAAGTCGTAACAAGG-TTTCCAGTCA--TTGGGT--G-AAA-
-CCTAAAGGCGCAATGAAAGT-GTCAG------------GTGGGGTCTCCTC------------CGGG--CG-
---GCCCCCCCGCTGGGAGGATGGGCTCTCC---GCACGTTTCTG-A------CGT--
CGGGGCCCCGCACTCCCGGGGCGTCTC--GTCCTCTCTCACG-AG----
CCGAGACGCACCAGGAGCGCACACGCTGGTACCCGAAAGATGGTGAACTATGCCTGGCCAGGACGAAGCCAGG
GGAAACCCTGGTGGAGGTCC--GAAGCG-ATTCTG--ACGTGCAAATCG-
ATCGTCTGAGCTGGGTATAGGGGCGAAAGACCAATCGAACCATCT----GTTAATTT--
ATAGCAGGTCTAGCCTGCTCAATGATTAGAATATC---
TAGTTAAATAGCCGCAGTATTTTGACTGTGCAAAGGTAGCATAATCAATTGTCTCTTAATTAGAGGCTTGTAT
GAATGGTTTAATGAAAAATAAACTTTCTTT----TTAAAAATTGA--
TGAATTTAATTTTTAGGTTAAAAAGCTTAAATGGTT-TTGTGGGACGAGAAGACCCTATAGAA--ATTTA-
CTATTGGTGT----------AGTATTTATTGTTATTTTAATTTTA----GATTAATGCTTATTTCGAT---
AGTTTTGTTGGGGCGACATAGAGA-TTTTAAAAACTCTCTTTTTTTGTAAA---TTATAAATACATATAT---
--TATTGATCCTT-----TATTAAGGATA--TTAAGATTAATTTACCTTAGGGATAAC
Corythucha_ciliata
ATGCAAGTCTCAGTACAAACTTTTTATGAAGTGAAACCGCAAAAGGCTCAGTAAACCAGTTTTAATTTACTAG
AGTAAACG-AGACTG-ACGTGGATAACTGTGGTAATTCTAGAGCTAATACATGCAACA-
CAATCCTGACCATTC--
ACTGGGAAGGATGCTTTTATTGGACCAAGACCAATCGCTTGGGCTCTTCCGGGCTCAGGCGTTGACTTGATGA
CTCT-AGATAAC-ACTTCA-GATCGTATGGTCACCGAGCTGGC-
GACCTATCTTTCAAATGCCTGACATATCAACTGTCGATGGTAGACTCTATGTCTACCATGGCTCTTACGGGTA
ACAGGGAATCAGGGTTCGATTCCGGAGAGGGAGCCTGAGAAACGGCTACCACATCCAAGGAAGGCAGCAGGCA
CGCAAATTACCCACTCCCGGCACGGGGAGGTAGTGACAAAAAATAACGATGCGGGACTCTTTTGAGGCCCCGT
AATCGGAATGAGTACACTTTAAATCCTTTAACGATAATCAATTAGAGGGCAAGTCTGGTGCCAGCAGCCGCGG
TAATTCCAGCTCTAATAGCGTATATTAAAGTTGTTGCGGTTAAAAA-
GCTCGTAGTTGGTTCTGCGTGCCACGCTGTCGGTTCGCCGCCTGACGGTGTAACTGGCATGTC-
GTGGCATGTCCTGTCGGTGGCGAACTGGG-GCCACATTCGTTTTCCCCAT--------TCGGGCTTGTCT---
CGGGTGTGTGGA-GCGGTGTGGTCACTTGGTTCG-CCGGCT-CTATCCTGCCGCGGTGCT-
TTTAACCAAGTGTCGAGGTAGGCCGACAC--
GTTTACTTTGAACAAATTAGAGTGCTCAAAGCAGGCTTAAATCTC-

TGCCTGAATAGTGGTGCATGGAATAATGGAACAGGACCTTGGTTCTATTCTGTTGGT-CTCCGGA-
ACACAAGGTAATGATCAATGTGGA-CAGGCGGGGGCATTCGTATTGCGACGTTAGA-----------------
-GGTGAAATTCTTGG-
ATCGTCGCAAGACGCACTAGAGCGAAAGCATTTGCCAAGTATGTCTTAATTGATCAAGAACGAAAGTTAGAGG
TTCGAAGGCGATCAGATACCGCCCTAGTTCTAACCATAAACGATGCCAGCCAGTGATCCGCCGACGTTCCTCC
GATGACTCGGCGGGGAGCTTCTTCCCGGGAAACCAAAGCTTTTGGGTTCCGGGGGAAGTATGGTTGCAAAGCT
GAAACTTAAAGGAATTGACGGAAGGGCACCACCAGGAGTGGAGCCTGCGGCTTAATTTGACTCAACACGGGAA
ACCTCACCAGGTCAGGACATTGGAA-GGATTGACAGAT-TGATAGCTCTTT-CTTGATT-CAGTGGTT-
AGTGGTGCATGGCCGTTCTTAGTTGGTGGATCGATTTGTCTGGTTAATTCCGATAACGAACGAGACTCTAGCC
TATTAACTAGGCGTTT-CCGGTATCG---ATGCTACCGGCGATAC-
TTATTCTTCTTAAGGGGACAGGCGGCTTCCAGCCGCACGAAATTGAGCAATAACAGGTCTGTGATGCCCTTAG
ATGTCCTGGGCCGCACGCGCGCTACACTGAAAGAATCAGCGTGTGC-
CTCCCTGGTCCGATAGGGCCGGGCAACCCGCTGAACCTCTTTCGTGCTAGGAATTGGGGCTTGCAATTGTTCC
CCATGAACGAGGAATTCCCAGTAAGCGCGAGTCATAAGCTCGCGTTGATTACGTCCCTGCCCTTTGTACACAC
CGCCCGTCGCTACTACCGATTGAATGATTTAGTGAGGTCTTCGGACC-GACGGTAAGCGATGCCGCCT-----
-----CCATACGG---TATCGATGTACGTTGGGAA-
GATGACCAAACTTGATCATTTAGAGGAAGTAAAAGTCGTAACAAGG-TTTCCAGTCA--
TTGGGTGTTACAAAACCTAAAGGCGAAATGAAAGT-----------------AAAGAGAGTTTTTTGA-----
----
CTCTCAAGGGAGGATGAGCTACTGCTCTGGCTGTCCTAACCGGCGGCTGAGCGTGTCTCCGCACTCCCGGGGC
GATTC--TTTCTC-ATTGCG-AG----
TGGAGGCGCACCAAGAGCGAGCACGCTGAAACCCGAAAGATGGTGAACTATGCCTGGCCAGGACGAAGCCAGG
GGAAACCCTGGTGGAGGTCC--GCAGCG-ATTCTG--ACGTGCAAATCG-
ATCGTCGGAGCTGGGTATAGGGGCGAAAGACCAATCGAACCATCTG-------TTTTTA-
TAAAAAATATAACCTGCCCTATGATT---------AAAAGTTAAATGGCCGCGATTTT---
ATCGTGCAAAGGTAGCATAATAATTTGTCCTTTAAATGAGGTCTAGAATGAAAGGTTGAACGAGAGA-
GTAGCTTTCTTATTTTAATTTTATTG----AATTTTATTTTTTGGTTAAAAAGCTAAAATGAAT-
TTATGGGACGATAAGACCCTATAGAA--TTTAATTAACC--------------
TTATAAGATATAGTTTTTAT--TTTT---AATTTTATA-TCTTATAAGGT-
TAATTTAGTTGGGGCGACTATTATATTTGATATACTATATTTTTATA-TTTT---TCATTGTTTTATGAAAA-
-TTT---GATCCTG-----AATTTTAGATT--ATAAGAATAAATTACCTTAGGGATAAC
Cyclopelta_obscura
ATGCAAGTCTCAGTACGAGCTGACT-
TAAAGTGAAACCGCAAAAGGCTCAGTAAACCGGTCATAGTTTACCGGA-TATATG-CCACTC-
ACGTGGATAACTGTGGTAATTCTAGAGCTAATACATGACACC-AAGTCCCGACCAGCG---
ATGGAAAGGACGCTTTTATTGGATCAAAACCATTCGCCCGGCCCGCCC-GGGC------
GTCTGCTTGATGACTCT-AGATAAC-ACCACA-GATCGCACGGTCACTGTACCGGC-
GACGTATCCGTCAAATGTCTGACTTATCAGCTGTCGATGGTAGACTCTGTGTCTACCATGGCGCTCACGGGTA
ACGGGGAATCAGGGTTCGATTCCGGAGAGGGAGCCTGAGAAACGGCTACCACATCCAAGGAAGGCAGCAGGCA
CGCAAATTACCCACTCCCGGCACGGGGAGGTAGTGACAAAAAATAACGATACGGGACTCATCCGAGGCCCCGT
AATCGGAATGAGAACACCCTAAATCCTTTAACGAGGATCTATTGGAGGGCAAGTCTGGTGCCAGCAGCCGCGG
TAATTCCAGCTCCAATAGCGTATATTAAAGTTGTTGCGGTTAAAAA-
GCTCGTAGTTGGTTCTGCGTGCCACGCTGTCGGTTCACCGCCTGTCGGTGCAACTGGCATGCC-
GTGGCATGTCCTGTCGGTGGCTGACAGGC-TTCGCG-GCGCGCCCTATG-------CGTTTCTAGCGTGT---
-AGGTAGTTTC--GCCG-CGTCGTCC-TTGTAGG-CCGTTT-CAATCCTACCGCGGTGCT-
CTTTGCCGAGTGTCGAGGTAGGCCGACACT-
GTTTACTTTGAACAAATTAGAGTGCTTCAAGCAGGCTCTGAAAGC-
AGCCTGAATAGTGGTGCATGGAATAATGGAACAGGACCTTGGTTCTATTTTGTT-GGTTTCCGGA-
ATCCAAGGTAATGATCAATAAGGA-CAGGCGGGGGCATTCGTATTGCGACGTTAGA-----------------
-GGTGAAATTCTTGG-
ATCGTCGCAAGACGCACTAGAGCGAAAGCATTTGCCAAGTATGTCTTAATTGATCAAGAACGAAAGTTAGAGG
TTCGAAGGCGATCAGATACCGCCCTAGTTCTAACCATAAACGATGCCAGCCAGCGATCCGCCGATGTTCCTCC
GATGACTCGGCGGGGAGCTT-
TCCCCGGGAAACCAAAGCTTTTGGGTTCCGGGGGAAGTATGGTTGCAAAGCTGAAACTTAAAGGAATTGACGG
AAGGGCACCACCAGTAGTGGAGCCTGCGGCTTAATTTGACTCAACACGGGAAACCTCACCAGGCCCGGACATT
GGAA-GGATTGACAGAT-CGATAGCTCTTT-CTCGATT-CAGTGGGT-
AGTGGTGCATGGCCGTTCTTAGTTGGTGGACTGATTTGTCTGGTTAATTCCGATAACGAACGAGACTCTGACC
TATTAACTAGGCGCTC-CCGGTATAA-ATA-CCTACCGGCGAGAT-

TTATTCTTCTTAAGGGGACAGGCGGCTCTCAGCCGCACGAGATTGAGCAATAACAGGTCTGTGATGCCCTTAG
ATGTTCTGGGCCGCACGCGCGCTACACTGAAGGAATCAGCGTGTGC--
TCCCCTGTCCGAAAGGACCGGGCAACCCGCTGAACCTCCTTCGTGCTAGGAATTGGGGCTTGCAATTGTTCCC
CATGAACGAGGAATTTCCAGTAAGCGCGAGTCATAAGCTCGCGTTGATTACGTCCCTGCCCTTTGTACACACC
GCCCGTCGCTACTACCGATTGAATGATTTAGTGAGGTCTTCGGACC-GGCGGTA--AC-CGGC-ACC--GGC-
----TCGTTCGG---TATCGGT-TTCGCTGGAAA-
GATGACCGAACTTGATCATTTAGAGGAAGTAAAAGTCGTAACAAGG-TTTCCAGTCA--TTGGGTGT---
AAA-CCTAAAGGCGAAATGAAAGT-GTCTT---------------GGTGGGCCTC-------------AAGG-
-------GCTCACCGGAGGGAGGATGGGCACCC----GTACGGCTTGCT-------CGT--
CGGGGCCCCGCACTCCCGGGGCGTCTC--GTTCTC-ATGACG-AG----
TAGAGACGCACCAGGAGCGCACACGATGGTACCCGAAAGATGGTGAACTATGCCTGGCCAGGACGAAGCCAGG
GGAAACCCTGGTGGAGGTCC--GAAGCG-ATTCTG--ACGTGCAAATCG-
ATCGTCTGAGCTGGGTATAGGGGCGAAAGACCAATCGAACCATCTGTTAATTATTTT--
ATTTTAGGTCTGTCCTGCCCTATGAGT-------
TTATATCTTGAATGGCCGCGGTAATTTGACCGTGCGAAGGTAGCATAATCATTTGTCTATTAATTGTAGGCTT
GTATGAATGGTTTAATGTAATATTATCTTTCTTTGTTTTATAATTG-----
TGAACTTAATATTTTAGTGAAAAAGCTTAAATTTAT-TTGGAGGACGAGAAGACCCTATAGAA--TTTTATT-
----------TACTGTTTTTTAATCATTTTTGGTAAATCAAT------ATATGGTTTAATATT-
TAGTAAATTTTGTTGGGGCGACATGGAAA-TTTTTTTAACTTTCTGTATT-GATATTG-
TTATTGATTTATATA-----CTAAAGATCCTTTA---TT--ATGGATT--
ATTAGATTAAATTACCTTAGGGATAAC
Cydnus_aterrimus
ATGCAAGTCTCAGTACGAGCTGACT-
TAAAGTGAAACCGCAAAAGGCTCAGTAAACCGGTCATAGTTTACCGGA-TATATG-CCACTC-
ACGTGGATAACTGTGGTAATTCTAGAGCTAATACATGCAACC-AAGTCCCGACCAGCG---
ATGGAAAGGACGCTTTTATTGGATCAAAACCATTCGCCCGGCTCGTCC-GGGC------
GTCTGCTTGATGACTCT-AGATAAC-ACCACA-GATCGCACGGTCACAGTACCGGC-
GACGTATCCGTCAAATGTCTGACTTATCAGCTGTCGATGGTAGACTCTGTGTCTACCATGGCGCTCACGGGTA
ACGGGGAATCAGGGTTCGATTCCGGAGAGGGAGCCTGAGAAACGGCTACCACATCCAAGGAAGGCAGCAGGCA
CGCAAATTACCCACTCCCGGCACGGGGAGGTAGTGACAAAAAATAACGATACGGGACTCATCCGAGGCCCCGT
AATCGGAATGAGAACACCCTAAATCCTTTAACGAGGATCTATTGGAGGGCAAGTCTGGTGCCAGCAGCCGCGG
TAATTCCAGCTCCAATAGCGTATATTAAAGTTGTTGCGGTTAAAAA-
GCTCGTAGTTGGTTCTGCGTGCCACGCTGTCGGTTCACCGCCTGTCGGTGCAACTGGCATGCC-
GTGGCATGTCCTGTCGGTGGCTGACAGGC-TTCGCG-GCGCGCCCTATG-------CGTTCAC-ACGTGT---
-AGGTGATCTC--GCCG-CGTTGTCC-TTGTAGG-CCGGCT-CAATCCTACCGCGGTGCT-
CTTTGCCGAGTGTCGAGGTAGGCCGACACT-
GTTTACTTTGAACAAATTAGAGTGCTTCAAGCAGGCTCTGAAAGC-
AGCCTGAATAGTGGTGCATGGAATAATGGAACAGGACCTTGGTTCTATTTTGTT-GGTTTTCGGA-
ATCCAAGGTAATGATCAATAAGGAACAGGCGGGGGCATTCGTATTGCGACGTTAGA-----------------
-GGTGAAATTCTTGG-
ATCGTCGCAAGACGCACTAGAGCGAAAGCATTTGCCAAGTATGTCTTAATTGATCAAGAACGAAAGTTAGAGG
TTCGAAGGCGATCAGATACCGCCCTAGTTCTAACCATAAACGATGCCAGCCAGCGATCCGCCGATGTTCCTCC
GATGACTCGGCGGGGAGCTT-
TCCCCGGGAAACCAAAGCTTTTGGGTTCCGGGGGAAGTATGGTTGCAAAGCTGAAACTTAAAGGAATTGACGG
AAGGGCACCACCAGGAGTGGAGCCTGCGGCTTAATTTGACTCAACACGGGAAACCTCACCAGGCCCGGACATT
GGAA-GGATTGACAGAT-CGATAGCTCTTT-CTCGATT-CAGTGGGT-
AGTGGTGCATGGCCGTTCTTAGTTGGTGGACTGATTTGTCTGGTTAATTCCGATAACGAACGAGACTCTGACC
TATTAACTAGGCGCTC-CCGGTATAG-ATA-CCTACCGGCGAGAT-
TTATTCTTCTTAAGGGGACAGGCGGCTCTTAGCCGCACGAGATTGAGCAATAACAGGTCTGTGATGCCCTTAG
ATGTTCTGGGCCGCACGCGCGCTACACTGAAGGAATCAGCGTGTGC--
TCCCCTGTCCGAAAGGTCCGGGCAACCCGCTGAACCTCCTTCGTGCTAGGAATTGGGGCTTGCAATTGTTCCC
CATGAACGAGGAATTCCCAGTAAGCGCGAGTCATAAGCTCGCGTTGATTACGTCCCTGCCCTTTGTACACACC
GCCCGTCGCTACTACCGATTGAATGATTTAGTGAGGTCTTCGGACC-GGCGGTA--AC-CGGC-ACC--GGC-
----TCGTTCGG---TATCGGT-TTCGCTGGAAA-
GATGACCGAACTTGATCATTTAGAGGAAGTAAAAGTCGTAACAAGG-TTTCCAGTCA--TTGGGTGT---
AAA-CCTAAAGGCGCAATGAAAGT-GTCAG-------------GGGGCACTCCTT-------------
TAGGAGC-----GCTCCCCGCAGGGAGGATGGGCCACC----GTACAATTATT--------TGT--
CGGAGCCCCGCACTCCCGGGGCGTCTC--GTTCTC-ATTACG-AG----

TGGAGACGCACCAGGAGCGCACACGCTGGTACCCGAAAGATGGTGAACTATGCCTGGCCAGGACGAAGCCAGG
GGAAACCCTGGTGGAGGTCC--GAAGCG-ATTCTG--ACGTGCAAATCG-
ATCGTCTGAGCTGGGTATAGGGGCGAAAGACCAATCGAACCATCTGA-TAAT--TTT--
ATTTTAGGTCAGACCTGCTCAGTGATA-------
TATTAAATTAAATAGCCGCAGTATTTTGACTGTGCAAAGGTAGCATAATCATTTGTCTTTTAATTGTGGGCTT
GTATGAATGGTTTAATGAAATATAATCTTTCTTTGTGTTATATT--A---
AAGAACTTAATTTTTAAGTTAAAAAGCTTAAATATAA-GTGGGGGACGAGAAGACCCTATAGAA--
TTTTATAT------TGTTACTGTTATGG-----ATTAT----TTATTATG-----
TATTAATTTGTATTGGTAATAATATTTTGTTGGGGTGATAGGAAGA-TT-TGTTAAACTCTTTTAAT-
TATTAAT-TTATTTATTAGTATTT--TTAA---GATCCTTAA---TT--TTGGATT--
ATTAGATTAAATTACCTTAGGGATAAC
Cyrtomenus_emarginatus
ATGCAAGTCTCAGTACGAGCTGACT-
TAAAGTGAAACCGCAAAAGGCTCAGTAAACCGGTCATAGTTTACCGGA-TATATG-CCACTC-
ACGTGGATAACTGTGGTAATTCTAGAGCTAATACATGCAACC-AAGTCCCGACCAGCG---
ATGGAAAGGACGCTTTTATTGGATCAAAACCATTCGCCCGGCTCGCCC-GAGC------
GTTTGCTTGATGACTCT-AGATAAC-ACCACA-GATCGCACGGTCACAGTACCGGC-
GACGTATCCGTCAAATGTCTGACTTATCAGCTGTCGATGGTAGACTCTGTGTCTACCATGGCGCTCACGGGTA
ACGGGGAATCAGGGTTCGATTCCGGAGAGGGAGCCTGAGAAACGGCTACCACATCCAAGGAAGGCAGCAGGCA
CGCAAATTACCCACTCCCGGCACGGGGAGGTAGTGACAAAAAATAACGATACGGGACTCATCCGAGGCCCCGT
AATCGGAATGAGAACACCCTAAATCCTTTAACGAGGATCTATTGGAGGGCAAGTCTGGTGCCAGCAGCCGCGG
TAATTCCAGCTCCAATAGCGTATATTAAAGTTGTTGCGGTTAAAAA-
GCTCGTAGTTGGTTCTGCGTGCCACGCTGTCGGTTCACCGCCTGTCGGTGCAACTGGCATGCC-
GTGGCATGTCCTGTCGGTGGCTGAAAGGC-TTCGCG-GCGCGCCCTATG-------CGTTTAC-GCGTGT---
-AGGTCGTCGC--GCCGCCGTTGTCC-TTGTAGG-CCGGCT-CAATCCTACCGCGGTGCT-
CTTTGCCGAGTGTCGAGGTAGGCCGACACT-
GTTTACTTTGAACAAATTAGAGTGCTTCAAGCAGGCTCTGAAAGC-
AGCCTGAATAGTGGTGCATGGAATAATGGAACAGGACCTTGGTTCTATTTTGTT-GGTTTTCGGA-
ATCCAAGGTAATGATCAATAAGGA-CAGGCGGGGGCATTCGTATTGCGACGTTAGA-----------------
-GGTGAAATTCTTGG-
ATCGTCGCAAGACGCACTAGAGCGAAAGCATTTGCCAAGTATGTCTTAATTGATCAAGAACGAAAGTTAGAGG
TTCGAAGGCGATCAGATACCGCCCTAGTTCTAACCATAAACGATGCCAGCCAGCGATCCGCCGATGTTCCTCC
GATGACTCGGCGGGGAGCTT-
TCCCCGGGAAACCAAAGCTTTTGGGTTCCGGGGGAAGTATGGTTGCAAAGCTGAAACTTAAAGGAATTGACGG
AAGGGCACCACCAGGAGTGGAGCCTGCGGCTTAATTTGACTCAACACGGGAAACCTCACCAGGCCCGGACATT
GGAAAGGATTGACAGAT-CGATAGCTCTTT-CTCGATT-CAGTGGGT-
AGTGGTGCATGGCCGTTCTTAGTTGGTGGACTGATTTGTCTGGTTAATTCCGATAACGAACGAGACTCTGACC
TATTAACTAGGCGCTC-CCGGTATAA-ATA-CCTACCGGCGAGAT-
TTATTCTTCTTAAGGGGACAGGCGGCTCTTAGCCGCACGAGATTGAGCAATAACAGGTCTGTGATGCCCTTAG
ATGTTCTGGGCCGCACGCGCGCTACACTGAAGGAATCAGCGTGTGC--
TCCCCTGTCCGAAAGGACCGGGCAACCCGCTGAACCTCCTTCGTGCTAGGAATTGGGGCTTGCAATTGTTCCC
CATGAACGAGGAATTCCCAGTAAGCGCGAGTCATAAGCTCGCGTTGATTACGTCCCTGCCCTTTGTACACACC
GCCCGTCGCTACTACCGATTGAATGATTTAGTGAGGTCTTCGGACC-GGCGGTA--AC-CGGC-ACC--GGC-
----TCGTTCGG---TATCGGT-TTCGCTGGAAA-
GATGACCGAACTTGATCATTTAGAGGAAGTAAAAGTCGTAACAAGG-TTTCCAGTCA--TTGGGTGT---
AAA-CCTAAAGGCGCAATGAAAGT-GTCAG-------------GTTGGCCTCCTT-------------
TAGGAGA-----GCCTACCGCAGGGAGGATGGGCCACC----GTACAATTATT--------TGT--
CGGAGCCCCGCACTCCCGGGGCGTCTC--GTTCTC-ATTACG-AG----
TGGAGACGCACCAGGAGCGCACACGCTGGTACCCGAAAGATGGTGAACTATGCCTGGCCAGGACGAAGCCAGG
GGAAACCCTGGTGGAGGTCC--GAAGCG-ATTCTG--ACGTGCAAATCG-
ATCGTCTGAGCTGGGTATAGGGGCGAAAGACCAATCGAACCATCTGG-TTATATT-T--
ATTTCAGGTCTGGCCTGCTCAATGATTA----TTATTTAA-
TTAAATGGCCGCAGTACTTTGACTGTGCGAAGGTAGCATAATCATTTGTCTTTTAATTGTAGGCTTGTATGAA
TGGTTTGATGAAGTACTAACTTTCTTTTGATCATAAAAAA-----
GAACTTAATCTTTGAGTTAAAAAGCTTAAATTCTT-TCATAGGACGAGAAGACCCTATAGAA--TTTTATT--
-----------ATAGGGGTTAT-ATTTTATGTGAGGTTTTT----ATTAATAGGATA-
TATCTCTTATAATTTTGTTGGGGTGACAGGGAAA-TTGTATAAACTTTCTTTTTAT-TTGTTT-

TTATTTATTTATATG-----TTATTGATCCCTTG--ATT--AGGGATA--
TTAAGATTAAATTACCTTAGGGATAAC
Dalcantha_alata_dilatata
ATGCAAGTCTCAGTACGAGCTGACT-
TAAAGTGAAACCGCAAAAGGCTCAGTAAACCGGTCATAGTTTACCGGA-TATATG-CCACTC-
ACGTGGATAACTGTGGTAATTCTAGAGCTAATACATGCAACC-AAGTCCCGACCAGCG---
ATGGAAAGGACGCTTTTATTGGATCAAAACCATTCGCCCGGCTCGCCC-GGGC------
GTCTGCTTGATGACTCT-AGATAAC-ACCACA-GATCGCACGGTCACAGTACCGGC-
GACGTATCCGTCAAATGTCTGACTTATCAGCTGTCGATGGTAGACTCTGTGTCTACCATGGCTCTCACGGGTA
ACGGGGAATCAGGGTTCGATTCCGGAGAGGGAGCCTGAGAAACGGCTACCACATCCAAGGAAGGCAGCAGGCA
CGCAAATTACCCACTCCCGGCACGGGGAGGTAGTGACAAAAAATAACGATACGGGACTCATCCGAGGCCCCGT
AATCGGAATGAGAACACCCTAAATCCTTTAACGAGGATCTATTGGAGGGCAAGTCTGGTGCCAGCAGCCGCGG
TAATTCCAGCTCCAATAGCGTATATTAAAGTTGTTGCGGTTAAAAA-
GCTCGTAGTTGGTTCTGCGTGCCACGCTGTCGGTTCACCGCCTGTCGGTGCAACTGGCATGCC-
GTGGCATGTCCTGTCGGTGGCTGACAGGC-CTCACG-GCGCGCCCTGCG-------CGTTCAC-GCGTGC---
-AGGCTGCTGC--GCCG-CGTCGTCC-TTGTAGG-CCGGCT-CAATCCTACCGCGGTGCT-
CTTTGCCGAGTGTCGAGGTAGGCCGACACT-
GTTTACTTTGAACAAATTAGAGTGCTTCAAGCAGGCTCTTAAAGC-
AGCCTGAATAGTGGTGCATGGAATAATGGAACAGGACCTTGGTTCTATTTTGTT-GGTTTCCGGA-
ATCCAAGGTAATGATCAATAAGGA-CAGGCGGGGGCATTCGTATTGCGACGTTAGA-----------------
-GGTGAAATTCTTGG-
ATCGTCGCAAGACGCACTAGAGCGAAAGCATTTGCCAAGTATGTCTTAATTGATCAAGAACGAAAGTTAGAGG
TTCGAAGGCGATCAGATACCGCCCTAGTTCTAACCATAAACGATGCCAGCCAGCGATCCGCCGATGTTCCTCC
GATGACTCGGCGGGGAGCTT-
TCCCCGGGAAACCAAAGCTTTTGGGTTCCGGGGGAAGTATGGTTGCAAAGCTGAAACTTAAAGGAATTGACGG
AAGGGCACCACCAGGAGTGGAGCCTGCGGCTTAATTTGACTCAACACGGGAAACCTCACCAGGCCCGGACATT
GGAA-GGATTGACAGAT-CGATAGCTCTTC-CTCGATT-CAGTGGGT-
AGTGGTGCATGGCCGTTCTTAGTTGGTGGACTGATTTGTCTGGTTAATTCCGATAACGAACGAGACTCTGACC
TATTAACTAGGCGCTC-CCGGTATAA-ATA-CCTACCGGCGAGAT-
TTATTCTTCTTAAGGGGACAGGCGGCTCTCAGCCGCACGAGATTGAGCAATAACAGGTCTGTGATGCCCTTAG
ATGTTCTGGGCCGCACGCGCGCTACACTGAAGGAATCAGCGTGTGC--
TCCCCTGTCCGAAAGGTCCGGGCAACCCGCTGAACCTCCTTCGTGCTAGGAATTGGGGCTTGCAATTGTTCCC
CATGAACGAGGAATTCCCAGTAAGCGCGAGTCATAAGCTCGCGTTGATTACGTCCCTGCCCTTTGTACACACC
GCCCGTCGCTACTACCGATTGAATGATTTAGTGAGGTCTTCGGACC-GGCGGTA--AC-CGGC-ACC--GGC-
----TCGTTCGG---TATCGGT-TTCGCTGGAAA-
GATGACCGAACTTGATCATTTAGAGGAAGTAAAAGTCGTAACAAGG-TTTCCAGTCA--TTGGGTGC---
AAAACCTAAAGGCGCAATGAAAGT-GTCAG---------------GGGCTGCCTC-------------CAGG-
-------GCTCCCCGTAGGGAGGATGGGCACC-----GCGCGGCACGCT-------CGC--
CGGGGCCCCGCACTCCCGGGGCGTCTC--GTTCTC-GCCACG-AG----
TGGAGACGCACCAGGAGCGCACACGCTGGTACCCGAAAGATGGTGAACTATGCCTGGCCAGGACGAAGCCAGG
GGAAACCCTGGTGGAGGTCC--GAAGCG-ATTCTG--ACGTGCAAATCG-
ATCGTCTGAGCTGGGTATAGGGGCGAAAGACCAATCGAACCATCT---GATA--TTT--
ATATTAGGTCTAGCCTGCCCACTGATAA------
TTTAAATTTAAAGGGCCGCAGTATTTTGACTGTGCGAAGGTAGCATAATCATTTGTCTTTTAATTGGGGACTT
GTATGAATGGTTGGATGAGACATTGACTTTCTTTA--TTATAATTTA----
TGAATTTAATTTTTTAGTTAAAAAGCTAAAATGTTT-TTATAGGACGAGAAGACCCTATAGAA--TTTTATT-
---------TAATTTTGTAATATTTAAATTGG-ATAATTATTT-----ATTTA--TTACTTTTATAT-
AAATTTTGTTGGGGCGATGTAGAAA-TTTTATGAACTTTCTATTTT-TTT-TA--TTATGAATTAATATT---
--TTATTGATCCTTTT---TT--AAGGATA--ACAAGATTAAATTACCTTAGGGATAAC
Dicranocephalus_alticolus
ATGCAAGTCTCAGTACGAGCTGACT-
TAAAGTGAAACCGCAAAAGGCTCAGTAAACCGGTCATAGTTTACCGGA-TATATG-CCACTC-
ACGTGGATAACTGTGGTAATTCTAGAGCTAATACATGCAAAC-AAGTCCCGACCAGCG---
ATGGAGAGGACGCTTTTATTGGATCAAAACCATTCGCCCGGCTTGCCC-GGGC------
GTCGGCTTGATGATTCT-AGATAAC-ACCACA-GATCGCACGGTCACAGTACCGGT-
GACATATCCTTCAAATGTCTGACTTATCAGCTGTCGATGGTAGACTCTGTGTCTACCATGGCGCTCACGGGTG
ACGGGGAATCAGGGTTCGATTCCGGAGAGGGAGCCTGAGAAACGGCTACCACATCCAAGGAAGGCAGCAGGCA
CGCAAATTACCCACTCCCGGCACGGGGAGGTAGTGACAAAAAATAACGATACGGGACTCATCCGAGGCCCCGT

AATCGGAATGAGAACACCCTAAATCCTTTAACGAGGATCTATTGGAGGGCAAGTCTGGTGCCAGCAGCCGCGG
TAATTCCAGCTCCAATAGCGTATATTAAAGTTGTTGCGGTTAAAAA-
GCTCGTAGTTGGTTCTGCGTGCCACGCTGTCGGTTCACCGCCTGTCGGTGCAACTGGCATGCC-
GTGGCATGTCCTGTCGGTGGCGGATAGGT-TTCGCG-GCGAGCCCTATGCGG----TCT-CC----GTGT---
-AGGTCGCCTC--GCCGCGT-TGTCC-TTGTACG-CCGGCT-CAATCCTACCGCGGTGCT-
CTTTACCGAGTGTCGAGGTAGGCCGACACT-
GTTTACTTTGAACAAATTAGAGTGCTTAAAGCAGGCTCTTAAATC-
AGCCTGAATAGTGGTGCATGGAATAATGGAACAGGACCTTGGTTCTATTTTGTT-GGTTTTCGGA-
ATCCAAGGTAATGATCAATAAGGA-CAGGCGGGGGCATTCGTATTGCGACGTTAGA-----------------
-GGTGAAATTCTTGG-
ATCGTCGCAAGACGCACTAGAGCGAAAGCATTTGCCAAGTATGTCTTAATTGATCAAGAACGAAAGTTAGAGG
TTCGAAGGCGATCAGATACCGCCCTAGTTCTAACCATAAACGATGCCAGCCAGCGATCCGCCGACGTTCCTCC
GATGACTCGGCGGGGAGCTT-
TCCCCGGGAAACCAAAGCTTTTGGGTTCCGGGGGAAGTATGGTTGCAAAGCTGAAACTTAAAGGAATTGACGG
AAGGGCACCACAAGGAGTGGAGCCTGCGGCTTAATTTGACTCAACACGGGAAACCTCACCAGGCCCGGACATT
GGAA-GGATTGACAGAT-CGATAGCTCTTT-CTCGATT-CAGTGGGT-
AGTGGTGCATGGCCGTTCTTAGTTGGTGAACTGATTTGTCTGGTTAATTCCGATAACGAACGAGACTCTAACC
TATTAACTAGGCGTTG-CCGGTATAAA-AG-GCTACCGGCGAGAT-
TTATTCTTCTTAAGGGGACAGGCGGCTCTTAGCCGCACGAGATTGAGCAATAACAGGTCTGTGATGCCCTTAG
ATGTTCTGGGCCGCACGCGCGCTACACTGAAGGAATCAGTGTGTGC--
ATCCCTGTCCGAAAGGTCCGGGTAACCCGCTGAACCTCCTTCGTGCTAGGAATTGGGGCTTGCAATTGTTCCC
CATGAACGAGGAATTCCCAGTAAGCGCGAGTCATAAGCTCGCGTTGATTACGTCCCTGCCCTTTGTACACACC
GCCCGTCGCTACTACCGATTGAATGATTTAGTGAGGTCTTCGGACC-GGCGATA--AC-CGGC-ACC--
GATTT------TTCGG---TATCGAT-TTCGCTGGAAA-
GATGACCGAACTTGATCATTTAGAGGAAGTAAAAGTCGTAACAAGG-TTTCCAGTCA--TTGGGTGT---
AAAACCTAAAGGCGTAATGAAAGT-GTCAG---------------AGGGCGCTCC----------------
GCGC--------TCTCAGTGGGAGGATGGGCTC-CC---GGCTTTACGGC-------------
CGGCGCCCCGCACTCCCGGGGCGTTTC--GTTCTC-GATTCG-AG----
TAGAGACGCACCAGGAGCGCACACGCTGGTACCCGAAAGATGGTGAACTATGCCTGGCCAGGACGAAGCCAGG
GGAAACCCTGGTGGAGGTCC--GAAGCG-ATTCTG--ACGTGCAAATCG-
ATCGTCTGAGCTGGGTATAGGGGCGAAAGACCAATCGAACCATCTAG----ATAT-
TATATTTTAGGTCTGACCTGCCCTATGATTTTTA----------
TTAAATGGCTGCAGTATTTTAACTGTACAAAGGTAGCATAATCATTTGTCTTTTAATTGGGGGCTAGAATGAA
TGGTTGGACGAGGGATGTACTTTCTTTATTTAA-AATAAA----
TGAATTTAATTTTTTAGTCAAAAAGCTTAAATTTTT-TTGTTAGACGAGAAGACCCTATAGAA--
TTTTATTT----------TAT-TATTAATGCTTGTTATTAGGTTAAATTTG---A-
ATTTGTAGGAATTGGTAATAAAATTTTGTTGGGGTGACAGTGAAA-TTTAAATAACTTTCATTAATTTTATT-
--TCATTGATTAATGTTT-----TATTGATCCTATA---TTAT--GGATT--
ATAAGATTAAATTACCTTAGGGATAAC
Dismegistus_sanguineus
ATGCAAGTCTCAGTACGAGCTTACT-
TAAAGTGAAACCGCAAAAGGCTCAGTAAACCAAACATAGTTTACCGGA-TATATT-CCACTC-
AGGTGGATAACTGTGGTAATTCTAGAGCTAATACATGCAACT-AAGTCCCGACCAGTG---
ATGGAAAGGACGCTTTTATTGGATCAAAACCATTCGCCCGGTTC-GCCCGGGC------
GTCTGTTTGATGACTCT-AGATAAC-ACCACA-GATCGCACGGTCATTGTACCGGC-
GACGTATCCGTCAAATGTCTGACTTATCAGCTGTCGATGGTAGACTCTGTGTCTACCATGGCGCTCACGGGTA
ACGGGGAATCAGGGTTCGATTCCGGAGAGGGAGCCTGAGAAACGGCTACCACATCCAAGGAAGGCAGCAGGCA
CGCAAATTACCCACTCCCGGCACGGGGAGGTAGTGACAAAAAATAACGATACGGGACTCATCCGAGGCCCCGT
AATCGGAATGAGAACACCCTAAATCCTTTAACGAGGATCTATTGGAGGGCAAGTCTGGTGCCAGCAGCCGCGG
TAATTCCAGCTCCAATAGCGTATATTAAAGTTGTTGCGGTTAAAAA-
GCTCGTAGTTGGTTCTGCGTGCCACGCTGTCGGTTCACCGCCTGTCGGTGCAACTGGCATGCC-
GTGGCATGTACTGTCGGTGGCCTGCGGGC-TTCGCG-GCGCACCCTGTGCTCGC--CATG---TGCGGGC-G-
TAGGTCGCTGT--TCCGC-GTCGTCT-CCGTAGG-CCGGCT-ATATCCTACCGCGGTGCT-
CTTTACCGAGTGTCGAGGTAGGCCGACACT-
GTTTACTTTGAACAAATTAGAGTGCTTAAAGCAGGCTTTTAAATTGAGCCTGAATAGTGGTGCATGGAATAAT
GGAATAGGACCTTGGTTCTATTTTGTT-GGTTTTACGA-ATCCAAGGTAATGATCAATAAGGA-
CAGGCGGGGGCATTCGTATTGCGACGTTAGA------------------GGTGAAATTCTTGG-
ATCGTCGCAAGACGCACTAGAGCGAAAGCATTTGCCAAGTATGTCTTAATTGATCAAGAACGAAAGTTAGAGG

TTCGAAGGCGATCAGATACCGCCCTAGTTCTAACCATAAACGATGCCAGCCAGCGATCCGCCGATGTTCCTCC
GATGACTCGGCGGGGAGCTT-
TCCCCGGGAAACCAAAGCTTTTGGGTTCCGGGGGAAGTATGGTTGCAAAGCTGAAACTTAAAGGAATTGACGG
AAGGGCACCACCAGGAGTGGAGCCTGCGGCTTAATTTGACTCAACACGGGAAACCTCACCAGGCCCGGACATT
GGAA-GGATTGACAGAT-CGATAGCTCTTT-CTCGATT-CAGTGGGT-
AGTGGTGCATGGCCGTTCTTAGTTGGTGGACTGATTTGTCTGGTTAATTCCGATAACGAACGAGACTCTAACC
TGTTAACTAGGCGTTT-CCGGTATACAGA--CCTACCGGCGAATTATTATTCTTCTT---
GGGACAGGCGGCTCTCAGCCGCACGAGATTGAGCAATAACAGGTCTGTGATGCCCTTAGATGTTCTGGGCCGC
ACGCGCGCTACACTGAAGGAATCAGCGTGTTT--
TCCCCTGTCCGAAAGGACCGGGCAACCCGCTGAACCTCCTTCGTGCTAGGAATTGGGGCTTGCAATTGTTCCC
CATGAACGAGGAATTCCCAGTAAGCGCGAGTCATAAGCTCGCGTTGATTACGTCCCTGCCCTTTGTACACACC
GCCCGTCGCTACTACCGATTGAATGATTTAGTGAGGTCTTCGGACC-GGCGGTA--AC-AGGC-ACC--
GGCT-----CGCCCGG---TATCGGT-TCCGCTGGAAA-
GATGACCGAACTTGATCATTTAGAGGAAGTAAAAGTCGTAACAAGG-TTTCCAGTCA--TTGGGTTTT-
TGAAACCTAAAGGCGAAATGAAAGT-
GTCTTCTCGGAGAGGCGGTAGCCCCTTTTCGCTCTTCCTCGAGCGAAAAGACTCCCCTCTCCGTCGGGAAGAT
GGGCCGCC----GTACAATTATT--------TGT--CGGAGCCCCGCACTCCCGGGGCGTCTC--
GTTCTCCATTGCGGAG----
TGGAGACGCACCAGGAGCGCACACGCTGGTACCCGAAAGATGGTGAACTATGCCTGGCCAGGACGAAGCCAGG
GGAAACCCTGGTGGAGGTCC--GAAGCG-ATTCTG--ACGTGCAAATCG-
ATCGTCTGAGCTGGGTATAGGGGCGAAAGACCAATCGAACCATCTG--TGATATTTT--
ATTTTAAGTCAAACCTGCTCAATGATTT--AT--------
ATTAAATAGCCGCAGTATTTTGACTGTGCAAAGGTAGCATAATCATTTGTCTTTTAATTGTAGGCTGGAATGA
ATGGTTAGATGAAATATTAACTTTCTTTATTTTATA--TTGAA--
AGAATTTAATTTTTTAGTTAAAAAGCTAAAATATTT-CTGTAAGACGAGAAGACCCTATAGAA--
TTTTACTTAATA---TA--------TATTAGTTGCATCTTAAAT-ATTACC----ATGTG--TTAAACACA-
TATTAAGTCTCGTTGGGGTGATGGGGAAA-TTTTATTAACTTTCTTTATATATAAT---
TTACTAATTTGTATATA--T-TAAAGATCCTTA----TATTA-AGATA--
ATAAGATTAAATTACCTTAGGGATAAC
Dolycoris_penicillatus_baccarum
ATGCAAGTCTCAGTACGAGCTGACT-
TAAAGTGAAACCGCAAAAGGCTCAGTAAACCGGTCATAGTTTACCGGA-TATATG-CCACTC-
ACGTGGATAACTGTGGTAATTCTAGAGCTAATACATGCAACC-AAGTCCCGACCAGCG---
ATGGAAAGGACGCTTTTATTGGATCAAAACCATTCGCCC-GGCTCGCCCGGGC------
GTCTGCTTGATGACTCT-AGATAAC-ACCACA-GATCGCACGGTCACAGTACCGGC-
GACGTATCCGTCAAATGTCTGACTTATCAGCTGTCGATGGTAGACTCTGTGTCTACCATGGCGCTCACGGGTA
ACGGGGAATCAGGGTTCGATTCCGGAGAGGGAGCCTGAGAAACGGCTACCACATCCAAGGAAGGCAGCAGGCA
CGCAAATTACCCACTCCCGGCACGGGGAGGTAGTGACAAAAAATAACGATACGGGACTCATCCGAGGCCCCGT
AATCGGAATGAGAACACCCTAAATCCTTTAACGAGGATCTATTGGAGGGCAAGTCTGGTGCCAGCAGCCGCGG
TAATTCCAGCTCCAATAGCGTATATTAAAGTTGTTGCGGTTAAAAA-
GCTCGTAGTTGGTTCTGCGTGCCACGCTGTCGGTTCACCGCCTGTCGGTGCAACTGGCATGCC-
GTGGCATGTCCTGTCGGTGGCCGACGGGC-TTCGCG-GCGCGCCCCACT-------CGCTCACG-CGTGT---
-GGGTCGACGC--GCCGT-GTCGTCC-TCG-TTGGCCGGCT-CAATCCTACCGCGGTGCT-
CTTAAACGAGTGTCGAGGTAGGCCGACACT-
GTTTACTTTGAACAAATTAGAGTGCTTCAAGCAGGCTCTGAAAGC-
AGCCTGAATAGTGGTGCATGGAATAATGGAACAGGACCTTGGTTCTATTTTGTT-GGTTCCCGGA-
ATCCAAGGTAATGATCAATAAGGA-CAGGCGGGGGCATTCGTATTGCGACGTTAGA-----------------
-GGTGAAATTCTTGG-
ATCGTCGCAAGACGCACTAGAGCGAAAGCATTTGCCAAGTATGTCTTAATTGATCAAGAACGAAAGTTAGAGG
TTCGAAGGCGATCAGATACCGCCCTAGTTCTAACCATAAACGATGCCAGCCAGCGATCCGCCGATGTTCCTCC
GATGACTCGGCGGGGAGCTT-
TCCCCGGGAAACCAAAGCTTTTGGGTTCCGGGGGAAGTATGGTTGCAAAGCTGAAACTTAAAGGAATTGACGG
AAGGGCACCACCAGGAGTGGAGCCTGCGGCTTAATTTGACTCAACACGGGAAACCTCACCAGGCCCGGACATT
GGAA-GGATTGACAGAT-CGATAGCTCTTT-CTCGATT-CAGTGGGT-
AGTGGTGCATGGCCGTTCTTAGTTGGTGGACTGATTTGTCTGGTTAATTCCGATAACGAACGAGACTCTGACC
TATTAACTAGGCGCTC-CCGGTATAA-ATA-CCTACCGGCGACAA-
TTATTCTTCTTAAGGGGACAGGCGGCTCTCAGCCGCACGAGATTGAGCAATAACAGGTCTGTGATGCCCTTAG
ATGTTCTGGGCCGCACGCGCGCTACACTGAAGGAATCAGCGTGTTC--

TCCCCTGTCCGAAAGGTCCGGGCAACCCGCTGAACCTCCTTCGTGCTAGGAATTGGGGCTTGCAATTGTTCCC
CATGAACGAGGAATTCCCAGTAAGCGCGAGTCATAAGCTCGCGTTGATTACGTCCCTGCCCTTTGTACACACC
GCCCGTCGCTACTACCGATTGAATGATTTAGTGAGGTCTTCGGACC-GGCGGTA--AC-CGGC-ACC--GG--
---CTCGTTCGG---TATCGGT-TTCGCTGGA?A-
GATGACCGAACTTGATCATTTAGAGGAAGTAAAAGTCGTAACAAGG-TTTCCAGTCA--TTGGGT--G-
TAAAACCTAAAGGCGCAATGAAAGT-GTCA-------------GGGTTGCCTCCTT------------TAGG-
-AG----CGCTTCCCGTAGGGAGGATGGGC-CGCC---GTACGATCC-C-T------CGT--
CGGAGCCCCGCACTCCCGGGGCGTCTC--TTTCTCA-TCACG-AG----
CGGAGACGCACCAGGAGCGCACACGCTGGTACCCGAAAGATGGTGAACTATGCCTGGCCAGGACGAAGCCAGG
GGAAACCCTGGTGGAGGTCC--GAAGCG-ATTCTG--ACGTGCAAATCG-
ATCGTCTGAGCTGGGTATAGGGGCGAAAGACCAATCGAACCATCTG---ATTTAATT--
ATACTAAGTCTGGCCTGCTCAATGATTTT-------T----
TTAAATAGCCGCAGTATATTGACTGTGCAAAGGTAGCATAATCATTTGTCTTTTAATTGAAGGCTTGTATGAA
TGGTTGGATGAGGGAACGACTTTCTTTA---TATTAGGA-AT--
TGAATTTAATTTTTTAGTTAAAAAGCTTAAATTTAT-AAGTGGGACGAGAAGACCCTATAGAA--TTTTA-
TTTTAT-TGT----------AATAATTACTATTTTAGGGTTTAAT----TATTAAATGT----
TACAATAAAATTTTGTTGGGGTGATGGTGAGA-TTTTTATAACTCTCA-TAATTTATATTT-
TTATTAATTAGTATTT-----TTAAGATCCTA-----TTTTATGGATA--
ATTAGTTTAAATTACCTTAGGGATAAC
Dysdercus_cingulatus
ATGCAAGTCTCAGTACGAGCTAATC-
TAAAGTGAAACCGCAAAAGGCTCAGTAAACCGGTCATAGTTTACAGGT-TAAATG-
CCACTTTACGTGGATAACTGTGGTAATTCTAGAGCTAATACATGCAAACCAAGTCCCGACCAGAG---
ATGGAGAGGACGCTTTTATTGGATCAAAACCATTCGCCCGGCTC-GTCCGGGC------
GTCTGCTTGATGACTCT-AGATAAC-ATCACA-GATCGCACGGTCCTGGTACCGGC-
GACATATCCTTCAAATGTCTGACTTATCAGCTGTCGATGGTAGATTCTGTGTCTACCATGGCTCTCACGGGTG
ACGGGGAATCAGGGTTCGATTCCGGAGAGGGAGCCTGAGAAACGGCTACCACATCCAAGGAAGGCAGCAGGCA
CGCAAATTACCCACTCCCGGCACGGGGAGGTAGTGACAAAAAATAACGATACGGGACTCATCCGAGGCCCCGT
AATCGGAATGAGAACATCCTAAATCCTTTAACGAGGATCTATTGGAGGGCAAGTCTGGTGCCAGCAGCCGCGG
TAATTCCAGCTCCAATAGCGTATATTAAAGTTGTTGCGGTTAAAAA-
GCTCGTAGTTGGTTCTGCGTGCCACGCTGTCGGTTCACCGCCTGTCGGTGCAACTGGCATGCC-
GTGGCATGTCCTGTCGGTGGCGTGCAGGC-TTCGCG-GCG--CTCCCTGC------ATGATACGTTGTGT---
-AGGTCGT-------CGA-GTAGTCC-TCGTACG-CCGTCT-CAATCCTGCCGCGGTGCT-
CTTTACCGAGTGTCGAGGTAGGCCGGCACA-
TTTTACTTTGAACAAATTAGAGTGCTTAAAGCAGGCTCTTAAATC-
AGCCTGAATAGTGGTGCATGGAATAATGGAACAGGACCTTGGTTCTATTTTGTT-GGTTTAGGA-
ATCCAAGGTAATGATCAATAAGGA-CAGGCGGGGGCATTCGTATTGCGACGTTAGA-----------------
-GGTGAAATTCTTGG-
ATCGTCGCAAGACGCACTAGAGCGAAAGCATTTGCCAAGTATGTCTTAATTGATCAAGAACGAAAGTTAGAGG
TTCGAAGGCGATCAGATACCGCCCTAGTTCTAACCATAAACGATGCCAGCCAGCGATCCGCCGATGTTCTTTC
GATGACACGGCGGGGAGCTT-
CCCCCGGGAAACCAAAGCTTTTGGGTTCCGGGGGAAGTATGGTTGCAAAGCTGAAACTTAAAGGAATTGACGG
AAGGGCACCACCAGGAGTGGAGCCTGCGGCTTAATTTGACTCAACACGGGAAACCTCACCAGGCCCGGACATT
GGAA-GGATTGACAGAT-CGATAGCTCTTT-CTCGATT-CAGTGGGT-
AGTGGTGCATGGCCGTTCTTAGTTGGTGGACTGATTTGTCTGGTTAATTCCGATAACGAACGAGACTCTAACC
TATTAACTAGGCGATA-CTGGTATATATTA--CTACCAGCGAGAT-
TTATTCTTCTTAAGGGGACAGGCGGCTCTTAGCCGCACGAGAATGAGCAATAACAGGTCTGTGATGCCCTTAG
ATGTTCTGGGCCGCACGCGCGCTACACTGAAGGAATCAGCGTGTAC--
CCCCTTGACCGAAAGGTCCGGGTAACCCGTTGAACCTCCTTCGTGCTAGGAATTGGGGCTTGCAATTGTTCCC
CATGAACGAGGAATTCCCAGTAAGCGCGAGTCATAAGCTCGCGTTGATTACGTCCCTGCCCTTTGTACACACC
GCCCGTCGCTACTACCGATTGAATGATTTAGTGAGGTCTTCGGACC-GTCGATT--AT-TGGC-ACC--GAC-
----TCGTTCG---GTATCGAT-TTCGATGGAAA-
GATGACCGAACTTGATCATTTAGAGGAAGTAAAAGTCGTAACAAGG-TTTCCAGTCA--TTGGGTGA---
AAAACCTAAAGGCGAAATGAAAGT-GTCAG---------------GGCTCGCTCTAA----------------
------CGCGGCTTATCGGGAGGATGGG-----C---GTTCC-TAA--------------
CGGGCGCCCCGCACTCCCGGGGCGTCTC--ATTCTC-GTTTCG-AG----
TAGAGACGCACCAGGAGCGTACACGCTGGTACCCGAAAGATGGTGAACTATGCCTGGCCAGGACGAAGCCAGG
GGAAACCCTGGTGGAGGTCC--GAAGCG-ATTCTG--ACGTGCAAATCG-

ATCGTCTGAGCTGGGTATAGGGGCGAAAGACCAATCGAACCATCAGA----AT---
TTAATTTTAAGTCTGACCTGCTCAATGA-----------TTTATT-
AAATTGCTATAGTATTTTAACTATACAAAGGTAGCATAATAATTTGTCTTTTAATTGGAGGCTTGTATGAATG
GTTGGACGAGAAGTAAACTTTCTTAATTTAAATTAAAT----
TGAATTTAATATTTATGTAAAAAAGCTTAAATATAA-TTGTGGGACGAGAAGACCCTTTAGAA--TTTTAAT-
----
TGAATTAGTAATTTTTTATTTTAGAAATTGTTTGTAATATTTTATTAATTTAATTTTTGTTGGGGTGACAGTA
AAA-ATTTTTTAACTTTTATT-ATTAAATTAA-TCATAAATTAGTGTAT-----TATTGATCCTTTA----
TATAAAGATT--ATAAGATTAAATTACCTTAGGGATAAC
Elasmostethus_interstinctus
ATGCAAGTCTCAGTACGAGCTGACT-
TAAAGTGAAACCGCAAAAGGCTCAGTAAACCGGTCATAGTTTACCGGA-TATATG-CCACTC-
ACGTGGACAACTGTGGTAATTCTAGAGCTAATACATGCAACC-AAGTCCCGACCAGCG---
ATGGAAAGGACGCTTTTATTGGATCAAAACCATTCGCCCGGCCCCGCCCGGGC------GTTT--
TTGATGACTCTCAGATAAC-ACCACA-
GATCGCACGGTCATAGTACCGGCCGACGTATCCGTCAAATGTCTGACTTATCAGCTGTCGATGGTAGACTCTG
TGTCTACCATGGCGCTCACGGGTAACGGGGAATCAGGGTTCGATTCCGGAGAGGGAGCCTGAGAAACGGCTAC
CACATCCAAGGAAGGCAGCAGGCACGCAAATTACCCACTCCCGGCACGGGGAGGTAGTGACAAAAAAATAACGA
TACGGGACTCATCCGAGGCCCCGTAATCGGAATGAGAACACCCTAAATCCTTTAACGAGGATCTATTGGAGGG
CAAGTCTGGTGCCAGCAGCCGCGGTAATTCCAGCTCCAATAGCGTATATTAAAGTTGTTGCGGTTAAAAAAGC
TCGTAGTTGGTTCTGCGTGCCACGCTGTCGGTTCACCGCCTGTCGGTGCAACTGGCATGCC-
GTGGCATGTCCTGTCGGTGGCTTCACAGGCTTCGCG-GCGCGCCCTATG-------CGTT---AACACGT-G-
TAGGTAGTTGC--GCCGC-GTTGTTC-TTGTAGG-CCGGCT-CAATCCTGCCGCGGTGCT-
CTTTACCGAGTGTCGAGGTAGGCCGACACT-
GTTTACTTTGAACAAATTAGAGTGCTTCAAGCAGGCTCTGAAAGC-
AGCCTGAATAGTGGTGCATGGAATAATGGAACAGGACCTTGGTTCTATTTTGTT-GGTTTTAGGA-
ATCCAAGGTAATGATCAATAAGGA-CAGGCGGGGGCATTCGTATTGCGACGTTAGA-----------------
-GGTGAAATTCTTGG-
ATCGTCGCAAGACGCACTAGAGCGAAAGCATTTGCCAAGTATGTCTTAATTGATCAAGAACGAAAGTTAGAGG
TTCGAAGGCGATCAGATACCGCCCTAGTTCTAACCATAAACGATGCCAGCCAGCGATCCGCCGATGTTCCTCC
GATGACTCGGCGGGGAGCTT-
TCCCCGGGAAACCAAAGCTTTTGGGTTCCGGGGGAAGTATGGTTGCAAAGCTGAAACTTAAAGGAATTGACGG
AAGGGCACCACCAGGAGTGGAGCCTGCGGCTTAATTTGACTCAACACGGGAAACCTCACCAGGCCCGGACATT
GGAA-GGATTGACAGAT-CGATAGCTCTTT-CTCGATT-CAGTGGGT-
AGTGGTGCATGGCCGTTCTTAGTTGGTGGACTGATTTGTCTGGTTAATTCCGATAACGAACGAGACTCTGACC
TATTAACTAGGCGCTC-CCGGTATAC-ATA-CCTACCGGCGAGAT-
TTATTCTTCTTAAAGGGACAGGCGGCTCTTAGCCGCACGAGATTGAGCAATAACAGGTCTGTGATGCCCTTAG
ATGTTCTGGGCCGCACGCGCGCTACACTGAAGGAATCAGCGTGTGC--
TCCCCTGTCCGAAAGGACCGGGCAACCCGCTGAACCTCCTTCGTGCTAGGAATTGGGGCTTGCAATTGTTCCC
CATGAACGAGGAATTCCCAGTAAGCGCGAGTCATAAGCTCGCGTTGATTACGTCCCTGCCCTTTGTACACACC
GCCCGTCGCTACTACCGATTGAATGATTTAGTGAGGTCTTCGGACC-GGCGGTA--AC-AGGC-ACC--
GGCA-----CGTTCGG---TATCGGT-TCCGCTGGAAA-
GATGACCGAACTTGATCATTTAGAGGAAGTAAAAGTCGTAACAAGG-GTTTCAGTCA--TTGGGTTCT--
AAA-CCTAAAGGCGCAATGAAAGT-GTC-------------AGGGGGGCCTCCT-------------
AAGGGGGG-----TCTCCCCGCAGGGAGGATGGGCCACC----GCACACCTG-T--------TGT--
CGGAGCCCCGCACTCCCGGGGCGTCTC--GTCCTC-ATCACG-AG----
TAGAGACGCACCAGGAGCGCACACGCTGGTACCCGAAAGATGGTGAACTATGCCTGGCCAGGACGAAGCCAGG
GGAAACCCTGGTGGAGGTCC--GAAGCG-ATTCTG--ACGTGCAAATCG-
ATCGTATGAGCTGGGTATAGGGGCGAAAGACCAATCGAACCATCT----GTAAATTT--
ATATTAAGTCTTGCCTGCTCAATGATAATAT-------
TTATTAAATAGCCGCAGTATTTTGACTGTGCAAAGGTAGCATAATCATTTGTCTTTTAATTGTGGACTTGTAT
GAATGGTTTGATGAGATATTAGCTTTCTTTA---TATAAAATTGA--
TTAATTTAATTTCTTAGTTAAAAAGCTGAGATTATC-TTATAGGACGAGAAGACCCTATAGAA--
TTTTATGTATAT--AT---------TTATAAATTATATTTGGATATTTTTT----A-TTTTTATGGATTTA--
--TATATTTTGTTGGGGTGACAGGGAAA-TTTATACAACTTTCTTTATTTATATT---
TTATTAATTTATATAT-----TTATGATCCTT-----TTTTAAGGATA--
CTAAGATTAAATTACCTTAGGGATAAC
Elasmucha_grisea

ATGCAAGTCTCAGTACGAGCTGTCT-
TAAAGCGAAACCGCAAAAGGCTCAGTAAACCGGTCATAGTTTACCGGA-TATATG-TCACTC-
ACGTGGACAACTGTGGTAATTCTAGAGCTAATACATGCAACC-AAGTCCCGACCAGCG---
ATGGAAAGGACGCTTTTATTGGATCAAAACCATTCGCCCGGCCCCGCCCGGGC------
GTCTGCTTGATGACTCT-AGATAAC-ACCACA-GATCGCACGGTCATAGTACCGGC-
GACATATCCGTCAAATGTCTGACTTATCAGCTGTCGATGGTAGACTCTGTGCCTACCATGGCGCTCACGGGTA
ACGGGGAATCAGGGTTCGATTCCGGAGAGGGAGCCTGAGAAACGGCTACCACATCCAAGGAAGGCAGCAGGCA
CGCAAATTACCCACTCCCGGCACGGGGAGGTAGTGACAAAAAATAACGATACGGGACTCATCCGAGGCCCCGT
AATCGGAATGAGAACACCCTAAATCCTTTAACGAGGATCTATTGGAGGGCAAGTCTGGTGCCAGCAGCCGCGG
TAATTCCAGCTCCAATAGCGTATATTAAAGTTGTTGCGGTTAAAAA-
GCTCGTAGTTGGTTCTGCGTGCCACGCTGTCGGTTCACCGCCTGTCGGTGCAACTGGCGTGCC-
GTGGCATGTCCTGTCGGTGGCTTCACAGGCTTCGTG-GCGCGCCCTACG-------CGCT---AACACGT-G-
TAGGTAGTTGC--GCCGC-GTTGTTC-TTGTAGG-CCGGCT-CAATCCTGCCGCGGTGCT-
CTTTACCGAGTGTCGGGGTAGGCCGACACT-
GTTTACTTTGAACAAATTAGAGTGCTTCAAGCAGGCTCTGAAAAT-
AGCCTGAATAGTGGTGCATGGAATAATGGAACAGGACCTTGGTTCTATTTTGTT-GGTTTTAGGA-
ATCCAAGGTAATGATCAATAAGGA-CAGGCGGGGGCATTCGTATTGCGACGTTAGA-----------------
-GGTGAAATTCTTGG-
ATCGTCGCAAGACGCACTAGAGCGAAAGCATTTGCCAAGTATGTCTTAATTGATCAAGAACGAAAGTTAGAGG
TTCGAAGGCGATCAGATACCGCCCTAGTTCTAACCATAAACGATGCCAGCCAGCGATCCGCCGATGTTCCTCC
GATGACTCGGCGGGGAGCTT-
TCCCCGGGAAACCAAAGCTTTTGGGTTCCGGGGGAAGTATGGTTGCAAAGCTGAAACTTAAAGGAATTGACGG
AAGGGCACCACCAGGAGTGGAGCCTGCGGCTTAATTTGACTCAACACGGGAAACCTCACCAGGCCCGGACATT
GGAA-GGATTGACAGAT-CGATAGCTCTTT-CTCGATT-CAGTGGGT-
AGTGGTGCATGGCCGTTCTTAGTTGGTGGACTGATTTGTCTGGTTAATTCCGATAACGAACGAGACTCTGACC
TATTAACTAGGCGCTC-CCGGTATAC-ATA-CCTACCGGCGAGAT-
TTATTCTTCTTAAAGGGACAGGCGGCTCTTAGCCGCACGAGATTGAGCAATAACAGGTCTGTGATGCCCTTAG
ATGTTCTGGGCCGCACGCGCGCTACACTGAAGGAATCAGCGTGTGC--
TCCCCTGTCCGAAAGGACCGGGCAACCCGCTGAACCTCCTTCGTGCTAGGAATTGGGGCTTGCAATTGTTCCC
CATGAACGAGGAATTCCCAGTAAGCGCGAGTCATAAGCTCGCGTTGATTACGTCCCTGCCCTTTGTACACACC
GCCCGTCGCTACTACCGATTGAATGATTTAGTGAGGTCTTCGGACC-GGCGGTA--AC-CGGC-ACC--
GGCT-----CGTTCGG---TATCGGT-TTCGCTGGAAA-
GATGACCGAACTTGATCATTTAGAGGAAGTAAAAGTCGTAACAAGG-TTTCCAGTCA--TTGGGTTTT--
GAA-CCTAAAGGCGAAATGAAAGT-GTC-------------CGGTTGGCCTCCC-------------
GAGGGGGG-----CCTTCCCACGGGGAGGATGGGTTTCC----GCACAGTTA-C--------TGT--
CGGAGCCCCGCACTCCCGGGGCGTCTC--GTTCTC-ATCACG-AG----
TAGAGACGCACCAGGAGCGCACACGCTGGTACCCGAAAGATGGTGAACTATGCCTGGCCAGGACGAAGCCAGG
GGAAACCCTGGTGGAGGTCC--GAAGCG-ATTCTG--ACGTGCAAATCG-
ATCGTATGAGCTGGGTATAGGGGCGAAAGACCAATCGAACCATCT----GTGTATTT--
ATATTAAGTCATACCTGCTCAATGATATTTAA------
TTATTAAATGGCCGCAGTATTTTGACTGTGCAAAGGTAGCATAATCATTTGTCTTTTAATTGTGGACTTGTAT
GAATGGTTTGATGAAATATTGACTTTCTTTA---TATAAAAAATA--
TTAATTTAATATTTTAGTTAAAAAGCTAAAATTGAT-TTATAGGACGAGAAGACCCTATAGAA--
TTTTATACTTTTATTA---------AAACAAATAATAATTGGTTTATT-TA----T-ATTTTGTTTTA-TGT-
--AGTATTTTGTTGGGGTGACAGGAAAA-TTTTTATAACTTTTCTTATTTATTAT---
TTATTTATTTGTATTT-----TAATGATCCTA-----TTTTAAGGATA--
TTAAGATTAAATTACCTTAGGGATAAC
Elasmucha_laeviventris
ATGCAAGTCTCAGTACGAGCTGACT-
TAAAGTGAAACCGCAAAAGGCTCAGTAAACCGGTCATAGTTTACCGGA-TATATG-TCACTC-
ACGTGGACAACTGTGGTAATTCTAGAGCTAATACATGCAACC-AAGTCCCGACCAGCG---
ATGGAAAGGACGCTTTTATTGGATCAAAACCATTCGCCCGGCCCCGCCCGGGC------
GTCTGCTTGATGACTCT-AGATAAC-ACCACA-GATCGCACGGTCATAGTACCGGC-
GACATATCCGTCAAATGTCTGACTTATCAGCTGTCGATGGTAGACTCTGTGTCTACCATGGCGCTCACGGGTA
ACAGGGAATCAGGGTTCGATTCCGGAGAGGGAGCCTGAGAAACGGCTACCACATCCAAGGAAGGCAGCAGGCA
CGCAAATTACCCACTCCCGGCACGGGGAGGTAGTGACAAAAAATAACGATACGGGACTCATCCGAGGCCCCGT
AATCGGAATGAGAACACCCTAAATCCTTTAACGAGGATCTATTGGAGGGCAAGTCTGGTGCCAGCAGCCGCGG
TAATTCCAGCTCCAATAGCGTATATTAAAGTTGTTGCGGTTAAAAA-

GCTCGTAGTTGGTTCTGCGTGCCACGCTGTCGGTTCACCGCCTGTCGGTGCAACTGGCATGCC-
GTGGCATGTCCTGTCGGTGGCTTCACAGGCTTCGTG-GCGCGCCCTACG-------CGCT---AACACGT-G-
TAGGTAGTTGC--GCCGC-GTTGTTC-TTGTAGG-CCGGCT-CAATCCTGCCGCGGTGCT-
CTTTACCGAGTGTCGGGGTAGGCCGACACT-
GTTTACTTTGAACAAATTAGAGTGCTTCAAGCAGGCTCTGAAAAT-
AGCCTGAATAGTGGTGCATGGAATAATGGAACAGGACCTTGGTTCTATTTTGTT-GGTTTTAGGA-
ATCCAAGGTAATGATCAATAAGGA-CAGGCGGGGGCATTCGTATTGCGACGTTAGA-----------------
-GGTGAAATTCTTGG-
ATCGTCGCAAGACGCACTAGAGCGAAAGCATTTGCCAAGTATGTCTTAATTGATCAAGAACGAAAGTTAGAGG
TTCGAAGGCGATCAGATACCGCCCTAGTTCTAACCATAAACGATGCCAGCCAGCGATCCGCCGATGTTCCTCC
GATGACTCGGCGGGGAGCTT-
TCCCCGGGAAACCAAAGCTTTTGGGTTCCGGGGGAAGTATGGTTGCAAAGCTGAAACTTAAAGGAATTGACGG
AAGGGCACCACCAGGAGTGGAGCCTGCGGCTTAATTTGACTCAACACGGGAAACCTCACCAGGCCCGGACATT
GGAA-GGATTGACAGAT-CGATAGCTCTTT-CTCGATT-CAGTGGGT-
AGTGGTGCATGGCCGTTCTTAGTTGGTGGACTGATTTGTCTGGTTAATTCCGATAACGAACGAGACTCTGACC
TATTAACTAGGCGCTC-CCGGTATAC-ATA-CCTACCGGCGAGAT-
TTATTCTTCTTAAAGGGACAGGCGGCTCTTAGCCGCACGAGATTGAGCAATAACAGGTCTGTGATGCCCTTAG
ATGTTCTGGGCCGCACGCGCGCTACACTGAAGGAATCAGCGTGTGC--
TCCCCTGTCCGAAAGGACCGGGCAACCCGCTGAACCTCCTTCGTGCTAGGAATTGGGGCTTGCAATTGTTCCC
CATGAACGAGGAATTCCCAGTAAGCGCGAGTCATAAGCTCGCGTTGATTACGTCCCTGCCCTTTGTACACACC
GCCCGTCGCTACTACCGATTGAATGATTTAGTGAGGTCTTCGGACC-GGCGGTA--AC-CGGC-ACC--
GGCT-----CGTTCGG---TATCGGT-TCCGCTGGAAA-
GATGACCGAACTTGATCATTTAGAGGAAGTAAAAGTCGTAACAAGG-TTTCCAGTCA--TTGGGTTCT--
GAAACCTAAAGGCGAAATGAAAGT-GTC-------------CGGTTGGCCTCCC-------------
GAGGGGGG-----CCTTCCCACGGGGAGGATGGGTTTCC----GCACAGTTA-C--------TGT--
CGGAGCCCCGCACTCCCGGGGCGTCTC--GTTCTC-ATCACG-AG----
TAGAGACGCACCAGGAGCGCACACGCTGGTACCCGAAAGATGGTGAACTATGCCTGGCCAGGACGAAGCCAGG
GGAAACCCTGGTGGAGGTCC--GAAGCG-ATTCTG--ACGTGCAAATCG-
ATCGTATGAGCTGGGTATAGGGGCGAAAGACCAATCGAACCATCT----GTATATTT--
ATATTAAGTCATACCTGCTCAATGATAATTAA------
TTATTAAATGGCCGCAGTATTTTGACTGTGCAAAGGTAGCATAATCATTTGTCTTTTAATTGTGGACTTGTAT
GAATGGTTTGATGAAATATTGACTTTCTTTA---TATAAAAAATG--
TTAACTTAATATTTTAGTTAAAAAGCTAAAATTATT-TTATAGGACGAGAAGACCCTATAGAA--
TTTTATATTTTATATA---------TAGTAATTAATATTTGGTTGATTATT----T-ATTTTATTATAATAA-
--AATATTTTGTTGGGGTGACAGGAAAA-TATCTAAAACTTTTTTTATTTATTTA---
TTATTTATTTATATTT-----TTATGATCCTA-----TTTTAAGGATA--
TTAAGATTAAATTACCTTAGGGATAAC
Erthesina_fullo
ATGCAAGTCTCAGTACGAGCTGACT-
TAAAGTGAAACCGCAAAAGGCTCAGTAAACCGGTCATAGTTTACCGGA-TATATG-CCACTC-
ACGTGGATAACTGTGGTAATTCTAGAGCTAATACATGCAACC-AAGTCCCGACCAGCG---
ATGGAAAGGACGCTTTTATTGGATCAAAACCATTCGCCCGGCT-CGCCCGGGC------
GTCTGCTTGATGACTCT-AGATAAC-ACCACA-GATCGCACGGTCACAGTACCGGC-
GACGTATCCGTCAAATGTCTGACTTATCAGCTGTCGATGGTAGACTCTGTGTCTACCATGGCGCTCACGGGTA
ACGGGGAATCAGGGTTCGATTCCGGAGAGGGAGCCTGAGAAACGGCTACCACATCCAAGGAAGGCAGCAGGCA
CGCAAATTACCCACTCCCGGCACGGGGAGGTAGTGACAAAAAATAACGATACGGGACTCATCCGAGGCCCCGT
AATCGGAATGAGAACACCCTAAATCCTTTAACGAGGATCTATTGGAGGGCAAGTCTGGTGCCAGCAGCCGCGG
TAATTCCAGCTCCAATAGCGTATATTAAAGTTGTTGCGGTTAAAAA-
GCTCGTAGTTGGTTCTGCGTGCCACGCTGTCGGTTCACCGCCTGTCGGTGCAACTGGCATGCC-
GTGGCATGTCCTGTCGGTGGCCGACGGGC-TTCGCG-GCGCGCCCCACT-------TGCTCAGG-CGTGT---
-GGGTCGACGC--GCCGT-GTCGTCC-TCG-TTGGCCGGCT-CAATCCTACCGCGGTGCT-
CTTAAACGAGTGTCGAGGTAGGCCGACACT-
GTTTACTTTGAACAAATTAGAGTGCTTAAAGCAGGCTAATAAAGG-
AGCCTGAATAGTGGTGCATGGAATAATGGAACAGGACCTTGGTTCTATTTTGTT-GGTTTACGGA-
ATCCAAGGTAATGATCAATAAGGA-CAGGCGGGGGCATTCGTATTGCGACGTTAGA-----------------
-GGTGAAATTCTTGG-
ATCGTCGCAAGACGCACTAGAGCGAAAGCATTTGCCAAGTATGTCTTAATTGATCAAGAACGAAAGTTAGAGG
TTCGAAGGCGATCAGATACCGCCCTAGTTCTAACCATAAACGATGCCAGCCAGCGATCCGCCGATGTTCCTCC

GATGACTCGGCGGGGAGCTT-
TCCCCGGGAAACCAAAGCTTTTGGGTTCCGGGGGAAGTATGGTTGCAAAGCTGAAACTTAAAGGAATTGACGG
AAGGGCACCACCAGGAGTGGAGCCTGCGGCTTAATTTGACTCAACACGGGAAACCTCACCAGGCCCGGACATT
GGAA-GGATTGACAGAT-CGATAGCTCTTT-CTCGATT-CAGTGGGT-
AGTGGTGCATGGCCGTTCTTAGTTGGTGGACTGATTTGTCTGGTTAATTCCGATAACGAACGAGACTCTGACC
TATTAACTAGGCGCTC-CCGGTATAA-ATA-CCTACCGGCGACAA-
TTATTCTTCTTAAGGGGACAGGCGGCTCTCAGCCGCACGAGATTGAGCAATAACAGGTCTGTGATGCCCTTAG
ATGTTCTGGGCCGCACGCGCGCTACACTGAAGGAATCAGCGTGTTC--
TCCCCTGTCCGAAAGGTCCGGGCAACCCGCTGAACCTCCTTCGTGCTAGGAATTGGGGCTTGCAATTGTTCCC
CATGAACGAGGAATTCCCAGTAAGCGCGAGTCATAAGCTCGCGTTGATTACGTCCCTGCCCTTTGTACACACC
GCCCGTCGCTACTACCGATTGAATGATTTAGTGAGGTCTTCGGACC-GGCGGTA--AC-CGGC-ACC--GG--
---CTCGCTCGG---TATCGGT-TTCGCTGGAAA-
GATGACCGAACTTGATCATTTAGAGGAAGTAAAAGTCGTAACAAGG-TTTCCAGTCA--TTGGGT--G-
GAAAACCTAAAGGCGCAATGAAAGT-GTCCC------------GGGGCGCATCCCT------------
CAGGGGAG----CGCGTCCCGTCGGGAGGATGGGC-CGCC---GCCCGGCTGAG-C------
CGTGGCGGAGCCCCGCACTCCCGGGGCGTCTC--GTTCTCA-TAGCG-AG----
TGGAGACGCACCAGGAGCGCACACGCTGGTACCCGAAAGATGGTGAACTATGCCTGGCCAGGACGAAGCCAGG
GGAAACCCTGGTGGAGGTCC--GAAGCG-ATTCTG--ACGTGCAAATCG-
ATCGTCTGAGCTGGGTATAGCGGCGAAAGACCAATCGAACCATCTG---TTATATT----
TATTAAGTCTGGCCTGCTCAATGATTAT---------
AAATTAAATAGCCGCGGTATTTTGACTGTGCAAAGGTAGCATAATCATTTGTCTTTTAATTGAAGGCTTGAAT
GAATGGTTTGATGAGAAAATGACTTTCTTTA---CATTATGG-AT--
TGAATTTAATGTTTTAGTTAAAAAGCTAAAATATAA-AAGTGGGACGAGAAGACCCTATAGAA--TTTTA--
TTTCTTATT----------GATTATT---AATTTTTTGGTTAAT----
ATATTATTCTTATTTAATAGGGAATTTTGTTGGGGTGACAGTGGGA-
TTTTTATAACTCCTATAATCAAATAT---TTACATATCAGTATTT-----TAAAGATCCTT-----TTTT-
TTGATA--ATTAGATTAAATTACCTTAGGGATAAC
Eurydema_maracandica
ATGCAAGTCTCAGTACGAGCTGACT-
TAAAGTGAAACCGCAAAAGGCTCAGTAAACCGGTCATAGTTTACCGGA-TATATG-CCACTC-
ACGTGGATAACTGTGGTAATTCTAGAGCTAATACATGCAACC-AAGTCCCGACCAGCG---
ATGGAAAGGACGCTTTTATTGGATCAAAACCATTCGCCCGGCT-CGCCCGGGC------
GTCTGCTTGATGACTCT-AGATAAC-ACCGCA-GATCGCACGGTCACAGTACCGGC-
GACGTATCCGTCAAATGTCTGACTTATCAGCTGTCGATGGTAGACTCTGTGTCTACCATGGCGCTCACGGGTA
ACGGGGAATCAGGGTTCGATTCCGGAGAGGGAGCCTGAGAAACGGCTACCACATCCAAGGAAGGCAGCAGGCA
CGCAAATTACCCACTCCCGGCACGGGGAGGTAGTGACAAAAAATAACGATACGGGACTCATCCGAGGCCCCGT
AATCGGAATGAGAACACCCTAAATCCTTTAACGAGGATCTATTGGAGGGCAAGTCTGGTGCCAGCAGCCGCGG
TAATTCCAGCTCCAATAGCGTATATTAAAGTTGTTGCGGTTAAAAA-
GCTCGTAGTTGGTTCTGCGTGCCACGCTGTCGGTTCACCGCCTGTCGGTGCAACTGGCATGCC-
GTGGCATGTCCTGTCGGTGGCCGACGGGC-TCCGCG-GCGCGCCCCACT-------CGCTCACG-CGTGT---
-GGGTCGACGC--GCCGT-GTTGTCC-TCG-TTGGCCGGCT-CAATCCTACCGCGGTGCT-
CTTAAACGAGTGTCGAGGTAGGCCGACACT-
GTTTACTTTGAACAAATTAGAGTGCTTCAAGCAGGCTCTGAAAGC-
AGCCTGAATAGTGGTGCATGGAATAATGGAACAGGACCTTGGTTCTATTTTGTT-GGTTTCCGGA-
ATCCAAGGTAATGATCAATAAGGA-CAGGCGGGGGCATTCGTATTGCGACGTTAGA-----------------
-GGTGAAATTCTTGG-
ATCGTCGCAAGACGCACTAGAGCGAAAGCATTTACCAAGTATGTCTTAATTGATCAAGAACGAAAGTTAGAGG
TTCGAAGGCGATCAGATACCGCCCTAGTTCTAACCATAAACGATGCCAGCCAGCGATCCGCCGATGTTCCTCC
GATGACTCGGCGGGGAGCTT-
TCCCCGGGAAACCAAAGCTTTTGGGTTCCGGGGGAAGTATGGTTGCAAAGCTGAAACTTAAAGGAATTGACGG
AAGGGCACCACCAGGAGTGGAGCCTGCGGCTTAATTTGACTCAACACGGGAAACCTCACCAGGCCCGGACATT
GGAA-GGATTGACAGAT-CGATAGCTCTTT-CTCGATT-CAGTGGGT-
AGTGGTGCATGGCCGTTCTTAGTTGGTGGACTGATTTGTCTGGTTAATTCCGATAACGAACGAGACTCTGACC
TATTAACTAGGCGCTC-CCGGTATAA-ATA-CCTACCGGCGACAA-
TTATTCTTCTTAAGGGGACAGGCGGCTCTCAGCCGCACGAGATTGAGCAATAACAGGTCTGTGATGCCCTTAG
ATGTTCTGGGCCGCACGCGCGCTACACTGAAGGAATCAGCGTGTTC--
TCCCCTGTCCGAAAGGTCCGGGCAACCCGCTGAACCTCCTTCGTGCTAGGAATTGGGGCTTGCAATTGTTCCC
CATGAACGAGGAATTCCCAGTAAGCGCGAGTCATAAGCTCGCGTTGATTACGTCCCTGCCCTTTGCACACACC

GCCCGTCGCTACTACCGATTGAATGATTTAGTGAGGTCTTCGGACC-GGCGGTA--AC-AGGC-ACC--GG--
---CTCGTCCGG---TATCGGT-TTCGCTGGAAA-
GATGACCGAACTTGATCATTTAGAGGAAGTAAAAGTCGTAACAAGG-TTTCCAGTCA--TTGGGT--G-
TAAAACCTAAAGGCGCAATGAAAGT-GTCA-------------GGGGTACCTCCTT------------TAGG-
-GG----CGTATCCCGCTGGGAGGATGGGC-CCCC---GTACGATAT-C-T------CGT--
CGGAGCCCCGCACTCCCGGGGCGTCTC--GTTCTCG-TTACG-AG----
TGGAGACGCACCAGGAGCGCACACGCTGGTACCCGAAAGATGGTGAACTATGCCTGGCCAGGACGAAGCCAGG
GGAAACCCTGGTGGAGGTCC--GAAGCG-GTTCTG--ACGTGCAAATCG-
ATCGTCTGAGCTGGGTATAGGGGCGAAAGACCAATCGAACCATCTG---TTATAATT--
ATATTAAGTCTGGCCTGCTCAATGATTTT---------
ATATTAAATAGCCGCAGTATTTTGACTGTGCGAAGGTAGCATAATCATTTGTCTTTTAATTGAAGGCTTGTAT
GAATGGTTGGATGAGGGAATAGCTTTCTTTA---TATTAAAT-AT--
TGAATTTAATTTTTTAGTTAAAAAGCTTAAATATTT-AAATGGGACGAGAAGACCCTATAGAA--
TTTTAATATTTAAATT----------ATTATTTAATAATTTTGGGTAATAC----
TTTTATTAATAATTTAAT----ATTTTTATTGGGGTGATAGTGAGA-
TTTAATTAACTCTCATAATATAATTT---TTATTAATTAATATAT-----TTAAGATCCTA-----
TATTAAGGATA--GTTAGATTAAATTACCTTAGGGATAAC
Eurygaster_maura
ATGCAAGTCTCAGTACGAGCTGACT-
TAAAGTGAAACCGCAAAAGGCTCAGTAAACCGGTCATAGTTTACCGGA-TATATG-CCACTC-
AGGTGGATAACTGTGGTAATTCTAGAGCTAATACATGCAACC-AAGTCCCGACCAGCG---
ATGGGAAGGACGCTTTTTTTGGATCAAAACCATTCGCCCGGCT-CGCCCGGGC------
GTCTGCTTGATGACTCT-AGATAAC-ACCACA-GATCGCACGGTCACAGCACCGGC-
GACGTATCCGTCAAATGTCTGACTTATCAGCTGTCGATGGTAGACTCTGTGTCTACCATGGCGCTCACGGGTG
ACGGGGAATCAGGGTTCGATTCCGGAGAGGGAGCCTGAGAAACGGCTACCACATAAAAAGAAGGCAGCAGGCA
CGCAAATTACCCACTCCCGGCACGGGGAGGTAGTGACAAAAAATAACGATACGGGACTCATCCGAGGCCCCGT
AATCGGAATGAGAACACCCTAAATCCTTTAACGAGGATCTATTGGAGGGCAAGTCTGGTGCCAGCAGCCGCGG
TAATTCCAGCTCCAATAGCGTATATTAAAGTTGTTGCGGTTAAAAA-
GCTCGTAGTTGGTTCTGCGTGCCACGCTGTCGGTTCACCGCCTGTCGGTGCAACTGGCATGCC-
GTGGCATGTCCTGTCGGTGGCCGACAGGC-TTCGGG-GCGCGCCCTGTG-------CGTTAAC-GCGTGC---
-AGGTGTCCGC--GCAC-CGTTGTCC-TTGTAGG-CCGACT-CAATCCTACCGCGGTGCT-
CTTTACCGAGTGTCGAGGTAGGCCGACACT-
GTTTACTTTGAACAAATTAGAGTGCTTCAAGCAGGCTCTGAAAGC-
AGCCTGAATAGTGGTGCATGGAATAATGGAACAGGACCTTGGTTCTATTTTGTT-GGTTTTCGGA-
ATCCAAGGTAATGATCAATAAGGA-CAGGCGGGGGCATTCGTATTGCGACGTTAGA-----------------
-GGTGAAATTCTTGG-
ATCGTCGCAAGACGCACTAGAGCGAAAGCATTTGCCAAGTATGTCTTAATTGATCAAGCACGATAGTTAGAGG
TTCGAAGGCGATCAGATACCGCCCTAGTYCTAACCATAAACKATGCCAACCAGCGATCCGCCGATGTTCCTCC
GATGACTCGGCGGGGAGCTT-
TCCCCGGGAAACCAAAGCTTTTGGGTTCCGGGGGAAGTATGGTTGCAAAGCTGAAACTTAAAGGAATTGACGG
AAGGGCACCACCAGGAGTGGAGCCTGCGGCTTAATTTGACTCAACACGGGAAACCTCACCAGGCCCGGACATT
GGAA-GGATTGACAGAT-CGATAGCTCTTT-CTCGATT-CAGTGGGT-
AGTGGTGCATGGCCGTTCTTAGTTGGTGGACTGATTTGTCTGGTTAATTCCGATAACGAACGAGACTCTGACC
TATTAACTAGGCGTTC-CCGGTATAA-ATA-CCTACCGGCGAGAT-
TTATTCTTCTTAAGGGGACAGGCGGCTCTTAGCCGCACGAGATTGAGCAATAACAGGTCTGTGATGCCCTTAG
ATGTTCTGGGCCGCACGCGCGCTACACTGAAGGAATCAGCGTGTGC--
TCCCCTGTCCGAAAGGTCCGGGCAACCCGCTGAACCTCCTTCGTGCTAGGAATTGGGGCTTGCAATTGTTCCC
CATGAACGAGGAATTCCCAGTAAGCGCGAGTCATAAGCTCGCGTTGATTACKTCCCTGCCCTTTGTACACACC
GCCCGTCGCTACTASCGATTGARTGATTTAGTGAGGTCTTCGGACC-GGCGGCA--AC-AGGC-ACC--GGC-
----TCGTTCGG---TATCGGT-TCCGCTGGAAA-
GATGACCGAACTTGATCATTTAGAGGAAGTAAAAGTCGTAACAAGG-TTTCCAGTCA--TTGGGTGA---
CAA-CCTAAAGGCGCAATGAAAGT-GTCAGC------------GCGGGCCTCCTA-------------
CGGGAGC-----GCCCGCCGTAGGGAGGATGGGCCGCC----CGGCGATCACT--------CGT--
CGGCGCCCCGCACTCCCGGGGCGTCTC--GTTCTC-ATCACG-AG----
TGGAGACGCACCAGGAGCGCACACGCTGGTACCCGAAAGATGGTGAACTATGCCTGGCCAGGACGAAGCCAGG
GGAAACCCTGGTGGAGGTCC--GAAGCG-ATTCTG--ACGTGCAAATCG-
ATCGTCTGAGCTGGGTATAGGGGCGAAAGACCAATCGAACCATCAAA--GTTTATTT--
ATTTCAAGTCGGGCCTGCTCAATGATTTATAATA------

ATTAAATAGCCGCAGTATTTTGACTGTGCGAAGGTAGCATAATCATTTGTCTTTTAATTGAAGGCTTGTATGA
AGGGTTTGACAAAATGTGGGCTTTCTTTACATTAAATCGTT-----
GAATTTAATTTTTGAGTTAAAAAGCTTAAATGTTT-TAATAGGACGAGAAGACCCTATAGAA--TTTTATT--
---------ATTTGTGGTTTATATTATAATTGGTTTATTTA--------
TTTATTATATGTTATAAATAATTTTGTTGGGGTGATGGGGAAA-TTTTCTTAACTTTCTTTATT--TATTTA-
ATATAAATTAATATA-----TTAATGATCCTT----TTT--ATGGATA--
ATAAGTTTAAATTACCTTAGGGATAAC
Eusthenes_cupreus
ATGCAAGTCTCAGTACGAGCTGACT-
TAAAGTGAAACCGCAAAAGGCTCAGTAAACCGGTCATAGTTTACCGGA-TATATG-CCACTC-
ACGTGGATAACTGTGGTAATTCTAGAGCTAATACATGCAACC-AAGTCCCGACCAGCG---
ATGGAAAGGACGCTTTTATTGGATCAAAACCATTCGCCCGGCT-CGCCCGGGC------
GTCTGCTTGATGACTCT-AGATAAC-ACCACA-GATCGCACGGTCACAGTACCGGC-
GACGTATCCGTCAAATGTCTGACTTATCAGCTGTCGATGGTAGACTCTGTGTCTACCATGGCGCTCACGGGTA
ACGGGGAATCAGGGTTCGATTCCGGAGAGGGAGCCTGAGAAACGGCTACCACATCCAAGGAAGGCAGCAGGCA
CGCAAATTACCCACTCCCGGCACGGGGAGGTAGTGACAAAAAATAACGATACGGGACTCATCCGAGGCCCCGT
AATCGGAATGAGAACACCCTAAATCCTTTAACGAGGATCTATTGGAGGGCAGGTCTGGTGCCAGCAGCCGCGG
TAATTCCAGCTCCAATAGCGTATATTAAAGTTGTTGCGGTTAAAAA-
GCTCGTAGTTGGTTCTGCGTGCCACGCTGTCGGTTCACCGCCTGTCGTTGCAACTGGCATGCC-
GTGGCATGTCCTGTCGGTGGCTGACAGGC-TTCACG-GCGCGCCCTGTG-------CGTTTAC-GCGTGC---
-AGGTTGCTGC--GCCG-CGTTGTCC-TTGTAGG-CCGGCT-CAATCCTACCGCGGTGCT-
CTTTGCCGAGTGTCGAGGTAGGCCGACACT-
GTTTACTTTGAACAAATTAGAGTGCTTCAAGCAGGCTCTTAAAGC-
AGCCTGAATAGTGGTGCATGGAATAATGGAACAGGACCTTGGTTCTATTTTGTT-GGTTTCCGGA-
ATCCAAGGTAATGATCAATAAGGA-CAGGCGGGGGCATTCGTATTGCGACGTTAGA-----------------
-GGTGAAATTCTTGG-
ATCGTCGCAAGACGCACTAGAGCGAAAGCATTTGCCAAGTATGTCTTAATTGATCAAGAACGAAAGTTAGAGG
TTCGAAGGCGATCAGATACCGCCCTAGTTCTAACCATAAACGATGCCAGCCAGCGATCCGCCGATGTTCCTCC
GATGACTCGGCGGGGAGCTT-
TCCCCGGGAAACCAAAGCTTTTGGGTTCCGGGGGAAGTATGGTTGCAAAGCTGAAACTTAAAGGAATTGACGG
AAGGGCACCACCAGGAGTGGAGCCTGCGGCTTAATTTGACTCAACACGGGAAACCTCACCAGGCCCGGACATT
GGAA-GGATTGACAGAT-CGATAGCTCTTT-CTCGATT-CAGTGGGT-
AGTGGTGCATGGCCGTTCTTAGTTGGTGGACTGATTTGTCTGGTTAATTCCGATAACGAACGAGACTCTGACC
TATTAACTAGGCGCTC-CCGGTATAA-ATA-CCTACCGGCGAGAT-
TTATTCTTCTTAAGGGGACAGGCGGCTCTCAGCCGCACGAGATTGAGCAATAACAGGTCTGTGATGCCCTTAG
ATGTTCTGGGCCGCACGCGCGCTACACTGAAGGAATCAGCGTGTGC--
TCCCCTGTCCGAAAGGTCCGGGCAACCCGCTGAACCTCCTTCGTGCTAGGAATTGGGGCTTGCAATTGTTCCC
CATGAACGAGGAATTCCCAGTAAGCGCGAGTCATAAGCTCGCGTTGATTACGTCCCTGCCCTTTGTACACACC
GCCCGTCGCTACTACCGATTGAATGATTTAGTGAGGTCTTCGGACC-GGCGGTA--AC-CGGC-ACC--GGC-
----TCGTTCGG---TATCGGT-TTCGCTGGAAA-
GATGACCGAACTTGATCATTTAGAGGAAGTAAAAGTCGTAACAAGG-TTTCCAGTCA--TTGGGTGA---
AAAACCTAAAGGCGCAATGAAAGT-GTCAG---------------GGGTTGCCTT-------------TAAG-
-------GCTCCCCGTAGGGAGGATGGGCCCC-----GTACGGCTTGCT-------CGT--
CGGAGCCCCGCACTCCCGGGGCGTCTC--GTTCTC-ATCACG-AG----
TGGAGACGCACCAGGAGCGCACACGCTGGTACCCGAAAGATGGTGAACTATGCCTGGCCAGGACGAAGCCAGG
GGAAACCCTGGTGGAGGTCC--GAAGCG-ATTCTG--ACGTGCAAATCG-
ATCGTCTGAGCTGGGTATAGGGGCGAAAGACCAATCGAACCATCT---GTTAAGTTT--
ATATCAGGTCTGGCCTGCCCACTGATAA------TTATA--
TTGAAGGGCCGCGGTATTTTGACCGTGCGAAGGTAGCATAATCATTTGTCTTTTAATTGTAGGCTTGTATGAA
TGGTTTGATGAAATATAAACTTTCTTTTATTTATAATAT-----
TTAATTTAATTTTTTAGTTAAAAAGCTAAAATTGTC-TTGTTGGACGAGAAGACCCTATAGAA--TTTTACT-
---------TGATCATTTA-TATTTACTTTTT-TTGGTTATTA-----
ATTTAAGTGTATATTATGTTAAGTTGTGTTGGGGCGACAGGGAAA-TTTATTTAACTTTCTTTATT-
TATATC--TTATTTATATATATA-----TTAATGATCCTAAA---TT--TTGGATA--
TTAAGATTAAATTACCTTAGGGATAAC
Fromundus_biimpressus
ATGCAAGTCTCAGTACGAGCTGACT-
TAAAGTGAAACCGCAAAAGGCTCAGTAAACCGGTCATAGTTTACCGGA-TATATG-CCACTC-

ACGTGGATAACTGTGGTAATTCTAGAGCTAATACATGCAACC-AAGTCCCGACCAGCG---
ATGGAAAGGACGCTTTTATTGGATCAAAACCATTCGCCCGGCT-CGCCCGGGC------
GTCTGCTTGATGACTCT-AGATAAC-ACCACA-GATCGCACGGTCACAGTACCGGC-
GACGTATCCGTCAAATGTCTGACTTATCAGCTGTCGATGGTAGACTCTGTGTCTACCATGGCGCTCACGGGTA
ACGGGGAATCAGGGTTCGATTCCGGAGAGGGAGCCTGAGAAACGGCTACCACATCCAAGGAAGGCAGCAGGCA
CGCAAATTACCCACTCCCGGCACGGGGAGGTAGTGACAAAAAATAACGATACGGGACTCATCCGAGGCCCCGT
AATCGGAATGAGAACACCCTAAATCCTTTAACGAGGATCTATTGGAGGGCAAGTCTGGTGCCAGCAGCCGCGG
TAATTCCAGCTCCAATAGCGTATATTAAAGTTGTTGCGGTTAAAAA-
GCTCGTAGTTGGTTCTGCGTGCCACGCTGTCGGTTCACCGCCTGTCGGTGTAACTGGCATGCC-
GTGGCATGTCCTGTCGGTGGCTGACAGGC-TTCGCG-GCGCGCCCTATG-------CGTTTAC-GCGTGT---
-AGGTTGTCGA--GCCGACGTTGTCC-TTGTAGG-CCGGCT-CAATCCTACCGCGGTGCT-
CTTTGCCGAGTGTCGAGGTAGGCCGACACT-
GTTTACTTTGAACAAATTAGAGTGCTTCAAGCAGGCTCTGAAAGC-
AGCCTGAATAGTGGTGCATGGAATAATGGAACAGGACCTTGGTTCTATTTTGTT-GGTTTCCGGA-
ATCCAAGGTAATGATCAATAAGGA-CAGGCGGGGGCATTCGTATTGCGACGTTAGA-----------------
-GGTGAAATTCTTGG-
ATCGTCGCAAGACGCACTAGAGCGAAAGCATTTGCCAAGTATGTCTTAATTGATCAAGAACGAAAGTTAGAGG
TTCGAAGGCGATCAGATACCGCCCTAGTTCTAACCATAAACGATGCCAGCCAGCGATCCGCCGATGTTCCTCC
GATGACTCGGCGGGGAGCTT-
TCCCCGGGAAACCAAAGCTTTTGGGTTCCGGGGGAAGTATGGTTGCAAAGCTGAAACTTAAAGGAATTGACGG
AAGGGCACCACCAGGAGTGGAGCCTGCGGCTTAATTTGACTCAACACGGGAAACCTCACCAGGCCCGGACATT
GGAA-GGATTGACAGAT-CGATAGCTCTTT-CTCGATT-CAGTGGGT-
AGTGGTGCATGGCCGTTCTTAGTTGGTGGACTGATTTGTCTGGTTAATTCCGATAACGAACGAGACTCTGACC
TATTAACTAGGCGCTC-CCGGTATAA-ATA-CCTACCGGCGAGAT-
TTATTCTTCTTAAGGGGACAGGCGGCTCTTAGCCGCACGAGATTGAGCAATAACAGGTCTGTGATGCCCTTAG
ATGTTCTGGGCCGCACGCGCGCTACACTGAAGGAATCAGCGTGTGC--
TCCCCTGGCCGAAAGGTCCGGGCAACCCGCTGAACCTCCTTCGTGCTAGGAATTGGGGCTTGCAATTGTTCCC
CATGAACGAGGAATTCCCAGTAAGCGCGAGTCATAAGCTCGCGTTGATTACGTCCCTGCCCTTTGTACACACC
GCCCGTCGCTACTACCGATTGAATGATTTAGTGAGGTCTTCGGACC-GGCGGTA--AC-CGGC-ACC--GGC-
----TCGTTCGG---TATCGGT-TTCGCTGGAAA-
GATGACCGAACTTGATCATTTAGAGGAAGTAAAAGTCGTAACAAGG-TTTCCAGTCA--TTGGGTTC---
AAA-CCTAAAGGCGCAATGAAAGT-GTCAG-------------GTGGGACTCCTT-------------
TAGGAGC-----TCCCACCGCAGGGAGGATGGGCCCCC----GTACATCTACT---------GT--
CGGAGCCCCGCACTCCCGGGGCGTCTC--GTTCTC-ATCACG-AG----
TAGAGACGCACCAGGAGCGCACACGCTGGTACCCGAAAGATGGTGAACTATGCCTGGCCAGGACGAAGCCAGG
GGAAACCCTGGTGGAGGTCC--GAAGCG-ATTCTG--ACGTGCAAATCG-
ATCGTCTGAGCTGGGTATAGGGGCGAAAGACCAATCGAACCATCCCA-GC--ATCGT--A-
ATTGAGTCACTGCCGGTCCATGATACTT-----CA---
ATTAAATGGCCGCGGTATTTTGACCGTGCAAAGGTAGCATAATCACTTGTCCTTTAATTGAGGGCTTGTATGA
ATGGTTCGATGAAGTGCTGACTTTCTTTATCTTTTGTTCTA-----
AAATTTATAGTCTCAGTTAAAAAGCTGGGATGATT-CCATAGGACGAGAAGACCCTATTGAA--TTTTACTC-
------TTATTTTCTATCATATTATATTTTAAGGATATTAG----ATATAAATTGTAATTTAGGC-
AAAGTTTTGTTGGGGTGACGGGTGAC-CTATTTTAACTTTCTATCTT--CCGATT-TTATTGAACATTATA--
--CCCTCTGATCCTGGT---TA--ACTGATA--ATATAATGATCTTACCTTAGGGATAAC
Fromundus_difficilis
ATGCAAGTCTCAGTACGAGCTGACT-
TAAAGTGAAACCGCAAAAGGCTCAGTAAACCGGTCATAGTTTACCGGA-TATATG-CCACTC-
ACGTGGATAACTGTGGTAATTCTAGAGCTAATACATGCAACC-AAGTCCCGACCAGCG---
ATGGAAAGGACGCTTTTATTGGATCAAAACCATTCGCCCGGCT-CGCCCGGGC------
GTCTGCTTGATGACTCT-AGATAAC-ACCACA-GATCGCACGGTCACAGTACCGGC-
GACGTATCCGTCAAATGTCTGACTTATCAGCTGTCGATGGTAGACTCTGTGTCTACCATGGCGCTCACGGGTA
ACGGGGAATCAGGGTTCGATTCCGGAGAGGGAGCCTGAGAAACGGCTACCACATCCAAGGAAGGCAGCAGGCA
CGCAAATTACCCACTCCCGGCACGGGGAGGTAGTGACAAAAAATAACGATACGGGACTCATCCGAGGCCCCGT
AATCGGAATGAGAACACCCTAAATCCTTTAACGAGGATCTATTGGAGGGCAAGTCTGGTGCCAGCAGCCGCGG
TAATTCCAGCTCCAATAGCGTATATTAAAGTTGTTGCGGTTAAAAA-
GCTCGTAGTTGGTTCTGCGTGCCACGCTGTCGGTTCACCGCCTGTCGGTGCAACTGGCATGCC-
GTGGCATGTCCTGTCGGTGGCTGACAGGC-TTCGCG-GCGCGCCCTATG-------CGTTTAC-GCGTGT---
-AGGTCGTCGC--GCCGACGTTGTCC-TTGTAGG-CCGGCT-CAATCCTACCGCGGTGCT-

CTTTGCCGAGTGTCGAGGTAGGCCGACACT-
GTTTACTTTGAACAAATTAGAGTGCTTCAAGCAGGCTCTGAAAGC-
AGCCTGAATAGTGGTGCATGGAATAATGGAACAGGACCTTGGTTCTATTTTGTT-GGTTTTCGGA-
ATCCAAGGTAATGATCAATAAGGA-CAGGCGGGGGCATTCGTATTGCGACGTTAGA-----------------
-GGTGAAATTCTTGG-
ATCGTCGCAAGACGCACTAGAGCGAAAGCATTTGCCAAGTATGTCTTAATTGATCAAGAACGAAAGTTAGAGG
TTCGAAGGCGATCAGATACCGCCCTAGTTCTAACCATAAACGATGCCAGCCAGCGATCCGCCGATGTTCCTCC
GATGACTCGGCGGGGAGCTT-
TCCCCGGGAAACCAAAGCTTTTGGGTTCCGGGGGAAGTATGGTTGCAAAGCTGAAACTTAAAGGAATTGACGG
AAGGGCACCACCAGGAGTGGAGCCTGCGGCTTAATTTGACTCAACACGGGAAACCTCACCAGGCCCGGACATT
GGAA-GGATTGACAGAT-CGATAGCTCTTT-CTCGATT-CAGTGGGT-
AGTGGTGCATGGCCGTTCTTAGTTGGTGGACTGATTTGTCTGGTTAATTCCGATAACGAACGAGACTCTGACC
TATTAACTAGGCGCTC-CCGGTATAA-ATA-CCTACCGGCGAGAT-
TTATTCTTCTTAAGGGGACAGGCGGCTCTTAGCCGCACGAGATTGAGCAATAACAGGTCTGTGATGCCCTTAG
ATGTTCTGGGCCGCACGCGCGCTACACTGAAGGAATCAGCGTGTGC--
TCCCCTGGCCGAAAGGTCCGGGCAACCCGCTGAACCTCCTTCGTGCTAGGAATTGGGGCTTGCAATTGTTCCC
CATGAACGAGGAATTCCCAGTAAGCGCGAGTCATAAGCTCGCGTTGATTACGTCCCTGCCCTTTGTACACACC
GCCCGTCGCTACTACCGATTGAATGATTTAGTGAGGTCTTCGGACC-GGCGGTA--AC-CGGC-ACC--GGC-
----TCGTTCGG---TATCGGT-TTCGCTGGAAA-
GATGACCGAACTTGATCATTTAGAGGAAGTAAAAGTCGTAACAAGG-TTTCCAGTCA--TTGGGTG----
AAA-CCTAAAGGCGCAATGAAAGT-GTCAG-------------GTGGGGCTCCTT-------------
TAGGAGT-----TCCCTCCGCAGGGAGGATGGGCCGCC----GTACAATTATT--------TGT--
CGGAGCCCCGCACTCCCGGGGCGTCTC--GTTCTC-ATCACG-AG----
TAGAGACGCACCAGGAGCGCACACGCTGGTACCCGAAAGATGGTGAACTATGCCTGGCCAGGACGAAGCCAGG
GGAAACCCTGGTGGAGGTCC--GAAGCG-ATTCTG--ACGTGCAAATCG-
ATCGTCTGAGCTGGGTATAGGGGCGAAAGACCAATCGAACCATCTTG-TATATTT----
TTATCAGGTCGGACCTGCTCAATGATAT-------TAT--
ATTAAATAGCCGCAGTATCTTGACTGTGCGAAGGTAGCATAATCATTTGTCTTTTAATTGTAGGCTTGTATGA
AGGGTTTGATGAAATTCTAGCTTTCTTTATTTTATATTTAA-----
TAATTTAATGTTTAAGTCAAAAGGCTTAGATGATT-CCATAGGACGAGAAGACCCTATAGAG--TTTTATT--
------GTT--ATT-TACATATTTTGTTATAGGT--AATTG----ATTGC-GTTTTATGTAAATG-
ATAATTGTGTTGGGGTGACAGGGAAA-TTTTATTAACTTTCTTTTT-TATTTAT-
TTATTGATGATTATAATAAGATAATGATCCTTTA---TT--ATGGATT--
TTAAGATCAAATTACCTTAGGGATAAC
Fromundus_pygmaeus
ATGCAAGTCTCAGTACGAGCTGACT-
TAAAGTGAAACCGCAAAAGGCTCAGTAAACCGGTCATAGTTTACCGGA-TATATG-CCACTC-
ACGTGGATAACTGTGGTAATTCTAGAGCTAATACATGCAACC-AAGTCCCGACCAGCG---
ATGGAAAGGACGCTTTTATTGGATCAAAACCATTCGCCCGGCT-CGCCCGGGC------
GTCTGCTTGATGACTCT-AGATAAC-ACCACA-GATCGCACGGTCACAGTACCGGC-
GACGTATCCGTCAAATGTCTGACTTATCAGCTGTCGATGGTAGACTCTGTGTCTACCATGGCGCTCACGGGTA
ACGGGGAATCAGGGTTCGATTCCGGAGAGGGAGCCTGAGAAACGGCTACCACATCCAAGGAAGGCAGCAGGCA
CGCAAATTACCCACTCCCGGCACGGGGAGGTAGTGACAAAAAATAACGATACGGGACTCATCCGAGGCCCCGT
AATCGGAATGAGAACACCCTAAATCCTTTAACGAGGATCTATTGGAGGGCAAGTCTGGTGCCAGCAGCCGCGG
TAATTCCAGCTCCAATAGCGTATATTAAAGTTGTTGCGGTTAAAAA-
GCTCGTAGTTGGTTCTGCGTGCCACGCTGTCGGTTCACCGCCTGTCGGTGCAACTGGCATGCC-
GTGGCATGTCCTGTCGGTGGCTGACAGGC-TTCGCG-GCGCGCCCTATG-------CGTTTAC-GCGTGT---
-AGGTCGTCGC--GCCGACGTTGTCC-TTGTAGG-CCGGCT-CAATCCTACCGCGGTGCT-
CTTTGCCGAGTGTCGAGGTAGGCCGACACT-
GTTTACTTTGAACAAATTAGAGTGCTTCAAGCAGGCTCTGAAAGC-
AGCCTGAATAGTGGTGCATGGAATAATGGAACAGGACCTTGGTTCTATTTTGTT-GGTTTCCGGA-
ATCCAAGGTAATGATCAATAAGGA-CAGGCGGGGGCATTCGTATTGCGACGTTAGA-----------------
-GGTGAAATTCTTGG-
ATCGTCGCAAGACGCACTAGAGCGAAAGCATTTGCCAAGTATGTCTTAATTGATCAAGAACGAAAGTTAGAGG
TTCGAAGGCGATCAGATACCGCCCTAGTTCTAACCATAAACGATGCCAGCCAGCGATCCGCCGATGTTCCTCC
GATGACTCGGCGGGGAGCTT-
TCCCCGGGAAACCAAAGCTTTTGGGTTCCGGGGGAAGTATGGTTGCAAAGCTGAAACTTAAAGGAATTGACGG
AAGGGCACCACCAGGAGTGGAGCCTGCGGCTTAATTTGACTCAACACGGGAAACCTCACCAGGCCCGGACATT

GGAA-GGATTGACAGAT-CGATAGCTCTTT-CTCGATT-CAGTGGGT-
AGTGGTGCATGGCCGTTCTTAGTTGGTGGACTGATTTGTCTGGTTAATTCCGATAACGAACGAGACTCTGACC
TATTAACTAGGCGCTC-CCGGTATAA-ATA-CCTACCGGCGAGAT-
TTATTCTTCTTAAGGGGACAGGCGGCTCTTAGCCGCACGAGATTGAGCAATAACAGGTCTGTGATGCCCTTAG
ATGTTCTGGGCCGCACGCGCGCTACACTGAAGGAATCAGCGTGTGC--
TCCCCTGGCCGAAAGGTCCGGGCAACCCGCTGAACCTCCTTCGTGCTAGGAATTGGGGCTTGCAATTGTTCCC
CATGAACGAGGAATTCCCAGTAAGCGCGAGTCATAAGCTCGCGTTGATTACGTCCCTGCCCTTTGTACACACC
GCCCGTCGCTACTACCGATTGAATGATTTAGTGAGGTCTTCGGACC-GGCGGTA--AC-CGGC-ACC--GGC-
----TCGTTCGG---TATCGGT-TTCGCTGGAAA-
GATGACCGAACTTGATCATTTAGAGGAAGTAAAAGTCGTAACAAGG-TTTCCAGTCA--TTGGGTGA---
AAAACCTAAAGGCGCAATGAAAGT-GTCAG-------------GTGGGTCTCCTT-------------
TAGGAGT-----TCCCACCGCAGGGAGGATGGGCCACC----GTACATTTATT---------GT--
CGGAGCCCCGCACTCCCGGGGCGTCTC--GTTCTC-ATCACG-AG----
TGGAGACGCACCAGGAGCGCACACGCTGGTACCCGAAAGATGGTGAACTATGCCTGGCCAGGACGAAGCCAGG
GGAAACCCTGGTGGAGGTCC--GAAGCG-ATTCTG--ACGTGCAAATCG-
ATCGTCTGAGCTGGGTATAGGGGCGAAAGACCAATCGAACCATCTTG-TATAATT----
TTTTTAGGTCGGGCCTGCTCAGTGATTA-------TAT-
AATTAAATAGCCGCAGTATTTTGACTGTGCGAAGGTAGCATAATCATTTGTCTTTTAATTGTAGGCTAGTATG
AAGGGTTTGATGAGGTGCTAACTATCTTAGTTTTATTTTTGG-----
GAATTTAATGTTTAAGTTAAAAGGCTTAAATTTTT-TCATAGGACGAGAAGACCCTATAGAG--TTTTACT--
------TCTAGTTT-TGGATAATTTATCTTTGGT--ATTTA----ATTATATTTTATTTGAGTAG--
GAGTTTTGTTGGGGTGACAGGGAAA-TTTTACTAACTTTCTTTATT-TATATAT-TTAATTATGATTAT----
-TTTAAAGATCCTTTAATATTTTATGGATA--GTAAGATTAAATTACCTTAGGGATAAC
Galgupha_australis
ATGCAAGTCTCAGTACGAGCTGAAT-
TAAAGTGAAACCGCTAAAGGCTCAGTAAACCGGTCATAGTTTACCGGA-TATATG-CCACAT-
ACGTGGACAACTGTGGTAATTCTAGAGCTAATACATGAAACA-AAGTCCCGACCAGCG---
ATGGAAAGGACGCTTTTATTGGATCAAAACCATTCGCCCGGCT-CGACCGGGC------
GTTCTCTTGACGACTCT-AGATAAC-AAAACA-GATCGCACGGTCACAGTGCCGGC-
GACTTATCCGTCAAATGTCTGACTTATCAGCTGTCGATGGTAGACTCTGTGTCTACCATGGCGCTCACGGGTA
ACGGGGAATCAGGGTTTGATTCCGGAGAGGGAGCCTGAGAAACGGCTACCACATCCAAGGAAGGCAGCAGGCA
CGCAAATTACCCACTCCCGGCACGGGGAGGTAGTGACAAAAAATAACGATACGGGACTCATCCGAGGCCCCGT
AATCGGAATGAGAACACCCTAAATCCTTTAACGAGGATCTATTGGAGGGCAAGTCTGGTGCCAGCAGCCGCGG
TAATTCCAGCTCCAATAGCGTATATTAAAGTTGTTGCGGTTAAAAA-
GCTCGTAGTTGGTTCTGCGTGCCACGCTGTCGGTTCACCGCCTGTCGGTGCAACTGGCATGCC-
GTGGCATGTCCTGTCGGTGGCTGACAGGC-TTCTCG-GCGCGCCCTATG-------CGTTCAC-TCGTGT---
-AGGTCGTTGC--GCCG-AGTTGTCCCTTGTAGG-CCGGCT-
CAATCCTACCGCGGTGCTTCTTTGCCGAGTGTCGAGGTAGGCCGACACTTGTTTACTTTGAACAAATTAAAGT
GCTTTCAAGCAGCTCTGAAAGC-
AACCTGAATAGTGGTGCATGGAATAATGGAACAGGACCTTGGTTCTATTTTGTT-GGTTTTCGGA-
ATCCAAGGTAATGATCAATAAGGA-CAGGCGGGGGCTTACGTATTGCGACGTTAGA-----------------
-GGTGAAATTCTTGG-
ATCGTCGCAAGACGCACTAGAGCGAAAGCATTTGCCAAGTATGTCTTAATTGATCAAGAACGAAAGTTAGAGG
ATCGAAGGCGATCAGATACCGCCCTAGTTCTAACCATAAACGATGCCAGCCAGCGATCCGCCGATGTTCCTCC
GATGACTCGGCGGGGAGCTT-
TCCCCGGGAAACCAAAGCTTTTGGGTTCCGGGGGAAGTATGGTTGCAAAGCTGAAACTTAAAGGAATTGACGG
AAGGGCACCACCAGAAGTGGAGCCTGCGGCTCAATTTGACTCAACACGGGAAACCTCACCAGGCCCGGACATT
GGAA-GGATTGACAGAT-CGATAGCTCTTT-CTCGATT-CAGTGGGT-
AGTGGTGCATGGCCGTTCTTAGTTGGTGGACTGATTTGTCTGGTTAATTCCGATAACGAACGAGACTCTGACC
TATTAACTAGGCGTTT-CCGGTATAA-ATA-CCTACCGGCGAGAT-
TTATTCTTCTTAAGGGGACAGGCGGCTCTTAGCCGCACGAGATTGAGCAATAACAGGTCTGTGATGCCCTTAG
ATGTTCTGGGCCGCACGCGCGCTACACTGAAGGAATCAGCGTGTTC--
TCCCCTGTCCGAAAGGTCCGGGCAACCCGCTGAACCTCCTTCGTGCTAGGAATTGGGGCTTGCAATTGTTCCC
CATGAACGAGGAATTCCCAGTAAGCGCGAGTCATAAGCTCGCGTTGATTACGTCCCTGCCCTTTGTACACACC
GCCCGTCGCTACTACCGATTGAATGATTTAGTGAGGTCTTCGGACC-GGCGGTA--AC-CGGC-ACC--GGC-
----TCGTTCGG---TATCGGT-TTCGCTGGAAA-
GATGACCGAACTTGATCATTTAGAGGAAGTAAAAGTCGTAACAAGG-TTTCCAGTC---TTGGGTGT---
AAA-CCTAAAGGCGCAATGAAAGT-GTCAG-------------GGGGGCCTCCTT-------------

TAGGAGC-----GTCTCCCGCGGGGAGGATGGGCTTCC----GTACAATTACT--------TGT--
CGGAGCCCCGCACTCCCGGGGCGTCTC--GTTCTC-GTTACG-AG----
TGGAGACGCACCAGGAGCGCACACGCTGGTACCCGAAAGATGGTGAACTATGCCTGGCCAGGACGAAGCCAGG
GGAAACCCTGGTGGAGGTCC--GAAGCG-ATTCTG--ACGTGCAAATCG-
ATCGTCTGAGCTGGGTATAGGGGCGAAAGACCAATCGAACTATCAGGGTTAATATTT--
ATTTTAGGTCTGGCCTGCTCAATGATCTAT----TATG-
GATTAAATGGCCGCAGTATTTTGACTGTGCGAAGGTAGCATAATCATTTGTCTTTTAATTGAAGGCTTGTATG
AATGGTTTAATGAGACACACACTTTCTTCATATCATATATTAT---
TTAATTTAATGTTTGAGTTAAAAAGCTTAAATTTAT-TTATAGGACGAGAAGACCCTATAGAA--TTTTGAT-
--------TTATATATTATATATTTAATGGTTTGGTTATTTC-----TCATT-
AATGTTTATTGTTATGAATTTTGTTGGGGCGACAGGGAAA-TTTTAGTAACTTTCTTTATT--TATATA-
TTATTTATTTATATA-----TTAATGATCCTATT---TT---AGGATA--
ATAAGATTAAATTACCTTAGGGATAAC
Garsauria_aradoides
ATGCAAGTCTCAGTACAGATTGATT-
TAAAGTGAAACCGCAAAAGGCTCAGTAAACCGGTCATAGTTTACCGGA-TATATG-CCACTC-
ACGTGGATAACTGTGGTAATTCTAGAGCTAATACATGCAACC-AAGTCCCGACCAGAG---
ATGGAAAGGACGCTTTTATTGGATCAAAACCATTCGCCCGGTT-CGCCCGGGC------
GTCTGCTTGATGACTCT-AGATAAC-ACCACA-GATCGCACGGTCACAGTACCGGC-
GACGTATCTTTCAAATGTCTGACTTATCAGCTGTCGATGGTAGACTCTGTGTCTACCATGGCGCTCACGGGTA
ACGGGGAATCAGGGTTCGATTCCGGAGAGGGAGCCTGAGAAACGGCTACCACATCCAAGGAAGGCAGCAGGCA
CGCAAATTACCCACTCCCGGCACGGGGAGGTAGTGACAAAAAATAACGATACGGGACTCATCCGAGGCCCCGT
AATCGGAATGAGAACATCCTAAATCCTTTAACGAGGATCTATTGGAGGGCAAGTCTGGTGCCAGCAGCCGCGG
TAATTCCAGCTCCAATAGCGTATATTAAAGTTGTTGCGGTTAAAAA-
GCTCGTAGTTGGTTCTGCGTGCCGCGCCGTCGGTTCGCCGCCTGTCGGTGTAACTGGCGTGCC-
GCGGCATGTCCTGTCGGTGGCCGACAGGC-TTCGCG-GCGTGCCCTATG-------CGTTTAA-ACGTGT---
-AGGTCGTTGC--GCCGACGTTGTCC-TTGTAGG-CCGGCT-CAATCCTACCGCGGTGCT-
CTTTACCGAGTGTCGAGGTAGGCCGACACT-
GTTTACTTTGAACAAATTAGAGTGCTTAAAGCAGGCTATGAAAGC-
AGCCTGAATAGTGGTGCATGGAATAATGGAAAAGGACCTTGGTTCTATTTTGTT-GGTTTTCGGA-
ATCCAAGGTAATGATCAATAAGGA-CAGGCGGGGGCATTCGTATTGCGACGTTAGA-----------------
-GGTGAAATTCTTGG-
ATCGTCGCAAGACGCACTAAAACGAACGCATTTGCCAAGTATGTCTTAATTGATCAAGAACGAAAGTTAGAGG
TTGGAAGGCGATCAGATACCGCCCTAGTTCTAACCATAAACGATGCCAGCCAGCGATCCGCCGACGTTCCTCC
GATGACTCGGCGGGGAGCTT-
TCCCCGGGAAACCAAAGCTTTTGGGTTCCGGGGGAAGTATGGTTGCAAAGCTGAAACTTAAAGGAATTGACGG
AAGGGCACCACCAGAAGTGGAGCCTGCGGCTTAATTTGACTCAACACGGGAAACCTCACCAGGCCCGGACATT
GGAAGG-ATTGACAGAT-CGATAGCTCTTT-CTCGATT-CAGTGGGT-
AGTGGTGCATGGCCGTTCTTAGTTGGTGGACTGATTTGTCTGGTTAATTCCGTTGACGAACGAGAATCTAGCC
TATTAACTAGGCGCTC-CCGGTATAG-ATA-CCTACCGGCGAGAT-
TTATTCTTCTTAAGGGGACAGGCGGCTTTCAGCCGCACGAGATTGAGCAATAACAGGTCAGTGATGCCCTTAG
ATGTTTTGGGCCGCACGCGCGCTACACTGAAGGAATCAGCGTGTGC--
TCCCCTGTCCGAAAGGTCCGGGTAACCCGCTGAACCTCCTTCGTGCTAGGAATTGGGGCTTGCAATTGTTCCC
CATGAACGAGGAATTCCCAGTAAGCGCGAGTCATAAGCTCGCGTTGATTACGTCCCTGCCCTTTGTACACACC
GCCCGTCGCTACTACCGATTGAATGATTTAGTGAGGTCTTCGGACC-GGCGACAGTAACCGGC-ACC--
GGCT---CTCGTTCGGC--TATCGGT-TTCGCTGGAAA-GATGACCGAC-
TTGATCATTTAGAGGAAGTAAAAGTCGTACCATTG-CCCCAAGACA--TTGGGTGT--
GAAAACCTAAAGGCGCAATGAAAGT-GTCTGC------------GGTCTTTAATTTTT-----------
TAAAGGC----------CGTTGGGGAGGATGGGCTCCC----GTCCCTCACGG---------GT--
CGGTGCCCCGCACTCCCGGGGCGTCTC--GCCGCTCATCACG-AG----
CTGAGACGCACCAGGAGCGCACACGCTGGTACCCGAAAGATGGTGAACTATGCCTGGCCAGGACGAAGCCAGG
GGAAACCCTGGTGGAGGTCC--GAAGCG-ATTCTG--ACGTGCAAATCG-
ATCGTCTGAGCTGGGTATAGGGGCGAAAGACCAATCGAACCATCTTT----TAATTT--
ATATTAAGTCTGGCCTGCTCAATGAATAT-------
ATTGTTTTAATAGCCGCAGTATTTTGACTGTGCAAAGGTAGCATAATCATTTGTCTTTTAATTGGAGGCTTGT
ATGAACGGTCGAATGAAATATAAACTTTCTTTATTATTTATTTTA-----
TAATTTAATTTTTGAGTTAAAAAGCTTAAATAAGCTATGGAGGACGAGAAGACCCTATAGAG--
TTTTATATTTTTTATT----------ATTAATTTATTAATAAATTAATTA---------

AATTAATTTTAAATTAATATTTTATTGGGGCAATGGGAATA-TTTTTATAATTATTCTTATTTA-ATTA--
TTATTAATTTGTA-AA----TTTAAGATCCTT-----TATTATGGATT--
ATAAGTTTAAATTACCTTAGGGATAAC
Geocoris_ater_pallidipennis
ACGCAAGTCTCAGTACGAGCTAAAT-
TAAAGTGAAACCGCAAAAGACTCAGTAAACCGGTCATAGTTTACCGGA-TAAATG-CCACTT-
ACGTGGATAACTGTGGTAATTCTAGAGCTAATACATGCATAA-AAGTCCTGACCAGCG---
ATGGAAGGGACGCTTTTATTTGAACAAAACCAATCGCCTGGCT-CGTCCGGGC------
GTAACCTTGGTGAAGCT-AGATAAC-AACACA-GATCGCACGGTCACCGTACCGGT-
GACGTATCCTTCAAATGTCTGACTTATCAGCTGTCGATGGTAGACTCTGTGTCTACCATGGCTCTCACGGGTG
ACGGGGAATCAGGGTTCGATTCCGGAGAGGGAGCCTGAGAAACGGCTACCACATCCAAGGAAGGCAGCAGGCA
CGCAAATTACCCACTCCCGGCACGGGGAGGTAGTGACAAAAAATAACGATACGGGACTCATCCGAGGCCCCGT
AATCGGAATGAGAACACTCTAAATCCTTTAACGATGATCTATTGGAGGGAAAGTCTGGTGCCAGCAGCCGCGG
TAATTCCAGCTCCAATAGCGTATATTAAAGTTGTTGCGGTTAAAAA-
GCTCGTAGTTGGTTCTGCGTGCCACGCTGTCGGTTCACCGCCTGCCGGTGCAACTGGCATGCC-
GTGGCATGTCCTGTCGGTGGCGGATAGGC-CTCACG-GCGTGCCCTGCGCGG----TATCACTACCGTGT---
-AGTTGGCCGC--GCCGCGT-TGTCC-TTGTACG-CCGGCT-CAATCCTGCCGCGGTGCT-
CTTTACCGAGTGTCGAGGTAGGCCGACACT-
GTTTACTTTGAACAAATTAGAGTGCTTCAAGCAGGCTCTTAAATC-
AGCCTGGATAGTGGTGCATGGAATAATGGAACAGGACCTTGGTTCTATTTTGTT-GGTTTACGGA-
ATTCAAGGTAATGATCAATAAGGA-CAGGCGGGGGCATTCGTATTGCGGCGTTAGA-----------------
-GGTGAAATTCTTGG-
ATCGTCGCAAGACGCACTAAAGCGAAAGCATTTGCCAAGTATGTCTTAATTGATCAAGAACGAAAGTTAGAGG
TCCGAAGGCGATCAGATACCGCCCTAGTTCTAACCATAAACGATGCCAGCCAGCGATCCGCCGACGTTCCTCC
GATGACTCGGCGGGGAGCTT-
TCCCCGGGAAACCAAAGCCTTTGGGTTCCGGGGGAAGTATGGTTGCAAAGCTGAAACTTAAAGGAATTGACGG
AAGGGAACCACCAGGAGTGGTGTCTGCGGCTTAATTTGACTCAACACGGGACATCCCACCAGGCCCGGACATT
GGAA-GGATTGACAGAT-CGATAGCTCTTT-CTCGATT-CGGTGGGT-
AGTGGTGCATGGCCGTTCTTAGTTGGTGGGCTGACTTGTCTGGTTAATTCCGATAACGAGCGAGACTCTAACC
TATTAACTAGGCGATA-CCGGTATATA-TA-GCTACCGGCGATAT-
TTATTCTTCTTAAGGGGACAGGCGGCTCTCAGCCGCACGAGATTGGGCAATAACAGGTCAGTGATGCCCTTAG
ATGTTCTGGGCCGCACGCGCGCTACACTGAAGGAATCAGCGTGTTC--
TCCCCTGTCCGAAAGGTCCGGGCAACCCGCTAAACCTCCTTCGTGCTAGGAGTTGGGGCTTGCAATTATTCCC
CATGAACGAGGAATTCCCAGTAAGCGCGAGTCATTAACTCGCGTTGATTACGTCCCTTCCCTTTGTACACACC
GCCCGTCGCTACTACCGATTGAATGATTTAGTGAGGTCTTCGGACC-GGCGATA--AC-CGGC-ACC--
GACTTG---TGTTCGG---TATCGAT-TTCGCTGGAAA-
GATGACCGAACTTGATTATTTAGAGGAAGTAAAAGTCGTAACAAGG-TTTCCAGTCA--CTGGGTGG---
AAAACCCAAAGGCGCAATGAAAGT-GTCTG---------------GGGGCGCCCA----------------
GCGC--------CCCCGGTGGGAGGATGGGTTA-CC---GGCCCCCCGGC-------------
CGGGGCCCCGCACTCCCGGGGCGTCTC--GTTCTCGTTTC-G-AG----
TGGAGACGCACCAGGAGCGCACACGCTGTTACCCGAAAGATGGTGAATTATGCCTGGCCAGGACGAAGCCAGG
GGAAACCCTGGTGGAGGTCC--GAAGCG-ATTCTG--ACGTGCAAATCG-
ATCGTCTGAGCTGGGTATAGGGGCGAAAGACCAATCGAACCATCTTG----TTAT-
ATAATATTAGGTCTATCCTGCTCAGTGATTTATT------------
AAATAGCCGCAGTATTTTGACTGTGCAAAGGTAGCATAATCATTTGTTTTTAATTGAAAACTTGTATGAATG
GGTGGACGAGGAATTAACTTTCTTTATTAAAATTAAGT----
TTAATTTAATTTTTTAGTTAAAAAGCTAAAATTTAT-ATTTAAGACGAGAAGACCCTATAGAA--TTTTAT--
------------TAATCTTTTTACATCTAAAA--TTAATTTTT---GTTTTTAGATGTAAAAAGATTAATTT-
-GGTTGGGGTGACTATGAAA-TTAAAATAACTTTCATTATTTATATT---TCATTGATTTATGTTTTT---
AA--GATCCTGTA---TTAT--GGATT--ATAAGATTAAATTACCTTAGGGATAAC
Geotomus_convexus
ATGCAAGTCTCAGTACGAGCTGACT-
TAAAGTGAAACCGCAAAAGGCTCAGTAAACCGGTCATAGTTTACCGGA-TATATG-CCACTT-
ACGTGGATAACTGTGGTAATTCTAGAGCTAATACATGCAACC-AAGTCCCGACCAGCG---
ATGGAAAGGACGCTTTTATTGGATCAAAACCATTCGCCCGGCT-CGCCCGGGC------
GTCTGCTTGATGACTCT-AGATAAC-ACCACA-GATCGCACGGTCACAGTACCGGC-
GACGTATCCGTCAAATGTCTGACTTATCAGCTGTCGATGGTAGACTCTGTGTCTACCATGGCGCTCACGGGTA
ACGGGGAATCAGGGTTCGATTCCGGAGAGGGAGCCTGAGAAACGGCTACCACATCCAAGGAAGGCAGCAGGCA

CGCAAATTACCCACTCCCGGCACGGGGAGGTAGTGACAAAAAATAACGATACGGGACTCATCCGAGGCCCCGT
AATCGGAATGAGAACACCCTAAATCCTTTAACGAGGATCTATTGGAGGGCAAGTCTGGTGCCAGCAGCCGCGG
TAATTCCAGCTCCAATAGCGTATATTAAAGTTGTTGCGGTTAAAAA-
GCTCGTAGTTGGTTCTGCGTGCCACGCTGTCGGTTCACCGCCTGTCGGTGTAACTGGCATGCC-
GTGGCATGTCCTGTCGGTGGCTGACAGGC-TTCGCG-GCGCGCCCTATG-------CGTTTAC-GCGTGT---
-AGGTTGTCGC--TCCGACGTTGTCC-TTGTAGG-CCGACT-CAATCCTACCGCGGTGCT-
CTTTGCCGAGTGTCGAGGTAGGCCGACACT-
GTTTACTTTGAACAAATTAGAGTGCTTCAAGCAGGCTCTGAAAGC-
AGCCTGAATAGTGGTGCATGGAATAATGGAACAGGACCTTGGTTCTATTTTGTT-GGTTTCCGGA-
ATCCAAGGTAATGATCAATAAGGA-CAGGCGGGGGCATTCGTATTGCGACGTTAGA-----------------
-GGTGAAATTCTTGG-
ATCGTCGCAAGACGCACTAGAGCGAAAGCATTTGCCAAGTATGTCTTAATTGATCAAGAACGAAAGTTAGAGG
TTCGAAGGCGATCAGATACCGCCCTAGTTCTAACCATAAACGATGCCAGCCAGCGATCCGCCGATGTTCCTCC
GATGACTCGGCGGGGAGCTT-
TCCCCGGGAAACCAAAGCTTTTGGGTTCCGGGGGAAGTATGGTTGCAAAGCTGAAACTTAAAGGAATTGACGG
AAGGGCACCACCAGGAGTGGGGCCTGCGGCTTAATTTGACTCAACACGGGAAACCTCACCAGGCCCGGACATT
GGAAAGGATTGACAGAT-CGATAGCTCTTT-CTCGATT-CAGTGGGT-
AGTGGTGCATGGCCGTTCTTAGTTGGTGGACTGATTTGTCTGGTTAATTCCGATAACGAACGAGACTCTGACC
TATTAACTAGGCGCTC-CCGGTATAA-ATA-CCTACCGGCGAGAT-
TTATTCTTCTTAAGGGGACAGGCGGCTCTTAGCCGCACGAGATTGAGCAATAACAGGTCTGTGATGCCCTTAG
ATGTTCTGGGCCGCACGCGCGCCACACTGAAGGAATCAGCGTGTGC--
TCCCCTGGCCGAAAGGTCCGGGCAACCCGCTGAACCTCCTTCGTGCTAGGAATTGGGGCTTGCAATTGTTCCC
CATGAACGAGGAATTCCCAGTAAGCGCGAGTCATAAGCTCGCGTTGATTACGTCCCTGCCCTTTGTACACACC
GCCCGTCGCTACTACCGATTGAATGATTTAGTGAGGTCTTCGGACC-GGCGGTA--AC-CGGC-ACC--
GGCGT---TCGTTCGG---TATCGGT-TTCGCTGGAAA-
GATGACCGAACTTGATCATTTAGAGGAAGTAAAAGTCGTAACAAGG-TTTCCAGTCA--TTGGGTGC---
AAA-CCTAAAGGCGCAATGAAAGT-GTCAG-------------GTGGGGCTCCTT-------------
TAGGAGC-----TCCCGCCGGAGGGAGGATGGGCCACC----GTACTTGTGT-----------T--
CGGAGCCCCGCACTCCCGGGGCGTCTC--GTTCTC-ATCACG-AG----
TGGAGACGCACCAGGAGCGCACACGCTGGTACCCGAAAGATGGTGAACTATGCCTGGCCAGGACGAAGCCAGG
GGAAACCCTGGTGGAGGTCC--GAAGCG-ATTCTG--ACGTGCAAATCG-
ATCGTCTGAGCTGGGTATAGGGGCGAAAGACCAATCGAACCATCTTG-TATAATT----
TTATTAGGTCGGGCCTGCTCAGTGATTA-------TTT-
AATTAAATAGCCGCGGTATTTTGACTGTGCGAAGGTAGCATAATCATTTGTCTTTTAATTGTAGGCTTGTATG
AATGGTTTGATGAAGTACAAACTTTCTTTATTTCAATATTAA-----
GAATTTAATGTTTAAGTCAAAAAGCTTAAATTTTT-CCATAGGACGAGAAGACCCTATAGAA--TTTTACT--
------CTTGTTAAATGAATAATTTATTTTTGGT--ATTTT----AT-ATAATTTGTTTGTTTAA-
AGAGTTTTGTTGGGGTGACAGGGAAA-TTTCAATAACTTTCTTTATA--ATGTTT-TTATTTATGATTAT---
--ATTAATGATCCTTTA-----TTAAGGATA--ATAAGATTAAATTACCTTAGGGATAAC
Gonopsis_sp._affinis
ATGCAAGTCTCAGTACGAGCTGACT-
TAAAGTGAAACCGCAAAAGGCTCAGTAAACCGGTCATAGTTTACCGGA-TATATG-CCACTC-
ACGTGGATAACTGTGGTAATTCTAGAGCTAATACATGCAACC-AAGTCCCGACCAGCG---
ATGGAAAGGACGCTTTTATTGGATCAAAACCATTCGCCCGG-TTCGCCCGGGC------
GTCTGCTTGATGACTCT-AGATAAC-ACCACA-GATCGCACGGTCACAGTACCGGC-
GACGTATCCGTCAAATGTCTGACTTATCAGCTGTCGATGGTAGACTCTGTGTCTACCATGGCGCTCACGGGTA
ACGGGGAATCAGGGTTCGATTCCGGAGAGGGAGCCTGAGAAACGGCTACCACATCCAAGGAAGGCAGCAGGCA
CGCAAATTACCCACTCCCGGCACGGGGAGGTAGTGACAAAAAATAACGATACGGGACTCATCCGAGGCCCCGT
AATCGGAATGAGAACACCCTAAATCCTTTAACGAGGATCTATTGGAGGGCAAGTCTGGTGCCAGCAGCCGCGG
TAATTCCAGCTCCAATAGCGTATATTAAAGTTGTTGCGGTTAAAAA-
GCTCGTAGTTGGTTCTGCGTGCCACGCTGTCGGTTCACCGCCTGTCGGTGCAACTGGCATGCC-
GTGGCATGTCCTGTCGGTGGCTGACGGGC-TTCGCG-GCGCGCCCCACT-------CGCTCACG-CGTGT---
-GGGTCGACGC--GCCGT-GTTGTCC-TCG-TTGGCCGGCT-CAATCCTACCGCGGTGCT-
CTTAAACGAGTGTCGAGGTAGGCCGACACT-
GTTTACTTTGAACAAATTAGAGTGCTTCAAGCAGGCTCTGAAAGC-
AGCCTGAATAGTGGTGCATGGAATAATGGAACAGGACCTTGGTTCTATTTTGTT-GGTTTCCGGA-
ATCCAAGGTAATGATCAATAAGGA-CAGGCGGGGGCATTCGTATTGCGACGTTAGA-----------------
-GGTGAAATTCTTGG-

ATCGTCGCAAGACGCACTAGAGCGAAAGCATTTGCCAAGTATGTCTTAATTGATCAAGAACGAAAGTTAGAGG
TTCGAAGGCGATCAGATACCGCCCTAGTTCTAACCATAAACGATGCCAGCCAGCGATCCGCCGATGTTCCTCC
GATGACTCGGCGGGGAGCTT-
TCCCCGGGAAACCAAAGCTTTTGGGTTCCGGGGGAAGTATGGTTGCAAAGCTGAAACTTAAAGGAATTGACGG
AAGGGCACCACCAGGAGTGGAGCCTGCGGCTTAATTTGACTCAACACGGGAAACCTCACCAGGCCCGGACATT
GGAA-GGATTGACAGAT-CGATAGCTCTTT-CTCGATT-CAGTGGGT-
AGTGGTGCATGGCCGTTCTTAGTTGGTGGACTGATTTGTCTGGTTAATTCCGATAACGAACGAGACTCTGACC
TATTAACTAGGCGCTC-CCGGTATAA-ATA-CCTACCGGCGACAA-
TTATTCTTCTTAAGGGGACAGGCGGCTCTCAGCCGCACGAGATTGAGCAATAACAGGTCTGTGATGCCCTTAG
ATGTTCTGGGCCGCACGCGCGCTACACTGAAGGAATCAGCGTGTTC--
TCCCCTGTCCGAAAGGTCCGGGCAACCCGCTGAACCTCCTTCGTGCTAGGAATTGGGGCTTGCAATTGTTCCC
CATGAACGAGGAATTCCCAGTAAGCGCGAGTCATAAGCTCGCGTTGATTACGTCCCTGCCCTTTGTACACACC
GCCCGTCGCTACTACCGATTGAATGATTTAGTGAGGTCTTCGGACC-GGCGGTA--AC-CGGC-ACC--GG--
---CTCGTTCGG---TATCGGT-TTCGCTGGAAA-
GATGACCGAACTTGATCATTTAGAGGAAGTAAAAGTCGTAACAAGG-TTTCCAGTCA--TTGGGT--G-
TAAAACCTAAAGGCGCAATGAAAGT-GTCA-------------GGGGTGCCTCCTT------------CAGG-
-AG----CGCATCCCGTTGGGAGGATGGGC-CGCC---GTACGGCATGC-T------CGT--
CGGAGCCCCGCACTCCCGGGGCGTCTC--GTTCTCA-TCACG-AG----
TGGAGACGCACCAGGAGCGCACACGCTGGTACCCGAAAGATGGTGAACTATGCCTGGCCAGGACGAAGCCAGG
GGAAACCCTGGTGGAGGTCC--GAAGCG-ATTCTG--ACGTGCAAATCG-
ATCGTCTGAGCTGGGTATAGGGGCGAAAGACCAATCGAACCATCTG---TTTTGGTT---
TATTAAGTCTGGCCTGCTCAATGATTAT-------T---
ATTAAATAGCCGCAGTATTTTGACTGTGCGAAGGTAGCATAATCATTTGTCTTTTAATTGGAGACTTGAATGA
ATGGTTTTATGAGGGAACGCCTTTCTTTA---TACTAAAT-TT--
TGAATTTAATATTTGTGTTAAAAAGCGTAAATTTTT-AAATGGGACGAGAAGACCCTATAGAA--TTTTA-
TTTTATATTT----------AATTATT--TGATCTATTGGTTTAT----
TATTAGATATAATTTAATTTAGAATTTTGTTGGGGCGACATTGAGA-TTTTTTTTACTCTCA-
TATATTATTTAT-TTATTAATTTTTATAT-----TTAGGATCCTT-----TATTATGGATA--
TTTAGTTTAAATTACCTTAGGGATAAC
Graphosoma_italicum
ATGCAAGTCTCAGTACGAGCTGACT-
TAAAGTGAAACCGCAAAAGGCTCAGTAAACCGGTCATAGTTTACCGGA-TATATG-CCACTC-
ACGTGGATAACTGTGGTAATTCTGGAGCTAATACATGCAACC-AAGTCCCGACCAGCG---
ATGGAAAGGACGCTTTTATTGGATCAAAACCATTCGCCCGGCT-CGCCCGGGC------
GTCTGCTTGATGACTCT-AGATAAC-ACAACA-GATCGCACGGTCACAGTACCGGC-
GACGTATCCGTCAAATGTCTGACTTATCAGCTGTCGATGGTAGACTCTGTGTCTACCATGGCGCTCACGGGTA
ACGGGGAATCAGGGTTCGATTCCGGAGAGGGAGCCTGAGAAACGGCTACCACATCCAAGGAAGGCAGCAGGCA
CGCAAATTACCCACTCCCGGCACGGGGAGGTAGTGACAAAAAATAACGATACGGGACTCATCCGAGGCCCCGT
AATCGGAATGAGAACACCCTAAATCCTTTAACGAGGATCTATTGGAGGGCAAGTCTGGTGCCAGCAGCCGCGG
TAATTCCAGCTCCAATAGCGTATATTAAAGTTGTTGCGGTTAAAAA-
GCTCGTAGTTGGTTCTGCGTGCCACGCTGTCGGTTCACCGCCTGTCGGTGCAACTGGCATGCC-
GTGGCATGTCCTGTCGGTGGCTGACGGGC-TTCGCG-GCGCGCCCCACT-------CGCTCACG-CGTGT---
-GGGTCGACGC--GCCGT-GTCGTCC-TCG-TTGGCCGGCT-CAATCCTACCGCGGTGCT-
CTTAAACGAGTGTCGAGGTAGGCCGACACT-
GTTTACTTTGAACAAATTAGAGTGCTTCAAGCAGGCTCTGAAAGC-
AGCCTGAATAGTGGTGCATGGAATAATGGAACAGGACCTTGGTTCTATTTTGTT-GGTTTCCGGA-
ATCCAAGGTAATGATCAATAAGGA-CAGGCGGGGGCATTCGTATTGCGACGTTAGA-----------------
-GGTGAAATTCTTGG-
ATCGTCGCAAGACGCACTAGAGCGAAAGCATTTGCCAAGTATGTCTTAATTGATCAAGAACGAAAGTTAGAGG
TTCGAAGGCGATCAGATACCGCCCTAGTTCTAACCATAAACGATGCCAGCCAGCGATCCGCCGATGTTCCTCC
GATGACTCGGCGGGGAGCTT-
TCCCCGGGAAACCAAAGCTTTTGGGTTCCGGGGGAAGTATGGTTGCAAAGCTGAAACTTAAAGGAATTGACGG
AAGGGCACCACCAGGAGTGGAGCCTGCGGCTTAATTTGACTCAACACGGGAAACCTCACCAGGCCCGGACATT
GGAA-GGATTGACAGAT-CGATAGCTCTTT-CTCGATT-CAGTGGGT-
AGTGGTGCATGGCCGTTCTTAGTTGGTGGACTGATTTGTCTGGTTAATTCCGATAACGAACGAGACTCTGACC
TATTAACTAGGCGCTC-CCGGTATAC-ATA-CCTACCGGCGAGAT-
TTATTCTTCTTAAAGGGACAGGCGGCTCTTAGCCGCACGAGATTGAGCAATAACAGGTCTGTGATGCCCTTAG
ATGTTCTGGGCCGCACGCGCGCTACACTGAAGGAATCAGCGTGTGC--

TCCCCTGTCCGAAAGGACCGGGCAACCCGCTGAACCTCCTTCGTGCTAGGAATTGGGGCTTGCAATTGTTCCC
CATGAACGAGGAATTCCCAGTAAGCGCGAGTCATAAGCTCGCGTTGATTACGTCCCTGCCCTTTGTACACACC
GCCCGTCGCTACTACCGATTGAATGATTTAGTGAGGTCTTCGGACC-GGCGGTA--AC-CGGC-ACC--GG--
---CTCGTTCGG---TATCGGT-TTCGCTGGAAA-
GATGACCGAACTTGATCATTTAGAGGAAGTAAAAGTCGTAACAAGG-TTTCCAGTCA--TTGGGT--G-
TAAA-CCTAAAGGCGCAATGAAAGT-GTCA-------------GGGGTGCCTCCTT------------TAGG-
-AG----CGCATCCCGCTGGGAGGATGGGC-CGCC---GTACGATCA-C-T------CGT--
CGGAGCCCCGCACTCCCGGGGCGTCTC--GTTCTCA-TCACG-AG----
TGGAGACGCACCAGGAGCGCACACGCTGGTACCCGAAAGATGGTGAACTATGCCTGGCCAGGACGAAGCCAGG
GGAAACCCTGGTGGAGGTCC--GAAGCG-ATTCTG--ACGTGCAAATCG-
ATCGTCTGAGCTGGGTATAGGGGCGAAAGACCAATCGAACCATCTG---TTTTTTTA--
TTATTAAGTCTGGCCTGCTCAATGATTTT-----------
ATTAAATAGCCGCAGTATTTTGACTGTGCGAAGGTAGCATAATCATTTGTCTTTTAATTGAAGGCTTGTATGA
ATGGTCGGATGAAAAAATGACTTTCTTTA---CATTAAAT-GT--
TGAATTTAATTTTTTGGTTAAAAAGCTAAAATAATT-GAATGGACGAGAAGACCCTATAGAA--TTTTA--
TTAAATTGT----------AGTTTTG---AATTTATTGGTTAAT----
TATTATTTTTAATTATAATTTTAATTTTGTTGGGGTGACATTGAGA-
TTTATTTAACTCTCATAATATAAAGT---TTATTGATTTATATAT-----AAAAGATCCTA-----
TTTTATGGATT--AATAGATTAAATTACCTTAGGGATAAC
Himacerus_mirmicoides
ATGCAAGTCTCAGTACAGACTGTAT-
TAAAGTGAAACCGCAAAAGGCTCAGTAAACCAGTTTTGATTCACTTGAGTAAACT-
TGACTGTCTTTGGATAACTGTGGTAATTCTAGAGCTAATACATGCAACA-AAGCCCTGACCTCCC---
ATGGAAAGGGCGCTTTTATTAGATCAAAACCATTCGCCCGGCT-CGCCCGGGC------
GTTGGCTTGATGAACCT-AGATAAC-AAATCA-GATCGTATGGTCACCGAACCGAC-
GACTTATCTTTCAAATGCCTGACTTATCAACTGTCGATGGTAGACTCTGTGTCTACCATGGCTCCTACGGGTA
ACGGGGAATCAGGGTTCGATTCCGGAGAGGGAGCCTGAGAAACGGCTACCACATCCAAGGAAGGCAGCAGGCA
CGCAAATTACCCACTCCCGGCACGGGGAGGTAGTGACAAAAAATAACGATACGGGACTCTTATGAGGCCTCGT
AATCGGAATGAGTACACTTTAAATCCTTTAACAAGGATCCATTGGAGGGCAAGTCTGGTGCCAGCAGCCGCGG
TAATTCCAGCTCCAATAGCGTATATTAAAGTTGTTGCGGTTAAAAA-
GCTCGTAGTTGGTTCTGCGTGCCACGCTGTCGGTACACCGTTTGTCGGTGCAACTGGCATGCC-
GTGGCATGTCCTGTCGGTGGTGACCAGGT-TCCCGGTGTG-------------------CCGAAGTTTCT---
GAAGTACCCG----CGGAC----TC--CCGGTTG-CCGACT-ATATCCTACCGCGGTGCT-
CTTAGTTGAGTGTCGAGGTAGGCCGACAC--GTTTACTTTGAACAAATTAGAGTGCTTAAAGCAG-
CCAAAATCTC-TGCCTGAATAGTGGTGCATGGAATGATAGAACAGGACCTTGGTTCTATTTTATTGGT-
TTTCGGA-ACACGAGGTAATGATCAATGTGGA-CAGGCGGGGGCATTCGTATTGCGACGTTAGA---------
---------GGTGAAATTCTTGG-ATCGTCGCA-
GACGCACTAGAGCGACAGCATTTGCCAAGTATGTCTTAATTGATCAAGAACGAAAGTTAGAGGTTCGAAGGCG
ATCAGATACCGCCCTAGTTCTAACCATAAACGATGCCAGCCAGCGATCCGCCGATGTTCCTCCGATGACTCGG
CGGGGAGCTT-
TCCCCGGGAAACCAAAGCTTTTGGGTTCCGGGGGAAGTATGGTTGCAAAGCTGAAACTTAAAGGAATTGACGG
AAGGGCACCACCAGGAGTGGAGCCTGCGGCTTAATTTGACTCAACACGGGAAACCTCACCAGGTCAGGACATT
GGAA-GGATTGACAGAT-TGATAGCTCTTT-CTTGATT-CAGTGGGT-
AGTGGTGCATGGCCGTTCTTAGTTGGTGGATCGATTTGTCTGGTTAATCGACGAAACGAACGAGACTCTAGCC
TATTAACTAGGCGTTT-CTGGTATCG---TAGCTACCAGCGAAAC-
ATATTCTTCTTAAGGGGACAGGCGGCTTATAGCCGCACGAGATTGAGCAATAACAGGTCTGTGATGCCCTTAG
ATGTCCTGGGCCGCACGCGCGCTACACTGAAGGAATCAGCGTGTGAGTTCCCTTGGCCGAGAGGTCCGGGTAA
CCCGCTGAACCTCCTTCGTGCTAGGAATTGGGGCTTGCAATTGTTCCCCATGAACGAGGAATTCCCAGTAAGC
GCGAGTCATAAGCTCGCGTTGATTACGTCCCTGCCCTTTGTACACACCGCCCGTCGCTACTACCGATTGAATG
ATTTAGTGAGGTCTTCGGACT-GGCGGTAAGCCTTGCCGACT----------TGT--CGG---
TTTTGCTGAACGCTGGAAA-GATGACCAAACTTGATCATTTAGAGGAAGTAAA-GTCGTAACAAGG-
TTTCCAGTCA--TTGGGTG-----AAACCTAAAGGCGGAATGAAAGT-----------------
AAAGGAAACTAGCT-----------------------------TTCTGAGGGAGGATGGGTTAC--------
CTGCCTTTACAGGCGGTG--------CTCCGCACTCCCGGGGCGTCTC--TTTCTC-ATTACG-AG----
TGGAGGCGCACCTTTAGCGTACACGCTGATACCCGAAAGATGGTGAACTATGCCTGGCCAGGACGAAGCCAGG
GGAAACCCTGGTGGAGGTCC--GCAGCG-ATTCTG--ACGTGCAAATCG-
ATCGTCGGAGCTTGGTATAGGGGCGAAAGACCAATCGAACCATCTGA----
GTTTATTAATATGAGGTCTGACCTGCCCTATGATTTAT------

ATGAATTAAATGGCCGCAGTATTCTAACTGTGCGAAGGTAGCATAATCATTTGTCCTTTAATTGGGGACTTGT
ATGAATGGTCTGATGAGGGATAAACTTTCTTTATTTTATTTTTAAT-----
AATTTAATTTATAAGTCAAAAAGCTTATATATTA-TTAAGTGACGAGAAGACCCTATAGAA--
CTTTACTATT---------------TATAATGATACTGTTATTTTGTTTAT---
TAATTGATAGTATTTTTATTAGTAGTTTTATTGGGGCGATAGTTAAATTTAATTAACTTAAACTTGATTAAAT
T---TCATTAATTAATGATTA--ATTATTGATCCAT-----TTTTATTGATT--
AAAAGATCAAGTTACCTTAGGGATAAC
Isodermus_planus_Libiocoris_heissi
ATGCAAGTCTCAGTACAAACTGATT-
TAAAGTGAAACCGCAGAAGGCTCAGTAAACCGGTCATGGTTCAAATGA-ACCCTT-TAATTC-
ATTTGGATAACTGTGGTAATTCTAGAGCTAATACATGCAACT-AAGTCCTGACCAGCG---
ATGGAAAGGACGCTTTTATTGGATCAAAACCATCCACCTGGCTTGCT--GGGT------
GTTGGCTTGATGACTCT-AGATAAC-ACCACG-GATCGCACGGTCTCTGAACCGGC-
GATGTATCTTTCAAATGTCTGACTTATCAGCCGTCGATGGTAGACTCTATGTCTACCATGGCGCTCACGGGTA
ACGGGGAATCAGGGTTCGATTCCGGAGAGGGAGCCTGAGAAACGGCTACCACATCCAAGGAAGGCAGCAGGCA
CGCAAATTACCCACTCCCAGCACGGGGAGGTAGTGACAATAAATAACGATACGGGACTCATCCGAGGCCCCGT
AATCGGAATGAGCACACTTTAAATCCTTTAACAAAAATCTATTGGAGGGCAAGTCTGGTGCCAGCAGCCGCGA
TAATTCCAGCTCCAATAGCGTATATTAAAGTTGTTGCGGTTAAAAA-
GCTCGTAGTTGGTTCTGCGTGCCATGCTGTCGGTTCACCGCCTGCCGGTGTAACTGACATGTC-
ATGGCATGTCTTGTCGGTGGTGGATCAAGGTCCTTTGGTGCATCATGCTACTACC-TGGCAGCAACAGCTAA-
GGGGTAGCTGTGTGCCTTGGTTGATCCTTGTCTG-CCTATT-CTATCCTACCACGGTGCT-
CTTTACCGAGTGTCGAGGTGGGCCGACAAT-
GTTTACTTTGAACAAATTAGAGTGCTCAAAGCAGGCTCTAACATT-
TGCCTAAATAGTGGTGCATGGAATAATAGAATAGGACCTTGGTTCTATTTTGTT-GGTTTTCGGA-
TCCCAAGGTAATGATCAATAAGGA-CAGGCGGGGGCATTCGTATTGCGACGTTAGA-----------------
-GGTGAAATTCTTGG-
ATCGTCGCAAGACGCACTAGAGCGAAAGCATTTGCCAAGTATGTCTTAATTGATCAAGAACGAAAGTTAGAGG
TTCGAAGGCGATCAGATACCGCCCTAGTTCTAACCATAAACGATGCCAGCCAGCGATCCGTCGATGTTCCTCC
GATGACTCGACGGGGAGCTT-
TCCCCGGGAAACCAAAGCTTTTGGGTTCCGGGGGAAGTATGGTTGCAAAGCTGAAACTTAAAGGAATTGACGG
AAGGGCACCAC-
AGGAGTGGGGCCTGCGGCTTAATTTGACTCAACACGGGGAACCTCACCAGGTCCGGACTTCGGAA-
GGATTGACAGAT-CGATAGCTCTTT-CTCGATT-CGACGGGT-
AGTGGTGCATGGCCGTTCTTAGTTGGTAGACTGATTTGTCTGGTTAATTCCGATAACGAACGAGACTCTAGCC
TATTAACTAGGCGTCT-CCGGTATACTATA-TCTACCGGCGAGAT-
TTATTCTTCTTAAGGGGACAGGCGGCTCATAGCCGCACGAGATTGAGCAATAACAGGTCAGTGATGCCCTTAG
ATGTTCTGGGCCGCACGCGCGCCACACTGAAGGAATCAGCGTGTTTTATCCCCTGTTCGAAAGGACCGGGTAA
CCCGCTAAACCTCCTTCGTGCTAGGAATTGGGGCTTGCAATTGTTCCCCATGAACGAGGAATTCCCAGTAAGC
ACGAGTCATAAGCTCGTGTTGATTACGTCCCTGCCCTTTGTACACACCGCCCGTCGCTACTACCGATTGAATG
ATTTAGTGAGGTCTTCAGACC-GGTGGTG--GT-CGGC-GCT--GACA-----TGTTCAG---TATCGGC-
TCCGCTGGAAA-GATGACCAAACTTGATTATTTAGAGGAAGTAAAAGTCGTAACAAGG-TTTTCAGTCAA--
TAGGTGT---AAAACCTAAAGGCGAAATGAAAGT-GTAACA------------GTATAAATTTTA--------
------AATAATT---TTTATTGTGATGGGAGGACAAG---------GTATTGTTAGTTA------
AATGGCATACCTTCGCACTCCCGGGGCGTTTT--ATTCTC-ATTGCG-AG----
TTAAAACGCACCAGGAGCGTACACGCTGGTACCCGAAAGATGGTGAACTATGCCTGATCAGGACGAAGCCAGG
GGAAACCCTGGTGGAGGTCC--GAAGCG-ATTCTG--ACGTGCAAATCG-
ATCGTCTGAGTTGGGTATAGGGGCGAAAGACCAATCGAACCATCTGG-----TTTTT---
TTTTAAATCTCGCCTGCTCAATGATTATT-----------
TTAAATAGCTGCAGTATTTTGACTGTACAAAGGTAGCATAATAATTTGTCTTTTAATTGAAGGCTTGAATGAA
TGGTAGGACGAGGGGCAAGCTTTCTTTAAACTATTTTATT-----
GAATTTAATTTTTTAGTTAAAAAGCTGAGATTTTA-TTGTAGGACGAGAAGACCCTGTAGAA--TTTTACCT-
----------------CTATTTGTTTTTATTATTTTATTTTA----
AATGTTATTTTAATGAAGGTTAGGTTTTGTTGGGGAGACAGAGTTT-
TTTTATTAACGAACTTTTGTTTATTT---TCATTGATTATTGTTG----TTAATGATCCTT-----
TATTATGGATT--GTAAGATTAAATTACCTCAGGGATAAC
Katakadia_caliginosa
ATGCAAGTCTCAGTACGAGCTGACT-
TAAAGTGAAACCGCAAAAGGCTCAGTAAACCGGTCAAAGTTTACCGGA-TATATG-CCACTC-

ACGTGGATAACTGTGGTAATTCTAGAGCTAATACATGCAACC-AAGTCCCGACCAGCG---
ATGGAAAGGACGCTTTTATTGGATCAAAACCATTCGCCCGGCTCGCCC-GGGC------
GTCTGCTTGATGACTCT-AGATAAC-ACCACA-GATCGCACGGTCATAGTACCGGC-
GACGTATCCGTCAAATGTCTGACTTATCAGCTGTCGATGGTAGACTCTGTGTCTACCATGGCGCTCACGGGTA
ACGGGGAATCAGGGTTCGATTCCGGAGAGGGAGCCTGAGAAACGGCTACCACATCCAAGGAAGGCAGCAGGCA
CGCAAATTACCCACTCCCGGCACGGGGAGGTAGTGACAAAAAATAACGATACGGGACTCATCCGAGGCCCCGT
AATCGGAATGAGAACACCCTAAATCCTTTAACGAGGATCTATTGGAGGGCAAGTCTGGTGCCAGCAGCCGCGG
TAATTCCAGCTCCAATAGCGTATATTAAAGTTGTTGCGGTTAAAAA-
GCTCGTAGTTGGTTCTGCGTGCCACGCTGTCGGTTCACCGCCTGTCGGTGCAACTGGCATGCC-
GTGGCATGTCCTGTCGGTGGCTGACAGGC-TTCGCG-GCGCGCCCTATG-------CGCTCAC-ACGTGT---
-AGGTCGTTGC--GCCG-CGTTGTCC-TTGTAGG-CCGGCT-CAATCCTACCGCGGTGCT-
CTTTGCCGAGTGTCGAGGTAGGCCGACACT-
GTTTACTTTGAACAAATTAGAGTGCTTCAAGCAGGCTCTGAAAGC-
AGCCTGAATAGTGGTGCATGGAATAATGGAACAGGACCTTGGTTCTATTTTGTT-GGTTTACGGA-
ATCCAAGGTAATGATCAATAAGGA-CAGGCGGGGGCATTCGTATTGCGACGTTAGA-----------------
-GGTGAAATTCTTGG-
ATCGTCGCAAGACGCACAAGAGCGAAAGCATTTGCCAAGTATGTCTTAATTGATCAAGAACGAAAGTTAGAGG
TTCGAAGGCGATCAGATACCGCCCTAGTTCTAACCATAAACGATGCCAGCCAGCGATCCGCCGATGTTCCTCC
GATGACTCGGCGGGGAGCTT-
TCCCCGGGAAACCAAAGCTTTTGGGTTCCGGGGGAAGTATGGTTGCAAAACTGAAACTTAAAGGAATTGACGG
AAGGGCACCACCAGGAGTGGAGCCTGCGGCTTAATTTGACTCAACACGGGAAACCTCACCAGGCCCGGACATT
GGAA-GGATTGACAGAT-CGATAGCTCTTT-CTCGATT-CAGTGGGT-
AGTGGTGCATGGCCGTTCTAACTTGGTGGGCTGATTTGTCTGGTTAATTCCGATAACGAACGAGACTATGACC
TATTAACTAGGCGCTC-CCGGTATAA-ATA-CCTACCGGCGAGAT-
TTATTCTTCTTAAGGGGACAGGCGGCTCTTAGCCGCACGAGATTGAGCAATAACAGGTCTGTGATGCCCTTAG
ATGTTCTGGGCCGCACGCGCGCTACACTGAAGGAATCAGCGTGTGC--
TCCCCTGTCCGAAAGGACCGGGCAACCCGCTGAACCTCCTTCGTGCTAGGAATTGGGGCTTGCAATTGTTCCC
CATGAACGAGGAATTCCCAGTAAGCGCGAGTCATAAGCTCGCGTTGATTACGTCCCTGCCCTTTGTACACACC
GCCCGTCGCTACTACCGATTGAATGATTTAGTGAGGTCTTCGGACC-GGCGCTA--AC-CGGC-ACC--GGC-
----TCGTTCGG---TATCGGT-TTCGCTGGAAA-
GATGACCGAACTTGATCATTTAGAGGAAGTAAAAGTCGTAACAAGG-TTTCCAGTCA--TTGGGTGT---
AAAACCTAAAGGCGCAATGAAAGT-GTCAG-------------GGGGGCCTCCTT-------------
TAGGAGC-----GCTCTCCGCAGGGAGGATGGGCGACC----GTACGATCACT--------TGT--
CGGAGCCTCGCACTCCCGGGGCGTCTC--GTTCTC-GTCACG-AG----
TGGAGACGCACCAGGAGCGCACACGCTGGTACCCGAAAGATGGTGAACTATGCCTGGCCAGGACGAAGCCAGG
GGAAACCCTGGTGGAGGTCC--GAAGCG-ATTCTG--ACGTGCAAATCG-
ATCGTCTGAGCTGGGTATAGGGGCGAAAGACCAATCGGACCATCTGA-GTTTAATTT--
ATATTAGGTCTTACCTGCTCAATGATA--------
TTTTTATTAAATAGCCGCAGTATTTTGACTGTGCAAAGGTAGCATAATCAATTGTCTTTTAATTGTAGGCTTG
TATGAATGGTTTGACGAGATATTATCTTTCTTTGTGTTATTAT--T---
TTGAATTTGATTATTGAGTTAAAAAGCTTAAATGTTT-TTATAGGACGAGAAGACCCTATAGAA--
ATTTATTAAT----TATTGTTTATTTCT------CTGTAATGGTTTTTGA-------
TTTGGTGGTTTACATTATTA-ATTGTGTTGGGGTGACAGGGAAA-TTTTATTAACTTTCTTTAAT-
TATTTAA-TTATTGATGAGTAAAT--TTTTAATGATCCTATT---TT--ATGGATA--
TTAAGATTAATTTACCTTAGGGATAAC
Kleidocerys_sp._resedae
ATGCAAGTCTCAGTACGAGCTTACT-
TAAAGTGAAACCGCAAAAGGCTCAGTAAACCGGTCATAGTTTACCGGA-TAAATG-CCACTC-
ACGTGGATAACTGTGGTAATTCTAGAGCTAATACATGAAAAC-AAGTCCCGACCAGCG---
ATGGAGAGGACGCTTTTATTTGATCAAAACCATTCGCTCGGCTTG-CC-GGGC------
GTCGGCTTGATGATTCT-AGATAAC-ACCACA-GATCGCACGGTCTCTGTACCGGT-
GACATATCCTTCAAATGTCTGACTTATCAGCTGTCGATGGTAGACTCTGTGTCTACCATGGCGCTCACGGGTG
ACGGGGAATCAGGGTTCGATTCCGGAGAGGGAGCCTGAGAAACGGCTACCACATCCAAGGAAGGCAGCAGGCA
CGCAAATTACCCACTCCCGGCACGGGGAGGTAGTGACAAAAAATAACGATACGGGACTCATCCGAGGCCCCGT
AATCGGAATGAGAACACCCTAAATCCTTTAGCGATGATCTATTGGAGGGTAAGCCTGGTGCCAGCAACCGCGG
TAACTCCAGCTCCAATAGCGTATATTAAAGTTGTTGCGGTTAAAAA-
GCTCGTAGTTGGTTCTGCGTGCCACGCTGTCGGTTCACCGCCTGCCGGTGCAACTGGCATGCC-
GTGGCATGTCCTGTCGGTGGCGGATAGGC-TTTGCG-GTGCGCGCTATGCGG----TAT-ACCAGCGCGT---

-AGACTGCCGT--ACCGCTT-TGTCC-TTGTACG-CCGACT-CAATCCTACCGCGGTGCT-
CTTCACCGAGTGTCGAGGTAGGCCGACACT-
GTTTACTTTGAACAAATTAGAGTGCTTCAAGCAGGCTCTTAAATC-
AGCCTGAATAATGGTGCATGGAATAATAGAACAGGACCTTGGTTCTATTTTGTT-GGTTTTCGGA-
ATCCAAGGTAATGATCAATAAGGA-
CAGGCGGGGGCATTCGTATTGCGACGTTAGACTTGGCAAATGCTTTCGCGGTGAAATTCTTGG-
ATCGTCGCAAGACGCACTAAAGCGAAAGCATTTGCCAAGTATGTCTTAATTGATCAAGAACGAAAGTTAGAGG
CTCGAAGGCGATCAGATACCGCCCTAGTTCTAACCATAAACGATGCCAGCCAGCGATCCGCCGACGTTCCTCC
GATGACTCGGCGGGGAGCTT-
TCCCCGGGAAACCAAAGCTTTTGGGTTCCGGGGGAAGTATGGTTGCAAAGCTGAAACTTAAAGGAATTGACGG
AAGGGCACCACCAGGACTGGAGCCTGCGGCTTAATTTGACTCAACACGGGAAACCTCACCGGGCCCGGACATT
GGAA-GGATTGACAGAT-CGATAGCTCTTT-CTCGATT-CAGTGGGT-
AGTGGTGCATGGCCGTTCTTAGTTGGTGGACTGATTTGTCTGGTTAATTCCGATAACGAACGAGACTCTGACC
TACTAACTAGGCGACA-CCGGTATAAAATA-GCTACCGGCGAGAT-
TTATTCTTCTTAAGGGGACAGGCGGCTCTTAGCCGCACGAGATTGAGCAATAACAGGTCAGTGATGCCCTTAG
ATGTTCTGGGCCGCACGCGCGCTACACTGAAGGAATCAGCGTGTGC--
TCCCCTGTCCGAAAGGTCCGGGTAACCCGCTGAACCTCCTTCGTGCTAGGAATTGGGGCTTGCAATTGTTCCC
CATGAACGAGGAATTCCCAGTAAGCGCGAGTCATAAACTCGCGTTGATTACGTCCCTGCCCTTTGTACACACC
GCCCGTCGCTACTACCGATTGAATGATTTAGTGAGGTCTTCGGACC-GGCGATA--AC-CGGC-ACC--
GGCTC-----GTCCGG---TATCGAT-TTCGCTGGAAA-
GATGACCGAACTTGATCATTTAGAGGAAGTAAAAGTCGTAACAAGG-TCTCCAGTCA--TTGGGTGT---
CAAACCTAAAGGCGCAATGAAAGT-GTCAGA--------------GGGGCGCTCA----------------
GCGCT-------CCTCGGAGGGAGGATGGGCTC-CC---GGCCCCCCGGC-------------
CGGTGCCCCGCACTCCCGGGGCGTTTC--ATTCTCGTTTACG-AG----
TCGAGACGCACCAGGAGCGCACACGCTGTTACCCGAAAGATGGTGAACTATGCCTGGCCAGGACGAAGCCAGG
GGAAACCCTGGTGGAGGTCC--GAAGCG-ATTCTG--ACGTGCAAATCG-
ATCGTCTGAGCTGGGTATAGGGGCGAAAGACCAATCGAACTATCTAG----ATTA-
TTAATTTTAGGTCTATCCTGCTCAGTGATTTATT----------
TTAAATAGCCGCAGTATTTTGACTGTGCAAAGGTAGCATAATCATTTGTCTTTTAATTGAAGGCTTGTATGAA
TGGTTGGACGAGATATTAACTTTCTTTATTTAA-TTTTTA----
TGAATTTAATTTTTTAGTGAAAAAGCTTAAATTATT-TTGTGGGACGAGAAGACCCTATAGAA--TTTAAGA-
-------------TTAATTTATTATATTTAA----TTAATTATT---
TGATTATTTTATATTATTCTTAATTTTTGGTTGGGGTGATTATAAAA-TTTTCTTAACTTTTATATTTTAT-
TT---TCATTGATTAGTGTTTT----TATTGATCCTTTA---TTAA--GGATT--
ATTAGATTAAATTACCTTAGGGATAAC
Lactistes_vericulatus
ACCAAAATTTGAGTACGAGCTAACT-
TAAAGTGAAACCGCAAAATGCTCAGTAAACCGGTCATAGTTTACCGGA-TATATG-CCACTC-
ACGTGGATAACTGTGGTAATTCTAGAGCTAATACATGCAACC-AAGTCCCGACCAGCG---
ATGGAAAGGACGCTTTTATTGGATCAAAACCATTCGCCCGGCTCGCCC-GGGC------
GTTTGCTTGATGACTCT-AGATAAC-ACCACA-GATCGCACGGTCACAGTACCGGC-
GACGTATCCTTCAAATGTCTGACTTATCAGCTGTCGATGGTAGACTCTGTGTCTACCATGGCGCTCACGGGTA
ACGGGGAATCAGGGTTCGATTCCGGAGAGGGAGCCTGAGAAACGGCTACCACATCCAAGGAAGGCAGCAGGCA
CGCAAATTACCCACTCCCGGCACGGGGAGGTAGTGACAAAAAATAACGATACGGGACTCATCCGAGGCCCCGT
AATCGGAATGAGAACACCCTAAATCCTTTAACGAGGATCTATTGGAGGGCAAGTCTGGTGCCAGCAGCCGCGG
TAATTCCAGCTCCAATAGCGTATATTAAAGTTGTTGCGGTTAAAAA-
GCTCGTAGTTGGTTCTGCGTGCCACGCTGTCGGTTCACCGCCTGTCGGTGCAACTGGCATGCC-
GTGGCATGTCCTGTCGGTGGCTGACAGGC-TTCGCG-GCGCGCCCTATG-------CGTTTAG-GCGTGT---
-AGGTCGTCGC--GCCGACGTTGTCC-TTGTAGG-CCGGCT-CAATCCTACCGCGGTGCT-
CTTTGCCGAGTGTCGGGGTAGGCCGACACT-
GTTTACTTTGAACAAATTAGAGTGCTTCAAGCAGGCTCTGAAAGC-
AGCCTGAATAGTGGTGCATGGAATAATGGAACAGGACCTTGGTTCTATTTTGTT-GGTTTTCGGA-
ATCCAAGGTAATGATCAATAAGGA-CAGGCGGGGGCATTCGTATTGCGACGTTAGA-----------------
-GGTGAAATTCTTGG-
ATCGTCGCAAGACGCACTAGAGCGAAAGCATTTGCCAAGTATGTCTTAATTGATCAAGAACGAAAGTTAGAGG
TTCGAAGGCGATCAGATACCGCCCTAGTTCTAACCATAAACGATGCCAGCCAGCGATCCGCCGATGTTCCTCC
GATGACTCGGCGGGGAGCTT-
TCCCCGGGAAACCAAAGCTTTTGGGTTCCGGGGGAAGTATGGTTGCAAAGCTGAAACTTAAAGGAATTGACGG

AAGGGCACCACCAGGAGTGGAGCCTGCGGCTTAATTTGACTCAACACGGGAAACCTCACCAGGCCCGGACATT
GGAA-GGATTGACAGAT-CGATAGCTCTTT-CTCGATT-CAGTGGGT-
AGTGGTGCATGGCCGTTCTTAGTTGGTGGACTAATTTGTCTGGTTAATTCCGATAACGAACGAGACTCTGACC
TATTAACTAGGCGCTC-CCGGTATAT-ATA-CCTACCGGCGAGAT-
TTATTCTTCTTAAGGGGACAGGCGGCTCTTAGCCGCACGAGATTGAGCAATAACAGGTCTGTGATGCCCTTAG
ATGTTCTGGGCCGCACGCGCGCTACACTGAAGGAATCAGCGTGTGC--
TCCCCTGGCCGAAAGGTCCGGGCAACCCGCTGAACCTCCTTCGTGCTAGGAATTGGGGCTTGCAATTGTTCCC
CATGAACGAGGAATTCCCAGTAAGCGCGAGTCATAAGCTCGCGTTGATTACGTCCCTGCCCTTTGTACACACC
GCCCGTCGCTACTATAGATTGAATGATTTAGTGAGGTCTTCGGACC-GGCGGTA--AC-CGGC-ACC--GGC-
----TCGTTCGG---TATCGGT-TTCGCTGGAAA-
GATGACCGAACTTGATCATTTAGATAAAGTAAAAGTCGTAACAAGG-TTTCCAGTCA--TTGGGGGA---
AAA-CCTAAAGGCGCAATGAAAGT-GTCAG-------------GTGAGACTCCTT-------------
TAGGAGC-----TCTCGCCGGAGGGAGGATGGGTTACC----GTACATTTATT--------TGT--
CGGAGCCCCGCACTCCCGGGGCGTCTC--GTTCTC-ATTACG-AG----
TGGAGACGCACCAGGAGCGCACACGCCGGTACCCGAAAGATGGTGAACTATGCCTGGCCAGGACGAAGCCAGG
GGAAACCCTGGTGGAGGTCC--GAAGCG-ATTCTG--ACGTGCAAATCG-
ATCGTCTGAGCTGGGTATAGGGGCGAAAGACCAATCGAACCATCTTG-ATTAAATAT--
ATATCAGGTCAAGTCTGCTCAATGATTT-------
ATTTAATTTAATAGCCGCAGTAATTTGACTGTGCGAAGGTAGCATAATCATTTGTCTATTAATTGTAGACTTG
TATGAAGGGCTTAATGAAATACTAACTTTCTTTTTTTTATATTGAT-----
GAATTTAATGTTTGAGTTAAAAGGCTTAAATTTAT-TTATGGGACGAGAAGACCCTATAGAA--TTTTATT--
------ATAATTTTA--TTTATTTATTTATTGTG--TTTTT----ATAATAAATTGGTTTTGTT--
ATAATTTTGTTGGGGCGACAGGGAAA-TTTTTTCAACTTTCTTTATT-ATTTATT-TTAGTGATTATTATT--
-TTTGATTGATCCTTTA---TT--ATGGATA--GTAAGATTAAATTACCTTAGGGATAAC
Lactistes_obesipes
ACCAAAATTTGAGTACGAGCTAACT-
TAAAGTGAAACCGCAAAATGCTCAGTAAACCGGTCATAGTTTACCGGA-TATATG-CCACTC-
ACGTGGATAACTGTGGTAATTCTAGAGCTAATACATGCAACC-AAGTCCCGACCAGCG---
ATGGAAAGGACGCTTTTATTGGATCAAAACCATTCGCCCGGCTCGCCC-GGGC------
GTTTGCTTGATGACTCT-AGATAAC-ACCACA-GATCGCACGGTCACAGTACCGGC-
GACGTATCCTTCAAATGTCTGACTGATCAGCTGTCGATGGTAGACTCTGTGTCTACCATGGCGCTCACGGGTA
ACGGGGAATCAGGGTTCGATTCCGGAGAGGGAGCCTGAGAAACGGCTACCACATCCAAGGAAGGCAGCAGGCA
CGCAAATTACCCACTCCCGGCACGGGGAGGTAGTGACAAAAAAATAACGATACGGGACTCATCCGAGGCCCCGT
AATCGGAATGAGAACACCCTAAATCCTTTAACGAGGATCTATTGGAGGGCAAGTCTGGTGCCAGCAGCCGCGG
TAATTCCAGCTCCAATAGCGTATATTAAAGTTGTTGCGGTTAAAAA-
GCTCGTAGTTGGTTCTGCGTGCCACGCTGTCGGTTCACCGCCTGTCGGGGCAACTGGCATGCC-
GTGGCATGTCCTGTCGGTGGCTGACAGGC-TTCGCG-GCGCGCCCTATG-------CGTTTAC-GCGTGT---
-AGGTCGTCGC--GCCGACGTTGTCC-TTGTAGG-CCGGCT-CAATCCTACCGCGGTGCT-
CTTTGCCGAGTGTCGGGGTAGGCCGACACT-
GTTTACTTTGAACAAATTAGAGTGCTTCAAGCAGGCTCTGAAAGC-
AGCCTGAATAGTGGTGCATGGAATAATGGAACAGGACCTTGGTTCTATTTTGTT-GGTTTTCGGA-
ATCCAAGGTAATGATCAATAAGGA-CAGGCGGGGGCATTCGTATTGCGACGTTAGA-----------------
-GGTGAAATTCTTGG-
ATCGTCGCAAGACGCACTAGAGCGAAAGCATTTGCCAAGTATGTCTTAATTGATCAAGAACGAAAGTTAGAGG
TTCGAAGGCGATCAGATACCGCCCTAGTTCTAACCATAAACGATGCCAGCCAGCGATCCGCCGATGTTCCTCC
GATGACTCGGCGGGGAGCTT-
TCCCCGGGAAACCAAAGCTTTTGGGTTCCGGGGGAAGTATGGTTGCAAAGCTGAAACTTAAAGGAATTGACGG
AAGGGCACCACCAGGAGTGGAGCCTGCGGCTTAATTTGACTCAACACGGGAAACCTCACCAGGCCCGGACATT
GGAA-GGATTGACAGAT-CGATAGCTCTTT-CTCGATT-CAGTGGGT-
AGTGGTGCATGGCCGTTCTTAGTTGGTGGACTGATTTGTCTGGTTCATACAAAAAACGAACGAGACTCTGACC
TATTAACTAGGCGCTC-CCGGTATAT-ATA-CCTACCGGCGAGAT-
TTATTCTTCTTAAGGGGACAGGCGGCTCTTAGCCGCACGAGATTGAGCAATAACAGGTCTGTGATGCCCTTAG
ATGTTCTGGGCCGCACGCGCGCTACACTGAAGGAATCAGCGTGTGC--
TCCCCTGGCCGAAAGGTCCGGGCAACCCGCTGAACCTCCTTCGTGCTAGGAATTGGGGCTTGCAATTGTTCCC
CATGAACGAGGAATTCCCAGTAAGCGCGAGTCATAAGCTCGCGTTGATTACGTCCCTGCCCTTTGTACACACC
GCCCGTCGCTACTATAGATTGAATGATTTAGTGAGGTCTTCGGACC-GGCGGTA--AC-CGGC-ACC--GGC-
----TCGTTCGG---TATCGGT-TTCGCTGGAAA-
GATGACCGAACTTGATCATTTAGATAAAGTAAAAGTCGTAACAAGG-TTTCCAGTCA--TTGGGTGA---

AAA-CCTAAAGGCGCAATGAAAGT-GTCAG-------------GTGAGACTCCTT-------------
TAGGAGC-----TCTCACCGGAGGGAGGATGGGTTACC----GTACATTTATT--------TGT--
CGGAGCCCCGCACTCCCGGGGCGTCTC--GTTCTC-ATTACG-AG----
TGGAGACGCACCAGGAGCGCACACGCCGGTACCCGAAAGATGGTGAACTATGCCTGGCCAGGACGAAGCCAGG
GGAAACCCTGGTGGAGGTCC--GAAGCG-ATTCTG--ACGTGCAAATCG-
ATCGTCTGAGCTGGGTATAGGGGCGAAAGACCAATCGAACCATCTGA-GGTTATAGT--
TAATCTGGTCAGTCTCGCTCATGATTGA-------
ATTTAATTTAATAGCCGCAGTAATTTGACTGTGCGAAGGTAGCATAATCATTTGTCTATTAATTGTAGACTTG
TATGAAGGGCTTAATGAAATACTAACTTTCTTTTTTTTATAAGAGT-----
GAATTTAATGTCTGAGTTAAAATGCTTAGATTTAT-TTATAGGACGAGAAGACCCTATAGAA--TTTTATT--
------ATAAATTTG--TTAATTTATTTTATGTA--TTTTG----ATAATGATTTGATGAATTT--
ATAATTTTGTTGGGGCGACAGGGAAA-TTTTATTAACTTTCTTT-TA-TTTTATT-TTAATTATTATTATT--
-CC-AATTGATCCTATA---TT--ATGGATA--GTAAGATTAAATTACCTTAGGGATAAC
Lattinestus_barrerai
ATGCAAGTCTCAGTACGAGCTACCT-
TAAAGCGAATCCGCAAATGGCTCAGTAAACCGGTCATTGTTTACCGGA-TATAAG-CCACTC-
ACGTGGATAACTGTGGTAATTCTAGAGCTAATACATGCATAC-AAGTCCCGACCAGTA---
ATGGGGCGAACGCTTTTATTGGAACAAAACCATTCGCCCGGCTCGCCC-GGGC------
GTCTCCTTGGTGACTCT-AGATAAC-ATAACA-GATCGCACGGTCTATGTACCGGC-
GACGTAACTTTCAAATGTCTGACTTATCAGCTATCGATGGTAGACTCTGTGTCTACCATGGCTCTCACGGGTA
ACGGGGAATCAGGGTTCGATTCCGGAGAGGGAGCCTGAGAGACGGCTACCACATCTAAGGAAGGCAGCAGGCA
CGCAAATTACCCACTCCCGGCACGGGGAGGTAGTGACAAAAAATAACGATACGGGACTCATCCGAGGCCCCGT
AATCGGAATGAGAACACTCTAAATCCTTTAACGAGGATCTATCGGAGGGCAAGTCTGGTGCCAGCAGCCGCGG
TAATTCCAGCTCCGATAGCGTATATTAAAGTTGTTGCGGTTAAAAA-
GCTCGTAGTTGGTTCTGCGTGCCACGCTGTCGGTTCACCGCCTGTCGGTGCAACTGGCATGCC-
GTGGCATGTCCTGTCGGTGGCCGAAGGGG-TTCCGG-TCGCGCGCTATGCCGGCCCGGGTAACCCGGGGT---
-AGGTCGGCGAG-GCCGCGTC-GCCC-TGGTAGG-CCGGCT-CAGTCCTACCGCGGTGCT-
CTTTGCCGAGTGTCGAGGTGGGCCGACACT-
GTTTACTTTGAACAAATTAGAGTGCTTAAAGCAGGCTCTTAAAGC-
CGCCTGAATAGTGGTGCATGGAATAATGGAACAGGACCTTGGTTCTATTTTGTT-GGTTTTCGGA-
ATCCGAGGTAATGATCAATAAGGA-CAGGCGGGGGCATTCGTATTGCGACGTTAGA-----------------
-GGTGAAATTCTTGG-
ATCGTCGCAAGACGCACTAGAGCGAAAGCATTTGCCAAGTATGTCTTAATTGATCAAGAACGAAAGTTAGGGG
TTCGAAGGCGATCAGATACCGCCCTAGTTCTAACCATAAACGATGCCAGCCAGCGATCCGCCGATGTTCCTCC
GATGACTCGGCGGGGAGCTC-
TACCCGGGAAACCAAAGCTTTTGGGTTCCGGGGGAAGTATGGTTGCAAAGCTGAAACTTAAAGGAATTGACGG
AAGGGCACTACCAGGCGTGGAGCCTGCGGCTTAATTTGACTCAACACGGGAAAGCTCACCAGGCCCGGACATT
GGAA-GGATTGACAGAT-CGATAGCTCTTT-CTCGATT-CAGTGGGT-
AGTGGTGCATGGCCGTTCGTAGTTGGTGGACTGATTTGTCTGGTTGATTCCGTTAACGAACGAGACTCTGGCC
TATTAAATAGGCGCTT-CCGGTATGG-ATA-CCTACCGGCGAGAT-
TTATTCTTCTTAAGGGGACCGGCGGCTCCAAGCCGCACGAGATTGAGCAATGACAGGTCAGTGATGCCCTTAG
ATGTTCTGGGCCGCACGCGCGCTACACTGAAGGAATCAGCGTGTGC--
TCCCCTGTCCGAAAGGACCGGGTAACCCGCTGAAACTCCTTCGTGCTAGGAATCGGGGCTTGCAATTCTTCCC
CATGAACGAGGAATGCCCAGTAAGTGCGAGTCATAAGCTCGCGCTGATTACGTCCCTGCCCTTTGTCCACACC
GCCCGTCGCTACTACCGATTGAATGATTTAGTGAGGTCTTCGGACC-GGCGGCG--ACGCGGC-ACC--GGC-
----TCGCCCGG---TGTCGGT-TTCGCTAAAAA-
GATGACCGAACTTGATCATTTAGAGGAAGTAAAAGTCGTAACAAGG-TTTCCAGTCT--CTGGGTGT---
GAAACCTACGGGCGCGATGACCGT-GTCTGTC----------AACCCGGCCCCCC-------------
CGGGGGCC---GGCCGGCGTTGGG-AGGATGGGCCTCCC---
GCCCCCCCCCGCAGCGGGGCGGGGCGGGGCCCCGCACTCCCGGGGCGTTTCGAGCCCTC-
CCTTCGAGGAGGGATGAGACGCACCAGGAGCGCGCGCGCTGGGACCCGAAAGATGGTGAACTATGCCTGGTCA
GGACGAAGCCAGGGGAAACCCTGGTGGAGGTCC--GAAGCG-ATTCTG--ACGTGCAAATCG-
ATCGTCTGAGCTGGGTATAGGCGCGAAAGAATAATCCAACTATCTTG-------
TTATAATATTAGGTTTGACCTGCTCAATGGTTAAT---------T-
TTAAATAGCTGCAGTATTTTGACTGTACAAAGGTAGCATAATCATTTGTCTTTTAATTGAAGACTAGTATGAA
GGGTTGGACGAGGTACTAACTTTCTTAA---TAATAATTA---
TAGAATTTAAATTTTAAGTTAAAATGCTTAAATTTAT-TTATGGGACGAGAAGACCCTATAGAA--
TTTTATTTTATT------------TTAATTATATAATTAACATTAATT-AT---TTTATATTTTTT-

TTAAAATA-TAATTTTATTGGGGTGATAGGAAAA-TTTATTAAACTTTTTTT--ATTAAAATT-
TCATTAATGAGTGTG----TTTAATGATCCTTAATAAGTATTGGGATA--
TGAAGATTAAATTACCTTAGGGATAAC
Legnotus_limbosus
ATGCA-GTCTCAGTACGAGCTTACT-
TAAAGTGAAACCGCAAAAGGCTCAGTAAACCGGTCATAGTTTACCGGA-TATATG-CCACTC-
ACGTGGATAACTGTGGTAATTCTAGAGCTAATACATGCAAAC-AAGTCCCGACCAGCG---
ATGGAAAGGACGCTTTTATTGGATCAAAACCATTCGCCCGGTTCGCCC-GGGC------
GTCTGCTTGATGACTCT-AGATAAC-ACCACA-GATCGCACGGTCATTGTACCGGC-
GACGTATCCGTCAAATGTCTGACTTATCAGCTGTCGATGGTAGACTCTGTGTCTACCATGGCCCTCACGGGTA
ACGGGGAATCAGGGTTCGATTCCGGAGAGGGAGCCTGAGAAACGGCTACCACATCCAAGGAAGGCAGCAGGCA
CGCAAATTACCCACTCCCGGCACGGGGAGGTAGTGACAAAAAATAACGATACGGGACTCATCCGAGGCCCCGT
AATCGGAATGAGAACACCCTAAATCCTTTAACGAGGATCTATTGGAGGGCAAGTCTGGTGCCAGCAGCCGCGG
TAATTCCAGCTCCAATAGCGTATATTAAAGTTGTTGCGGTTAAAAA-
GCTCGTAGTTGGTTCTGCGTGCCACGCTGTCGGTTCACCGCCTGTCGGTGCAACTGGCATGCC-
GTGGCATGTCCTGTCGGTGGCCTACTGGC-TTCGCG-GCGCACCCTATG-------CG-TTCACGCGTGT---
-AGGTCGTTGC--GCTGC-GTTGTCC-TCGTAGG-CCGGCT-CAATCCTACCGCGGTGCT-
CTTTACCGAGTGTCGAGGTAGGCCGACACT-
GTTTACTTTGAACAAATTAGAGTGCTTAAAGCAGGCTCTGAAAGC-
AGCCTGAATAGTGGTGCATGGAATAATGGAACAGGACCTTGGTTCTATTTTGTT-GGTTTTCGGA-
ATCCAAGGTAATGATCAATAAGGA-CAGGCGGGGGCATTCGTATTGCGACGTTAGA-----------------
-GGTGAAATTCTTGG-
ATCGTCGCAAGACGCACTAGAGCGAAAGCATTTGCCAAGTATGTCTTAATTGATCAAGAACGAAAGTTAGAGG
TTCGAAGGCGATCAGATACCGCCCTAGTTCTAACCATAAACGATGCCAGCCAGCGATCCGCCGATGTTCCTCC
GATGACTCGGCGGGGAGCTT-
CCCCCGGGAAACCAAAGCTTTTGGGTTCCGGGGGAAGTATGGTTGCAAAGCTGAAACTTAAAGGAATTGACGG
AAGGGCACCACCAGGAGTGGAGCCTGCGGCTTAATTTGACTCAACACGGGAAACCTCACCAGGCCCGGACATT
GGAA-GGATTGACAGAT-CGATAGCTCTTT-CTCGATT-CAGTGGGT-
AGTGGTGCATGGCCGTTCTTAGTTGGTGGACTGATTTGTCTGGTTAATTCCGATAACGAACGAGACTCTAACC
TATTAACTAGGCGTCT-CCGGTATAC-ATA-CCTACCGGCGAGAC-
TTATTCTTCTTAAGGGGACAGGCGGCTCTTAGCCGCACGAGATTGAGCAATAACAGGTCTGTGATGCCCTTAG
ATGTTCTGGGCCGCACGCGCGCTACACTGAAGGAATCAGCGTGTTC--
TCCCCTGTCCGAAAGGACCGGGCAACCCGCTGAACCTCCTTCGTGCTAGGAATTGGGGCTTGCAATTGTTCCC
CATGAACGAGGAATTCCCAGTAAGCGCGAGTCATAAGCTCGCGTTGATTACGTCCCTGCCCTTTGTACACACC
GCCCGTCGCTACTACAGATTGAATGATTTAGTGAGGTCTTCGGACC-GGCGGTA--AC-AGGC-ATC--
GGCT-----CGCCCTT---TATCTGT-TCCGCTGGAAA-
GATGACCGAACTTGATCATTTAGATGAAGTAAAAGTCGTAACAAGG-TTTCCAGTCA--TTGGGTGT---
AAA-CCTAAAGGCGCAATGAAAGT-GTC-------------AGGGGGTCCTCCT-------------
ACGGGTGG----ATCTCCCCGCTGGGAAGATGGGCCACC----GTACAATTAAT--------TGT--
CGGAGCCCCGCATTCCCGGGGCGTCTC--GTCCTC-ATTGCG-AG----
TAGAGACGCACCAGGAGCGCACACGCTGGTACCCGAAAGATGGTGAACTATGCCTGGCCAGGACGAAGCCAGG
GGAAACCCTGGTGGAGGTCC--GAAGCG-ATTCTG--ACGTGCAAATCG-
ATCGTCTGAGCTGGGTATAGGGGCGAAAGACCAATCGAACCATCTTGTT-TATTTTT--
ATTTTAAGTCAATCCTGCTCAATGATCATTTA------
TGATTAAATAGCCGCAGTATTTTGACTGTGCAAAGGTAGCATAATCATTTGTCTGTTAATTATAGGCTGGTAT
GAACGGATAAATGAAATATTAACTTTCTTTATTTTATATTTAT-----
GAATTTAATTTTTAGGTTAAAAAGCTTAAATGTTT-TTGTAGGACGAGAAGACCCTATAGAA--
TTTTATTTATTT--GT--------TGATTAGTT-TAATTTTTTT-ATTATT----ATTAT-TTTTAATAGA--
--TGAATTTTGTTGGGGTGACAGGGAAA-ATTTTATAACTTTCTTTATTAATTG--T-
TTATTGATTTATATGT-----TAAGGATCCTT-----TATTAGGGAA---
GTAAGATTAAATTACCTTAGGGATAAC
Lestonia_haustorifera
ATGCAAGTCTCAGTACGAACTTCCT-
TAAAGTGAAACCGCAAAAGGCTCAGTAAACCGGTCATAGTTTACCGGA-TGTATT-CCACTT-
ACGTGGATAACTGTGGTAATTCTAGAGCTAATACATGCACTT-AAGTCTCGACCAGCG---
ATGGTCGCGACGCTTTTATTGGATCAAAACCATTCGCCCGGCTCCGGCCGGGC------
GCAACAATGGCGACTCT-AGATAAT-ACCACA-GATCGCACGGTCTCAGTACCGGC-
GACGTATCCGTCAAATGTCTGACTTATCAGCCGTCGATGGTAGACTCTGTGTCTACCATGGCTTTCACGGGTG

ACGGGGAATCAGGGTTCGATTCCGGAGAGGGAGCCTGAGAAACGGCTACCACATCCAAGGAAGGCAGCAGGCA
CGCAAATTACCCACTCCCGGCACGGGGAGGTAGTGACAAAAAATAACGATACGGGACTCATCCGAGGCCCCGT
AATCGGAATGAGAACACTCCAAATCCTTTAACGAGTATCTATTGGAGGGCAAGTCTGGTGCCAGCAGCCGCGG
TAATTCCAGCTCCAATAGCGTATATTAAAGTTGTTGCGGTTAAAAA-
GCTCGTAGTTGGTTCTGCGTGCCACGCTGTCGGTTCACCGCCTGTCGGTGCAACTGGCATGCC-
GTGGCATGTCCTGTCGGTGGCCCCACATGCCTCGCGTGGGCGCCCTATG-------CG-TTAACGCGCGT---
-AGG-AGTCGC--TCTGC-TAGGTCA-TCGTGGGGCCGGTT-CAATCCTGCCGCGGTGCT-
CTTTACCGAGTGTCGAGGTAGGCCGACAAT-
GTTTACTTTGAACAAATTAGAGTGCTTAAAGCAGGCTATGAAAGT-
AGCCTGAATAGTGTTGCATGGAATAATGGAACAGGACCTTGGTTCTATTTTGTT-GGTTTCCGGA-
ATCCAAGGTAATGATCAATAAGGA-CAGGCGGGGGCATTCGTATTGCGGCGTTAGA-----------------
-GGTGAAATTCTTGG-
ATCGTCGCAAGACGCACTAGAGCGAAAGCATTTGCCAAGTATGTCTTAATTGATCAAGAACGAAAGTTAGAGG
TTCGAAGGCGATCAGATACCGCCCTAGTTCTAACCATAAACGATGCCAGCCAGCGATCCGCCGATGTTCCTCC
GATGACTCGGCGGGGAGCTT-
CCCCCGGGAAACCAAAGCTTTTGGGTTCCGGGGGAAGTATGGTTGCAAAGCTGAAACTTAAAGGAATTGACGG
AAGGGCACCACCAGGAGTGGAGCATGCGGCTTAATTTGACTCAACACGGGAAACCTCACCAGGCCCGGACATT
GGAA-GGATTGACAGAT-CGATAGCTCTTT-CTCGATT-CAGTGGGT-
AGTGGTGCATGGCCGTTCTTAGTTGGTGGACTGATTTGTCTGGTTAATTCCGATAACGAACGAGACTCTGGCC
TATTAACTAGGCGATC-CCGGTATAC-ATA-CCTACCGGCGATAT-
TTATTCTTCTTAAAGGGACAGGCGGCTCTTAGCCGCACGAGATTGAGCAATAACAGGTCTGTGATGCCCTTAG
ATGTTCTGGGCCGCACGCGCGCTACACTGAAGGAATCAGCGTGTTC--
TCCCCTGTCCGAAAGGACCGGGCAACCCGCTGAACCTCCTTCGTGCTAGGAATTGGGGCTTGCAATTCTTCCC
CATGAACGAGGAATTCCCAGTAAGCGCGAGTCATAAGCTCGCGCTGATTACGTCCCTGCCCTTTGTACACACC
GCCCGTCGCTACTACTGATTGAATGATTTAGTGAGGTCTTCGGACC-GGCGGTA--AC-AGGC-ACC--
GGCT-----CGCCCGG---TATCGGT-TCCGCTGGAAA-
GATGACCGAACTTGGTCATTTAGAGGAAGTAAAAGTCGTAACAAGT-GCGCGAGTCA--TTGGGTTCT--
AAAACCTAAAGGCGCAATGAAAGT-GTC-------------AGGTGGGCCTCTC-------------
AAGGGAGG-----TCTCCCCGCTGGGAGGATGGTCTACC----GGACAACTA-T--------TGT--
CGGTGACCCGCACTCCCGGGGCGTCTC--GTTCTC-ATCACG-AG----
TAGAGACGCACCAGGAGCGCACACGCTGGTACCCGAAAGATGGTGAACTATGCCTGGCCAGGACGAAGCCAGG
GGAAACCCTGGTGGAGGTCC--GAAGCG-ATTCTG--ACGTGCAAATCG-
ATCGTATGAGCTGGGTATAGGGGCGAAAGACCAATCGAACCATCTAG--GTAAATTT------
TAGCTGGTTCCCTCTGAATGATA-TTTAA-----TTA--
AAATAGCCGCAGTATTTTGACTGTGCAAAGGTAGCATAATCATTTGTCTTTTAATTGGGGACTGGTATGAAGG
GTTTGATGAAATATCAACTTTCTTTA---TATAAATATTT--
AAAACTTAATTTTTTAGTTAAAAAGCTAAGATTTTA-TTATAGGACGAKAAGACCCTATAGAA--
TTTTATAGTATGGTTA----------A-TATAAATTATTTGGATATTT--A----AGTGTATATTAGT-TAT-
--AATATTTTGTTGGGGTGACAGGGAAA-TAATATTAACTTTCTTTATTTATTTT---
TTATTAATGTATATAT-----AGTTGATCCTT-----GGTGGAGGAAA--
ATAAGATTAAATTACCTTAGGGATAAC
Lindbergicoris_hochii
ATGCAAGTCTCAGTACGAGCTGACT-
TAAAGTGAAACCGCAAAAGGCTCAGTAAACCGGTCATAGTTTACCGGG-TATATG-CCACTC-
ACGTGGACAACTGTGGTAATTCTAGAGCTAATACATGCAACC-AAGTCCCGACCAGCG---
ATGGAAAGGACGCTTTTATTGGATCAAAACCATTCGCCCGGCCCCGCCCGGGC------
GTCTGCTTGATGACTCT-AGATAAC-ACCACA-GATCGCACGGTCATAGTACCGGC-
GACGTATCCGTCAAATGTCTGACTTATCAGCTGTCGATGGTAGACTCTGTGTCTACCATGGCGCTCACGGGTA
ACGGGGAATCAGGGTTCGATTCCGGAGAGGGAGCCTGAGAAACGGCTACCACATCCAAGGAAGGCAGCAGGCA
CGCAAATTACCCACTCCCGGCACGGGGAGGTAGTGACAAAAAATAACGATACGGGACTCATCCGAGGCCCCGT
AATCGGAATGAGAACACCCTAAATCCTTTAACGAGGATCTATTGGAGGGCAAGTCTGGTGCCAGCAGCCGCGG
TAATTCCAGCTCCAATAGCGTATATTAAAGTTGTTGCGGTTAAAAA-
GCTCGTAGTTGGTTCTGCGTGCCACGCTGTCGGTTCACCGCCTGTCGGTGCAACTGGCATGCC-
GTGGCATGTCCTGTCGGTGGCCTCACAGGCTTCGTG-GCGCGCCCTACG-------CG-TTAACACGTGT---
-AGGTAGTTGC--GCCGC-GTTGTTC-TTGTAGG-CCGGCT-CAATCCTGCCGCGGTGCT-
CTTTACCGAGTGTCGAGGTAGGCCGACACT-
GTTTACTTTGAACAAATTAGAGTGCTTCAAGCAGGCTCTGAAAGC-
AGCCTGAATAGTGGTGCATGGAATAATGGAACAGGACCTTGGTTCTATTTTGTT-GGTTTTAGGA-

ATCCAAGGTAATGATCAATAAGGA-CAGGCGGGGGCATTCGTATTGCGACGTTAGA-----------------
-GGTGAAATTCTTGG-
ATCGTCGCAAGACGCACTAGAGCGAAAGCATTTGCCAAGTATGTCTTAATTGATCAAGAACG?AAGTTAGAGG
TTCGAAGGCGATCAGATACCGCCCTAGTTCTAACCATAAACGATGCCAGCCAGCGATCCGCCGATGTTCCTCC
GATGACTCGGCGGGGAGCTT-
TCCCCGGGAACCCAAAGCTTTTGGGTTCCGGGGGAAGTATGGTTGCAAAGCTGAAACTTAAAGGAATTGACGG
AAGGGCACCACCAGGAGTGGAGCCTGCGGCTTAATTTGACTCAACACGGGAAACCTCACCAGGCCCGGACATT
GGAA-GGATTGACAGAT-CGATAGCTCTTT-CTCGATT-CAGTGGGT-
AGTGGTGCATGGCCGTTCTTAGTTGGTGGACTGATTTGTCTGGTTAATTCCGATAACGAACGAGACTCTGACC
TATTAACTAGGCGCTC-CCGGTATAC-ATA-CCTACCGGCGAGAT-
TTATTCTTCTTAAAGGGACAGGCGGCTCTTAGCCGCACGAGATTGAGCAATAACAGGTCTGTGATGCCCTTAG
ATGTTCTGGGCCGCACGCGCGCTACACTGAAGGAATCAGCGTGTGC--
TCCCCTGTCCGAAAGGACCGGGCAACCCGCTGAACCTCCTTCGTGCTAGGAATTGGGGCTTGCAATTGTTCCC
CATGAACGAGGAATTCCCAGTAAGCGCGAGTCATAAGCTCGCGTTGATTACGTCCCTGCCCTTTGTACACACC
GCCCGTCGCTACTACCGATTGAATGATTTAGTGAGGTCTTCGGACC-GGCGGTA--AC-CGGC-ACC--
GGCA-----CGTTCGG---TATCGGT-TTCGCTGGAAA-
GATGACCGAACTTGATCATTTAGAGGAAGTAAAAGTCGTAACAAGG-TTTCCAGTCA--TTGGGTTCT--
AAAACCTAAAGGCGAAATGAAAGT-GTC-------------AGGGAGGCCTCCT-------------
AAGGGAGG-----TCTTCTCACTGGGAGGATGGGCCACC----GTACAATTA-C--------TGT--
CGGAGCCCCGCACTCCCGGGGCGTCTC--GTTCTC-ATCACG-AG----
TAGAGACGCACCAGGAGCGCACACGCTGGTACCCGAAAGATGGTGAACTATGCCTGGCCAGGACGAAGCCAGG
GGAAACCCTGGTGGAGGTCC--GAAGCG-ATTCTG--ACGTGCAAATCG-
ATCGTATGAGCTGGGTATAGGGGCGAAAGACCAATCGAACCATCTT---GTATATTT--
ATATTAAGTTTTACCTGCTCAATGATATTAA-------
ACATTAAATAGCCGCAGTATTTTGACTGTGCAAAGGTAGCATAATCATTTGTCTTTTAATTGTGGACTTGTAT
GAATGGTTTGATGAAATATTTACTTTCTTTA---TCTAAAATCAT--
TTAATTTAATCTTTTAGTTAAAAAGCTAAAATGACT-TTATAGGACGAGAAGACCCTATAGAA--
TTTTATAAATCT-TTA---------TA-TAATTTATTTATAGTTATTTTTA----ATAAAATATATTTATA--
--TTTATTTTGTTGGGGTGACAGGGAAA-TTTAATTAACTTTCTTTATTAAAATG---
TTATTGATTTATATAT-----TTATAATCCTA-----TTTTAAGGATA--
TTAAGATTAAATTACCTTAGGGATAAC
Lygaeus_equestris
ATGCAAGTCTCAGTACGAGCTGACT-
TAAAGTGAAACCGCAAAAGGCTCAGTAAACCGGTCATAGTTTACCGGA-TAAATG-CCACTC-
ACGTGGATAACTGTGGTAATTCTAGAGCTAATACATGCAAAC-AAGTCCCGACCAGCG---
ATGGAGAGGACGCTTTTATTTGATCAAAACCATTCGCCCGGCTTGTCC-GGGC------
GTCGGCTTGATGACTCT-GGATAAC-ACCACA-GATCGCACGGTCACAGTACCGGT-
GACATATCCTTCAAATGTCTGACTTATCAGCTGTCGATGGTAGACTCTGTGTCTACCATGGCGCTCACGGGTG
ACGGGGAATCAGGGTTCGATTCCGGAGAGGGAGCCTGAGAAACGGCTACCACATCCAAGGAAGGCAGCAGGCA
CGCAAATTACCCACTCCCGGCACGGGGAGGTAGTGACAAAAAATAACGATACGGGACTCATCCGAGGCCCCGT
AATCGGAATGAGAACACTCTAAATCCTTTAACGATGATCTATTGGAGGGCAAGCCTGGTGCCAGCAGCCGCGG
TAATTCCAGCTCCAATAGCGTATATTAAAGTTGTTGCGGTTAAAAA-
GCTCGTAGTTGGTTCTGCGTGCCACGCTGTCGGTTCACCGCCTGCCGGTGCAACTGGCATGCC-
GTGGCATGTCCTGTCGGTGGCGGATAGGC-CTCCCG-GCGTGCCCTATGCGG----TAT-ACCAGCGTGT---
-AGGCTGCTGC--GCCGGGGGTGTCC-TTGTACG-CCGACT-CAATCCTACCGCGGTGCT-
CTTTACCGAGTGTCGAGGTAGGCCGACACT-
GTTTACTTTGAACAAATTAGAGTGCTTCAAGCAGGCTCTTAAATC-
AGCCTGAATAGTGGTGCATGGAATAATGGAACAGGACCTTGGTTCTATTTTGTT-GGTTTCCGGA-
ATCCAAGGTAATGATCAATAAGGA-CAGGCGGGGGCATTCGTATTGCGACGTTAGA-----------------
-GGTGAAATTCTTGG-
ATCGTCGCAAGACGCACTAAAGCGAAAGCATTTGCCAAGTATGTCTTAATTGATCAAGAACGAAAGTTAGAGG
TTCGAAGGCGATCAGATACCGCCCTAGTTCTAACCATAAACGATGCCAGCCAGCGATCCGCCGACGTTCCTCC
GATGACTCGGCGGGGAGCTT-
TCCCCGGGAAACCAAAGCTTTTGGGTTCCGGGGGAAGTATGGTTGCAAAGCTGAAACTTAAAGGAATTGACGG
AAGGGCACCACCAGGAGTGGAGCCTGCGGCTTAATTTGACTCAACACGGGAAACCTCACCAGGCCCGGACATT
GGAA-GGATTGACAGAT-CGATAGCTCTTT-CTTAATT-CAGTGGGT-
AGTGGTGCATGGCCGTTCTTAGTTGGTGGACTGATTTGTCTGGTTAATTCCGATAACGAACGAGACTCCGACC
TATTAACTAGGCGATA-CCGGTATAGA-TG-ACTACCGGCGAGAT-

TTATTCTTCTTAAAGGGACAGGCGGCTCTTAGCCGCACGAGATTGAGCAATAACAGGTCAGTGATGCCCTTAG
ATGTTCTGGGCCGCACGCGCGCTACACTGAAGGAATCAGCGTGTGC--
TCCCCTGTCCGAAAGGTCCGGGTAACCCGCTGAACCTCCTTCGTGCTAGGAATTGGGGCTTGCAATTTTTCCC
CATGAACGAGGAATTCCCAGTAAGCGCGAGTCATAAGCTCGCGTTGATTAAGTCCCTGCCCTTTGTACACACC
GCCCGTCGCTACTACCGATTGAATGATTTAGTGAGGTCTTCGGACC-GGCGATA--AC-CGGC-ACC--
GGCTTT--CTGTTCGG---TATCGAT-TTCGCTGGAAA-
GATGACCGAACTTGATCATTTAGAGGAAGTAAAAGTCGTAACAAGG-TTTCCAGTCA--TTGGGTGG---
AAAACCTAAAGGCGCAATGAAAGT-GTCTG---------------GGGACGC---------------------
----------CTTCGGCGGGAAAATGGGCCCACC---GGCCCTCCGGC-------------
CGGAGCCTTGCACTCCCGGG-CGTCTC--GTTCTCATTTA-G-AG----
TCGAGACGCACCAGGAGCGCACACGCTGTTACCCGAAAGATGGTGAACTATGCCTGGCCAGGACGAAGCCAGG
GGAAACCCTGGTGGAGGTCC--GAAGCG-ATTCTG--ACGTGCAAATCA-
ATCGTCTAAGCTGGGTATAGGGGCGAAAGACCAATCGAACTATCTAG----AT-A-
TTAATTTTAGGTTTAATCTGCTCAGTGATTAATT----------
TTAAATAGCTGCAGTATTTTGACTGTACAAAGGTAGCATAATCATTTGTCTTTTAATTGGAGGCTTGTATGAA
TGATTGGACGAAAAATAATCTTTCTTTATTTAA-TAATTT----
TGAATTTAATTTTTTAGTTAAAAAGCTAAAATGAAT-TTGTAAGACGAGAAGACCCTATAGAA--TTTTAT--
------------TTTATTTATTTTATTTAT----TTTATTTTT---AAATGTTTTTAATTTAAATTAAATTT-
-GGTTGGGGTGACTATGAAA-ATTTTTTAACTTTCATTTATTTT-TT---TCATAAATTAATGTTGTA---
TATTGATCCTGTT---TTAT--AGATT--AAAAGATTAAATTACCTTAGGGATAAC
Macroscytus_annulipoides
ATGCAAGTCTCAGTACGAGCTGACT-
TAAAGTGAAACCGCAAAAGGCTCAGTAAACCGGTCATAGTTTACCGGA-TATATG-CCACTC-
ACGTGGATAACTGTGGTAATTCTAGAGCTAATACATGCAACC-AAGTCCCGACCAGCG---
ATGGAAAGGACGCTTTTATTGGATCAAAACCATTCGCTCGGCTCGCCCCGGGC------
GTCTGCTTGATGACTCT-AGATAAC-ACCACA-GATCGCACGGTCACAGTACCGGC-
GACGTATCCGTCAAATGTCTGACTTATCAGCTGTCGATGGTAGACTCTGTGTCTACCATGGCGCTCACGGGTA
ACGGGGAATCAGGGTTCGATTCCGGAGAGGGAGCCTGAGAAACGGCTACCACATCCAAGGAAGGCAGCAGGCA
CGCAAATTACCCACTCCCGGCACGGGGAGGTAGTGACAAAAAATAACGATACGGGACTCATCCGAGGCCCCGT
AATCGGAATGAGAACACCCTAAATCCTTTAACGAGGATCTATTGGAGGGCAAGTCTGGTGCCAGCAGCCGCGG
TAATTCCAGCTCCAATAGCGTATATTAAAGTTGTTGCGGTTAAAAA-
GCTCGTAGTTGGTTCTGCGTGCCACGCTGTCGGTTCACCGCCTGTCGGTGCAACTGGCATGCC-
GTGGCATGTCCTGTCGGTGGCTGACAGGC-TTCGCG-GCGCGCCCTATG-------CGTTCAC-GCGTGT---
-AGGTCGTCGC--GCCGCCGTTGTCC-TTGTAGG-CCGGCT-CAATCCTACCGCGGTGCT-
CTTTGCCGAGTGTCGAGGTAGGCCGACACT-
GTTTACTTTGAACAAATTAGAGTGCTTCAAGCAGGCTCTGAAAGC-
AGCCTGAATAGTGGTGCATGGAATAATGGAACAGGACCTTGGTTCTATTTTGTT-GGTTTCCGGA-
ATCCAAGGTAATGATCAATAAGGA-CAGGCGGGGGCATTCGTATTGCGACGTTAGA-----------------
-GGTGAAATTCTTGG-
ATCGTCGCAAGACGCACTAGAGCGAAAGCATTTGCTAAGTATGTCTTAATTGATCAAGAACGAAAGTTAGAGG
TTCGAAGGCGATCAGATACCGCCCTAGTTCTAACCATAAACGATGCCAGCCAGCGATCCGCCGATGTTCCTCC
GATGACTCGGCGGGGAGCTT-
TCCCCGGGAAACCAAAGCTTTTGGGTTCCGGGGGAAGTATGGTTGCAAAGCTGAAACTTAAAGGAATTGACGG
AAGGGCACCACCAGGAGTGGAGCCTGCGGCTTAATTTGACTCAACACGGGAAACCTCACCAGGCCCGGACATT
GGAA-GGATTGACAGAT-CGATAGCTCTTT-CTCGATT-CAGTGGGT-
AGTGGTGCATGGCCGTTCTTAGTTGGTGGACTGATTTGTCTGGTTAATTCCGATAACGAACGAGACTCTGACC
TATTAACTAGGCGCTC-CCGGTATAA-ATA-CCTACCGGCGAGAT-
TTATTCTTCTTAAGGGGACAGGCGGCTCTTAGCCGCACGAGATTGAGCAATAACAGGTCTGTGATGCCCTTAG
ATGTTCTGGGCCGCACGCGCGCTACACTGAAGGAATCAGCGTGTGC--
TCCCCTGTCCGAAAGGTCCGGGCAACCCGCTGAACCTCCTTCGTGCTAGGAATTGGGGCTTGCAATTGTTCCC
CATGAACGAGGAATTCCCAGTAAGCGCGAGTCATAAGCTCGCGTTGATTACGTCCCTGCCCTTTGTACACACC
GCCCGTCGCTACTACCGATTGAATGATTTAGTGAGGTCTTCGGACC-GGCGGTA--AC-CGGC-ACC--GGC-
----TCGTTCGG---TATCGGT-TTCGCTGGAAA-
GATGACCGAACTTGATCATTTAGAGGAAGTAAAAGTCGTAACAAGG-TTTCCAGTCA--TTGGGTGT---
AAAACCTAAAGGCGCAATGAAAGT-GTCAG-------------GTGGGACTCCTT-------------
TAGGAGC-----TCCCACCGCAGGGAGGATGGGCCACC----GTACAATTACT--------TGT--
CGGAGCCCCGCACTCCCGGGGCGTCTC--GTTCTC-ATTGCG-AG----
TAGAGACGCACCAGGAGCGCACACGCTGGTACCCGAAAGATGGTGAACTATGCCTGGCCAGGACGAAGCCAGG

GGAAACCCTGGTGGAGGTCC--GAAGCG-ATTCTG--ACGTGCAAATCG-
ATCGTCTGAGCTAGGTATAGGGGCGAAAGACCAATCGAACCATCTTG-TT--AAATT--
ATATTAAGTCAGGCCTGCTCAGTGATTATT-----AATA-
ATTAAATAGCCGCAGTAATTTGACTGTGCGAAGGTAGCATAATCATTTGTCTTTTAATTGGAGGCTTGTATGA
ATGGTTTGATGAAACTCTAGCTTTCTTTATTGTAAAT-TTA-----
GAATTTAATATGTGAGTTAAAAAGCTTAGATATTT-CCATAGGACGAGAAGACCCTATAGAA--TTTTACTT-
------ATATTTATAAATTTATT-AGTATAGGGTG-ATTTT----ATTACAGATAAGTTTTATAT-A-
AATTGTGTTGGGGTGACAAAGAAA-TTAATTTAACTTTCTTTTATGTTTA----TTAATTATGTTTATA----
TT-TATGATCCTTTT---TT--ATGGATA--GTAAGATTAAATTACCTTAGGGATAAC
Macroscytus_badius
ATGCAAGTCTCAGTACGAGCTGACT-
TAAAGTGAAACCGCAAAAGGCTCAGTAAACCGGTCATAGTTTACCGGA-TATATG-CCACTC-
ACGTGGATAACTGTGGTAATTCTAGAGCTAATACATGCAACC-AAGTCCCGACCAGCG---
ATGGAAAGGACGCTTTTATTGGATCAAAACCATTCGCTCGGCTCGCCCCGGGC------
GTCTGCTTGATGACTCT-AGATAAC-ACCACA-GATCGCACGGTCACAGTACCGGC-
GACGTATCCGTCAAATGTCTGACTTATCAGCTGTCGATGGTAGACTCTGTGTCTACCATGGCGCTCACGGGTA
ACGGGGAATCAGGGTTCGATTCCGGAGAGGGAGCCTGAGAAACGGCTACCACATCCAAGGAAGGCAGCAGGCA
CGCAAATTACCCACTCCCGGCACGGGGAGGTAGTGACAAAAAATAACGATACGGGACTCATCCGAGGCCCCGT
AATCGGAATGAGAACACCCTAAATCCTTTAACGAGGATCTATTGGAGGGCAAGTCTGGTGCCAGCAGCCGCGG
TAATTCCAGCTCCAATAGCGTATATTAAAGTTGTTGCGGTTAAAAA-
GCTCGTAGTTGGTTCTGCGTGCCACGCTGTCGGTTCACCGCCTGTCGGTGCAACTGGCATGCC-
GTGGCATGTCCTGTCGGTGGCTGACAGGC-TTCGCG-GCGCGCCCTATG-------CGTTCAC-GCGTGT---
-AGGTCGTCGC--GCCGCTGTTGTCC-TTGTAGG-CCGGCT-CAATCCTACCGCGGTGCT-
CTTTGCCGAGTGTCGAGGTAGGCCGACACT-
GTTTACTTTGAACAAATTAGAGTGCTTCAAGCAGGCTCTGAAAGC-
AGCCTGAATAGTGGTGCATGGAATAATGGAACAGGACCTTGGTTCTATTTTGTT-GGTTTCCGGA-
ATCCAAGGTAATGATCAATAAGGA-CAGGCGGGGGCATTCGTATTGCGACGTTAGA-----------------
-GGTGAAATTCTTGG-
ATCGTCGCAAGACGCACTAGAGCGAAAGCATTTGTTAAGAATGTCTTAATTGATCAAGAACGAAAGTTATAGG
TTCGAAGGCGATCAGATACCGCCCTAGTTCTAACCAAAAACGATGCCAGCCTCCGATCCGCCGATGTTCCTCC
GATAACTCGGCGGGGAGCTT-
TCCCCGGGAAACCAAAGCTTTTGGGTTCCGGGGGATGAATGGTTGCTAAGCTGAAACTTAAAGGAATTGCCGG
AAGGGCACCACCAGGAGTGGAGCCTGCGGCTTAATTTGACTCAACACGGGAAACCTCACCAGGCCCGGACATT
GGAA-GGATTGACACAA-CCATAGCTCTTT-CTCGATT-CAGTGGAT-
AGTGGTGCATGGCCGTTCTTAGTTGGACGACTGATTTCTCTCGTTAATTCCGATAACGAACGAGAATCTGACC
TATTAAGTAGGCCGTC-CCGGTATAA-ATA-CCTACGGGCGAGAT-
TTATTCTTCTTAAGGGGACAGGCGGCTCTTGTCCGCACGAGATTGAGCAATAACAGGTCTGTGATGCCCTTAG
ATGTTCTGGGCCGCACGCGCGCTACGCTGAAAGAATCAGCGTGTGC--
TCCGCTGTCCGAAAGGTCCGGGCAACCCGCTGAACCTCCTTCATGCTAGGAATTGGGGCTTGCAATTGTTCCC
CATGAACGAGGAATTCCCAGTAAGCGCGAGTCATAAGCTCGCGTTGATTACGTCCCTGCCCTTTGTACACGCC
GCCCGTCGCTACTACCGAATGAATGATTTAGTGAGGTCATCGGACC-GGCACTG--AC-CGGC-ACC--CGC-
----TCTGTCTG---TATCGGT-TTCGCTGGATA-
GATGAGCGAACTTGTTCATTTAGAGGAAGTAAAAGTCGTAACAAGG-TTTCCAGTCA--TTGGGTGT---
AAAACCTAAAGGCGCAATGAAAGT-GTCAG-------------GTGGGACTCCTT-------------
TAGGAGC-----TCCCACCGCAGGGAGGATGGGCCACC----GTACAATTATT--------TGT--
CGGAGCCCCGCACTCCCGGGGCGTCTC--GTTCTC-ATTGCG-AG----
TAGAGACGCACCAGGAGCGCACACGCTGGTACCCGAAAGATGGTGAACTATGCCTGGCCAGGACGAAGCCAGG
GGAAACCCTGGTGGAGGTCC--GAAGCG-ATTCTG--ACGTGCAAATCG-
ATCGTCTGAGCTAGGTATAGGGGCGAAAGACCAATCGAACCATCTTG-TTATAAATT--
ATATCAAGTCAGGCCTGCTCAGTGATTATA-----TAGA-
ATTAAATAGCCGCAGTAATTTGACTGTGCGAAGGTAGCATAATCATTTGTCTTTTAATTGAAGGCTTGTATGA
ATGGTTTGATGAAACTCTGACTTTCTTCACTGTAAATGTTA-----
AAATTTAATATTTGAGTTAAAAAGCTTAGATATTT-CCATAGGACGAGAAGACCCTATAGAA--TTTTACT--
------GTATGTTTAATATTATT-TATTTTAGGT--ATTTT----ATTTTTGAGAATTTTAAATT-
ACAGTTATGTTGGGGTGACAAAGAAA-TTAACTTAACTTTCTTTATT-TTTGATT-TTAATGATTTTTATA--
--TT-TATGATCCTTTA---TT--ATGGATA--GTAAGATTAAATTACCTTAGGGATAAC
Macroscytus_brunneus

ATGCAAGTCTCAGTACGAGCTGACT-
TAAAGTGAAACCGCAAAAGGCTCAGTAAACCGGTCATAGTTTACCGGA-TATATG-CCACTC-
ACGTGGATAACTGTGGTAATTCTAGAGCTAATACATGCAACC-AAGTCCCGACCAGCG---
ATGGAAAGGACGCTTTTATTGGATCAAAACCATTCGCTCGGCTCGCCCCGGGC------
GTCTGCTTGATGACTCT-AGATAAC-ACCACA-GATCGCACGGTCACAGTACCGGC-
GACGTATCCGTCAAATGTCTGACTTATCAGCTGTCGATGGTAGACTCTGTGTCTACCATGGCGCTCACGGGTA
ACGGGGAATCAGGGTTCGATTCCGGAGAGGGAGCCTGAGAAACGGCTACCACATCCAAGGAAGGCAGCAGGCA
CGCAAATTACCCACTCCCGGCACGGGGAGGTAGTGACAAAAAATAACGATACGGGACTCATCCGAGGCCCCGT
AATCGGAATGAGAACACCCTAAATCCTTTAACGAGGATCTATTGGAGGGCAAGTCTGGTGCCAGCAGCCGCGG
TAATTCCAGCTCCAATAGCGTATATTAAAGTTGTTGCGGTTAAAAA-
GCTCGTAGTTGGTTCTGCGTGCCACGCTGTCGGTTCACCGCCTGTCGGTGCAACTGGCATGCC-
GTGGCATGTCCTGTCGGTGGCTGACAGGC-TTCGCG-GCGCGCCCTATG-------CGTTCAC-GCGTGT---
-AGGTCGTCGC--GCCGCTGTTGTCC-TTGTAGG-CCGGCT-CAATCCTACCGCGGTGCT-
CTTTGCCGAGTGTCGAGGTAGGCCGACACT-
GTTTACTTTGAACAAATTAGAGTGCTTCAAGCAGGCTCTGAAAGC-
AGCCTGAATAGTGGTGCATGGAATAATGGAACAGGACCTTGGTTCTATTTTGTT-GGTTTCCGGA-
ATCCAAGGTAATGATCAATAAGGA-CAGGCGGGGGCATTCGTATTGCGACGTTAGA-----------------
-GGTGAAATTCTTGG-
ATCGTCGCAAGACGCACTAGAGCGAAAGCATTTGCCAAGTATGTCTTAATTGATCAAGAACGAAAGTTAGAGG
TTCGAAGGCGATCAGATACCGCCCTAGTTCTAACCATAAACGATGCCAGCCAGCGATCCGCCGATGTTCCTCC
GATGACTCGGCGGGGAGCTT-
TCCCCGGGAAACCAAAGCTTTTGGGTTCCGGGGGAAGTATGGTTGCAAAGCTGAAACTTAAAGGAATTGACGG
AAGGGCACCACCAGGAGTGGAGCCTGCGGCTTAATTTGACTCAACACGGGAAACCTCACCAGGCCCGGACATT
GGAA-GGATTGACAGAT-CGATAGCTCTTT-CTCGATT-CAGTGGGT-
AGTGGTGCATGGCCGTTCTTAGTTGGTGGACTGATTTGTCTGGTTAATTCCGATAACGAACGAGACTCTGACC
TATTAACTAGGCGCTC-CCGGTATAA-ATA-CCTACCGGCGAGAT-
TTATTCTTCTTAAGGGGACAGGCGGCTCTTAGCCGCACGAGATTGAGCAATAACAGGTCTGTGATGCCCTTAG
ATGTTCTGGGCCGCACGCGCGCTACACTGAAGGAATCAGCGTGTGC--
TCCCCTGTCCGAAAGGACCGGGCAACCCGCTGAACCTCCTTCGTGCTAGGAATTGGGGCTTGCAATTGTTCCC
CATGAACGAGGAATTCCCAGTAAGCGCGAGTCATAAGCTCGCGTTGATTACGTCCCTGCCCTTTGTACACACC
GCCCGTCGCTACTACCGATTGAATGATTTAGTGAGGTCTTCGGACC-GGCGCTA--AC-CGGC-ACC--GGC-
----TCGTTCGG---TATCGGT-TTCGCTGGAAA-
GATGACCGAACTTGATCATTTAGAGGAAGTAAAAGTCGTAACAAGG-TTTCCAGTCA--TTGGGTGT---
AAA-CCTAAAGGCGCAATGAAAGT-GTCAG-------------GTGGGACTCCTT-------------
TAGGAGC-----TCCCACCGCAGGGAGGATGGGCCACC----GTACAATTATT--------TGT--
CGGAGCCCCGCACTCCCGGGGCGTCTC--GTTCTC-ATTGCG-AG----
TAGAGACGCACCAGGAGCGCACACGCTGGTACCCGAAAGATGGTGAACTATGCCTGGCCAGGACGAAGCCAGG
GGAAACCCTGGTGGAGGTCC--GAAGCG-ATTCTG--ACGTGCAAATCG-
ATCGTCTGAGCTAGGTATAGGGGCGAAAGACCAATCGAACCATCTTG-TTTTAAATT--
ATATTAAGTCAGGCCTGCTCAGTGATTATA-----TAGA-
ATTAAATAGCCGCAGTAATTTGACTGTGCGAAGGTAGCATAATCATTTGTCTTTTAATTGAAGGCTTGTATGA
ATGGTTTGATGAAACTCTGACTTTCTTCACTGCAAAGATTA-----
GAATTTAATATTTGAGTTAAAAAGCTTAGATATTT-CCATAGGACGAGAAGACCCTATAGAA--TTTTACT--
------ATATATTTAATATTATT-TATTTTAGGT--ATTTT----ATTTTTAAGAATTTTAGATT-
ATAGTTGTGTTGGGGTGACAAAGAAA-TTAATTTAACTTTCTTTATTATTTGATT-TTAATGATTTTTATA--
--TT-AATGATCCTTTA---TT--ATGGATA--GTAAGATTAAATTACCTTAGGGATAAC
Macroscytus_fraterculus
ATGCAAGTCTCAGTACGAGCTGACT-
TAAAGTGAAACCGCAAAAGGCTCAGTAAACCGGTCATAGTTTACCGGA-TATATG-CCACTC-
ACGTGGATAACTGTGGTAATTCTAGAGCTAATACATGCAACC-AAGTCCCGACCAGCG---
ATGGAAAGGACGCTTTTATTGGATCAAAACCATTCGCTCGGCTCGCCCCGGGC------
GTCTGCTTGATGACTCT-AGATAAC-ACCACA-GATCGCACGGTCACAGTACCGGC-
GACGTATCCGTCAAATGTCTGACTTATCAGCTGTCGATGGTAGACTCTGTGTCTACCATGGCGCTCACGGGTA
ACGGGGAATCAGGGTTCGATTCCGGAGAGGGAGCCTGAGAAACGGCTACCACATCCAAGGAAGGCAGCAGGCA
CGCAAATTACCCACTCCCGGCACGGGGAGGTAGTGACAAAAAATAACGATACGGGACTCATCCGAGGCCCCGT
AATCGGAATGAGAACACCCTAAATCCTTTAACGAGGATCTATTGGAGGGCAAGTCTGGTGCCAGCAGCCGCGG
TAATTCCAGCTCCAATAGCGTATATTAAAGTTGTTGCGGTTAAAAA-
GCTCGTAGTTGGTTCTGCGTGCCACGCTGTCGGTTCACCGCCTGTCGGTGCAACTGGCATGCC-

GTGGCATGTCCTGTCGGTGGCTGACAGGC-TTCGCG-GCGCGCCCTATG-------CGTTCAC-GCGTGT---
-AGGTCGTCGC--GCCGCTGTTGTCC-TTGTAGG-CCGGCT-CAATCCTACCGCGGTGCT-
CTTTGCCGAGTGTCGAGGTAGGCCGACACT-
GTTTACTTTGAACAAATTAGAGTGCTTCAAGCAGGCTCTGAAAGC-
AGCCTGAATAGTGGTGCATGGAATAATGGAACAGGACCTTGGTTCTATTTTGTT-GGTTTCCGGA-
ATCCAAGGTAATGATCAATAAGGA-CAGGCGGGGGCATTCGTATTGCGACGTTAGA-----------------
-GGTGAAATTCTTGG-
ATCGTCGCAAGACGCACTAGAGCGAAAGCATTTGCCAAGTATGTCTTAATTGATCAAGAACGAAAGTTAGAGG
TTCGAAGGCGATCAGATACCGCCCTAGTTCTAACCATAAACGATGCCAGCCAGCGATCCGCCGATGTTCCTCC
GATGACTCGGCGGGGAGCTT-
TCCCCGGGAAACCAAAGCTTTTGGGTTCCGGGGGAAGTATGGTTGCAAAGCTGAAACTTAAAGGAATTGACGG
AAGGGCACCACCAGGAGTGGAGCCTGCGGCTTAATTTGACTCAACACGGGAAACCTCACCAGGCCCGGACATT
GGAA-GGATTGACAGAT-CGATAGCTCTTT-CTCGATT-CAGTGGGT-
AGTGGTGCATGGCCGTTCTTAGTTGGTGGACTGATTTGTCTGGTTAATTCCGATAACGAACGAGACTCTGACC
TATTAACTAGGCGCTC-CCGGTATAA-ATA-CCTACCGGCGAGAT-
TTATTCTTCTTAAGGGGACAGGCGGCTCTTAGCCGCACGAGATTGAGCAATAACAGGTCTGTGATGCCCTTAG
ATGTTCTGGGCCGCACGCGCGCTACACTGAAGGAATCAGCGTGTGC--
TCCCCTGTCCGAAAGGACCGGGCAACCCGCTGAACCTCCTTCGTGCTAGGAATTGGGGCTTGCAATTGTTCCC
CATGAACGAGGAATTCCCAGTAAGCGCGAGTCATAAGCTCGCGTTGATTACGTCCCTGCCCTTTGTACACACC
GCCCGTCGCTACTACCGATTGAATGATTTAGTGAGGTCTTCGGACC-GGCGCTA--AC-CGGC-ACC--GGC-
----TCGTTCGG---TATCGGT-TTCGCTGGAAA-
GATGACCGAACTTGATCATTTAGAGGAAGTAAAAGTCGTAACAAGG-TTTCCAGTCA--TTGGGTGT---
AAA-CCTAAAGGCGCAATGAAAGT-GTCAG-------------GTGGGACTCCTT-------------
TAGGAGC-----TCCCACCGCAGGGAGGATGGGCCACC----GTACAATTATT--------TGT--
CGGAGCCCCGCACTCCCGGGGCGTCTC--GTTCTC-ATTGCG-AG----
TGGAGACGCACCAGGAGCGCACACGCTGGTACCCGAAAGATGGTGAACTATGCCTGGCCAGGACGAAGCCAGG
GGAAACCCTGGTGGAGGTCC--GAAGCG-ATTCTG--ACGTGCAAATCG-
ATCGTCTGAGCTAGGTATAGGGGCGAAAGACCAATCGAACCATCTTG-TTA-ATATT--
ATATTAGGTCAAGCCTGCTCAGTGATTAT------TATT-
ATTAAATAGCCGCAGTAATTTGACTGTGCGAAGGTAGCATAATCATTTGTCTTTTAATTGGAGGCTTGTATGA
ATGGTTTGATGAAACTTTAACTTTCTTTGCTATAAATATTA-----
GAATTTAATGTTTGAGTTAAAAAGCTTAGATATTT-CCATAGGACGAGAAGACCCTATAGAG--TTTTACT--
------ATAATTATGAT-TTATT-TAAATTAGGT--ATGTA----TTTTTAAATATTTACTTTTT-
ATATTTATGTTGGGGTGACAGAGAAA-TTAATATAACTTTCTTTATT-TATATTG-TTATTGATGTTTATA--
--TT-ATTGATCCTTTA---TT--ATGGATA--ATAAGATTAAATTACCTTAGGGATAAC
Macroscytus_japonensis
ATGCAAGTCTCAGTACGAGCTGACT-
TAAAGTGAAACCGCAAAAGGCTCAGTAAACCGGTCATAGTTTACCGGA-TATATG-CCACTC-
ACGTGGATAACTGTGGTAATTCTAGAGCTAATACATGCAACC-AAGTCCCGACCAGCG---
ATGGAAAGGACGCTTTTATTGGATCAAAACCATTCGCTCGGCTCGCCCCGGGC------
GTCTGCTTGATGACTCT-AGATAAC-ACCACA-GATCGCACGGTCACAGTACCGGC-
GACGTATCCGTCAAATGTCTGACTTATCAGCTGTCGATGGTAGACTCTGTGTCTACCATGGCGCTCACGGGTA
ACGGGGAATCAGGGTTCGATTCCGGAGAGGGAGCCTGAGAAACGGCTACCACATCCAAGGAAGGCAGCAGGCA
CGCAAATTACCCACTCCCGGCACGGGGAGGTAGTGACAAAAAATAACGATACGGGACTCATCCGAGGCCCCGT
AATCGGAATGAGAACACCCTAAATCCTTTAACGAGGATCTATTGGAGGGCAAGTCTGGTGCCAGCAGCCGCGG
TAATTCCAGCTCCAATAGCGTATATTAAAGTTGTTGCGGTTAAAAA-
GCTCGTAGTTGGTTCTGCGTGCCACGCTGTCGGTTCACCGCCTGTCGGTGCAACTGGCATGCC-
GTGGCATGTCCTGTCGGTGGCTGACAGGC-TTCGCG-GCGCGCCCTATG-------CGTTCAC-GCGTGT---
-AGGTCGTCGC--GCCGCTGTTGTCC-TTGTAGG-CCGGCT-CAATCCTACCGCGGTGCT-
CTTTGCCGAGTGTCGAGGTAGGCCGACACT-
GTTTACTTTGAACAAATTAGAGTGCTTCAAGCAGGCTCTGAAAGC-
AGCCTGAATAGTGGTGCATGGAATAATGGAACAGGACCTTGGTTCTATTTTGTT-GGTTTTCGGA-
ATCCAAGGTAATGATCAATAAGGA-CAGGCGGGGGCATTCGTATTGCGACGTTAGA-----------------
-GGTGAAATTCTTGG-
ATCGTCGCAAGACGCACTAGAGCGAGAGCATTTGCCAAGTATGTCTTAATTGATCAAGAACGAAAGTTAGAGG
TTCGAAGGCGATCAGATACCGCCCTAGTTCTAACCATAAACGATGCCAGCCAGCGATCCGCCGATGTTCCTCC
GATGACTCGGCGGGGAGCTT-
TCCCCGGGAAACCAAAGCTTTTGGGTTCCGGGGGAAGTATGGTTGCAAAGCTGAAACTTAAAGGAATTGACGG

AAGGGCACCACCAGGAGTGGAGCCTGCGGCTTAATTTGACTCAACACGGGAAACCTCACCAGGCCCGGACATT
GGAA-GGATTGACAGAT-CGATAGCTCTTT-CTCGATT-CAGTGGGT-
AGTGGTGCATGGCCGTTCTTAGTTGGTGGACTGATTTGTCTGGTTAATTCCGATAACGAACGAGACTCTGACC
TATTAACTAGGCGCTC-CCGGTATAA-ATA-CCTACCGGCGAGAT-
TTATTCTTCTTAAGGGGACAGGCGGCTCTTAGCCGCACGAGATTGAGCAATAACAGGTCTGTGATGCCCTTAG
ATGTTCTGGGCCGCACGCGCGCTACACTGAAGGAATCAGCGTGTGC--
TCCCCTGTCCGAAAGGTCCGGGCAACCCGCTGAACCTCCTTCGTGCTAGGAATTGGGGCTTGCAATTGTTCCC
CATGAACGAGGAATTCCCAGTAAGCGCGAGTCATAAGCTCGCGTTGATTACGTCCCTGCCCTTTGTACACACC
GCCCGTCGCTACTACCGATTGAATGATTTAGTGAGGTCTTCGGACC-GGCGCTA--AC-CGGC-ACC--GGC-
----TCGTTCGG---TATCGGT-TTCGCTGGAAA-
GATGACCGAACTTGATCATTTAGAGGAAGTAAAAGTCGTAACAGGG-TTTCCAGTCA--TTGGGTGT---
AAAACCTAAAGGCGCAATGAAAGT-GTCAG-------------GTGGGACTCCTT-------------
TAGGAGC-----TCCCACCGCAGGGAGGATGGGCCACC----GTACAATTATT--------TGT--
CGGAGCCCCGCACTCCCGGGGCGTCTC--GTTCTC-ATTGCG-AG----
TGGAGACGCACCAGGAGCGCACACGCTGGTACCCGAAAGATGGTGAACTATGCCTGGCCAGGACGAAGCCAGG
GGAAACCCTGGTGGAGGTCC--GAAGCG-ATTCTG--ACGTGCAAATCG-
ATCGTCTGAGCTAGGTATAGGGGCGAAAGACCAATCGAACCATCTTG-TTA-ATATT--
ATATTAGGTCAAGCCTGCTCAGTGATTAT------TATT-
ATTAAATAGCCGCAGTAATTTGACTGTGCGAAGGTAGCATAATCATTTGTCTTTTAATTGGAGGCTTGTATGA
ATGGTTTGATGAAACTTTAACTTTCTTTGCTATAAATATTA-----
GAATTTAATGTTTGAGTTAAAAAGCTTAGATATTT-CCATATGACGAGAAGACCCTATAGAG--TTTTACT--
------ATAATTATGAT-TTATT-TAAATTAGGT--ATGTA----TTTTTAAATATTTACTTTTT-
ATATTTATGTTGGGGTGACAGAGAAA-TTAATATAACTTTCTCTATT-TATATTG-TTATTGATGTTTATA--
--TT-ATTGATCCTTTA---TT--ATGGATA--ATAAGATTAAATTACCTTATGGATAAC
Macroscytus_minimus
ATGCAAGTCTCAGTACGAGCTGACT-
TAAAGTGAAACCGCAAAAGGCTCAGTAAACCGGTCATAGTTTACCGGA-TATATG-CCACTC-
ACGTGGATAACTGTGGTAATTCTAGAGCTAATACATGCAACC-AAGTCCCGACCAGTG---
ATGGAAAGGACGCTTTTATTGGATCAAAACCATTCGCTCGGCTCGCCCCGGGC------
GTCTGCTTGATGACTCT-AGATAAC-ACCACA-GATCGCACGGTCACAGTACCGGC-
GACGTATCCGTCAAATGTCTGACTTATCAGCTGTCGATGGTAGACTCTGTGTCTACCATGGCGCTCACGGGTA
ACGGGGAATCAGGGTTCGATTCCGGAGAGGGAGCCTGAGAAACGGCTACCACATCCAAGGAAGGCAGCAGGCA
CGCAAATTACCCACTCCCGGCACGGGGAGGTAGTGACAAAAAATAACGATACGGGACTCATCCGAGGCCCCGT
AATCGGAATGAGAACACCCTAAATCCTTTAACGAGGATCTATTGGAGGGCAAGTCTGGTGCCAGCAGCCGCGG
TAATTCCAGCTCCAATAGCGTATATTAAAGTTGTTGCGGTTAAAAA-
GCTCGTAGTTGGTTCTGCGTGCCACGCTGTCGGTTCACCGCCTGTCGGTGCAACTGGCATGCC-
GTGGCATGTCCTGTCGGTGGCTGACAGGC-TTCGCG-GCGCGCCCTATG-------CGTTCAC-GCGTGT---
-AGGTCGTCGC--GCCGACGTTGTCC-TTGTAGG-CCGACT-CAATCCTACCGCGGTGCT-
CTTTGCCGAGTGTCGAGGTAGGCCGACACT-
GTTTACTTTGAACAAATTAGAGTGCTTCAAGCAGGCTCTGAAAGC-
AGCCTGAATAGTGGTGCATGGAATAATGGAACAGGACCTTGGTTCTATTTTGTT-GGTTTCCGGA-
ATCCAAGGTAATGATCAATAAGGA-CAGGCGGGGGCATTCGTATTGCGACGTTAGA-----------------
-GGTGAAATTCTTGG-
ATCGTCGCAAGACGCACTAGAGCGAAAGCATTTGCCAAGTATGTCTTAATTGATCAAGAACGAAAGTTAGAGG
TTCGAAGGCGATCAGATACCGCCCTAGTTCTAACCATAAACGATGCCAGCCAGCGATCCGCCGATGTTCCTCC
GATGACTCGGCGGGGAGCTT-
TCCCCGGGAAACCAAAGCTTTTGGGTTCCGGGGGAAGTATGGTTGCAAAGCTGAAACTTAAAGGAATTGACGG
AAGGGCACCACCAGGAGTGGAGCCTGCGGCTTAATTTGACTCAACACGGGAAACCTCACCAGGCCCGGACATT
GGAA-GGATTGACAGAT-CGATAGCTCTTT-CTCGATT-CAGTGGGT-
AGTGGTGCATGGCCGTTCTTAGTTGGTGGACTGATTTGTCTGGTTAATTCCGATAACGAACGAGACTCTGACC
TATTAACTAGGCGCTC-CCGGTATAA-ATA-CCTACCGGCGAGAT-
TTATTCTTCTTAAGGGGACAGGCGGCTCTTAGCCGCACGAGATTGAGCAATAACAGGTCTGTGATGCCCTTAG
ATGTTCTGGGCCGCACGCGCGCTACACTGAAGGAATCAGCGTGTGC--
TCCCCTGTCCGAAAGGACCGGGCAACCCGCTGAACCTCCTTCGTGCTAGGAATTGGGGCTTGCAATTGTTCCC
CATGAACGAGGAATTCCCAGTAAGCGCGAGTCATAAGCTCGCGTTGATTACGTCCCTGCCCTTTGTACACACC
GCCCGTCGCTACTACCGATTGAATGATTTAGTGAGGTCTTCGGACC-GGCGGTA--AC-CGGC-ACC--GGC-
----TCGTTCGG---TATCGGT-TTCGCTGGAAA-
GATGACCGAACTTGATCATTTAGAGGAAGTAAAAGTCGTAACAAGG-TTTCCAGTCA--TTGGGTTT---

AAA-CCTAAAGGCGCAATGAAAGT-GTCAG-------------GTGGGACTCCTT-------------
TAGGAGC-----TCCCACCGCAGGGAGGATGGGCCACC----GTACAATTATT--------TGT--
CGGAGCCCCGCACTCCCGGGGCGTCTC--GTTCTC-ATTGCG-AG----
TAGAGACGCACCAGGAGCGCACACGCTGGTACCCGAAAGATGGTGAACTATGCCTGGCCAGGACGAAGCCAGG
GGAAACCCTGGTGGAGGTCC--GAAGCG-ATTCTG--ACGTGCAAATCG-
ATCGTCTGAGCTAGGTATAGGGGCGAAAGACCAATCGAACCATCTTG-AATAAGA----
ATATTAGGTCAGGCCTGCTCAATGATTAAT-----
TATTGATTAAATAGCCGCAGTAATTTGACTGTGCGAAGGTAGCATAATCATTTGTCTTTTAATTGGAGGCTTG
TATGAATGGTTTGATGAAGTACCTACTTTCTTTACTATAAAGGTTA-----
AAATTTAATATTTGAGTTAAAAAGCTTAGATATTT-CCATAGGACGAGAAGACCCTATAGAG--TTTTACT--
------GTATTGGTGAA-TTACT-T-TATAAGGTA-ATGT-----ATTTTAAGGAGTTTACACTA-
TCAGTTATGTTGGGGTGACAAAGGAA-TTTTAATAACTTCCTTTATTAGATG-TT-TTATTGATATATATG--
--TA-TATGATCCTTTA---TT--ATGGATA--TTAAGATTAAATTACCTTAGGGATAAC
Megacopta_sp._cribraria ATGCAAGTCTCAGTACGAGCTGTAT-
TAAAGTGAAACCGCAAAAGGCTCAGTAAACCGGTCATAGTTTACCGGG-TATATG-CCACTT-
AGGTGGATAACTGTGGTAATTCTAGAGCTAATACATGCAACC-AAGTCCCGACCAGTG---
ATGGAAAGGACGCTTTTATTGGATCAAAACCATTCGCCCCGGTCCGCCGGGGC------
GTCTGCTTGATGACTCT-AGATAAC-ACCACA-GATCGCACGGTCACAGTACCGGC-
GACGTATCCGTCAAATGTCTGACTTATCAGCTGTCGATGGTAGACTCTGTGTCTACCATGGCGCTCACGGGTA
ACGGGGAATCA?GGTTCGATTCCGGAGAGGGAGCCTGAGAAACGGCTACCACATCCAAGGAAGGCAGCAGGCA
CGCAAATTACCCACTCCCGGCACGGGGAGGTAGTGACAAAAAATAACGAT-CGGGA-
TCATCCGAGGCCCCGTAATC?GAATGAGAACACCCTAAATCCTTTAACGAGGATCTATTGGAGGGCAAGTCTG
GTGCCAGCAGCCGCGATAATTCCAGCTCCAATAGCGTATATTAAAGTTG?TGCGGTTAAAAA-
GCTCGTAGTTGGTTCTGCGTGCCACGCTGTCGGTTCACCGCCTGTCGGTGCAACTGGCATGCC-
GTGGCATGTCCTGTCGGTGGCCGACAGGC-TTCGCGGCG-CGCCCTATT-------CGTTCACA-CGTGT---
-AGGTCGTCGC--GCCG-AGTTGTCC-TCG-TAGGCCGGCTTCAATCCTACCGCGGTGCT-
CTTTACCGAGTGTCGAAGTACGCCGACACT-
GTTTACTTTGAACAAATTAAAGTGCTTAAAGCAGGCTTTGAAAGC-
AGCCTGAATAGTGGTGCATGGAATAATGGAACAGGACCTTGGTTCTATTTTGTGAGGTTTTCTGACATCCAAG
GTAATGGTCAATAAGGA-CAGGCGGGGGCATTCGTATTGCGACGTTAGA------------------
GGTGAAATTCTTGG-
ATCGTCGCAAGACGCATTAGAGCGAAAGCATTTGCCAAGTATGTCTTAATTGATCAAGAACGAAAGTTAGAGG
TTCGAAGGCGATCAGATACCGCCCTAGTTCTAACCATAAACGATGCCAGCCAGCGATCCGCCGATGTTCCTCC
GATGACTCGGCGGGGAGCTT-
TCCCCGGGAAACCAAAGCTTTTGGGTTCCGGGGGAAGTATGGTTGCAAAGCTGAAACTTAAAGGAATTGACGG
AGGGGCACCACCAGGAGTGGAGCCTGCGGCTTAATTTGACTCAACACGGGAAACCTCACCAGGCCCGGACATT
GGAA-GGATTGACAGAT-CGATAGCTCTTT-CTCGATT-CAGTGGGT-
AGTGGTGCATGGCCGTTCTTAGTTGGTGGACTGATTTGTCTGGTTAATTCCGATAACGAACGAGACTCTGACC
TATTAACTAGGCGCTC-CCGGTATAA-ATG-CCTACCGGCGAGAT-
TTTATCTTCTTAAGGGGACAGGCGGCTCTTAGCCGCACGAGATTGAGCAATAACAGGTCTGTGATGCCCTTAG
ATGTTCTGGGCCGCACGCGCGCTACACTGAAGGAATCAGCGTGTTC--
TCCCCTGGCCGGAAGGTCCGGGCAACCCGCTGAACCTCCTTCGTGCTAGGAATTGGGGCTTGCAATTGTTCCC
CATGAACGGGGAATTCCCAGTAAGCGCGAGTCATAAGCTCGCGTTGATTACGTCCCTGCCCTTTGTACACACC
GCCCGTCGCTACTACCGATTGAATGATTTAGTGAGGTCTTCGGACC-GGCGGTA--AC-CGGC-ACC--GG--
---CTTGTTCGG---TATCGGT-TTCGCTGGAAA-
GATGACCGAACTTGATCATTTAGAGGAAGTAAAAGTCGTAACAAGG-TTTCCAGTCA--TTGGGTTCA-
TAAAACCTAAAGGCGCAATGAAAGT-GTCAG------------GGCGGGTCTCCTC------------CGGG-
-AG----GCCCCCCCGCTGGGAAGATGGGCACTCC---GCACGTT-CTA-C------CGT--
CGGGGCCCCGCACTCCCGGGGCGTCTC--TACCTCTCTCA-G-AG----
CGGAGACGCACCAGGAGCGCACACGCTGGTACCCGAAAGATGGTGAACTATGCCTGGCCAGGACGAAGCCAGG
GGAAACCCTGGTGGAGGTCC--GAAGCG-ATTCTG--ACGTGCAAATCG-
ATCGTCTGAGCTGGGTATAGGGGCGAAAGACCAATCGAACCATCTT---GTTTATTT--
ATTTTAGGTCTGGCCTGCTCAATGATTATTATTT--------
TAAATAGCCGCAGTATTTTGACTGTGCAAAGGTAGCATAATCATTTGTCTCTTAATTGGGGGCTTGTATGAAT
GGTTTGATAAGGATTCGACTTTCTTTA---CTTAATATTAA--
TGAATTTAATTTTTGGGTTAAAAAGCTCAAATATTT-TTATGGGACGAGAAGACCCTATAGAA--ATTTA-
CTCTATTTTT----------GGTGTTTATTTTTATTCT--TTTTG----GAATTTTTATCATTACTAG---

AGTTTTGTTGGGGTGACATGAAGA-TTGTTATAACTCTTTGTTATTACTTT---TTATTTATTTATATAC---
--AACTGATCCTT-----TTTTATGGATT--AAAAGATTAATTTACCTTAGGGATAAC
Megymenum_sp._brevicorne
ATGCAAGTCTCAGTACGAGCTTAAT-
TAAAGTGAAACCGCAAAAGGCTCAGTAAACCGGTCATAGTTTACCGGG-TATATG-CCACTC-
ACGTGGATAACTGTGGTAATTCTAGAGCTAATACATGATACC-AAGTCCCGACCAGAG---
ATGGAAAGGACGATTTTATTGGATCAAAACCATTCGCCCGGCTTGTCC-GGGC------
GTCTTCTTGACGAGTCT-AGATAAC-AAAACA-GATCGCACGGTCACAGTACCGGC-
GACGTATCCGTCAAATGTCTGACTTATCAGCTGTCGATGGTAGACTCTGTGTCTACCATGGCGCTCACGGGTA
ACGGGGAATCAGGGTTCGATTCCGGAGAGGGAGCCTGAGAAACGGCTACCACATCCAAGGAAGGCAGCAGGCA
CGCAAATTACCCACTCCCGGCACGGGGAGGTAGTGACAAAAAATAACGATACGGGACTCATCCGAGGCCCCGT
AATCGGAATGAGAACACCCTAAATCCTTTAACGAGGATCCATTGGAGGGCAAGTCTGGTGCCAGCAGCCGCGG
TAATTCCAGCTCCAATAGCGTATATTAAAGTTGTTGCGGTTAAAAA-
GCTCGTAGTTGGTTCTGCGTGCCACGCTGTCGGTTCACCGCCTGTCGGTGCAACTGGCATGCC-
GTGGCATGTCCTGTCGGTGGCCGACAGGC-TTCACG-GGGCGCCCTATG-------CGCTTCTAGCGCGT---
-AGGTAGTTGC--TCCG-AGTTGTCC-TTGTAGG-CCGGCT-CAATCCTACCGCGGTGCT-
CTTTGCCGAGTGTCGAGGTAGGCCGACACT-
GTTTACTTTGAACAAATTAGAGTGCTTAAAGCAGGCTCTGAAAGC-
AGCCTGAATAGTGGTGCATGGAATAATGGAACAGGACCTTGGTTCTATTTTGTT-GGTTTCCGGA-
ATCCAAGGTAATGATCAATAAGGA-CAGGCGGGGGCATTCGTATTGCGACGTTAGA-----------------
-GGTGAAATTCTTGG-
ATCGTCGCAAGACGCACTAGAGCGAAAGCATTTGCCAAGTATGTCTTAATTGATCAAGAACGAAAGTTAGAGG
TTCGAAGGCGATCAGATACCGCCCTAGTTCTAACCATAAACGATGCCAGCCAGCGATCCGCCGATGTTCCTCC
GATGACTCGGCGGGGAGCTT-
TCCCCGGGAAACCAAAGCTTTTGGGTTCCGGGGGAAGTATGGTTGCAAAGCTGAAACTTAAAGGAATTGACGG
AAGGGCACCACCAGAAGTGGAGCCTGCGGCTTAATTTGACTCAACACGGGAAACCTCACCAGGCCCGGACATT
GGAA-GGATTGACAGAT-CGATAGCTCTTT-CTCGATT-CAGTGGGT-
AGTGGTGCATGGCCGTTCTTAGTTGGTGGACTGATTTGTCTGGTTAATTCCGATAACGAACGAGACTCTAACC
TATTAACTAGGCGTTG-CCGGTATAA-AAC-GCTACCGGCGAGAT-
TTATTCTTCTTAAGGGGACAGGCGGCTCTTAGCCGCACGAGATTGAGCAATAACAGGTCTGTGATGCCCTTAG
ATGTTCTGGGCCGCACGCGCGCTACACTGAAGGAATCAGCGTGTGC--
ATCCCTGTCCGAAAGGTCCGGGTAACCCGCTGAACCTCCTTCGTGCTAGGAATTGGGGCTTGCAATTGTTCCC
CATGAACGAGGAATTCCCAGTAAGCGCGAGTCATAAGCTCGCGTTGATTACGTCCCTGCCCTTTGTACACACC
GCCCGTCGCTACTACCGATTGAATGATTTAGTGAGGTCTTCGGACC-GGCGATA--AC-CGGC-ACC--GAT-
----TT-TTCGG---TATCGAT-TTCGCTGGAAA-
GATGACCGAACTTGATCATTTAGAGGAAGTAAAAGTCGTAACAAGG-TTTCCAGTCA--CTGGGTGC---
AAA-CCTAAAGGCGCAATGAAAGT-GTCACA-------------GGGTGGGCCAT-------------CTGG-
-------TCCCACCCCAGGGAGGATGGGCACCC----GTACGGCTTGCT-------CGT--
CGGGGCCCCGCACTCCCGGGGCGTCTC--GTTCTC-ATCACG-AG----
TAGAGACGCACCAGGAGCGCACACGATGGTACCCGAAAGATGGTGAACTATGCCTGGCCAGGACGAAGCCAGG
GGAAACCCTGGTGGAGGTCC--GAAGCG-ATTCTG--ACGTGCAAATCG-
ATCGTCTGAGCTGGGTATAGGGGCGAAAGACCAATCGAACCATCT--TGGATATTT---
AAATCAGGTCTAACCTGCCCACTGA---------
TAGTTAATTGAAGGGCCGCAGTAATTTGACTGTGCGAAGGTAGCATAATCATTTGTCTATTAATTGTAGGCTT
GTATGAATGGTTTGATGAGATACTGGCTTTCTTTATAT-AAAAGGA-----
TGAATTTAATTTTTAAGTTAAAAAGCTTAAATAGTA-TTGGAGGACGAGAAGACCCTATAGAA--
TTTTATTA---------TTATATTATTTTATCTTTTTTTGGATGTTTAATG-----
ATTAAATTTAAGATAATAAATAATTGTGTTGGGGCGATATGGAAA-TATTATGAACTTTCTTTATT-TAT-
TAT-TTATTGATTTGTATT-----TTAAGGATCCTCTT---TT--TGGGATT--
ATAAGATTAAATTACCTTAGGGATAAC
Microporus_nigrita
ATGCAAGTCTCAGTACGAGCTGACT-
TAAAGTGAAACCGCAAAAGGCTCAGTAAACCGGTCATAGTTTACCGGA-TATATG-CCACTC-
ACGTGGATAACTGTGGTAATTCTAGAGCTAATACATGCAACC-AAGTCCCGACCAGCG---
ATGGAAAGGACGCTTTTATTGGATCAAAACCATTCGCCCGGCTCGCCC-GGGC------
GTCTGCTTGATGACTCT-AGATAAC-ACCACA-GATCGCACGGTCACAGTACCGGC-
GACGTATCCGTCAAATGTCTGACTTATCAGCTGTCGATGGTAGACTCTGTGTCTACCATGGCGCTCACGGGTA
ACGGGGAATCAGGGTTCGATTCCGGAGAGGGAGCCTGAGAAACGGCTACCACATCCAAGGAAGGCAGCAGGCA

CGCAAATTACCCACTCCCGGCACGGGGAGGTAGTGACAAAAAATAACGATACGGGACTCATCCGAGGCCCCGT
AATCGGAATGAGAACACCCTAAATCCTTTAACGAGGATCTATTGGAGGGCAAGTCTGGTGCCAGCAGCCGCGG
TAATTCCAGCTCCAATAGCGTATATTAAAGTTGTTGCGGTTAAAAA-
GCTCGTAGTTGGTTCTGCGTGCCACGCTGTCGGTTCACCGCCTGTCGGTGTAACTGGCATGCC-
GTGGCATGTCCTGTCGGTGGCTGACAGGC-TTCGCG-GCGCGCCCTATG-------CGTTTAC-GCGTGT---
-AGGTCGTCGC--GCCGCCGTTGTCC-TTGTAGG-CCGGCT-CAATCCTACCGCGGTGCT-
CTTTGCCGAGTGTCGAGGTAGGCCGACACT-
GTTTACTTTGAACAAATTAGAGTGCTTCAAGCAGGCTCTGAAAGC-
AGCCTGAATAGTGGTGCATGGAATAATGGAACAGGACCTTGGTTCTATTTTGTT-GGTTTCCGGA-
ATCCAAGGTAATGATCAATAAGGA-CAGGCGGGGGCATTCGTATTGCGACGTTAGA-----------------
-GGTGAAATTCTTGG-
ATCGTCGCAAGACGCACTAGAGCGAAAGCATTTGCCAAGTATGTCTTAATTGATCAAGAACGAAAGTTAGAGG
TTCGAAGGCGATCAGATACCGCCCTAGTTCTAACCATAAACGATGCCAGCCAGCGATCCGCCGATGTTCCTCC
GATGACTCGGCGGGGAGCTT-
TCCCCGGGAAACCAAAGCTTTTGGGTTCCGGGGGAAGTATGGTTGCAAAGCTGAAACTTAAAGGAATTGACGG
AAGGGCACCACCAGGAGTGGAGCCTGCGGCTTAATTTGACTCAACACGGGAAACCTCACCAGGCCCGGACATT
GGAA-GGATTGACAGAT-CGATAGCTCTTT-CTCGATT-CAGTGGGT-
AGTGGTGCATGGCCGTTCTTAGTTGGTGGACTGATTTGTCTGGTTAATTCCGATAACGAACGAGACTCTGACC
TATTAACTAGGCGCTC-CCGGTATAA-ATA-CCTACCGGCGAGAT-
TTATTCTTCTTAAGGGGACAGGCGGCTCTTAGCCGCACGAGATTGAGCAATAACAGGTCTGTGATGCCCTTAG
ATGTTCTGGGCCGCACGCGCGCTACACTGAAGGAATCAGCGTGTGC--
TCCCCTGTCCGAAAGGACCGGGCAACCCGTTGAACCTCCTTCGTGCTAGGAATTGGGGCTTGCAATTGTTCCC
CATGAACGAGGAATTCCCAGTAAGCGCGAGTCATAAGCTCGCGTTGATTACGTCCCTGCCCTTTGTACACACC
GCCCGTCGCTACTACCGATTGAATGATTTAGTGAGGTCTTCGGACC-GGCGGTA--AC-CGGC-ACC--GGC-
----TCGTTCGG---TATCGGT-TTCGCTGGAAA-
GATGACCGAACTTGATCATTTAGAGGAAGTAAAAGTCGTAACAAGG-TTTCCAGTCA--TTGGGTGA---
AAA-CCTAAAGGCGCAATGAAAGT-GTCGG-------------GTGGGGCTCCTT-------------
TTGGAGT-----TTCCACCGAGGGGAGGATGGGCCACC----GTACGCTAACT---------GT--
CGGAGCCCCGCACTCCCGGGGCGTCTC--GTTCTC-ATCACG-AG----
TAGAGACGCACCAGGAGCGCACACGCTGGTACCCGAAAGATGGTGAACTATGCCTGGCCAGGACGAAGCCAGG
GGAAACCCTGGTGGAGGTCC--GAAGCG-ATTCTG--ACGTGCAAATCG-
ATCGTCTGAGCTGGGTATAGGGGCGAAAGACCAATCGAACCATCTTG-TATAAGT----
TTATTAAGTCAGGCCTGCTCAATGATTAT------
AATTAATTAAATAGCCGCAGTATTTTGACTGTGCGAAGGTAGCATAATCATTTGTCTTTTAATTGTGGGCTTG
TATGAATGGTTTGATGAGATATTAACTTTCTTTATTCAATATTTAC-----
GAAATTAATACTTGAGTTAAAAAGCTTAAATGTTT-TCGTGGGACGAGAAGACCCTATAGAG--TTTTATA--
------ATTATAAT-TATTAAATTATTCATTGGT--AAGTT----TCATTAATTCAATAGTTTAA--
TTATTTTGTTGGGGTGACGGGAAAA-TTTTATTAACTTTTCTTTAT-TTTATAT-TAA-TAATTATTATGT--
TTAGAATGATCCTTTA---TT--AAGGATA--ATAAGATTAAATTACCTTAGGGATAAC
Microporus_pallidipennis
ATGCA-GTCTCAGTACGAGCTGACT-
TAAAGTGAAACCGCAAAAGGCTCAGTAAACCGGTCATAGTTTACCGGA-TATATG-CCACTC-
ACGTGGATAACTGTGGTAATTCTAGAGCTAATACATGCAACC-AAGTCCCGACCAGCG---
ATGGAAAGGACGCTTTTATTGGATCAAAACCATTCGCCCGGCTCGCCC-GGGC------
GTCTGCTTGATGACTCT-AGATAAC-ACCACA-GATCGCACGGTCACAGTACCGGC-
GACGTATCCGTCAAATGTCTGACTTATCAGCTGTCGATGGTAGACTCTGTGTCTACCATGGCGCTCACGGGTA
ACGGGGAATCAGGGTTCGATTCCGGAGAGGGAGCCTGAGAAACGGCTACCACATCCAAGGAAGGCAGCAGGCA
CGCAAATTACCCACTCCCGGCACGGGGAGGTAGTGACAAAAAATAACGATACGGGACTCATCCGAGGCCCCGT
AATCGGAATGAGAACACCCTAAATCCTTTAACGAGGATCTATTGGAGGGCAAGTCTGGTGCCAGCAGCCGCGG
TAATTCCAGCTCCAATAGCGTATATTAAAGTTGTTGCGGTTAAAAA-
GCTCGTAGTTGGTTCTGCGTGCCACGCTGTCGGTTCACCGCCTGTCGGTGCAACTGGCATGCC-
GTGGCATGTCCTGTCGGTGGCTGACAGGC-TTCGCG-GCGCGCCCTATG-------CGTTTAC-GCGTGT---
-AGGTTGTCGC--GCCGACGTTGTCC-TTGTAGG-CCGGCT-CAATCCTACCGCGGTGCT-
CTTTGCCGAGTGTCGAGGTAGGCCGACACT-
GTTTACTTTGAACAAATTAGAGTGCTTCAAGCAGGCTCTGAAAAT-
AGCCTGAATAGTGGTGCATGGAATAATGGAACAGGACCTTGGTTCTATTTTGTT-GGTTTCCGGA-
ATCCAAGGTAATGATCAATAAGGA-CAGGCGGGGGCATTCGTATTGCGACGTTAGA-----------------
-GGTGAAATTCTTGG-

ATCGTCGCAAGACGCACTAGAGCGAAAGCATTTGCCAAGTATGTCTTAATTGATCAAGAACGAAAGTTAGAGG
TTCGAAGGCGATCAGATACCGCCCTAGTTCTAACCATAAACGATGCCAGCCAGCGATCCGCCGATGTTCCTCC
GATGACTCGGCGGGGAGCTT-
TCCCCGGGAAACCAAAGCTTTTGGGTTCCGGGGGAAGTATGGTTGCAAAGCTGAAACTTAAAGGAATTGACGG
AAGGGCACCACCAGGAGTGGAGCCTGCGGCTTAATTTGACTCAACACGGGAAACCTCACCAGGCCCGGACATT
GGAA-GGATTGACAGAT-CGATAGCTCTTT-CTCGATT-CAGTGGGT-
AGTGGTGCATGGCCGTTCTTAGTTGGTGGACTGATTTGTCTGGTTAATTCCGATAACGAACGAGACTCTGACC
TATTAACTAGGCGCTC-CCGGTATAA-ATA-CCTACCGGCGAGAT-
TTATTCTTCTTAAGGGGACAGGCGGCTCTTAGCCGCACGAGATTGAGCAATAACAGGTCTGTGATGCCCTTAG
ATGTTCTGGGCCGCACGCGCGCTACACTGAAGGAATCAGCGTGTGC--
TCCCCTGGCCGAAAGGTCCGGGCAACCCGCTGAACCTCCTTCGTGCTAGGAATTGGGGCTTGCAATTGTTCCC
CATGAACGAGGAATTCCCAGTAAGCGCGAGTCATAAGCTCGCGTTGATTACGTCCCTGCCCTTTGTACACACC
GCCCGTCGCTACTACCGATTGAATGATTTAGTGAGGTCTTCGGACC-GGCGGTA--AC-CGGC-ACC--GGC-
----TCGTTCGG---TATCGGT-TTCGCTGGAAA-
GATGACCGAACTTGATCATTTAGAGGAAGTAAAAGTCGTAACAAGG-TTTCCAGTCA--TTGGGTGC---
AAA-CCTAAAGGCGCAATGAAAGT-GTCAG-------------GTGAGGCTCCTT-------------
TAGGAGT-----CCCCACCGCAGGGAGGATGGCCTCC----GTACAATTATT--------TGT--
CGGATCCCCGCACTCCCGGGGCGTCTC--GTTCTC-ATCACG-AG----
TGGAGACGCACCAGGAGCGCACACGCTGGTACCCGAAAGATGGTGAACTATGCCTGGCCAGGACGAAGCCAGG
GGAAACCCTGGTGGAGGTCC--GAAGCG-ATTCTG--ACGTGCAAATCG-
ATCGTCTGAGCTGGGTATAGGGGCGAAAGACCAATCGAACCATCTTG-TGTAGTT----
ATATTAAGTCAGGCCTGCTCAATGATTA-------
GTTTAATTAAATAGCCGCAGTATTTTGACTGTGCGAAGGTAGCATAATCATTTGTCTTTTAATTGTAGGCTAG
TATGAAGGGTTTGATGAAGTTTATACTTTCTTTGTTTTAATATTAA-----
TAATTTAATGTTTAAGTTAAAAGGCTTAAATTTAT-TCGGAGGACGAGAAGACCCTATAGAAATTTTTATT--
------ATTACTTA-GTAAAGTTTACTTTTGGTT--TTATG----TTGTTTAATCTTTATTATTA-
ATAATTGTGTTGGGGTGACAGAGGGG-TTTTTTTAACCCCCTTTATT-AAT-TAT-TAT-
TGTTGATTATCATGTTTAT-TGATCCTTGG---TTTCGAAGATT--ATAAGATTAATTTACCTTAGGGATAAC
Neolethaeus_assamensis
ATGCAAGTCTCAGTACAAGCTGAAC-
AAAAGTGAAACCGCAAAAGGCTCAGTAAACCAATCATAGTTTACAGGA-TATATG-CCACTC-
ACGTGGATAACTGTGGTAATTCTAGAGCTAATACATGCAAAC-AAGTCCCGACCAGCG---
ATGGGGAGGACGCTTTTATTGGATCAAAACCATTCGCCCGGCTCGCCC-GGGC------
GTCGGCTTGATGACTCT-AGATAAC-ACCACA-GATCGCACGGTCCCAGAACCGGT-
GACATATCCTTCAAATGTCTGACTTATCAGCTGTCGATGGTAGACTCTATGTCTACCATGGCGCTCACGGGTG
ACGGGGAATCAGGGTTCGATTCCGGAGAGGGAGCCTGAGAAACGGCTACCACATCCAAGGAAGGCAGCAGGCA
CGCAAATTACCCACTCCCGGCACGGGGAGGTAGTGACAAAAAATAACGATACGGGACTCATCCGAGGCCCCGT
AATCGGAATGAGAACACCCTAAATCCTTTAACGAGGATCTATTGGAGGGCAAGTCTGGTGCCAGCAGCCGCGG
TAATTCCAGCTCCAATAGCGTATATTAAAGTTGTTGCGGTTAAAAA-
GCTCGTAGTTGGTTCTGCGTGCCACGCTGTCGGTTCACCGCCTGCCGGTGCAACTGGCATGCC-
GTGGCATGTCCTGTTGGTGGCGGATAGGC-TTCCCG-GCGTGCCCTATGCGG----TAT-ACCAACGTGT---
-AGGCTGTCGC--GTCGG-GTTGTCC-TTGTACG-CCGGCT-CAATCCTACCGCGGTGCT-
CTTTACCGAGTGTCGAGGTAGGCCGACACT-
GTTTACTTTGAACAAATTAGAGTGCTTAAAGCAGGCTCTTAAATC-
AGCCTGAATAGTGGTGCATGGAATAATGGAACAGGACCTTGGTTCTATTTTGTT-GGTTTTAGGA-
GTCCAAGGTAATGATCAATAAGGA-CAGGCGGGGGCATTCGTATTGCGACGTTAGA-----------------
-GGTGAAATTCTTGG-
ATCGTCGCAAGACGCACTAGAGCGAAAGCAATTGCCAAGTATGTCTTAATTGATCAAGAACGAAAGTTAGAGG
TTCGAAGGCGATCAGATACCGCCCTAGTTCTAACCATAAACGATGCCAGCCAGCGATCCGCCGACGTTCCTCC
GATGACTCGGCGGGGAGCTT-
CCCCCGGGAAACCAAAGCTTTTGGGTTCCGGGGGAAGTATGGTTGCAAAGCTGAAACTTAAAGGAATTGACGG
AAGGGCACCACCAGGAGTGGGGCATGCGGCTTAATTTGACTCAACACGGGAAACCTCACCAGGCCCGGACATT
GGAA-GGATTGACAGAT-CGATAGCTCTTT-CTCGATT-CAGTGGGT-
AGTGGTGCATGTCCGTTCTTAGTTCGTGGAATGATTTGTCTGGTTAATTCCGATAACGAACGAGACTCTAGCC
TATTAACTAGGCGATG-CCGGTATACC-TA-GCTACCGGCGAGAT-
TTATTCTTCTTAAGGGGACAGGCGGCTCTTAGCCGCACGAGATTGAGCAATAACAGGTCTGTGATGCCCTTAG
ATGTTCTGGGCCGCACGCGCGCCACACTGAAGGAATCAGCGTGTGC--
TCCCCTGGCCGAAAGGTCCGGGTAACCCGCTGAACCTCCTTCGTGCTAGGAATTGGGGCTTGCAATTGTTCCC

CATGAACGAGGAATTCCCAGTAAGCGCGAGTCATAAGCTCGCGTTGATTACGTCCCTGCCCTTTGTACACACC
GCCCGTCGCTACTACCGATTGAATGATTTAGTGAGGTCTTCGGGCC-GGCGATA--AC-CGAC-
ACACAGACTC-----GTTCTG---TGTCGAT-TTCGCTGGAAA-
GATGACCGAACTTGATGATTTAGAGGAAGTAAAAGTCGTAACAAGG-TTTCCAGTCA--TTGGGTGG---
AAAACCTAAAGGCGTAATGAAAGT-GTCAG---------------CGAACGCTCTA--------------
CGCGC--------TCGCAGTGGGAGGATGGGCTTTCC---GGCCTTGCGGC-------------
CGGTGCCCCGCACTCCCGGGGCGTCTC--ATTCTC-GTTTCG-AG----
TTGAGACGCACCAGGAGCGCACACGCTGGTACCCGAAAGATGGTGAACTATGCCTGGCCAGGACGAAGCCAGG
GGAAACCCTGGTGGAGGTCC--GAAGCG-ATTCTG--ACGTGCAAATCG-
ATCGTCTGAGCTGGGTATAGGGGCGAAAGACCAATCGAACCATCTAG----
ATTTATTAATTTTAGGTCTGACCTGCCCAATGATGGAAAG---------
TTTAATGGCCGCAGTATTTTAACTGTGCAAAGGTAGCATAATCATTTGTCTCTTAATTGGAGGCTTGTATGAA
AGGTTGGACGAGAGATATTCTTTCTTTATTTAA-TATAAA----
TTAATTTGATTTTTCAGTTAAAAAGCTGAGATCTTT-TTGTGGGACGAGAAGACCCTATAGAA--
TTTTATTA----------TTTATTCTTATTTATTTTATTT--TTGTTTTTA---
ATATATTACTTAATTATTAATAA--TTTGGTTGGGGTGATTGTGAGA-
TTAAATTAACTCTTATTTACTTTTTG---TCATTAATTAGTGTTTA----T-TTGATCCTTTG---TTAT--
GGATT--TCAAGATTAAATTACCTTAGGGATAAC
Ochetostethomorpha_secunda
ATGCA-GTCTCAGTACGAGCTTACT-
TTAAGTGAAACCGCAAAAGGCTCAGTAAACCGGTCATAGTTTACCGGA-TAAA---CCACTT-
ACGTGGATAACTGTGGTAATTCTAGAGCTAATACATGCAATC-AAGTCCCGACCAGAG---
ATGGAAAGGACGCTTTTATTGGATCAAAACCATTCGCCAGGTTCGCCC-TGGC------
GTCTGCTTGATGACTCT-AGATAACCACAACCAGATCGCACGGTCCTTGTACCGGC-
GACGTATCCGTCAAATGTCTGACTTATCAGCTGTCGATGGTAGACTCTGTGTCTACCATGGCGCTCACGGGTA
ACGGGGAATCAGGGTTCGATTCCGGAGAGGGAGCCTGAGAAACGGCTACCACATCCAAGGAAGGCAGCAGGCA
CGCAAATTACCCACTCCCGGCACGGGGAGGTAGTGACAAAAAATAACGATACGGGACTCATCCGAGGCCCCGT
AATCGGAATGAGAACACCCTAAATCCTTTAACGAGGATCTATTGGAGGGCAAGTCTGGTGCCAGCAGCCGCGG
TAATTCCAGCTCCAATAGCGTATATTAAAGTTGTTGCGGTTAAAAA-
GCTCGTAGTTGGTTCTGCGTGCCACGCTGTCGGTTCACCGCCTGTCGGTGCAACTGGCATGCC-
GTGGCATGTCCTGTCGGTGGCCTACTGGT-TTCGCG-GCGCACCCTATG-------CGTT---CACACGT-G-
TGGGTCGTTGC--ACTGC-GTTGTCC-TAGTAGG-CCGGCT-CAATCCTACCGCGGTGCT-
CTTTACCGAGTGTCGAGGTAGGCCGACACT-
GTTTACTTTGAACAAATTAGAGTGCTTCAAGCAGGCTCTGAAAGC-
AGCCTGAATAGTGGTGCATGGAATAATGGAACAGGACCTTGGTTCTATTTTGTT-GGTTTTCGGA-
ATCCAAGGTAATGATCAATAAGGA-CAGGCGGGGGCATTCGTATTGCGACGTTAGA-----------------
-GGTGAAATTCTTGG-
ATCGTCGCAAGACGCACTAGAGCGAAAGCATTTGCCAAGTATGTCTTAATTGATCAAGAACGAAAGTTAGAGG
TTCGAAGGCGATCAGATACCGCCCTAGTTCTAACCATAAACGATGCCAGCCAGCGATCCGCCGATGTTCCTCC
GATGACTCGGCGGGGAGCTT-
CCCCCGGGAAACCAAAGCTTTTGGGTTCCGGGGGAAGTATGGTTGCAAAGCTGAAACTTAAAGGAATTGACGG
AAGGGCACCACCAGGAGTGGAGCCTGCGGCTTAATTTGACTCAACACGGGAAACCTCACCAGGCCCGGACATT
GGAA-GGATTGACAGAT-CGATAGCTCTTT-CTCGATT-CAGTGGGT-
AGTGGTGCATGGCCGTTCTTAGTTGGTGGATTGATTTGTCTGGTTAATTCCGATACGAAACGAGAACCTTGCC
TATTAACTAGGCGCTC-CCGGTATTCCATATCCTACCGGCGAGAC-
TTATTCTTCTTAAAGGGACAGGCGGCTCTTAGCCGCACGAGATTGAGCAATAACAGGTCTGTGATGCCCTTAG
ATGTTCTGGGCCGCACGCGCGCTACACTGAAGGAATCAGCGTGTTC--
TCCCCTGTCCGAAAGGACCGGGCAACCCGCTGAACCTCCTTCGTGCTAGGAATTGGGGCTTGCAATTGTTCCC
CATGAACGAGGAATTCCCAGTAAGCGCGAGTCATAAGCTCGCGTTGATTACGTCCCTGCCCTTTGTACACACC
GCCCGTCGCTACTACTGATTGGATGATTTAGTGAGGTCTTCAGACC-GGCGGTA--AC-AGGC-ACC--
GGCT-----CGTTCGG---TATCTGT-TCCGCTGGAAA-
GATGACCGAACTTGATCATTTATAAGAAGTAAAAGTCTTAACAAGG-TTTACAGTC---TTGGGTGT---
AAAACCTAAAGGCGCAATGAAAGT-GTC-------------AGGGGGTCCTCTCTC---------
GCGAGGGAGAGG--AACCCCCCGCTGGGAAGATGGGCTACCC---GTACAATTACT--------TGT--
CGGAGCCCCGCACTCCCGGGGCGTCTC--TATCTC-ATTGCG-AG----
TAGAGACGCACCAGGAGCGCACACGCTGGTACCCGAAAGATGGTGAACTATGCCTGGCCAGGACGAAGCCAGG
GGAAACCCTGGTGGAGGTCC--GAAGCG-ATTCTG--AAGTGCAAATCG-
ATCGTCTGAGCTGGGTATAGGGGCGAAAGACCAATCGAACCATCTTGA-AATATTT---

ATTTTAAGTCTAACCTGCTCAATGACT--TGA------
AAGTTTAATAGCCGCAGTATTCTGACTGTGCAAAGGTAGCATAATCATTTGTCTTTTAATTGTAGGCTAGTAT
GAAGGGTTAAATGAGATATTAACTTTCTTTGTTATAAATATTTA---
TGAATTTAATTTTTAAGTTAAAAAGCTTAAATGTTA-TTGTGGGACGAGAAGACCCTATAGAA--
TTTAATTTATTTAATTAATGTTGACAATTATTAATTATTTTTGTAAATGTT----ATTTATCATTATTAATA-
AATAAATTTTGTTGGGGTGACAGGGAAA-
TATATTTAACTTTCTTTATTTAAATCATGTTATTAATCTATATAT-----TAATGATCCTT-----
TATTAAGGATA--ATAAGATTAAATTACCTTAGGGATAAC
Ochetostethus_opacus
ATGCA-GTCTCAGTACGAGCTTACT-
TTAAGTGAAACCGCAAAAGGCTCAGTAAACCGGTCATAGTTTACCGGA-TAAA---CCACTT-
ACGTGGATAACTGTGGTAATTCTAGAGCTAATACATGCAATC-AAGTCCCGACCAGAG---
ATGGAAAGGACGCTTTTATTGGATCAAAACCATTCGCCAGGTTCGCCC-TGGC------
GTCTGCTTGATGACTCT-AGATAACCACAACCAGATCGCACGGTCCTTGTACCGGC-
GACGTATCCGTCAAATGTCTGACTTATCAGCTGTCGATGGTAGACTCTGTGTCTACCATGGCGCTCACGGGTA
ACGGGGAATCAGGGTTCGATTCCGGAGAGGGAGCCTGAGAAACGGCTACCACATCCAAGGAAGGCAGCAGGCA
CGCAAATTACCCACTCCCGGCACGGGGAGGTAGTGACAAAAAATAACGATACGGGACTCATCCGAGGCCCCGT
AATCGGAATGAGAACACCCTAAATCCTTTAACGAGGATCTATTGGAGGGCAAGTCTGGTGCCAGCAGCCGCGG
TAATTCCAGCTCCAATAGCGTATATTAAAGTTGTTGCGGTTAAAAA-
GCTCGTAGTTGGTTCTGCGTGCCACGCTGTCGGTTCACCGCCTGTCGGTGCAACTGGCATGCC-
GTGGCATGTCCTGTCGGTGGCCTACTGGT-TTCGCG-GCGCACCCTATG-------CGTT---CACACGT-G-
TGGGTCGTTGC--ACTGC-GTTGTCC-TAGTAGG-CCGGCT-CAATCCTACCGCGGTGCT-
CTTTACCGAGTGTCGAGGTAGGCCGACACT-
GTTTACTTTGAACAAATTAGAGTGCTTCAAGCAGGCTCTGAAAGC-
AGCCTGAATAGTGGTGCATGGAATAATGGAACAGGACCTTGGTTCTATTTTGTT-GGTTTTCGGA-
ATCCAAGGTAATGATCAATAAGGA-CAGGCGGGGGCATTCGTATTGCGACGTTAGA-----------------
-GGTGAAATTCTTGG-
ATCGGGACTGGTTATAGCAGATGCGAAGCATTTGCCAAGAATGTCTTAATTGATCAAGAACGAAAGTTAGAGG
TTCGAAGGCGATCAGATACCGCCCTAGTTCTAACCATAAACGATGCCAGCCAGCGATCCGCCGATGTTCCTCC
AATGACTCGGCGGGGAGCTT-
CCCCCGGGAAACCAAAGCTTTTGGGTTCCGGGGGAAGTATGGTTGCAAAGCTGAAACTTAAAGGAATTGACGG
AAGGGCACCACCAGGACTGGAGCCTGCGGCTTAATTTGACTCAACACGGGAAACCTCACCAGGCCCGGACATT
GGAA-GGATTGACAGAT-CGATAGCTCTTT-CTCGATT-CAGTGGGT-
AGTGGTGCATGGCCGTTCTTAGTTCGTGGACTGATTTGTCTGGTTAATTCCGATAACGAACGAGACTCTAACC
TATTAACTAGGCGTCC-CCGGTATAC-ATA-CCTACCGGCGAGAC-
TTATTCTTCTTAAAGGGACAGGCGGCTCTTAGCCGCACGAGATTGAGCAATAACAGGTCTGTGATGCCCTTAG
ATGTTCTGGGCCGCACGCGCGCTACACTGAAGGAATCAGCGTGTTC--
TCCCCTGTCCGAAAGGACCGGGCAACCCGCTGAACCTCCTTCGTGCTAGGAATTGGGGCTTGCAATTGTTCCC
CATGAACGAGGAATTCCCAGTAAGCGCGAGTCATAAGCTCGCGTTGATTACGTCCCTGCCCTTTGTACACACC
GCCCGTCGCTACTACCGATTGAATGATTTAGTGAGGTCTTCGGACC-GGCGGTA--AC-AGGC-ACC--
GGCT-----CGTCCGG---TATCGGT-TCCGCTGGAAA-
GATGACCGAACTTGATCATTTAGAGGAAGTAAAAGTCGTAACAAGG-TTTCCAGTTCATTTGGGTGT---
AAAACCTAAAGGCGCAATGAAAGTTGTC-------------AGGGGGACCTCTC-------------
GAGGGAGG-----ACCCCCCGCTGGGAAGATGGGCCGCC----GTACAATTATT--------TGT--
CGGAGCCCCGCATTCCCGGGGCGTCTC--TTTCTC-ATTGCG-AG----
TAGAGACGCACCAGGAGCGCACACGCTGGTACCCGAAAGATGGCGAACTATGCCTGGCCAGGACGAAGCCAGG
GGAAACCCTGGTGGAGGTCC--GAAGCG-ATTCTG--ACGTGCAAATCG-
ATCGTCTGAGCTGGGTATAGGGGCGAAAGACCAATCGAACCATCTTGTTAATTTTT---
ATTTTAAGTCAAGCCTGCTCAATGATTATTGA------
AATTTTAATAGCCGCAGTATTTTGACTGTGCAAAGGTAGCATAATCACTTGTCTTTTAATTGTAGGCTGGTAT
GAACGGTTAGATGAAATATTAGCTTTCTTTATTTTATAATTGAA---
TGAATTTAATATTTGGGTTAAAAAGCTTAAATGTTT-TTGTAAGACGAGAAGACCCTATAGAA--
TTTTATTTTCTTATTT--------TGATTATT--TTATTATTATTGTTGTT----
TATTATAATTAACAAAATGGAAAATTTTGTTGGGGTGACAGGGAAA-
ATTTATTAACTTTCTTTATAAAAATTAT--TATTGATGTATATAT-----GTAAGATCCTT-----
TATTAAGGATA--ATAAGGTTAAATTACCTTAGGGATAAC
Odontoscelis_fuliginosa

ATGCAAGTCTCAGTACGAGCTGACT-
TAAAGTGAAACCGCAAAAGGCTCAGTAAACCGGTCATAGTTTCCCGGA-TATATG-CCACTC-
AGGTGGATAACTGTGGTAATTCTAGAGCTAATACATGCAACC-AAGTCCCGACCAGCG---
ATGGGAAGGACGCTTTTATTGGATCAAAACCATTCGCCCGGCTCGCCC-GGGC------
GTCTGCTTGATGACTCT-AGATAAC-ACCGCA-GATCGCACGGTCACAGCACCGGC-
GACGTATCCGTCAAATGTCTGACTTATCAGCTGTCGATGGTAGACTCTGTGTCTACCATGGCGCTCACGGGTG
ACGGGGAATCAGGGTTCGATTCCGGAGAGGGAGCCTGAGAAACGGCTACCACATCCAAGGAAGGCAGCAGGCA
CGCAAATTACCCACTCCCGGCACGGGGAGGTAGTGACAAAAAATAACGATACGGGACTCATCCGAGGCCCCGT
AATCGGAATGAGAACACCCTAAATCCTTTAACGAGGATCTATTGGAGGGCAAGTCTGGTGCCAGCAGCCGCGG
TAATTCCAGCTCCAATAGCGTATATTAAAGTTGTTGCGGTTAAAAA-
GCTCGTAGTTGGTTCTGCGTGCCACGCTGTCGGTTCACCGCCTGTCGGTGCAACTGGCATGCC-
GTGGCATGTCCTGTCGGTGGCCGACAGGC-TTCGGG-GCGCGCCCTGTG-------CGTTAAC-GCGTGC---
-AGGTGTCCGT--GCAC-CGTTGTCC-TTGTAGG-CCGACT-CAATCCTACCGCGGTGCT-
CTTTACCGAGTGTCGAGGTAGGCCGACACT-
GTTTACTTTGAACAAATTAGAGTGCTTCAAGCAGGCTCTGAAAGC-
AGCCTGAATAGTGGTGCATGGAATAATGGAACAGGACCTTGGTTCTATTTTGTT-GGTTTTCGGA-
ATCCAAGGTAATGATCAATAAGGA-CAGGCGGGGGCATTCGTATTGCGACGTTAGA-----------------
-GGTGAAATTCTTGG-
ATCGTCGCAAGACGCACTAGAGCGAAAGCATTTGCCAAGTATGTCTTAATTGATCAAGAACGAAAGTTAGAGG
TTCGAAGGCGATCAGATACCGCCCTAGTTCTAACCATAAACGATGCCAGCCAGCGATCCGCCGATGTTCCTCC
GATGACTCGGCGGGGAGCTT-
TCCCCGGGAAACCAAAGCTTTTGGGTTCCGGGGGAAGTATGGTTGCAAAGCTGAAACTTAAAGGAATTGACGG
AAGGGCACCACCAGGAGTGGAGCCTGCGGCTTAATTTGACTCAACACGGGAAACCTCACCAGGCCCGGACATT
GGAA-GGATTGACAGAT-CGATAGCTCTTT-CTCGATT-CAGTGGGT-
AGTGGTGCATGGCCGTTCTTAGTTGGTGGACTGATTTGTCTGGTTAATTCCGATAACGAACGAGACTCTGACC
TATTAACTAGGCGTTT-CCGGTATAA-ATA-CCTACCGGCGAGAT-
TTATTCTTCTTAAGGGGACAGGCGGCTCTTAGCCGCACGAGATTGAGCAATAACAGGTCTGTGATGCCCTTAG
ATGTTCTGGGCCGCACGCGCGCTACACTGAAGGAATCAGCGTGTTA--
TCCCCTGTCCGAAAGGTCCGGGCAACCCGCTGAACCTCCTTCGTGCTAGGAATTGGGGCTTGCAATTGTTCCC
CATGAACGAGGAATTCCCAGTAAGCGCGAGTCATAAGCTCGCGTTGATTACGTCCCTGCCCTTTGTACACACC
GCCCGTCGCTACTACCGATTGAATGATTTAGTGAGGTCTTCGGACC-GGCGGCA--AC-AGGC-ACC--GGC-
----TCGTCCGG---TATCGGT-TCCGCTGGAAA-
GATGACCGAACTTGATCATTTAGAGGAAGTAAAAGTCGTAACAAGG-TTTCCAGTCA--TTGGGTGA---
AAAACCTAAAGGCGCAATGAAAGT-GTCAGC------------GCGGGCCTCCTA-------------
CGGGAGC-----GCCCGCCGTAGGGAGGATGGGCCGCC----CGGCGATCACT--------CGT--
CGGCGCCCCGCACTCCCGGGGCGTCTC--GTTCTC-ATTACG-AG----
TGGAGACGCACCAGGAGCGCACACGCTGGTACCCGAAAGATGGTGAACTATGCCTGGCCAGGACGAAGCCAGG
GGAAACCCTGGTGGAGGTCC--GAAGCG-ATTCTG--ACGTGCAAATCG-
ATCGTCTGAGCTGGGTATAGGGGCGAAAGACCAATCGAACCATCGGG--ATT-ATTT--
TTCTTAAGTCGGGCCTGCTCAATGATTT----TT------
ATTAAATAGCCGCAGTATTTTGACTGTGCGAAGGTAGCATAATCATTTGTCTTTTAATTGAAGGCTTGTATGA
AGGGTTTGACGAAATGCAATCTTTCTTTGTATCAAAT-ATT-----
GAATTTAATTTTTAGGTTAAAAAGCTTAAATGTTT-TTATGGGACGAGAAGACCCTATAGAA--TTTTATT--
---------ATTTTATGTATTGGATAATTTTGGTGTATTTA--------
ATTATTTATTATGTAAAATAATTTTGTTGGGGTGACATGGAAA-TTTCTTTAACTTTCTTTACT--TATTTT-
ATATTGATTAATATT-----TTTAAGATCCTAAG--TTT--ATGGA-A--
ATAAGTTTAAATTACCTTAGGGATAAC
Pangaeus_bilineatus
ATGCAAGTCTCAGTACGAGCTGAAT-
TAAAGTGAAACCGCAAAAGGCTCAGTAAACCGGTCATAGTTTACCGGA-TATATG-CCACTC-
ACGTGGATAACTGTGGTAATTCTAGAGCTAATACATGCAACA-AAGTCCCGACCAGTG---
ATGGAAAGGACGCTTTTATTGGATCAAAACCATTCGCCCGGCTCGCCC-GGGC------
GTCTGCTTGATGACTCT-AGATAAC-ACCACA-GATCGCACGGTCACAGTACCGGC-
GACGTATCCGTCAAATGTCTGACTTATCAGCTGTCGATGGTAGACTCTGTGTCTACCATGGCGCTCACGGGTA
ACGGGGAATCAGGGTTCGATTCCGGAGAGGGAGCCTGAGAAACGGCTACCACATCCAAGGAAGGCAGCAGGCA
CGCAAATTACCCACTCCCGGCACGGGGAGGTAGTGACAAAAAATAACGATACGGGACTCATCCGAGGCCCCGT
AATCGGAATGAGAACACCCTAAATCCTTTAACGAGGATCTATTGGAGGGCAAGTCTGGTGCCAGCAGCCGCGG
TAATTCCAGCTCCAATAGCGTATATTAAAGTTGTTGCGGTTAAAAA-

GCTCGTAGTTGGTTCTGCGTGCCACGCTGTCGGTTCACCGCCTGTCGGTGCAACTGGCATGCC-
GTGGCATGTCCTGTCGGTGGCTGAAAGGC-TTCACG-GCGCGCCCTATG-------CGTTCAC-GCGTGT---
-AGGTCGTCGC--GCCGCGTTTGTCC-TTGTAGG-CCGGCT-CAATCCTACCGCGGTGCT-
CTTTGCCGAGTGTCGAGGTAGGCCGACACT-
GTTTACTTTGAACAAATTAGAGTGCTTCAAGCAGGCTCTGAAAGC-
AGCCTGAATAGTGGTGCATGGAATAATGGAACAGGACCTTGGTTCTATTTTGTT-GGTTTTCGGA-
ATCCAAGGTAATGATCAATAAGGA-CAGGCGGGGGCATTCGTATTGCGACGTTAGA-----------------
-
GGTGAAATTCTTGGGATCGTCGCAAGACGCACTAGAGCGAACACATTTGCCAAGTATGTCTTAATTGATCAAG
AACGAAAGTTAGAGGTTCGAAGGCGATCAGATACCGCCCTAGTTCTAACCATAAACGATGCCAGCCAGCGATC
CGCCGATGTTCCTCCGATGACTCGGCGGGGAGCTT-
TCCCCGGGAAACCAAAGCTTTTGGGTTCCGGGGGAAGTATGGTTGCAAAGCTGAAACTTAAAGGAATTGACGG
AAGGGCACCACCAGGAGTGGAGCCTGCGGCTTAATTTGACTCAACACGGGAAACCTCACCAGGCCCGGACATT
GGAA-GGATTGACAGAT-CGATAGCTCTTT-CTCGATT-CAGTGGGT-
AGTGGTGCATGGCCGTTCTTAGTTGGTGGAGTGATTTGTCTGGTTAATTCCGATAACGAACGAGACTCTGACC
TATTAACTAGGCGCTC-CCGGTATAA-ATA-CCTACCGGCGAGAT-
TTATTCTTCTTAAGGGGACAGGCGGCTCTTAGCCGCACGAGATTGAGCAATAACAGGTCTGTGATGCCCTTAG
ATGTTCTGGGCCGCACGCGCGCTACACTGAAGGAATCAGCGTGTGC--
TCCCCTGTCCGAAAGGACCGGGCAACCCGCTGAACCTCCTTCGTGCTAGGAATTGGGGCTTGCAATTGTTCCC
CATGAACGAGGAATTCCCAGTAAGCGCGAGTCATAAGCTCGCGTTGATTACGTCCCTGCCCTTTGTACACACC
GCCCGTCGCTACTACTGATTGGATGATTTAGTGAGGTCTTCGGACC-GGCGGTA--AC-CGGC-ACC--GGC-
----TCGTTCGG---TATCGGT-TTCGCTGGAAA-
GATGACCGAACTTGATCATTTAGAAGAAGTAAAAGTCGTAACAAGG-TTTCCAGTCA--TTGGGTGA---
AAA-CCTAAAGGCGCAATGAAAGT-GTCTG-------------GGTGGCCTCCTT-------------
TAGGAGA-----GTCACCCGTAGGGAGGATGGGCCACC----GTACAATTATT--------TGT--
CGGAGCCCCGCACTCCCGGGGCGTCTC--GTTCTC-ATTACG-AG----
TGGAGACGCACCAGGAGCGCACACGCTGGTACCCGAAAGATGGTGAACTATGCCTGGCCAGGACGAAGCCAGG
GGAAACCCTGGTGGAGGTCC--GAAGCG-ATTCTG--ACGTGCAAATCG-
ATCGTCTGAGCTGGGTATAGGGGCGAAAGACCAATCGAACCATCTTG-TGTAATT-T---
TATTAGGTCGGGCCTGCTCAATGATTAAAGTTAAGGTAAATTAAATAGCCGCAGTATTTTGACTGTGCGAAGG
TAGCATAATCATTTGTCTTTTAATTGTAGGCTTGTATGAATGGTTTGATGAAATACTATCTTTCTTTACATTA
TAATATA-----TAATTTAATTTTTTAGTTAAAAAGCTAAAATTTTT-
TCATAGGACGAGAAGACCCTATAGAG--TTTTATT--------GTATGATATATTTTATTATTCTTTTGG-
ATATATT----ATTAGTTTAATAATTTATTTTACAATTGTGTTGGGGTGACAGGGAAA-
TTTTATTAACTTTCTTTATATATTTTAT-T-ATTAATTAGTATT-----TAAATGATCCTTT---ATT--
ATGGATA--GTAAGATTAAATTACCTTAGGGATAAC
Pangaeus_rugiceps
TTGCA-GTCTCAGTACGAGCTGACT-
TAAAGTGAAACCGCAAAAGGCTCAGTAAACCGGTCATAGTTTACCGGA-TATATG-CCACTC-
ACGTGGATAACTGTGGTAATTCTAGAGCTAATACATGCAACA-AAGTCCCGACCAGTG---
ATGGAAAGGACGCTTTTATTGGATCAAAACCATTCGCCCGGCTCGCCC-GGGC------
GTCTGCTTGATGACTCT-AGATAAC-ACCACA-GATCGCACGGTCACAGTACCGGC-
GACGTATCCGTCAAATGTCTGACTTATCAGCTGTCGATGGTAGACTCTGTGTCTACCATGGCGCTCACGGGTA
ACGGGGAATCAGGGTTCGATTCCGGAGAGGGAGCCTGAGAAACGGCTACCACATCCAAGGAAGGCAGCAGGCA
CGCAAATTACCCACTCCCGGCACGGGGAGGTAGTGACAAAAAATAACGATACGGGACTCATCCGAGGCCCCGT
AATCGGAATGAGAACACCCTAAATCCTTTAACGAGGATCTATTGGAGGGCAAGTCTGGTGCCAGCAGCCGCGG
TAATTCCAGCTCCAATAGCGTATATTAAAGTTGTTGCGGTTAAAAA-
GCTCGTAGTTGGTTCTGCGTGCCACGCTGTCGGTTCACCGCCTGTCGGTGCAACTGGCATGCC-
GTGGCATGTCCTGTCGGTGGCTGAAAGGC-TTCACG-GCGCGCCCTATG-------CGTTCAC-GCGTGT---
-AGGTCGTCGC--GCCGCGTTTGTCC-TTGTAGG-CCGGCT-CAATCCTACCGCGGTGCT-
CTTTGCCGAGTGTCGAGGTAGGCCGACACT-
GTTTACTTTGAACAAATTAGAGTGCTTCAAGCAGGCTCTGAAAGC-
AGCCTGAATAGTGGTGCATGGAATAATGGAACAGGACCTTGGTTCTATTTTGTT-GGTTTTCGGA-
ATCCAAGGTAATGATCAATAAGGA-CAGGCGGGGGCATTCGTATTGCGACGTTAGA-----------------
-GGTGAAATTCTTGG-
ATCGTCGCAAGACGCACTAGAGCGAAAGCATTTGCCAAGTATGTCTTAATTGATCAAGAACGAAAGTTAGAGG
TTCGAAGGCGATCAGATACCGCCCTAGTTCTAACCATAAACGATGCCAGCCAGCGATCCGCCGATGTTCCTCC
GATGACTCGGCGGGGAGCTT-

TCCCCGGGAAACCAAAGCTTTTGGGTTCCGGGGGAAGTATGGTTGCAAAGCTGAAACTTAAAGGAATTGACGG
AAGGGCACCACCAGGAGTGGAGCCTGCGGCTTAATTTGACTCAACACGGGAAACCTCACCAGGCCCGGACATT
GGAA-GGATTGACAGAT-CGATAGCTCTTT-CTCGATT-CAGTGGGT-
AGTGGTGCATGGCCGTTCTTAGTTGGTGGAGTGATTTGTCTGGTTAATTCCGATAACGAACGAGACTCTGACC
TATTAACTAGGCGCTC-CCGGTATAA-ATA-CCTACCGGCGAGAT-
TTATTCTTCTTAAGGGGACAGGCGGCTCTTAGCCGCACGAGATTGAGCAATAACAGGTCTGTGATGCCCTTAG
ATGTTCTGGGCCGCACGCGCGCTACACTGAAGGAATCAGCGTGTGC--
TCCCCTGTCCGAAAGGACCGGGCAACCCGCTGAACCTCCTTCGTGCTAGGAATTGGGGCTTGCAATTGTTCCC
CATGAACGAGGAATTCCCAGTAAGCGCGAGTCATAAGCTCGCGTTGATTACGTCCCTGCCCTTTGTACACACC
GCCCGTCGCTACTACCGATTGAATGATTTAGTGAGGTCTTCGGACC-GGCGGTA--AC-CGGC-ACC--GGC-
----TCGTTCGG---TATCGGT-TTCGCTGGAAA-
GATGACCGAACTTGATCATTTAGAGGAAGTAAAAGTCGTAACAAGG-TTTCCAGTCA--TTGGGTGA---AA-
-CCTAAAGGCGCAATGAAAGT-GTCTG-------------GGTGGCCTCCTT-------------TAGGAGA-
----GTCACCCGTAGGGAGGATGGGCCACC----GTACAATTATT--------TGT--
CGGAGCCCCGCACTCCCGGGGCGTCTC--GTTCTC-ATTACG-AG----
TGGAGACGCACCAGGAGCGCACACGCTGGTACCCGAAAGATGGTGAACTATGCCTGGCCAGGACGAAGCCAGG
GGAAACCCTGGTGGAGGTCC--GAAGCG-ATTCTG--ACGTGCAAATCG-
ATCGTCTGAGCTGGGTATAGGGGCGAAAGACCAATCGAACCATCTTG-TGGAATT-T---
TATTAGGTCGGGCCTGCTCAATGATTAAATTTAATATAA-
TTAAATAGCCGCAGTATTTTGACTGTGCGAAGGTAGCATAATCATTTGTCTTTTAATTGTAGGCTTGTATGAA
TGGTTTGATGAAATACTATCTTTCTTTACATCATAATTTA-----
TAATTTAATTTTTTAGTTAAAAAGCTAAAATTTTT-TCATAGGACGAGAAGACCCTATAGAG--TTTTATT--
------GTATGATATATTTTATTATTCTTTTGG-ATATGTT----
ATTAGTTTAATAATTTGTTTTACAATTGTGTTGGGGTGACAGGGAAA-
TTTTATTAACTTTCTTTATATATTTTAT-T-ATTGATTAGTATT-----TTAATGATCCTTT---ATT--
ATGGATA--GTAAGATTAAATTACCTTAGGGATAAC
Parachilocoris_minutus
ATGCAAGTCTCAGTACGAGCTCATT-
TAAAGTGAAACCGCAAAAGGCTCAGTAAACCGGTCATAGTTTACCGGA-TATATG-CCACTA-
ACGTGGATAACTGTGGTAATTCTAGAGCTAATACATGCAAAC-AAGTCCCTACCAGCG---
ATGGAAAGGACGCTTTTATTGGATCAAAACCATTCGCCCGGCTCGCCC-TGGC------
GTCTGCTTGATGACTCT-AGATAAC-ACCGCA-GATCGCACGGTCATAGTACCGGC-
GACGTATCCGTCAAATGTCTGACTTATCAGCTGTCGATGGTAGACTCTGTGTCTACCATGGCGCTCACGGGTA
ACGGGGAATCAGGGTTCGATTCCGGAGAGGGAGCCTGAGAAACGGCTACCACATCCAAGGAAGGCAGCAGGCA
CGCAAATTACCCACTCCCGGCACGGGGAGGTAGTGACAAAATATAACGATACGGGACTCATCCGAGGCCCCGT
AATCGGAATGAGAACACCCTAAATCCTTTAACGAGGATCTATTGGAGGGAAAGTCTGGTGCCAGCAGCCGCGG
TAATTCCAGCTCCAATAGCGTATATTAAAGTTGTTGCGGTTAAAAA-
GCTCGTAGTTGGTTCTGCGTGCCACGCTGTCGGTTCACCGCCTGTCGGTGCAACTGGCATGCCCGTGGCATGT
CCTGCCGGTGGCCGACGGGC-TTC-GGGTAGCGACCCGCCCGCG---
TGTCAACAGCACGTCGACGGGTAGTTGCC-GCCC--GTTGTAC-CCGTAGG-CCGGCT-
CAATCCTACCGCGGTGCT-CTTTGCCGAGTGTCGAGGTAGGCCGGCACT-
GTTTACTTTGAACAAATTAGAGTGCTTCAAGCAGGCTATGAAAGC-
AGCCTGAATAGTGGTGCATGGAATAATGGAACAGGACCTTGGTTCTATTT-GTT-GGTTCC-GG--
ATCCAAGGTA-TGATCA-TAA-GA-CAGGCGGGGGCATTCGTATTGCGACGTTAGA-----------------
-GGTGAAATTCTTGG-
ATCGTCGCAAGACGCACTAGAGCGAAAGCATTTGCCAAGAATGTCTTAATTGATCAAGAACGAAAGTTAGAGG
CTCGAAGGCGATCAAATACCACCCTAGTTCTAACCATAAACGATGCCAGCCAGCGATCCGCCGATGTTCCTCC
GATGACTCGGCGGGGAGCTT-
TCCCCGGGAAACCAAAGCTTTTGGGTTCCGGGGGAAGTATGGTTGCAAAGCTGAAACTTAAAGGAATTGACGG
AAGGGCACCACCAGAAGTGGAGCCTGCGGCTTAATTTGACTCAACACGGGCAAGCTCACCAGGCCCGGACATT
GGAA-GGATTGACAGAT-CGATAGCTCTTT-CTCGATT-CAGTGGGT-
AGTGGTGCATGGCCGTTCTTAGTTGGTGGACTGATTTGTCTGGTTAATTCCGATAACGAACGAGACTCTGACC
TATTAACTAGGCGTTT-CCGGTATAA-ATATACTACCGGCGAAAT-
TTATTCTTCTTAAGGGGACAGGCGGCTCTCAGCCGCACGAGATTGAGCAATAACAGGTCAGTGATGCCCTTAG
ATGTTCTGGGCCGCACGCGCGCTACACTGAAGGAATCAGCGTGTGC--
TCCCCTGTCCGAAAGGTCCGGGCAACCCGCTGAACCTCCTTCGTGCTAGGAATTGGGGCTTGCAATTGTTCCC
CATGAACGAGGAATTCCCAGTAAGCGCGAGTCATAAGCTCGCGTTGATTACGTCCCTGCCCTTTGTACACACC
GCCCGTCGCTAATACCGATTGAATGATTTAGTGAGGTCTTCGGACC-GGCGGTT--AC-CGGC-ACC--

GGCGTTCCACGCTCGG---TATCGGT-TCCGCTGGAAA-
GATGACCGAACTTGATTATTTAGAGGAATTAAAAGTCGTAACAAGG-TTTCCAGTC---TTGGGTGT----
TAACCTAAAGGCGAGATGAT-GT-GGA-------------AAGGTGTCCTCCTG-----------
GCTCTCAAGGGAGGAT--GAGCTACTGCT--CTGGTCG-TT---CTATGCG-CGGAT------GAC--
CGTGTCTCCGCACTCCCGGGGCGATTC---TTTTT-CATTGCAAG----
TGGAAGCGCACCAAGAACGATCACGCTGAAACCCGAAAGATGGTGAACTATGCCTGGCCAGGACGAATTCAGG
GGAAACCCTGGTGGAGGTCC--GCAACG-ATTCTG--ACGTGCAAATCT-
ATCGACGGAACTGGGTATAGGGGCGAAAGACCAATCGAACCATCTGATAATATATTTAAATAATAAGTCTGAC
CTGCTCAATGATATA----------
TATTAAATAGCCGCAGTATTTTGACTGTGCAAAGGTAGCATAATCATTTGTCCTTTAATTGTGGCTTGTATG
AATGGTTTGATGAAATATAATCTTTCTTTATTATA-ATAATTAT--
TGAATTTAATTTTTTGGTTAAAAAGCTAAAATGTTT-TTATGGGACGAGAAGACCCTATAGAA--
TTTAATAATTTTTATT---------TATTAAATTTAAATTATGTTAAAA-------TTATAATTTAT-
AGATAAATTTATTATGTTGGGGTGACAGGGAAA-TTAATTAAACTTTCTTTTATTAATAA--
ATTATAAATTAGTATATA--TTTAATGATCTTA-----TATTATAGATT--
AAAAGAATAAATTACCTTAGGGATAAC
Parastrachia_japonensis
ATGCAAGTCTCAGTACGAGCTTACT-
TAAAGTGAAACCGCAAAAGGCTCAGTAAACCGGTCATAGTTTACCGGG-TATATG-CCACTC-
AGGTGGATAACTGTGGTAATTCTAGAGCTAATACATGCAACT-AAGTCCCGACCAGTG---
ATGGAAAGGACGCTTTTATTGGATCAAAGCCATTCGCCCGGTTCGCCC-GGGC------
GTCTGCTTGATGACTCT-AGATAAC-ACCACA-GATCGCACGGTCATTGTACCGGC-
GACGTATCCGTCAAATGTCTGACTTATCAGCTGTCGATGGTAGACTCTGTGTCTACCATGGCGCTCACGGGTA
ACGGGGAATCAGGGTTCGATTCCGGAGAGGGAGCCTGAGAAACGGCTACCACATCCAAGGAAGGCAGCAGGCA
CGCAAATTACCCACTCCCGGCACGGGGAGGTAGTGACAAAAAATAACGATACGGGACTCATCCGAGGCCCCGT
AATCGGAATGAGAACACCCTAAATCCTTTAACGAGGATCTATTGGAGGGCAAGTCTGGTGCCAGCAGCCGCGG
TAATTCCAGCTCCAATAGCGTATATTAAAGTTGTTGCGGTTAAAAA-
GCTCGTAGTTGGTTCTGCGTGCCACGCTGTCGGTTCACCGCCTGTCGGTGCAACTGGCATGCC-
GTGGCATGTCCTGTCGGTGGCCTACAGGC-TTCGCG-GCGCACCCTATG-------CGTT---CATTCGT-G-
TGGGTCGTTGC--GCTGT-GTTGTCC-TCGTAGG-CCGGCT-CAATCCTACCGCGGTGCT-
CTTTACCGAGTGTCGAGGTGGGCCGACAAT-
GTTTACTTTGAACAAATTAGAGTGCTTAAAGCAGGCTCTGAAAGC-
AGCCTGAATAGTAGTGCATGGAATAATGGAACAGGACCTTGGTTCTATTTCGTT-GGTTTTCTGA-
ATCCAAGGTAATGATCAATAAGGA-CAGGCGGGGGCATTCGTATTGCGACGTTAGA-----------------
-GGTGAAATTCTTGG-
ATCGTCGCAAGACGCACTAGAGCGAAAGCAATTGCCAAGTATGTCTTAATTGATCAAGAACGAAAGTTAGAGG
TTCGAAGGCGATCAGATACCGCCCTAGTTCTAACCATAAACGATGTCAGCCAGCGATCCGCCGATGTTCCTCC
GATGACACGGCGGGGAGCTT-
TCCCCGGGAAACCAAAGCTTTTGGGTTCCGGGGGAAGTATGGTTGCAAAGCTGAAACTTAAAGGAATTGACGG
AAGGGCACCACCAGGAGTGGAGCCTGCGGCTTAATTTGACTCAACACGGGAAACCTCACCAGGCCCGGACATT
AGAA-GGATTGACAGAT-CGATAGCTCTTT-CTCGATT-CAGTGGGT-
AGTGGTGCATGGCCGTTCTTAGTTGGTGGACTGATTTGTCTGGTTAATTCCGATAACGAACGAGACTCTAGCC
TATTAACTAGGCGCTC-CCGGTATTCCATATCCTACCGGCGAGAT-TTATTCTTCTT---
GGGACAGGCGGCTCTTAGCCGCACGAGATTGAGCAATAACAGGTCTGTGATGCCCTTAGATGTTCTGGGCCGC
ACGCGCGCTACACTGAAGGAATCAGCGTGTTC--
TCCCCTGTCCGAAAGGACCGGGCAACCCGCTGAACCTCCTTCGTGCTAGGAATTGGGGCTTGCAATTGCTCCC
CATGAACGAGGAATTCCCAGTAAGCGCGAGTCATAAGCTCGCGTTGATTACGTCCCTGCCCTTTGTACACACC
GCCCGTCGCTACTACCGATTGAATGATTTAGTGAGGTCTTCGGACC-GGCGGTA--AC-AGGC-ACC--
GGCT-----CGTCCAG---TATCTGT-TCCGCTGGAAA-
GATGACCGAACTTGATCATTTAGAGGAAGTAAAAGTCGTAACAAGG-TTTCCAGTCA--TTGGGTGT---
AAAACCTAAAGGCGCAATGAAAGT-GTC-------------AGTGGGTCATCCTC-------------
CGGGATG-----CCTACTCGCTGGGAAGATGGGTTGCC----GTACAATTATT--------TGT--
CGGAGCCCCGCACTCCCGGGGCGTCTC--GTTCTC-ATTGCG-AG----
TAGAGACGCACCAGGAGCGCACACGCTGGTACCCGAAAGATGGTGAACTATGCCTGGCCAGGACGAAGCCAGG
GGAAACCCTGGTGGAGGTCC--GAAGCG-ATTCTG--ACGTGCAAATCG-
ATCGTCTGAGCTGGGTATAGGGGCGAAAGACCAATCGAACCATCTGATTAATAATTT--
ATTTTAAGTCTAACCTGCTCGATGATTA--AT------
TTATTAAATGGCCGCAGTATTTTGACTGTGCAAAGGTAGCATAATCATTTGTCTTTTAATTTGAGACTGGAAT

GAACGGTTGGACGAAATATTGACTTTCTTTACTTTATTATTTATA--
TGAATTTAATATTTGAGTTAAAAAGCTTAAATATAT-AAATAAGACGAGAAGACCCTATAGAA--
TTTTATATAAAAATTTA--------TATTA-TGTTTTTTGGGAT-TTGTTC----ATGTA--TTTAAT----
TTTTGTATTTTGTTGGGGTGACAGGGAAA-ATTATTTAACTTTCTTTTTATAAATA---
TTAATAATTATTATAT-----TAACGATCCTT-----TATTATGGATA--
TTAAGATTAAATTACCTTAGGGATAAC
Physopelta_gutta
ATGCAAGTCTCAGTACGAGCTGAAT-
TAAAGTGAAACCGCAAAAGGCTCAGTAAACCGGTCATAGTTTACCGGA-TATATG-CCACTA-
ACGTGGATAACTGTGGTAATTCTAGAGCTAATACATGCAAAC-AAGTCCCGACCAGCG---
ATGGAGAGGACGCTTTTATTGGATCAAAACCATTCGCCCG-TTTATTC-GGGC------
GTCTGCTTGATGACTCT-AGATAAC-ACCACA-GATCGCACGGTCTTAGTACCGAT-
GACGTATCCTTCAAATGTCTGACTTATCAGCTGTCGATGGTAGACTCTGTGTCTACCATGGCTCTCACGGGTG
ACGGGGAATCAGGGTTCGATTCCGGAGAGGGAGCCTGAGAAACGGCTACCACATCCAAGGAAGGCAGCAGGCA
CGCAAATTACCCACTCCCGGCACGGGGAGGTAGTGACAAAAAATAACGATACGGGACTCATCCGAGGCCCCGT
AATCGGAATGAGAACACCCTAAATCCTATAACGAGGATCCATTGGAGGGAAAGTCTGGTGCCAGCAGCCGCGG
TAATTCCAGCTCCAATAGCGTATATTAAAGTTGTTGCGGTTAAAAA-
GCTCGTAGTTGGTTCTGCGTGCCACGCTGTCGGTTCACCGCCTGCCGGTGCAACTGGCATGCC-
GTGGCATGTCCTGTCGGTGGCTGATAGGC-TTCACG-GCGTGCACCATGC------AGTTCATGCTGTGT---
-GGGTTGTTGC--GCCGA-GTTGTCC-TTGTAAG-CCGGCT-CAATCCTACCGCGGTGCT-
CTTTACCGAGTGTCGAGGTAGGCCGACACT-
GTTTACTTTGAACAAATTAGAGTGCTTAAAGCAGGCTCTTAAATC-
AGCCTGAATAGTGGTGCATGGAATAATGGAACAGGACCTTGGTTCTATTTTGTT-GGTTTTAGGA-
ATCCAAGGTAATGATCAATAAGGA-CAGGCGGGGGCATTCGTATTGCGACGTTAGA-----------------
-GGTGAAATTCTTGG-
ATCGTCGCAAGACGCACTAGAGCGAAAGCATTTGCCAAGTATGTCCTAATTGATCAAGAACGAAAGTTAGAGG
TTCGAAGGCGATCAGATACCGCCCTAGTTCTAACCATAAACGATGCCAGCCAGCGATCCGCCGAAGTTCCTCC
GATGACTCGGCGGGGAGCTT-
TCCCCGGGAAACCAAAGCTTTTGGGTTCCGGGGGAAGTATGGTTGCAAAGCTGAAACTTAAAGGAATTGACGG
AAGGGCACCACCAGGAGTGGAGTCTGCGGCTTAATTTGACTCAACACGGGAAACCTCACCAGGCCCGGACATT
TGAA-GGATTGACAGAT-CGATAGCTCTTT-CTCGATT-AAGTGGGT-
AGTGGTGCATGGCCGTTCTTAGTTGGTGGACTGATTTGTCTGGTTAATTCCGATAACGAACGAGACTCTAACC
TATTAGCTAGGCGTTT-CCGGTATACAAAG--CTACCGGCGAGAT-
TTATTCTTCTTAAGGGGACAAACGGCTTTTAGCCGCCCGAGAATGAGCAATAACAGGTCTGTGATGCCCCTAG
ATGCTCTGGGCCGCACGCGCGCTACACTGAAGGAATCAGCGTGTGC--
CCCCCTGGCCGAAAGGCCCGGGTAACCCGCTGAACCTCCTTCGTGCTAGGAATTGGGGCTTGCAATTGTTCCC
CATGAACGAGGAATTCCCAGTAAGCGCGAGTCATAAACTCGCGTTGATTACGTCCCTGCCCTTTGTACACACC
GCCCGTCGCTACTACCGATTGAATGATTTAGTGAGGTCTTCGGACC-GGCGATA--AC-CGGC-ACC--GGT-
----TTCATCGCTGGTATCGAT-TTCGCTGGAAA-
GATGACCGAACTTGATCATTTAGAGGAAGTAAAAGTCGTAACAAGG-TTTCCAGTCA--TTGGGTGT---
AAAACCTAAAGGCGCAATGAAAGT-GTCAG---------------AGTTAGCCCAGT----------------
------GCTTTCTCCGTGGGAAGATGGGT--TCC---GTGCCGTAAGG------------
CCGGTGCCCCGCACTCCCGGGGCGTTTC--ATTCTC-ACTTGT-AG----
ACGAGACGCACCAGGAGCGCACACGCTGGTATCCGAAAGATGGTGAACTATGCCTGGCCAGGACGAAGCCAGG
GGAAACCCTGGTGGAGGTCC--GAAGCG-ATTCTG--ACGTGCAAATCG-
ATCGTCTGAGCTGGGTATAGGGGCGAAAGACCAATCGAACCATCTTG----TT--
AATAATTTTAAGTCTAGCCTGCTCAATGAATA--------
TTTATTTTAATAGCCATAGTATTTTAACTATGCAAAGGTAGCATAATCATTTGTCTTTTAATTGAAGGCTTGT
ATGAAGGGTTTGACGAGGGGTAGACTTTCTTAATTTAAATTTAAT----
TAAACTTAACTTCTAGGTAAAAAGGCTTAGATATAG-TTTTGGGACGAGAAGACCCTATAGAA--TTTTA-T-
-----------------TATTATATTTTATTTTTGTTATTATA----
TTAATAAGAGTTATTTGTAATAATTT-TGTTGGGGTGACAGTGGAA-
TTTTTTTAACTTCTATTATTTATATT---TCATTAATTAGTGTTT-----TATTGATCCTTTA----T-
TATGGATT--ATAAGATTAAATTACCTTAGGGATAAC
Picromerus_lewisi
ATGCAAGTCTCAGTACGAGCTGACT-
TAAAGTGAAACCGCAAAAGGCTCAGTAAACCGGTCATAGTTTACCGAA-TATATG-CCACTC-
ACGTGGATAACTGTGGTAATTCTAGAGCTAATACATGCAACC-AAGTCCCGACCAGCG---

ATGGAAAGGACGCTTTTATTGGATCAAAACCATTCGCCCGGTTCGCCC-GGGC------
GTCTGCTTGATGACTCT-AGATAAC-ACCACA-GATCGCACGGTCACAGTACCGGC-
GACGTATCCGTCAAATGTCTGACTTATCAGCTGTCGATGGTAGACTCTGTGTCTACCATGGCGCTCACGGGTA
ACGGGGAATCAGGGTTCGATTCCGGAGAGGGAGCCTGAGAAACGGCTACCACATCCAAGGAAGGCAGCAGGCA
CGCAAATTACCCACTCCCGGCACGGGGAGGTAGTGACAAAAAATAACGATACGGACTCATCCGAGGCCCCGT
AATCGGAATGAGAACACCCTAAATCCTTTAACGAGGATCTATTGGAGGGCAAGTCTGGTGCCAGCAGCCGCGG
TAATTCCAGCTCCAATAGCGTATATTAAAGTTGTTGCGGTTAAAAA-
GCTCGTAGTTGGTTCTGCGTGCCACGCTGTCGGTTCACCGCCTGTCGGTGCAACTGGCATGCC-
GTGGCATGTCCTGTCGGTGGCTGACGGGC-TTCGCGGCG-CGCCCCACT-------CGCTCAGG-CGTGT---
-GGGT-GACGC--GCCGT-GTTGTCC-TCG-TTGGCCGGCT-CAATCCTACCGCGGTGCT-
CTTAAACGAGTGTCGAGGTAGGCCGACACT-
GTTTACTTTGAACAAATTAGAGTGCTTCAAGCAGGCTCTGAAAGC-
AGCCTGAATAGTGGTGCATGGAATAATGGAACAGGACCTTGGTTCTATTTTGTT-GGTTTTCTGA-
ATCCAAGGTAATGATCAATAAGGA-CAGGCGGGGGCATTCGTATTGCGACGTTAGA-----------------
-GGTGAAATTCTTGG-
ATCGTCGCAAGACGCACTAGAGCGAAAGCATTTGCCAAGTATGTCTTAATTGATCAAGAACGAAAGTTAGAGG
TTCGAAGGCGATCAGATACCGCCCTAGTTCTAACCATAAACGATGCCAGCCAGCGATCCGCCGATGTTCCTCC
GATGACTCGGCGGGGAGCTT-
TCCCCGGGAAACCAAAGCTTTTGGGTTCCGGGGGAAGTATGGTTGCAAAGCTGAAACTTAAAGGAATTGACGG
AAGGGCACCACCAGGAGTGGAGCCTGCGGCTTAATTTGACTCAACACGGGAAACCTCACCAGGCCCGGACATT
GGAA-GGATTGACAGAT-CGATAGCTCTTT-CTCGATT-CAGTGGGT-
AGTGGTGCATGGCCGTTCTTAGTTGGTGGACTGATTTGTCTGGTTAATTCCGATAACGAACGAGACTCTGACC
TATTAACTAGGCGCTC-CCGGTATAA-ATA-CCTACCGGCGACAA-
TTATTCTTCTTAAAGGGACAGGCGGCTCTCAGCCGCACGAGATTGAGCAATAACAGGTCAGTGATGCCCTTAG
ATGTTCTGGGCCGCACGCGCGCTACACTGAAGGAATCAGCGTGTTC--
TCCCCTGTCCGAAAGGACCGGGCAACCCGCTGAACCTCCTTCGTGCTAGGAATTGGGGCTTGCAATTGTTCCC
CATGAACGAGGAATTCCCAGTAAGCGCGAGTCATAAGCTCGCGTTGATTACGTCCCTGCCCTTTGTACACACC
GCCCGTCGCTACTACCGATTGAATGATTTAGTGAGGTCTTCGGACC-GGCGGTA--AC-CGGC-ACC--GG--
---CTCGTCCGG---TATCGGT-TTCGCTGGAAA-
GATGACCGAACTTGATCATTTAGAGGAAGTAAAAGTCGTAACAAGG-TTTCCAGTCA--TTGGGT--G-
TAAAACCTAAAGGCGCAATGAAAGT-GTCA-------------GGGGTGCCTCCTT------------TAGG-
-AG----CGCATCCCGTTGGGAGGATGGGC-CGCC---GTACGATCA-C-T------CGT--
CGGAGCCCCGCACTCCCGGGGCGTCTC--GTTCTCA-TCACG-AG----
TGGAGACGCACCAGGAGCGCACACGCTGGTACCCGAAAGATGGTGAACTATGCCTGGCCAGGACGAAGCCAGG
GGAAACCCTGGTGGAGGTCC--GAAGCG-ATTCTG--ACGTGCAAATCG-
ATCGTCTGAGCTGGGTATAGGGGCGAAAGACCAATCGAACCATC-G---GTGTATTT--
GTATTAAGTCTAGCCTGCTCAATGATTTTTATTATATAGGATTAAATGGCCGCAGTATTTTGACTGTGCGAAG
GTAGCATAATCATTTGTCTTTTAATTGAAGGCTTGCATGAATGGTTGGATGAGGGAATAGCTTTCTTTA---
TATTAAAA-GT--TGAATTTAATGTTTCAGTTAAAAAGCTGAGATTTTT-
AAGTGGGACGAGAAGACCCTATAGAA--TTTT--TACTAACAGG----------
GTTTAAGTATTATTTTGATAAGATC----AAATACTTATATACTAGTT--
AAGTTTTGTTGGGGTGACAATGAGA-TTTATTTAACTCTCA-AATATAATTTAT-TTATTAATTTATATAT--
---TTAGGATCCTA-----TTATAAGGATA--ATTAGATTAAATTACCTTAGGGATAAC
Poecilocoris_druraei
ATGCAAGTCTCAGTACGAGCTGACT-
TAAAGTGAAACCGCAAAAGGCTCAGTAAACCGGTCATAGTTTACCGGA-TATATG-CCACTC-
ACGTGGATAACTGTGGTAATTCTAGAGCTAATACATGCAACC-AAGTCCCGACCAGCG---
ATGGGAAGGACGCTTTTATTGGATCAAAACCATTCGCCCGGCCCGCCC-GGGC------
GTCTGCTTGATGACTCT-AGATAAC-ACCACA-GATCGCACGGTCACAGTACCGGC-
GACGTATCCGTCAAATGTCTGACTTATCAGCTGTCGATGGTAGACTCTGTGTCTACCATGGCGCTCACGGGTG
ACGGGGAATCAGGGTTCGATTCCGGAGAGGGAGCCTGAGAAACGGCTACCACATCCAAGGAAGGCAGCAGGCA
CGCAAATTACCCACTCCCGGCACGGGGAGGTAGTGACAAAAAATAACGATACGGACTCATCCGAGGCCCCGT
AATCGGAATGAGAACACCCTAAATCCTTTAACGAGGATCTATTGGAGGGCAAGTCTGGTGCCAGCAGCCGCGG
TAATTCCAGCTCCAATAGCGTATATTAAAGTTGTTGCGGTTAAAAA-
GCTCGTAGTTGGTTCTGCGTGCCACGCTGTCGGTTCACCGCCTGTCGGTGCAACTGGCATGCC-
GTGGCATGTCCTGTCGGTGGCCGACAGGC-TTCGCG-GCGCGCCCTGTG-------CGTTCAC-GCGTGC---
-AGGTCGCCGC--GCCT-CGTCGTCC-TTGTAGG-CCGGCT-CAATCCTACCGCGGTGCT-
CTTTGCCGAGTGTCGAGGTAGGCCGACACT-

GTTTACTTTGAACAAATTAGAGTGCTTCAAGCAGGCTCTGAAAGC-
AGCCTGAATAGTGGTGCATGGAATAATGGAACAGGACCTTGGTTCTATTTTGTT-GGTTTTCGGA-
ATCCAAGGTAATGATCAATAAGGA-CAGGCGGGGGCATTCGTATTGCGACGTTAGA-----------------
-GGTGAAATTCTTGG-
ATCGTCGCAAGACGCACTAGAGCGAAAGCATTTGCCAAGTATGTCTTAATTGATCAAGAACGAAAGTTAGAGG
TTCGAAGGCGATCAGATACCGCCCTAGTTCTAACCATAAACGATGCCAGCCAGCGATCCGCCGATGTTCCTCC
GATGACTCGGCGGGGAGCTT-
TCCCCGGGAAACCAAAGCTTTTGGGTTCCGGGGGAAGTATGGTTGCAAAGCTGAAACTTAAAGGAATTGACGG
AAGGGCACCACCAGGAGTGGAGCCTGCGGCTTAATTTGACTCAACACGGGAAACCTCACCAGGCCCGGACATT
GGAA-GGATTGACAGAT-CGATAGCTCTTT-CTCGATT-CAGTGGGT-
AGTGGTGCATGGCCGTTCTTAGTTGGTGGACTGATTTGTCTGGTTAATTCCGATAACGAACGAGACTCTGACC
TATTAACTAGGCGATT-CCGGTATAA-ATA-CCTACCGGCGAGAT-
TTATTCTTCTTAAGGGGACAGGCGGCTCTTAGCCGCACGAGATTGAGCAATAACAGGTCTGTGATGCCCTTAG
ATGTTCTGGGCCGCACGCGCGCTACACTGAAGGAATCAGCGTGTGC--
TCCCCTGTCCGAAAGGTCCGGGCAACCCGCTGAACCTCCTTCGTGCTAGGAATTGGGGCTTGCAATTGTTCCC
CATGAACGAGGAATTCCCAGTAAGCGCGAGTCATAAGCTCGCGTTGATTACGTCCCTGCCCTTTGTACACACC
GCCCGTCGCTACTACCGATTGAATGATTTAGTGAGGTCTTCGGACC-GGCGGCA--AC-CGGC-ACC--GGC-
----TCGTTCGG---TATCGGT-TCCGCTGGAAA-
GATGACCGAACTTGATCATTTAGAGGAAGTAAAAGTCGTAACAAGG-TTTCCAGTCA--TTGGGTGA---
AAAACCTAAAGGCGCAATGAAAGT-GTCAGC------------GGGGGCCTCCTA-------------
CGGGAGA-----GCCCTCCGTTGGGAGGATGGGCCGCC----CGGCGTTCACT--------CGC--
CGGCGCCCCGCACTCCCGGGGCGTCTC--GTTCTC-ATCGCG-AG----
TGGAGACGCACCAGGAGCGCACACGCTGGTACCCGAAAGATGGTGAACTATGCCTGGCCAGGACGAAGCCAGG
GGAAACCCTGGTGGAGGTCC--GAAGCG-ATTCTG--ACGTGCAAATCG-
ATCGTCTGAGCTGGGTATAGGGGCGAAAGACCAATCGAACCATCTG-----TAATTG--
ATTTCAAGTCGGACCTGCTCAATGATTACCTTTATAGGGTATTAAATAGCCGCAGTATTTTGACTGTGCGAAG
GTAGCATAATCATTTGTCTTTTAATTGTAGGCTTGTATGAATGGTTTAACAAAATGTAATCTTTCTTTATATT
AATATTAT-----GAATTTAATTTTTGAGTTAAAAAGCTTAAATTTAT-
TTATAGGACGAGAAGACCCTGTAGAA--TTTTACT-----------
TAATATGTATTATTTTAATTTTGGTTATTTAT--------
TTTATTTTATTTTATATTAAGTTTTATTGGGGCGATAGGGAAA-TTTTATTAACTTTCTTTTTT--TATTTA-
TTATTAATTTGTATA-----TTATTGATCCTTA----TT--TTGGATA--
AAAAGATTAAATTACCTCAGGGATAAC
Pseudoscoparipes_fraterculus
ATGCAAGTCTCAGTACGAGCTGACT-
TAAAGTGAAACCGCAAAAGGCTCAGTAAACCGGTCATAGTTTACCGGA-TATATG-CCACTC-
ACGTGGATAACTGTGGTAATTCTAGAGCTAATACATGCAACC-AAGTCCCGACCAGCG---
ATGGAAAGGACGCTTTTATTGGATCAAAACCATTCGCCCGGCTCGCCC-GGGC------
GTCTGCTTGATGACTCT-AGATAAC-ACCACA-GATCGCACGGTCACAGTACCGGC-
GACGTATCCGTCAAATGTCTGACTTATCAGCTGTCGATGGTAGACTCTGTGTCTACCATGGCGCTCACGGGTA
ACGGGGAATCAGGGTTCGATTCCGGAGAGGGAGCCTGAGAAACGGCTACCACATCCAAGGAAGGCAGCAGGCA
CGCAAATTACCCACTCCCGGCACGGGGAGGTAGTGACAAAAAATAACGATACGGGACTCATCCGAGGCCCCGT
AATCGGAATGAGAACACCCTAAATCCTTTAACGAGGATCTATTGGAGGGCAAGTCTGGTGCCAGCAGCCGCGG
TAATTCCAGCTCCAATAGCGTATATTAAAGTTGTTGCGGTTAAAAA-
GCTCGTAGTTGGTTCTGCGTGCCACGCTGTCGGTTCACCGCCTGTCGGTGCAACTGGCATGCC-
GTGGCATGTCCTGTCGGTGGCTGACAGGC-TTCGCG-GCGCGCCCTATG-------CGTTCAC-ACGTGT---
-AGGTCGTTGC--GCCG-CGTTGTCC-TTGTAGG-CCGGCT-CAATCCTACCGCGGTGCT-
CTTTGCCGAGTGTCGAGGTAGGCCGACACT-
GTTTACTTTGAACAAATTAGAGTGCTTCAAGCAGGCTCTGAAAGC-
AGCCTGAATAGTGGTGCATGGAATAATGGAACAGGACCTTGGTTCTATTTTGTT-GGTTTCCGGA-
ATCCAAGGTAATGATCAATAAGGA-CAGGCGGGGGCATTCGTATTGCGACGTTAGA-----------------
-GGTGAAATCTTTTG-
ATCTTCGCAAGACGCACTAGAGCGAAAGCATTTGCCAAGTATGTCTTAATTGATCAAGAACGAAAGTTAGAGG
TTCGAAGGCGATCAGATACCGCCCTAGTTCTAACCATAAACGATGCCAGCCAGCGATCCGCCGATGTTCCTCC
GATGACTCGGCGGGGAGCTT-
TCCCCGGGAAACCAAAGCTTTTGGGTTCCGGGGGAAGTATGGTTGCAAAGCTGAAACTTAAAGGAATTGACGG
AAGGGCACCACCAGGAGTGGAGCCTGCGGCTTAATTTGACTCAACACGGGAAACCTCACCAGGCCCGGACATT
GGAA-GGATTGACAGAT-CGATAGCTCTTT-CTCGATT-CAGTGGGT-

AGTGGTGCATGGCCGTTCTTAGTTGGTGGACTGATTTGTCTGGTTAATTCCGATAACGAACGAGACTCTGACC
TATTAACTAGGCGCTC-CCGGTATAA-ATA-CCTACCGGCGAGAT-
TTATTCTTCTTAAGGGGACAGGCGGCTCATAGCCGCACGAGATTGAGCAATAACAGGTCTGTGATGCCCTTAG
ATGTTCTGGGCCGCACGCGCGCTACACTGAAGGAATCAGCGTGTGC--
TCCCCTGTCCGAAAGGTCCGGGCAACCCGCTGAACCTCCTTCGTGCTAGGAATTGGGGCTTGCAATTGTTCCC
CATGAACGAGGAATTCCCAGTAAGCGCGAGTCATAAGCTCGCGTTGATTACGTCCCTGCCCTTTGTACACACC
GCCCGTCGCTACTACCGATTGAATGATTTAGTGAGGTCTTCGGACC-GGCGGTA--AC-CGGC-ACC--GGC-
----TCGTTCGG---TATCGGT-TTCGCTGGAAA-
GATGACCGAACTTGATCATTTAGAGGAAGTAAAAGTCGTAACAAGG-TTTCCAGTCA--TTGGGTGT---
AAAACCTAAAGGCGCAATGAAAGT-GTCAG-------------GGGGGCCTCCTT-------------
TAGGAGC-----GCTCTCCGCAGGGAGGATGGGCCACC----GTACAATTACT--------TGT--
CGGAGCCCCGCACTCCCGGGGCGTCTC--GTTCTC-ATCACG-AG----
TGGAGACGCACCAGGAGCGCACACGCTGGTACCCGAAAGATGGTGAACTATGCCTGGCCAGGACGAAGCCAGG
GGAAACCCTGGTGGAGGTCC--GAAGCG-ATTCTG--ACGTGCAAATCG-
ATCGTCTGAGCTGGGTATAGGGGCGAAAGACCAATCGAACCATCTGT--GTT-GTAT--
TTATTAGGTCAGGCCTGCTCAATGATT-GT----
TTATGAATTTAATAGCCGCAGTATTTTGACTGTGCAAAGGTAGCATAATCATTTGTCTCTTAATTGTGGGCTT
GTATGAATGGTTTGACGAGACATTGGCTTTCTTTACATTATAGT--A---
TTGAATTTAATTTTTGAGTTAAAAAGCTTAAATGGTT-TTGTAGGACGAGAAGACCCTATAGAA--
ATTTATTAATA--ATGTTTATCTTTTAT-----GCTTTTAGGTTATTATA----
GAGTTTATGGGGTATTATTATTA-ATTTTGTTGGGGTGACAGGGGGA-TTATTTTAACTCCCTTTAAA-
TGTTTAT-TTATTGATTTATATAT--GTGT---GATCCTGTA---TT--ATGGATA--
GTAAGATTAATTTACCTTAGGGATAAC
Pseudoscoparipes_kinabalensis
ATGCAAGTCTCAGTATGAGCTGACT-
TAAAGTGAAACCGCAAAAGGCTCAGTAAACCGGTCATAGTTTACCGGA-TATATG-GCACTC-
ACGTGGATAAGTGTGGTAATTCTAGAGCTAATACATGCAACC-AAGTCCCGACCAGCG---
ATGGAAAGGACGCTTTTATTGGATCAAAACCATTCGCCCGGCTCGCCC-GGGC------
GTCTGCTTGATGACTTT-AGATAAC-ACCACA-GATCGCACGGTCACAGTACCGGC-
GACGTATCCGTCAAATGTCTGACTTATCAGCTGTCGATGGTAGACTCTGTGTCTACCATGGCGCTCACGGGTA
ACGGGGAATCAGGGTTCGATTCCGGAGAGGGAGCCTGAGAAACGGCTACCACATCCAAGGAAGGCAGCAGGCA
CGCAAATTACCCACTCCCGGCACGGGGAGGTAGTGACAAAAAATAACGATACGGGACTCATCCGAGGCCCCGT
AATCGGAATGAGAACACCCTAAATCCTTTAACGAGGATCTATTGGAGGGCAAGTCTGGTGCCAGCAGCCGCGG
TAATTCCAGCTCCAATAGCGTATATTAAAGTTGTTGCGGTTAAAAA-
GCTCGTAGTTGGTTCTGCGTGCCACGCTGTCGGTTCACCGCCTGTCGGTGCAACTGGCATGCC-
GTGGCATGTCCTGTCGGTGGCTGACAGGC-TTCGCG-GCGCGCCCTATG-------CGTTCAC-ACGTGT---
-AGGTCGTTGC--GCCG-CGTTGTCC-TTGTAGG-CCGGCT-CAATCCTACCGCGGTGCT-
CTTTGCCGAGTGTCGAGGTAGGCCGACACT-
GTTTACTTTGAACAAATTAGAGTGCTTCAAGCAGGCTCTGAAAGC-
AGCCTGAATAGTGGTGCATGGAATAATGGAACAGGACCTTGGTTCTATTTTGTT-GGTTTCCGGA-
ATCCAAGGTAATGATCAATAAGGA-CAGGCGGGGGCATTCGTATTGCGACGTTAGA-----------------
-GGTGAAATCTTTGG-
ATCGTCGCAAGACGCACTAGAGCGAAAGCATTTGCCAAGAATGTGTGAATTGATCAAGAACGAAAGTTAGAGG
TTCGAAGGCGATCAGATACCGCCCTAGTTCTAACCATAAACGATGCCAGCCAGCGATCCGCCGATGTTCCTCC
GATGACTCGGCGGGGAGCTT-
TCCCCGGGAAACCAAAGCTTTTGGGTTCCGGGGGAAGTATGGTTGCAAAGCTGAAACTTAAAGGAATTGACGG
AAGGGCACCACCAGGAGTGGAGCCTGCGGCTTAATTTGACTCAACACGGGAAACCTCACCAGGCCCGGACATT
GGAA-GGATTGACAGAT-CGATAGCTCTTT-CTCGATT-CAGTGGGT-
AGTGGTGCATGGCCGTTTTTAGTTGGTGGACTGATTTGTCTGGTTAATTCCGATAACGAACGAGACTTTGACC
TATTAACTAGGCGCTC-CCGGTATAA-ATA-CCTACCGGCGAGAT-
TTATTCTTCTTAAGGGGACAGGCGGCTCATAGCCGCACGAGATTGAGCAATAACAGGTCTGTGATGCCCTTAG
ATGTTTTGGGCCGCACGCGCGCTACACTGAAGGAATCAGCGTGTGC--
TCCCCTGTCCGAAAGGTCCGGGCAACCCGCTGAACCTCCTTCGTGCTAGGAATTGGGGCTTGCAATTGTTCCC
CATGAACGAGGAATTCCCAGTAAGCGCGAGTCATAAGCTCGCGTTGATTACGTCCCTGCCCTTTGTACACACC
GCCCGTCGCTACTACCGATTGAATGATTTAGTGAGGTCTTCGGACC-GGCGGTA--AC-CGGC-ACC--GGC-
----TCGTTCGG---TATCGGT-TTCGCTGGAAA-
GATGACCGAACTTGATCATTTAAAGGAAGTAAAAGTCGTAACAAGG-TTTCCAGTCA--TTGGGTGT---
AAA-CCTAAAGGCGCAATGAAAGT-GTCAG-------------GGGGGCCTCCTT-------------

TAGGAGC-----GCTCTCCGCAGGGAGGATGGGCCACC----GTACAATTACT--------TGT--
CGGAGCCCCGCACTCCCGGGGCGTCTC--GTTCTC-ATCACG-AG----
TGGAGACGCACCAGGAGCGCACACGCTGGTACCCGAAAGATGGTGAACTATGCCTGGCCAGGACGAAGCCAGG
GGAAACCCTGGTGGAGGTCC--GAAGCG-ATTCTG--ACGTGCAAATCG-
ATCGTCTGAGCTGGGTATAGGGGCGAAAGACCAATCGAACCATCTGT-TGGGTGTAT--
TTATTAGGTCAGGCCTGCTCA-TGATT--------
AATTGTTTAAATAGCCGCAGTATTTTGACTGTGCAAAGGTAGCATAATCATTTGTCTTTTAATTGTAGGCTTG
TATGAATGGTTTGACGAGACATTGACTTTCTTTGGGTTATTGATAA---
TTGAATTTAATTTTTAGTCAAAAAGCTAAGATGCTT-TTATAGGACGAGAAGACCCTGTAGAA--
ATTTATTAATT--
ATATTTTCTTTTTCTTTTGGGTTATGGAGTTTTTATATGAAGTTTTAATTTGAGATTATAATTA-
ATTGTGTTGGGGTGACAGGGAAA-TTTTATTAACTTTCTTTAAT-TATT-AT-TTATTAATTTGTATGT--
TAGG---GATCCTATT---TT--AAGGATA--ATAAGATTAATTTACCTTAGGGATAAC
Pseudoscoparipes_vollenhoveni
ATGCAAGTCTCAGTACGAGCTGACT-
TAAAGTGAAACCGCAAAAGGCTCAGTAAACCGGTCATAGTTTACCGGA-TATATG-CCACTC-
ACGTGGATAACTGTGGTAATTCTAGAGCTAATACATGCAACC-AAGTCCCGACCAGCG---
ATGGAAAGGACGCTTTTATTGGATCAAAACCATTCGCCCGGCTCGCCC-GGGC------
GTCTGCTTGATGACTCT-AGATAAC-ACCACA-GATCGCACGGTCACAGTACCGGC-
GACGTATCCGTCAAATGTCTGACTTATCAGCTGTCGATGGTAGACTCTGTGTCTACCATGGCGCTCACGGGTA
ACGGGGAATCAGGGTTCGATTCCGGAGAGGGAGCCTGAGAAACGGCTACCACATCCAAGGAAGGCAGCAGGCA
CGCAAATTACCCACTCCCGGCACGGGGAGGTAGTGACAAAAAATAACGATACGGGACTCATCCGAGGCCCCGT
AATCGGAATGAGAACACCCTAAATCCTTTAACGAGGATCTATTGGAGGGCAAGTCTGGTGCCAGCAGCCGCGG
TAATTCCAGCTCCAATAGCGTATATTAAAGTTGTTGCGGTTAAAAA-
GCTCGTAGTTGGTTCTGCGTGCCACGCTGTCGGTTCACCGCCTGTCGGTGCAACTGGCATGCC-
GTGGCATGTCCTGTCGGTGGCTGACAGGC-TTCGCG-GCGCGCCCTATG-------CGTTTAC-ACGTGT---
-AGGTCGTTGC--GCCG-CGTTGTCC-TTGTAGG-CCGGCT-CAATCCTACCGCGGTGCT-
CTTTGCCGAGTGTCGAGGTAGGCCGACACT-
GTTTACTTTGAACAAATTAGAGTGCTTCAAGCAGGCTCTGAAAGC-
AGCCTGAATAGTGGTGCATGGAATAATGGAACAGGACCTTGGTTCTATTTTGTT-GGTTTCCGGA-
ATCCAAGGTAATGATCAATAAGGA-CAGGCGGGGGCATTCGTATTGCGACGTTAGA-----------------
-GGTGAAATTCTTGG-
ATCGTCGCAAGACGCACTAGAGCGAAAGCATTTGCCAAGTATGTCTTAATTGATCAAGAACGAAAGTTAGAGG
TTCGAAGGCGATCAGATACCGCCCTAGTTCTAACCATAAACGATGCGAGCCAGCGATCCGCCGATGTTCCTCC
GATGACTCGGCGGGGAGCTT-
TCCCCGGGAAACCAAAGCTTTTGGGTTCCGGGGGAAGTATGGTTGCAAAGCTGAAACTTAAAGGAATTGACGG
AAGGGCACCACCAGGAGTGGAGCCTGCGGCTTAATTTGACTCAACACGGGAAACCTCACCAGGCCCGGACATT
GGAA-GGATTGACAGAT-CGATAGCTCTTT-CTCGATT-CAGTGGGT-
AGTGGTGCATGGCCGTTCTTAGTTGGTGGACTGATTTGTCTGGTTAATTCCGATAACGAACGAGACTCTGACC
TATTAACTAGGCGCTC-CCGGTATAA-ATA-CCTACCGGCGAGAT-
TTATTCTTCTTAAGGGGACAGGCGGCTCATAGCCGCACGAGATTGAGCAATAACAGGTCTGTGATGCCCTTAG
ATGTTCTGGGCCGCACGCGCGCTACACTGAAGGAATCAGCGTGTGC--
TCCCCTGTCCGAAAGGTCCGGGCAACCCGCTGAACCTCCTTCGTGCTAGGAATTGGGGCTTGCAATTGTTCCC
CATGAACGAGGAATTCCCAGTAAGCGCGAGTCATAAGCTCGCGTTGATTACGTCCCTGCCCTTTGTACACACC
GCCCGTCGCTACTACCGATTGAATGATTTAGTGAGGTCTTCGGACC-GGCGGTA--AC-CGGC-ACC--GGC-
----TCGTTCGG---TATCGGT-TTCGCTGGAAA-
GATGACCGAACTTGATCATTTAGAGGAAGTAAAAGTCGTAACAAGG-TTTCCAGTCA--TTGGGTGT---
AAAACCTAAAGGCGCAATGAAAGT-GTCAG-------------GGGGGCCTCCTT-------------
TAGGAGC-----GCTCTCCGGAGGGAGGATGGGCCACC----GTACAATTACT--------TGT--
CGGAGCCCCGCACTCCCGGGGCGTCTC--GTTCTC-ATCACG-AG----
TGGAGACGCACCAGGAGCGCACACGCTGGTACCCGAAAGATGGTGAACTATGCCTGGCCAGGACGAAGCCAGG
GGAAACCCTGGTGGAGGTCC--GAAGCG-ATTCTG--ACGTGCAAATCG-
ATCGTCTGAGCTGGGTATAGGGGCGAAAGACCAATCGAACCATCTGT--GTTTATTT--
ATATTAGGTTAGGCCTGCTCAATGATTTAT----
TTATTGATTTAATAGCCGCAGTATTTTGACTGTGCAAAGGTAGCATAATCATTTGTCTTTTAATTGTAGGCTT
GTATGAATGGTTTGACGAGACATTGGCTTTCTTTGAACTATTTT--G---
TTGAATTTAATTTTTGAGTTAAAAAGCTTAAATGCTT-TTGTAGGACGAGAAGACCCTATAGAA--
ATTTATTA-----GTGTATATATCTTTT-----GCTTTTAGATTAATAAA----

GTTTTATATTGATATTACTATTA-ATTGTGTTGGGGTGACAGGGTGA-TTGTTATAACTCACTATGAT-
TGTTTAT-TTATTGATTTATATAT--ATTT---GATCCTATT---TT--AAGGACA--
GTAAGATTAATTTACCTTAGGGATAAC
Pyrrhocoris_apterus
ATGCAAGTCTCAGTACGAGCTTTCT-
TAAAGTGAAACCGCAAAAGGCTCAGTAAACCGGTCATAGTTTACAGGA-TAAATG-CCACTT-
ACGTGGATAACTGTGGTAATTCTAGAGCTAATACATGCAAACCAAGTCCCGACCAGCG---
ATGGAGAGGACGCTTTTATTGGATCAAAACCATTCGCCCGGCTCGCCC-GGGC------
GTCTGCTTGATGACTCT-AGATAAC-AACACA-GATCGTACGGTCATAGTACCGGC-
GACGTATCCTTCAAATGTCTGACTTATCAGCTGTCGATGGTAGATTCTGTGTCTACCATGGCGCTCACGGTG
ACGGGGAATCAGGGTTCGATTCCGGAGAGGGAGCCTGAGAAACGGCTACCACATCCAAGGAAGGCAGCAGGCA
CGCAAATTACCCACTCCCGGCACGGGGAGGTAGTGACAAAAAATAACGATACGGGACTCATCCGAGGCCCCGT
AATCGGAATGAGAACATCCTAAATCCTTTAACGAGGATCTATTGGAGGGCAAGTCTGGTGCCAGCAGCCGCGG
TAATTCCAGCTCCAATAGCGTATATTAAAGTTGTTGCGGTTAAAAA-
GCTCGTAGTTGGTTCTGCGTGCCACGCTGTCGGTTCACCGCCTGTCGGTGCAACTGGCATGCC-
GTGGCATGTCCTGTCGGTGGCGGATAGGC-TTCGCA-ACG--CTCCCTGC------ACAAC-TATTGTGT---
-AGGTCGT-------CGT-GTCGTCC-TTGTACG-CCGGCT-CAATCCTGCCGCGGTGCT-
CTTTACCGAGTGTCGAGGTAGGCCGACACA-
TTTTACTTTGAACAAATTAGAGTGCTTAAAGCAGGCTCTTAAATC-
AGCCTGAATAGTGGTGCATGGAATAATGGAACAGGACCTTGGTTCTATTTTGTT-GGTTTTAGGA-
ATCCAAGGTAATGATCAATAAGGA-CAGGCGGGGGCATTCGTATTGCGACGTTAGA----------------
-GGTGAAATTCTTGG-
ATCGTCGCAAGACGCACTAGAGCGAAAGCATTTGCCAAGTATGTCTTAATTGATCAAGAACGAAAGTTAGAGG
TTCGAAGGCGATCAGATACCGCCCTAGTTCTAACCATAAACGATGCCAGCCAGCGATCCGCCGATGTTCTTTC
GATGACACGGCGGGGAGCTT-
TCCCCGGGAAACCAAAGCTTTTGGGTTCCGGGGGAAGTATGGTTGCAAAGCTGAAACTTAAAGGAATTGACGG
AAGGGCACCACCAGGAGTGGAGCCTGCGGCTTAATTTGACCCAACACGGGAAACCTCACCAGGCCCGGACATT
GGAA-GGATTGACAGAT-CGATAGCTCTTT-CTCGATT-CAGTGGGT-
AGTGGTGCATGGCCGTTCTTAGTTGGTGGACTGATTTGTCTGGTTAATTCCGATAACGAACGAGACTCTAACC
TATTAACTAGGCGATA-CCGGTATAAAATA--CTACCGGCGAGAT-
TTATTCTTCTTAAGGGGACAGGCGGCTCTCAGCCGCACGAGAATGAGCAATAACAGGTCTGTGATGCCCTTAG
ATGTTCTGGGCCGCACGCGCGCTACACTGAAGGAATCAGCGTGTAC--
CCCCTTGACCGAAAGGTCCGGGTAACCCGTTGAACCTCCTTCGTGCTAGGAATTGGGGCTTGCAATTGTTCCC
CATGAACGAGGAATTCCCAGTAAGCGCGAGTCATAAGCTCGCGTTGATTACGTCCCTGCCCTTTGTACACACC
GCCCGTCGCTACTACCGATTGAATGATTTAGTGAGGTCTTCGGACC-GGCGATA--AC-CGGC-ACC--GAC-
----TCGTTCG---GTATCGAT-TTCGCTGGAAA-
GATGACCGAACTTGATCATTTAGAGGAAGTAAAAGTCGTAACAAGG-TTTCCAGTCA--TTGGGTGA---
AAAACCTAAAGGCGCAATGAAAGT-GTCAG---------------AGACCGCTCTG----------------
------CGGCGCTTTTTGGGAGGATGGG-----C---GT-CC-TAA--------------
CAGGCGCCCCGCACTCCCGGGGCGTCTC--ATTCTC-TTTTCG-AG----
TCGAGACGCACCAGGAGCGTACACGCTGGTACCCGAAAGATGGTGAACTATGCCTGGCCAGGACGAAGCCAGG
GGAAACCCTGGTGGAGGTCC--GAAGCG-ATTCTG--ACGTGCAAATCG-
ATCGTCTGAGCTGGGTATAGGGGCGAAAGACCAATCGAACCATCATG----
ATCAATTAATTTTAAGTCTGACCTGCTCAATGAA----------
TTTATTTAAATTGCTATAGTATTTTAACTATACAAAGGTAGCATAATAATTTGTCTTTTAATTGAAGGCTAGT
ATGAATGGTTGGACGAAAAATAAACTTTCTTAGTTAAAATTATAT----
TGAATTTGATTTTTATGTAAAAAAGCTTAAATTTTA-TTGTGGACGAGAAGACCCTATAGAA--ATTTAAT-
-----------------TAATATTTAAATCCTTTACTATAGTT-
TATTTTATAATATATTAATATATTAATTTATGTTGGGGTGACAGTAGAA-
AATTGATAACTTCTATTTATTAAATTT--TCATAAATTAGTGTAT-----TATTGATCCTTT-----
TTTATGGATT--AAAAGATTAATTTACCTTAGGGATAAC
Rhytidoporus_indentatus
ATGCAAGTCTCAGTACGAGCTGACT-
TAAAGTGAAACCGCAAAAGGCTCAGTAAACCGGTCATAGTTTACCGGA-TATATG-CCACTC-
ACGTGGATAACTGTGGTAATTCTAGAGCTAATACATGCAACC-AAGTCCCGACCAGCG---
ATGGAAAGGACGCTTTTATTGGATCAAAACCATTCGCCCGGCTCGCCC-GGGC------
GTCTGCTTGATGACTCT-AGATAAC-ACCACA-GATCGCACGGTCACAGTACCGGC-
GACGTATCCGTCAAATGTCTGACTTATCAGCTGTCGATGGTAGACTCTGTGTCTACCATGGCGCTCACGGTA

ACGGGGAATCAGGGTTCGATTCCGGAGAGGGAGCCTGAGAAACGGCTACCACATCCAAGGAAGGCAGCAGGCA
CGCAAATTACCCACTCCCGGCACGGGGAGGTAGTGACAAAAAATAACGATACGGGACTCATCCGAGGCCCCGT
AATCGGAATGAGAACACCCTAAATCCTTTAACGAGGATCTATTGGAGGGCAAGTCTGGTGCCAGCAGCCGCGG
TAATTCCAGCTCCAATAGCGTATATTAAAGTTGTTGCGGTTAAAAA-
GCTCGTAGTTGGTTCTGCGTGCCACGCTGTCGGTTCACCGCCTGTCGGTGCAACTGGCATGCC-
GTGGCATGTCCTGTCGGTGGCCGAAAGGC-TTCGCG-GCGCTCCCTATG-------CGTTCAC-GCGTGT---
-AGGTCGTTGC--GCCGCTGTTGTCC-TTGTAGG-CCGGCT-CAATCCTACCGCGGTGCT-
CTTTGCCGAGTGTCGAGGTAGGCCGACACT-
GTTTACTTTGAACAAATTAGAGTGCTTCAAGCAGGCTCTGAAAGC-
AGCCTGAATAGTGGTGCATGGAATAATGGAACAGGACCTTGGTTCTATTTTGTT-GGTTTCCGGA-
ATCCAAGGTAATGATCAATAAGGA-CAGGCGGGGGCATTCGTATTGCGACGTTAGA-----------------
-GGTGAAATTCTTGG-
ATCGTCGCAAGACGCACTAGAGCGGCAGCATTTGCCAAGTATGTCTTAATTGATCAAGAACGAAAGTTAGAGG
TTCGAAGGCGATCAGATACCGCCCTAGTTCTAACCATAAACGATGCCAGCCAGCGATCCGCCGATGTTCCTCC
GATGACTCGGCGGGGAGCTT-
TCCCCGGGAAACCAAAGCTTTTGGGTTCCGGGGGAAGTATGGTTGCAAAGCTGAAACTTAAAGGAATTGACGG
AAGGGCACCACCAGGAGTGGAGCCTGCGGCTTAATTTGACTCAACACGGGAAACCTCACCAGGCCCGGACATT
GAAA-GGATTGACAGAT-CGATAGCTCTTT-CTCGATT-CAGTGGGT-
AGTGGTGCATGGCCGTTCTTAGTTGGTGGAGTGATTTGTCTGGTTAATTCCGATAACGAACGAGACTCTGACC
TATTAACTAGGCGCTC-CCGGTATAA-ATA-CCTACCGGCGAGAT-
TTATTCTTCTTAAGGGGACAGGCGGCTCTTAGCCGCACGAGATTGAGCAATAACAGGTCTGTGATGCCCTTAG
ATGTTCTGGGCCGCACGCGCGCTACACTGAAGGAATCAGCGTGTGC--
TCCCCTGTCCGAAAGGACCGGGCAACCCGCTGAACCTCCTTCGTGCTAGGAATTGGGGCTTGCAATTGTTCCC
CATGAACGAGGAATTCCCAGTAAGCGCGAGTCATAAGCTCGCGTTGATTACGTCCCTGCCCTTTGTACACACC
GCCCGTCGCTACTACCGATTGAATGATTTAGTGAGGTCTTCGGACC-GGCGGTA--AC-CGGC-ACC--GGC-
----TCGTTCGG---TATCGGT-TTCGCTGGAAA-
GATGACCGAACTTGATCATTTAGAGGAAGTAAAAGTCGTAACAAGG-TTTCCAGTCA--TTGGGTGT---
AAA-CCTAAAGGCGCAATGAAAGT-GTCAG-------------GGGGGCCTCCTT-------------
TAGGAGA-----GCTTCCCGCAGGGAGGATGGGCCGCC----GTACAATTATT--------TGT--
CGGAGCTCCGCACTCCCGGGGCGTCTC--GTTCTC-ATTACG-AG----
TGGAGACGCACCAGGAGCGCACACGCTGGTACCCGAAAGATGGTGAACTATGCCTGGCCAGGACGAAGCCAGG
GGAAACCCTGGTGGAGGTCC--GAAGCG-ATTCTG--ACGTGCAAATCG-
ATCGTCTGAGCTGGGTATAGGGGCGAAAGACCAATCGAACCATCTGT-TTGTATA-A---
TATCAGGTCAGACCTGCTCAATGATTAGA---
AGTGTTAATTAAATAGCCGCAGTATTTTGACTGTGCGAAGGTAGCATAATCATTTGTCTTTTAATTGTAGGCT
TGTATGAATGGTTAGATGAAACATTAGCTTTCTTTACATCAATTTTTA-----
TAATTTAATGTTTGAGTCAAAAAGCTTAAATTCTT-TCATAGGACGAGAAGACCCTATAGAA--TTTTATT--
--------ATCATATATTTTATTATATTATGGGTATTTATT----ATTATTTTG-TAATAG---
TGGTAATTGTGTTGGGGTGACAGGGAAA-ATGTATTAACTTTCTTTATTTATTGTGT-
TTATTGATTTATATA-----TTAATGATCCTTT---ATT--AAGGATA--
TAAAGATTAAATTACCTTAGGGATAAC
Riptortus_pedestris
ATGCAAGTCTCAGTACGAGCTGACT-
TAAAGTGAAACCGCAAAGGGCTCAGTAAACCGGTCATAGTTTACCGGA-TATATG-CCACTC-
ACGTGGATAACTGTGGTAATTCTAGAGCTAATACATGCAAAC-AAGTCCCGACCAGCG---
ATGGAGAGGACGCTTTTATTGGATCAAAACCATTCGCCCGAGC-GATC-GGGC------
GTCGGCTTGATGACTCT-AGATAAC-ACCACA-GATCGCACGGTCATAGTACCGGT-
GACGTATCCTTCAAATGTCTGACTTATCAGCTGTCGATGGTAGACTCTGTGTCTACCATGGCGCTCACGGGTG
ACGGGGAATCAGGGTTCGATTCCGGAGAGGGAGCCTGAGAAACGGCTACCACATCCAAGGAAGGCAGCAGGCA
CGCAAATTACCCACTCCCGGCACGGGGAGGTAGTGACAAAAAATAACGATACGGGACTCATCCGAGGCCCCGT
AATCGGAATGAGAACACCCTAAATCCTTTAACGAGGATCTATTGGAGGGCAAGTCCGGTGCCAGCAGCCGCGG
TAATTCCGGCTCCAATAGCGTATATTAAAGTTGTTGCGGTTAAAAA-
GCTCGTAGTTGGTTCTGCGTGCCGCGCTGTCGGTTCACCGCCTGCCGGTGCAACTGGCATGCC-
GCGGCATGTCCTGTCGGTGGCGGACAGGC-TTCCCG-GCGTGCCCTACGCGT----TTA-AC-AGCGTGT---
-AGGCCGCCGC--GCCGGGT-TGTCC-TTGAACG-CCGGCT-CAATCCTACCGCGGTGCT-
CTTTACCGAGTGTCGAGGTAGGCCGACACT-
GTTTACTTTGAACAAATTAGAGTGCTTAAAGCAGGCTCTTAAATC-
AGCCTGAATAGTGGTGCATGGAATAATGGAACAGGACCTTGGTTCTATTTTGTT-GGTTTTCGGA-

ATCCAAGGTAATGATCAATAAGGA-CAGGCGGGGGCATTCGTATTGCGACGTTAGA-----------------
-GGTGAAATTCTTGG-
ATCGTCGCAAGACGCACTAGAGCGAAAGCATTTGCCAAGTATGTCTTAATTGATCAAGAACGAAAGTTAGAGG
TTCGAAGGCGATCAGATACCGCCCTAGTTCTAACCATAAACGATGCCAGCCAGCGATCCGCCGATGTTCCTCC
GATGACTCGGCGGGGAGCTT-
TCCCCGGGAAACCAAAGCTTTTGGGTTCCGGGGGAAGTATGGTTGCAAAGCTGAAACTTAAAGGAATTGACGG
AAGGGCACCACCAGGAGTGGAGCCTGCGGCTTAATTTGACTCAACACGGGAAACCTCACCAGGCCCGGACATT
GGAA-GGATTGACAGAT-CGATAGCTCTTT-CTCGATT-CAGTGGGT-
AGTGGTGCATGGCCGTTCTTAGTTGGTGGACTGATTTGTCTGGTTAATTCCGATAACGAACGAGACTCTAGCC
TATTAACTAGGCGTTG-CCGGTATAAA-CA-GCTACCGGCGAGAT-
TTTTTCTTCTTAAGGGGACAGGCGGCTCTCAGCCGCACGAGATTGAGCAATAACAGGTCTGTGATGCCCTTAG
ATGTTCTGGGCCGCACGCGCGCTACACTGAAGGAATCAGCGTGTGC--
TCCCCTGTCCGAAAGGTCCGGGTAACCCGCTGAACCTCCTTCGTGCTAGGAATTGGGGCTTGCAATTGTTCCC
CATGAACGAGGAATTCCCAGTAAGCGCGAGTCATAAGCTCGCGTTGATTACGTCCCTGCCCTTTGTACACACC
GCCCGTCGCTACTACCGATTGAATGATTTAGTGAGGTCTTCGGACC-GGCGATA--AC-CGGC-ACC--
GACTC-----GTTCGG---TATCGAT-TTCGCTGGAAA-
GATGACCGAACTTGATCATTTAGAGGAAGTAAAAGTCGTAACAAGG-TTTCCAGTCA--TTGGGCGT---
AAAACCCAAAGGCGCAATGAAAGT-GTCTG---------------AGAGCGCTC-----------------
GCGC--------TCTCGGCGGGAGGATGGGCTC-CC---GGCCTCCCGGC-------------
CGGTGCCCCGCACTCCCGGGGCGTCTC--ATTCTC-ATTGCG-AG----
TCGAGACGCACCAGGAGCGCACACGCTGGTACCCGAAAGATGGTGAACTATGCCTGGCCAGGACGAAGCCAGG
GGAAACCCTGGTGGAGGTCC--GAAGCG-ATTCTG--ACGTGCAAATCG-
ATCGTCTGAGCTGGGTATAGGGGCGAAAGACCAATCGAACCATCTAG----
ATTTAATAATTTTAGGTCTAGTCTGCCCAATGATTTA------------
TTTAATGGCTGCAGTATTTTAACTGTACGAAGGTAGCATAATCATTTGTCTTTTAATTGGAGGCTTGTATGAA
TGGCTGGACGAGGAACTTACTTTTTTTATTTAA-ATTTTA----
TGAATTTAATTTTTTAGTCAAAAAGCTAAAATTTTT-TTGCAAGACGAGAAGACCCTATAGAA--TTTTATT-
----------TATTTATATTAATTAAATATT---AAGTTATTT---AACTATTTTATTTATATAAATAAA-
TTTTGTTGGGGTGACAGTGAAA-TTTAAATAACTTTCATATATA-CAAT---TCATTGATTTATGTAAA----
ATT-GATCCCTTT---TTATTAGGATT--ACTAGATTAAATTACCTTAGGGATAAC
Sastragala_sp._scutellata
ATGCAAGTCTCAGTACGAGCTGACT-
TAAAGTGAAACCGCAAAAGGCTCAGTAAACCGGTCATAGTTTACCGGA-TATATG-CCACTC-
ACGTGGACAACTGTGGTAATTCTAGAGCTAATACATGCAACC-AAGTCCCGACCAGCG---
ATGGAAAGGACGCTTTTATTGGATCAAAACCATTCGCCCGGCCCCGCCCGGGC------
GTCTGCTTGATGACTCT-AGATAAC-ACCACA-GATCGCACGGTCATAGTACCGGC-
GACGTATCCGTCAAATGTCTGACTTATCAGCTGTCGATGGTAGACTCTGTGTCTACCATGGCGCTCACGGGTA
ACGGGGAATCAGGGTTCGATTCCGGAGAGGGAGCCTGAGAAACGGCTACCACATCCAAGGAAGGCAGCAGGCA
CGCAAATTACCCACTCCCGGCACGGGGAGGTAGTGACAAAAAATAACGATACGGGACTCATCCGAGGCCCCGT
AATCGGAATGAGAACACCCTAAATCCTTTAACGAGGATCTATTGGAGGGCAAGTCTGGTGCCAGCAGCCGCGG
TAATTCCAGCTCCAATAGCGTATATTAAAGTTGTTGCGGTTAAAAA-
GCTCGTAGTTGGTTCTGCGTGCCACGCTGTCGGTTCACCGCCTGTCGGTGCAACTGGCATGCC-
GTGGCATGTCCTGTCGGTGGCTTCACAGGCTTCGTG-GCGCGCCCTATG-------CGTT---AACACGT-G-
TAGGTAGTTGC--GCCGC-GTTGTTC-TTGTAGG-CCGGCT-CAATCCTGCCGCGGTGCT-
CTTTACCGAGTGTCGAGGTGGGCCGACACT-
GTTTACTTTGAACAAATTAGAGTGCTTCAAGCAGGCTCTGAAAGC-
AGCCTGAATAGTGGTGCATGGAATAATGGAACAGGACCTTGGTTCTATTTTGTT-GGTTTTAGGA-
ATCCAAGGTAATGATCAATAAGGA-CAGGCGGGGGCATTCGTATTGCGACGTTAGA-----------------
-GGTGAAATTCTTGG-
ATCGTCGCAAGACGCACTAGAGCGAAAGCATTTGCCAAGTATGTCTTAATTGATCAAGAACGAAAGTTAGATG
CTCGAAGGCGATCAGATACCGCCCTAGTTCTAACCATAAACGATGCCAGCCAGCGATCCGCCGATGTTCCTCC
GATGACTCGGCGGGGAGCTT-
TCCCCGGGAAACCAAAGCTTTTGGGTTCCGGGGGAAGTATGGTTGCAAAGCTGAAACTTAAAGGAATTGACGG
AAGGGCACCACCAGGAGTGGAGCCTGCGGCTTAATTTGACTCAACACGGGAAACCTCACCAGGCCCGGACATT
GGAA-GGATTGACAGAT-CGATAGCTCTTT-CTCGATT-CAGTGGGT-
AGTGGTGCATGGCCGTTCTTAGTTGGTGGACTGATTTGTCTGGTTAATTCCGATAACGAACGAGACTCTGACC
TATTAACTAGGCGCTC-CCGGTATAC-ATA-CCTACCGGCGAGAT-
TTTTTCTTCTTAAAGGGACAGGCGGCTCTTAGCCGCACGAGATTGAGCAATAACAGGTCTGTGATGCCCTTAG

ATGTTCTGGGCCGCACGCGCGCTACACTGAAGGAATCAGCGTGTTT--
TCCCCTGTCCGAAAGGACCGGGCAACCCGCTGAACCTCCTTCGTGCTAGGAATTGGGGCTTGCAATTGTTCCC
CATGAACGAGGAATTCCCAGTAAGCGCGAGTCATAAGCTCGCGTTGATTACGTCCCTGCCCTTTGTACACACC
GCCCGTCGCTACTACCGATTGAATGATTTAGTGAGGTCTTCGGACC-GGCGGTA--AC-CGGC-ACC--
GGCA-----CGTTCGG---TATCGGT-TTCGCTGGAAR-
GATGACCGAACTTGATCATTTAGAGGAAGTAAAAGTCGTAACAAGG-TTTCCAGTCA--TTGGGTTCT--
AAAACCTAAAGGCGCAATTAACGT-GTC-------------AGGGGGGCCTCCT-------------
CAGGGAGG-----TCTCCCCGCGGGGAGGATGGGCCACC----GCACAGTTA-C--------TGT--
CGGAGCCCCGCACTCCCGGGGCGTCTC--GTTCTC-ATCACG-AG----
TAGAGACGCACCAGGAGCGCACACGCTGGTACCCGAAAGATGGTGAACTATGCCTGGCCAGGACGAAGCCAGG
GGAAACCCTGGTGGAGGTCC--GAAGCG-ATTCTG--ACGTGCAAATCG-
ATCGTATGAGCTGGGTATAGGGGCGAAAGACCAATCGAACCATCTTG--ATTAATTT--
ATATTAGGTTTTACCTGCTCAATGATAATTAAA-----
TTATTAAATAGCCGCAGTATATTGACTGTGCAAAGGTAGCATAATCATTTGTCTTTTAATTGAGGACTTGTAT
GAATGGTTTGATGAGATATTGACTTTCTTTA---TATAAAATTAA--
AAAAATTAATTTTTTAGTTAAAAGGCTAAAATAATT-TTAAGGGACGAGAAGACCCTATAGAA--
TTTTATACTTTTTAAA---------TT-TATAATGTTTCTGGTTATTTTTA----A-GTTTTATTTTTATA--
--AGTATTTTGTTGGGGTGACAGGGAAA-TTTCTTAAACTTTCTTTATTTATACA---
TTATTAATGAATAAAT-----TTTTGATCCTA-----TTTTAAGGATT--
TTAAGATTAAATTACCTTAGGGATAAC
Scaptocoris_divergens
GTGCTAGACTCAGTACCACCTGTCT-
TAAAGTGAATCTGCAAAAGGCTCACTAAACCGGTGATTGTGGACCGGT-CATACG-CCACTT-
ACGTGACAGACTGTGGTAATAACAGAGCTTATCGGGCTTAGA-AACTCCTAGCCAGGG---
ATGGAAAGCACGCATTTATGCCATCAAAGCCATTCGCCCGGCTCGCCC-GCGC------
GGCTGCTTTATGCCTCA-AGATAAC-CACACA-GATAGAACGGTCACGGTACCGGC-
GACTTATCCGTCAAATGTCTGACTTAGCAGCTGTCGGTGGTAGACTCTGTGTCTACCATGGCGCTCACGGGTA
ACGGGGAATCAGGGTTCAATTCCGGAGAGGGAGCCTGAGAGACGGCTACCACATCCAAGGAAGGCAGCAGGCA
CGCAAATTACCCACTCCCGGCACGGGGAGGTAGTGACAAAAAATAACGATACGGGACTCATCCGAGGCCCCGT
AATCGGAATGAGAACACCCTAAATCCTTTAACGAGGATCTATTGGAGGGCAAGTCTGGTGCCAGCAGCCGCGA
TAATTCCAGCTCCGATAGCGTATATTAAAGTTGTTGCGGTTAAAAA-
GCTCGTAGTTGGTTCTGCGTGCCACGCTGTCGGTTCACCGCCTGTCGGTGCAACTGGCGTGCC-
GTGGCATGTCCTGTCGGTGGCTGACAGGC-TTCGCG-GCGCGCCCTATCC--------GTCTACCGGTCT---
-AGGCCGCCGC--GCCGGGTCTGCCC-TTGTAGG-CCGTCT-CAATCCAACCGCGGTGCT-
CTTTGCCGAGTGTCGAGGTAGGCCGACACT-
GTTTACTTTGAACAAATTAGAGTGCTTTAAGCAGGCTCTGGAATC-
AGCCTGAATAGTGGTGCATGGAATAATGGAACAGGACCTTGGTTCTATTTTGTT-GGTTTCCGGA-
ATCCTAGGTAATGATCGATAAGGA-CAGGCGGGGGCATTCGTATTGCGACGTTAGA-----------------
-GGTGAAATTCTTGG-
ATCATCGCAAGACGCACTAGAGCGAAAGCATTTGCCAAGTATGTCTTAATTGATCAAGAACGAAAGTTAGAGG
TTCGAAGGCGATCAGATACCGCCCTAGTTCTAACCATAAACGATGCCAGCCAGCGATCCGCCGATGTTCCTCC
GATGACTCGGCGGGGAGCTT-
TCCCCGGGAAACCAAAGCTTTTGGGTTCCGGGGGAAGTATGGTTGCAAAGCTGAAACTTAAAGGAATTGACGG
AAGGGCACCACCAGGCGTGGAGCCTGCGGCTTAATTTGACTCAACACGGGAAAGCTCACCAGGCCCGGACATT
GGGA-GGATTGACAGAT-CGATAGCTCTTT-CTCGATT-CAGTGGGT-
AGTGGTGCATGGCCGTTCTTAGTTGGTGGACTGATTTGTCTGGTTGATTCCGTTAACGAACGAGACTCTGGCC
TATTAAATAGGCGCTT-CCGGTATGG-ACA-CCTACCGGCGAGAT-
TTATTCTTCTTAAGGGGACCGGCGGCTCCAAGCCGCACGAGATTGAGCAATGACAGGTCAGTGATGCCCTTAG
ATGTTCTGGGCCGCACGCGCGCTACACTGAAGGAATCAGCGTGTGC--
TCCCCTGTCCGAAAGGACCGGGTAACCCGCTGAAACTCCTTCGTGCTAGGAATCGGGGCTTGCAATTCTTCCC
CGTGAACGAGGAATGCCCAGTAAGTGCGAGTCATAAGCTCGCGCTGATTACGTCCCTGCCCTTTGTCCACACC
GCCCGTCGCTACTACCGATTGAATGATTTAGTGAGGTCTTCGGACC-GGCGGCG--ACGCGGC-ACC--GGC-
----TCGCCCGG---TGCCGGT-ACCGCTGTAAA-
GATGACCGAACTTGATCATTTAGAGGAAGTAAAAGTCGTAACAAGG-TTTCCAGTCA--TTGGATGC---
AAAACCTAACGGCGCAATGAAAGT-GTCAGT-------------CCGGCCGCGCC-------------
CACGGGCC---GGCCGGCGCCGGGGAGGATGGGCCCTC----GTACAATTATT----------
GGGCGGGGCCCCGCACTCCCGGGGCGTCTCGAGCCCTC-CCCGCGAGG---
GCTGAGACGCACCAGGAGCGCGCGCGCTGGTACCCGAAAGATGGTGAACTATGCCTGGTCAGGACGAAGCCAG

GGGAAACCATGGTGGAGGTCC--GAAGCG-ATTCTG--ACGTGCAAATCG-
ATCGTCTGAGCTGGGTATAGGGGCGAAAGACCAATCGAACTATCTTG-------
TTTAAATATTAGGTTTAACCTGCTCAATGATAATT---------
AATTAAATAGCTGCAGTATTTTGACTGTACAAAGGTAGCATAATCATTTGTTTTTTAATTGAAGACTTGTATG
AATGGTTGGACGAGGTACTTACTTTCTTAA---TAACAAAAA---
TTGAATTTAAATTTTAGGTTAAAATGCTTAAATTTAT-TTATGGGACGAGAAGACCCTATAGAA--
TTTTATTTTATT------------TAAGTGTTAATAGTAACTTTAATT-AT---
TCTATCTTTTTTACTTTAATA-GAATTTTGTTGGGGTGACAGGGAAA-TTTTATTAACTTTCTTT--
ATTTAAGTT-TCATAAATTCATGTG----TTTTAAGATCCTTTAGATT--
ATGAAAAAAGATTTAATTACCTTACGTTGAATAAACC
Sehirus_luctuosus
ATGCAAGTCTCAGTACGAGCTTACT-
TAAAGTGAAACCGCAAAAGGCTCAGTAAACCGGTCATAGTTTACCGGA-TATATG-CCACTC-
ACGTGGATAACTGTGGTAATTCTAGAGCTAATACATGCAACT-AAGTCCCGACCAGCG---
ATGGAAAGGACGCTTTTATTGGATCAAAACCATTCGCCCGGTTCGCCC-GGGC------
GTCTGCTTGATGACTCT-AGATAAC-ACCACA-GATCGCACGGTCATTGTACCGGC-
GACGTATCCGTCAAATGTCTGACTTATCAGCTGTCGATGGTAGACTCTGTGTCTACCATGGCGCTCACGGGTA
ACGGGGAATCAGGGTTCGATTCCGGAGAGGGAGCCTGAGAAACGGCTACCACATCCAAGGAAGGCAGCAGGCA
CGCAAATTACCCACTCCCGGCACGGGGAGGTAGTGACAAAAAATAACGATACGGGACTCATCCGAGGCCCCGT
AATCGGAATGAGAACACCCTAAATCCTTTAACGAGGATCTATTGGAGGGCAAGTCTGGTGCCAGCAGCCGCGG
TAATTCCAGCTCCAATAGCGTATATTAAAGTTGTTGCGGTTAAAAA-
GCTCGTAGTTGGTTCTGCGTGCCACGCTGTCGGTTCACCGCCTGTCGGTGCAACTGGCATGCC-
GTGGCATGTCCTGTCGGTGGCCTACTGGT-TTCGCG-GCGCACCCTATG-------CGTT---AACTCGTTG-
TAGGTCGTTGCT-GCCGC-GTTGTCC-TAGTAGG-CCGGCT-CAATCCTACCGCGGTGCT-
CTTTACCGAGTGTCGAGGTAGGCCGACACT-
GTTTACTTTGAACAAATTAGAGTGCTTAAAGCACGCTCTGAAAGC-
AGCCTGAATAGTGGTGCATGGAATAATGGAACAGGACCTTGGTTCTATTTTGTT-GGTTTTCTGA-
ATCCAAGGTAATGATCAATAAGGA-CAGGCGGGGGCATTCGTATTGCGACGTTAGA-----------------
-GGTGAAATTCTTGG-
ATCGTCGCAAGACGCACTAGAGCGAAAGCATTTGCCAAGTATGTCTTAATTGATCAAGAACGAAAGTTAGAGG
TTCGAAGGCGATCAGATACCGCCCTAGTTCTAACCATAAACGATGCCAGCCAGCGATCCGCCGATGTTCCTCC
GATGACTCGGCGGGGAGCTT-
CCCCCGGGAAACCAAAGCTTTTGGGTTCCGGGGGAAGTATGGTTGCAAAGCTGAAACTTAAAGGAATTGACGG
AAGGGCACCACCAGGAGTGGAGCCTGCGGCTTAATTTGACTCAACACGGGAAACCTCACCAGGCCCGGACATT
GGAA-GGATTGACAGAT-CGATAGCTCTTTTCTCGATT-CAGTGGGT-
AGTGGTGCATGACCGTTCTTAGTTGGTGACTGATTTTGTCTGGTTAATTCCGATAACGAACGAGACTCTAACC
TATTAACTAGGCGCTC-CCGGTATAC-ATA-CCTACCGGCGAGAC-
TTATTCTTCTTAAGGGGACAGGCGGCTCTTAGCCGCACGAGATTGAGCAATAACAGGTCTGTGATGCCCTTAG
ATGTTCTGGGCCGCACGCGCGCTACACTGAAGGAATCAGCGTGTTC--
TCCCCTGTCCGAAAGGACCGGGCAACCCGCTGAACCTCCTTCGTGCTAGGAATTGGGGCTTGCAATTGTTCCC
CATGAACGAGGAATTCCCAGTAAGCGCGAGTCATAAGCTCGCGTTGATTACGTCCCTGCCCTTTGTACACACC
GCCCGTCGCTACTACCGATTGAATGATTTAGTGAGGTCTTCGGACC-GGCGGTA--AC-AGGC-ATC--
GGCT-----CGCCCTT---TATCTGT-TCCGCTGGAAA-
GATGACCGAACTTGATCATTTAGAGGAAGTAAAAGTCGTAACAAGG-TTTCCAGTCA--TTGGGTGT---
AAAACCTAAAGGCGCAATGAAAGT-GTC-------------AGGGGGCCTTTCCCG---------
TGAGGGGGAG----GTCTCCCCGCTGGGAAGATGGGTTACC----GTACTCTTAAT--------TGT--
CGGGGCCCCGCACTCCCGGGGCGTCTC--GTTCTC-ATTGCG-AG----
TAGAGACGCACCAGGAGCGCACACGCTGGTACCCGAAAGATGGTGAACTATGCCTGGCCAGGACGAAGCCAGG
GGAAACCCTGGTGGAGGTCC--GAAGCG-ATTCTG--ACGTGCAAATCG-
ATCGTCTGAGCTGGGTATAGGGGCGAAAGACCAATCGAACCATCTTGTTATATTTTT--
ATTTTAAGTCAAGCCTGCTCAATGATTA--AT------
TAATTAAATAGCCGCAGTATTTTGACTGTGCAAAGGTAGCATAATCATTTGTCTATTAATTGTAGGCTGGTAT
GAATGGCTAGATGAAATATTAACTTTCTTTATTTTATATTTATT---
TGAATTTAATTTTTAGGTTAAAAAGCTTAAATGTTTATTGTAGGACGAGAAGACCCTATAGAA--
TTTTATTTAATT--AT--------TGGTTTGATATAAATTATTTGATTATT----ATTTA-AATTAATAAT--
--TAAATTTTGTTGGGGTGACAGGGAAA-ATATAATAACTTTCTTTATTTATTA--T-
TTATAAATTAATATAT-----TAAAAATCCTT-----TATATAAGGAA--
ATAAGATTAAATTACCTTAGGGATAAC

Stibaropus_indonesicus
ATGCAAGTCTCAGTACGAGCTGTCT-
TAAAGTGAAACCGCAAAAGGCTCAGTAAACCGGTCATAGTTTACCGGA-TATATG-
CCACTTTACGTGGATAACTGTGGTAATTCTAGAGCTAATACATGCAACA-AAGTCCCGACCAGCG---
ATGGAAAGGACGCTTTTATTGGATCAAAGCCATTCGCCCGGCTCGCCC-GGGC------
GTCTGCTTGATGACTCT-AGATAAC-CACACA-GATCGAACGGTCACAGTACCGGC-
GACTTATCCGTCAAATGTCTGACTTATCAGCTGTCGATGGTAGACTCTGTGTCTACCATGGCGCTCACGGGTA
ACGGGGAATCAGGGTTCGATTCCGGAGAGGGAGCCTGAGAAACGGCTACCACATCCAAGGAAGGCAGCAGGCA
CGCAAATTACCCACTCCCGGCACGGGGAGGTAGTGACAAAAAATAACGATACGGGACTCATCCGAGGCCCCGT
AATCGGAATGAGAACACCCTAAATCCTTTAACGAGGATCTATTGGAGGGCAAGTCTGGTGCCAGCAGCCGCGA
TAATTCCAGCTCCAATAGCGTATATTAAAGTTGTTGCGGTTAAAAA-
GCTCGTAGTTGGTTCTGCGTGCCACGCTGTCGGTTCACCGCCTGTCGGTGCAACTGGCATGCC-
GTGGCATGTCCTGTCGGTGGCTGACAGGC-TTCGCC-GCGCACCCTATGC--------GTTTACGCGTGT---
-AGGTAGTCGC--GCTGACTATGTCC-TTGTAGG-CCGGCT-CAATCCTACCGCGGTGCT-
CTTTGCCGAGTGTCGAGGTAGGCCGACACT-
GTTTACTTTGAACAAATTAGAGTGCTTCAAGCAGGCTCTGAAAGC-
AGCCTGAATAGTGGTGCATGGAATAATGGAACAGGACCTTGGTTCTATTTTGTT-GGTTTCCGGA-
ATCCAAGGTAATGATCAATAAGGA-CAGGCGGGGGCATTCGTATTGCGACGTTAGA-----------------
-GGTGAAATTCTTGG-
ATCGTCGCAAGACGCACTAGAGCGAAAGCATTTGCCAAGTATGTCTTAATTGATCAAGAACGAAAGTTAGAGG
TTCGAAGGCGATCAGATACCGCCCTAGTTCTAACCATAAACGATGCCAGCCAGCGATCCGCCGATGTTCCTCC
GATGACTCGGCGGGGAGCTT-
TCCCCGGGAAACCAAAGCTTTTGGGTTCCGGGGGAAGTATGGTTGCAAAGCTGAAACTTAAAGGAATTGACGG
AAGGGCACCACCAGGAGTGGAGCCTGCGGCTTAATTTGACTCAACACGGGAAACCTCACCAGGCCAGGACATT
GGAA-GGATTGACAGAT-CGATAGCTCTTT-CTCGATT-CAGTGGGT-
AGTGGTGCATGGCCGTTCTTAGTTGGTGGACGGATTTGTCTGGTTGATTCCGTTAACGAACAAGACTCTGGCC
TATTAAATAGGCGCTT-CCGGTAGGG-ACA-CCTACCGGCGAGAT-
TTATTCTTCTTAAGGGGACCGGCGGCTCCAAGCCGCACGAGATTGAGCAATGACAGGTCAGTGATGCCCTTAG
ATGTTCTGGGCCGCACGCGCGCTACACTGAAGGAATCAGCGTGTGC--
TCCCCTGTCCGAAAGGACCGGGTAACCCGCTGAACCTCCTTCGTGCTATGAATCGGGGCTTGCAATTCTTCCC
CGTGAACGAGGAATGCCCAGTAAGTGCGAGTCATAAGCTCGCGCTGATTACGTCCCTGCCCTTTGTCCACACC
GCCCGTCGCTACTACCGATTGAATGATTTAGTGAGGTCTTCGGACC-GGCGGCG--ACGCGGC-ACC--GGC-
----TCGCCCGG---TGCCGGT-ACCGCTGTAAA-
GATGACCGAACTTGATCATTTGGAGGAAGTAAAAGTCGTAACAAGG-TTTCCAGTCA--TTGGGTGC---
AAAACCTAAAGGCGCAATGAAAGT-GTCAGGT----------GGGACTCCCAATT-------------
CAGGGGT------CCCTCCACGGGGAGGATGGGCCGCC----GCACAATTATTT----------
GTCGGAGCCCCGCAATCCCGGGGCGTCTC--GTCCTC-ATCACGAGT-----
AGAGACGCACCAGGAGCGCACACGCTGGTACCCGAAAGATGGTGAACTATGCCTGGCCAGGACGAAGCCAGGG
GAAACCCTGGTGGAGGTCC--GAAGCG-ATTCTG--ACGTGCAAATCG-
ATCGTCTGAGCTGGGTATAGGGGCGAAAGACCAATCGAACTATCCAG-------TC-
ATATTTTACGTCTGGCCTGCTCA-TGATTATTTAT----
ATAATTAAATAGCCGCAGTATCCTGACTGTGCGAAGGTAGCATAATCATTTGTCTTTTAATTGTAGGCTAGTA
TGAATGGTCGGATGAGGCATCAACTTTCTTTA---TCTTATAAT---
TTGAATTTAAAGTTTGAGTTAAAAGGCTTAAATTTAT-TCGTGGGACGAGAAGACCCTATAGAA--
TTTTATTGTAAT------------TGTG-ATTTTATCCATTTTTGGTTGAT---
ATTTTGGTATGGAGTTGTATTGCAATTGTGTTGGGGCGACAGGGAAA-TTAATGTAACTTTCTTTC-
ATTTATTTA-TTACTGATTAGTATA----TTTAAAGATCCTGTATTAT-----GGATT--
TAAAGATTAAATTACCTTAGGGATAAC
Stictopleurus_punctatonervosus_subviridis
ATGCAAGTCTCAGTACGAGCTGACT-
TAAAGTGAAACCGCAAAAGGCTCAGTAAACCGGTCATAGTTTACCGGA-TATA-G-CCACTC-
ACGTGGATAACTGTGGTAATTCTAGAGCTAATACATGCAAAC-AAGTCCCGACCAGCG---
ATGGAGAGGACGCTTTTATTGGATCAAAACCATTCGCCCGGCTTGCC--GGGC------
GTCGGCTTGATGACTCT-AGATAAC-ACCACA-GATCGCACGGTCACAGTACCGGT-
GACATATCCTTCAAATGTCTGACTTATCAGCTGTCGATGGTAGACTCTGTGTCTACCATGGCGCTCACGGGTG
ACGGGGAATCAGGGTTCGATTCCGGAGAGGGAGCCTGAGAAACGGCTACCACATCCAAGGAAGGCAGCAGGCA
CGCAAATTACCCACTCCCGGCACGGGGAGGTAGTGACAAAAAATAACGATACGGGACTCATCCGAGGCCCCGT
AATCGGAATGAGAACACCCTAAATCCTTTAACGAGGATCTATTGGAGGGCAAGTCTGGTGCCAGCAGCCGCGG

TAATTCCAGCTCCAATGGCGTATATTAAAGTTGTTGCGGTTAAAAA-
GCTCGTAGTTGGTTCTGCGTGCCACGCTGTCGGTTCACCGCCTGCCGGTGCAACTGGCATGCC-
GTGGCATGTCCTGTCGGTGGCGGATGGGC-T-CGTG-GCGTGCCCTATGCGG----TAT-TCCAACGTGT---
-AGGTTGCTAC--GCCGCGA-TGTTC-T-GTTCG-CCGGCT-CAATCCTACCGCGGTGCT-
CTTTACCGAGTGTCGAGGTAGGCCGACACT-
GTTTACTTTGAACAAATTAGAGTGCTTAAAGCAGGCTCTTAAATC-
AGCCTGAATAGTGGTGCATGGAATAATGGAACAGGACCTTGGTTCTATTTTGTT-GGTTTTCGGA-
GTCCAAGGTAATGATCAATAAGGA-CAGGCGGGGGCATTCGTATTGCGGCGTTAGA-----------------
-GGTGAAATTCTTGG-
ATCGTCGCAAGACGCACTAGAGCGAAAGCATTTGCCAAGTATGTCTTAATTGATCAAGAACGAAAGTTAGAGG
TTCGAAGGCGATCAGATACCGCCCTAGTTCTAACCATAAACGATGCCAGCCAGCGATCCGCCGATGTTCCTCC
GATGACTCGGCGGGGAGCTT-
TCCCCGGGAAACCAAAGCTTTTGGGTTCCGGGGGAAGTATGGTTGCAAAGCTGAAACTTAAAGGAATTGACGG
AAGGGCACCACCAGGAGTGGAGCCTGCGGCTTAATTTGACTCAACACGGGAAACCTCACCAGGCCCGGACATT
GGAA-GGATTGACAGAT-CGATAGCTCTTT-CTCGATT-CAGTGGGT-
AGTGGTGCATGGCCGTTCTTAGTTGGTGGACTGATTTGTCTGGTTAATTCCGATAACGAACGAGACTCTAACC
TATTAACTAGGCGTTT-CCGGTATACA-AA-TCTACCGGCGAGAT-
TTATTCTTCTTAAGGGGACAGGCGGCTCTCAGCCGCACGAGATTGAGCAATAACAGGTCTGTGATGCCCTTAG
ATGTTCTGGGCCGCACGCGCGCTACACTGAAGGAATCAGCGTGTGC--
TCCCCTGTCCGAAAGGTCCGGGTAACCCGCTGAACCTCCTTCGTGCTAGGAATTGGGGCTTGCAATTGTTCCC
CATGAACGAGGAATTCCCAGTAAGCGCGAGTCATAAGCTCGCGTTGATTACGTCCCTGCCCTTTGTACACACC
GCCCGTCGCTACTACCGATTGAATGATTTAGTGAGGTCTTCGGACC-GGCGATA--AC-CGGC-ATC--
GACTC-----GTTCGG---TATCGAT-TTCGCTGGAAA-
GATGACCGAACTTGATCATTTAGAGGAAGTAAAAGTCGTAACAAGG-TTTCCAGTCA--TTGGGTGT---
AAAACCTAAAGGCGTAATGAAAGT-GTCAG---------------AGGGCGCTT-----------------
GCGT--------CCTCAGTGGGAGGATGAGCTC-CC---GGCCTTTCGGC-------------
CGGCGCTCCGCACTCCCGGGGCGTTTC--ATTCTC-T-TGCG-AG----
TCGAAACGCACCAGGAGCGCACACGCTGGTACCCGAAAGATGGTGAACTATGCCTGGCCAGGACGAAGCCAGG
GGAAACCCTGGTGGAGGTCC--GAAGCG-ATTCTG--ACGTGCAAATCG-
ATCGTCTGAGCTGGGTATAGGGGCGAAAGACCAATCGAACCATCTTG----ATTT-TA-
ATATAAGGTCGGGCCTGCCCAATGATTTT-A----------
TTTAATGGCCGCAGTATCCTAACTGTGCAAAGGTAGCATAATCATTTGTCTTTTAATTAAAGGCTGGAATGAA
TGGTTGGACGAAGGATTAACTTTTTTTATTTAA-ATTTTA----
TGAATTTAATTTTTTGGTTAAAAAGCTAAAATTTTT-TTGTTAGACGAGAAGACCCTATAGAA--
TTTTAATA----------TTTATATTTAT-TTAGTTATT---TTGATTTTG---
ATAAAATTAGTTATTTATAATTAT-TTTTGTTGGGGTGACAATGAAA-
ATTAAATAACTTTCATTATTTTTTAT---TCATTAATTTATGTATA----TTTTGATCCTATA---TTAT--
GGATT--GTAAGATTAAATTACCTTAGGGATAAC
Thaumastella_elizabethae
ATGCAAGTCTCAGTACGAGCTGACT-
TAAAGTGAAACCGCAAAAGGCTCAGTAAACCGGTCATAGTTTACCGGA-TATATG-CCACTC-
ACGTGGATAACTGTGGTAATTCTAGAGCTAATACATGCAACC-AAGTCCCGACCAGAG---
ATGGAGAGGACGCTTTTATTGGATCAAAACCATTCGCCCGGCTCGCCC-GGGC------
GTCTGTTTGATGACTCT-AGATAAC-ACCACA-
GATCGCACGGTCACAGTACCGGGCGACGTATTCTTCAAATGTCTGACTTATCAGCTGTCGATGGTAGACTCTG
TGTCTACCATGGCGCTCACGGGTAACGGGGAATCAGGGTTCGATTCCGGAGAGGGAGCCTGAGAAACGGCTAC
CACATCCAAGGAAGGCAGCAGGCACGCAAATTACCCACTCCCGGCACGGGGAGGTAGTGACAATAAATAACGA
TACGGGACTCATCCGAGGCCCCGTAATCGGAATGAGAACACCCTAAATCCTTTAACGAGGATCTATTGGAGGG
TAAGTCTGGTGCCAGCAACCGCGGTAATTCCAGCTCCAATAGCGTATATTAAAGTTGTTGCGGTTAAAAAGC
TCGTAGTTGGTTCTGCGTGCCACGCTGTCGGTTCACCGCCTGTCGGTGCAACTGGCATGCC-
GTGGCATGTCCTGTCGGTGGCCGACAGGC-TGCGTGGCGGCGACCTATG-------CGTTAACAACGTGT---
-AGGTACTGTT--GCCG-CGTTGTCC-TTTTAGG-CCGACT-CAATCCTACCTTGGTGCT-
CTTTACCGAGTGTCGAGGTAGGCCGACACT-
GTTTACTTTGAACAAATTAGAGTGCTTCAAGCAGGCTCTGAAAGT-
AGCCTGAATAGTGGTGCATGGAATAATGGAACAGGACCTTGGTTCTATTTTGTT-GGTTTCCGGA-
ATCCAAGGTAATGATCAATAAGGA-CAGGCGGGGGCATTCGTATTGCGACGTTAGA-----------------
-GGTGAAATTCTTGG-
ATCGTCGCAAGACGCACTAGAGCGAAAGCATTTGCCAAGTATGTCTTAATTGATCAAGAACGAAAGTTAGAGG

TTCGAAGGCGATCAGATACCGCCCTAGTTCTAACCATAAACGATGCCAGCCAGCGATCCGCCGATGTTCCTCC
GATGACTCGGCGGGGAGCTT-
TCCCCGGGAAACCAAAGCTTTTGGGTTCCGGGGGAAGTATGGTTGCAAAGCTGAAACTTAAAGGAATTGACGG
AAGGGCACCACAAGGAGTGGGGCCTGCGGCTTAATTTGACTCAACACGGGAAACCTCACCAGGCCCGGACATT
GGAAGG-ATTGACAGAT-CGATAGCTCTTT-CTCGATT-CAGTGGGT-
AGTGGTGCATGGCCGTTCTTAGTTCGTGGACTGATTTGTCTGGTTAATTCCGATAACGAACGAGACTCTAACC
TATTAACTAGGCGCTT-CCGGTACAG-ATA-CTTACCGGCGAGAT-TTATTCTTCTTA-
GGGGACAGGCGGCTCTTAGCCGCACGAGATTGAGCAATAACAGGTCTGTGATGCCCTTAGATGTTCTGGGCCG
CACGCGCGCCACACTGAAGGAATCAGCGTGTGC--
TCCCCTGTCCGAAAGGTCCGGGTAACCCGCTGAACCTCCTTCGTGCTAGGAATTGGGGCTTGCAATTGTTCCC
CATGAACGAGGAATTCCCAGTAAGCGCGAGTCATAAGCTCGCGTTGATCACGTCCCTGCCCTTTGTACACACC
GCCCGTCGCTACTACTGATTGAATGATTTAGTGAGGTCTTCGGACC-GGCGAA---AACCGGC-ACC--GG--
---CTCGTTCGG---TATCGGT-TTCGCTGGAAA-
GATGACCGAACTTGATCATTTAGAGGAAGTAAAAGTCGTAACAAGGGTTTCCAGTCA--TTGGGTG---
AAAAACCTAAAGGCGCAATGAAAGT-GTCAGG------------GGAGGTCACCTTTA-----------
CGGGGGTG---TCCTCTTCGTCGGGAGGATGGGCTCCC----GTCC-TCACGG----------T--
CGGGGCCCCGCACTCCCGGGGCGTTTC--GCCACTCGTTCCG-AG----
TAGAGACGCACCAGGAGCGCACACGCTGGTACCCGAAAGATGGTGAACTATGCCTGGCCAGGACGAAGCCAGG
GGAAACCCTGGTGGAGGTCC--GAAGCG-ATTCTG--ACGTGCAAATCG-
ATCGTCTGAGCTGGGTATAGGGGCGAAAGACCAATCGAACCATCTAT----T----A--
TTATCAGGTTTAACCTGCCCACTGATTTA-------ATTATTTAAAGG-
CCGCAGTATATTAACTGTGCAAAGGTAGCATAATCAATTGTCTTTTAATTGAAGGCTAGAATGAATGGTTGAA
TGAGAGATAGACTTTCTTTGCTATAT-TTATT-----
GAATTTAATTTTTTAGTTAAAAAGCTAAAATTTGTT-TAAAGGACGAGAAGACCCTATAGAA--
TTTTACTCATATAGATG--------AATTATTT-ATTATATTAGGTTATGT---------
AATGTTTATTTTATT-GAGTTTTGTTGGGGTGACAAGGAAAATGTTTATAACTTTCTTTATTTATAATA--
TTATTTATATATATAT----T-AGAGATCCTT-----ATTTTAAGATA--
TAAAGATTAAATTACCTTAGGGATAAC
Thaumastella_namaquensis
ATGCAAGTCTCAGTACGAGCTGACT-
TAAAGTGAAACCGCAAAAGGCTCAGTAAACCGGTCATAGTTTACCGGA-TATATG-CCACTC-
ACGTGGATAACTGTGGTAATTCTAGAGCTAATACATGCAACA-AAGTCCCGACCAGAG---
ATGGAGAGGACGCTTTTATTGGATCAAAACCATTCGCCCGGCTCGCCC-GGGC------
GTCTGTTTGATGACTCT-AGATAAC-ACCACA-
GATCGCACGGTCACAGTACCGGGCGACGTATTCTTCAAATGTCTGACTTATCAGCTGTCGATGGTAGACTCTG
TGTCTACCATGGCGCTCACGGGTAACGGGGAATCAGGGTTCGATTCCGGAGAGGGAGCCTGAGAAACGGCTAC
CACATCCAAGGAAGGCAGCAGGCACGCAAATTACCCACTCCCGGCACGGGGAGGTAGTGACAATAAATAACGA
TACGGGACTCATCCGAGGCCCCGTAATCGGAATGAGAACACCCTAAATCCTTTAACGAGGATCTATTGGAGGG
TAAGTCTGGTGCCAGCAACCGCGGTAATTCCAGCTCCAATAGCGTATATTAAAGTTGTTGCGGTTAAAAA-
GCTCGTAGTTGGTTCTGCGTGCCACGCTGTCGGTTCACCGCCTGTCGGTGCAACTGGCATGCC-
GTGGCATGTCCTGTCGGTGGCCGACAGGC-TGCGTGGCGGCGACCCATT-------C-TTAA--ACGTGT---
-GGGTACTGTT--GCCG-CGTTGTCC-TTTTAGG-CCGACT-CAATCCTACCTTGGTGCT-
CTTTACCGAGTGTCGAGGTAGGCCGACACT-
GTTTACTTTGAACAAATTAGAGTGCTTCAAGCAGGCTCTGAAAGT-
AGCCTGAATAGTGGTGCATGGAATAATGGAACAGGACCTTGGTTCTATTTTGTT-GGTTTCCGGA-
ATCCAAGGTAATGATCAATAAGGA-CAGGCGGGGGCATTCGTATTGCGACGTTAGA-----------------
-GGTGAAATTCTTGG-
ATCGTCGCAAGACGCACTAGAGCGAAAGCATTTGCCAAGTATGTCTTAATTGATCAAGAACGAAAGTTAGAGG
TTCGAAGGCGATCAGATACCGCCCTAGTTCTAACCATAAACGATGCCAGCCAGCGATCCGCCGATGTTCCTCC
GATGACTCGGCGGGGAGCTT-
TCCCCGGGAAACCAAAGCTTTTGGGTTCCGGGGGAAGTATGGTTGCAAAGCTGAAACTTAAAGGAATTGACGG
AAGGGCACCACAAGGAGTGGGGCCTGCGGCTTAATTTGACTCAACACGGGAAACCTCACCAGGCCCGGACATT
GGAAGGTATTGACAGAT-
CGATAGCTCTTTGCTCGTTTACAGTGGGTGAGTGGTGCATGGCCTTTCTTAGTTCGTGGACTGATTTGTCTGG
TTAATTCCGATAACGAACGAGACTCTAACCTATTAACTAGGCGCTT-CCGGTACAG-ATA-
CTTACCGGCGAGAT-TTATTCTTCTTA-
GGGGACAGGCGGCTCTTAGCCGCACGAGATTGAGCAATAACAGGTCTGTGATGCCCTTAGATGTTCTGGGCCG
CACGCGCGCCACACTGAAGGAATCAGCGTGTGC--

TCCCCTGTCCGAAAGGTCCGGGTAACCCGCTGAACCTCCTTCGTGCTAGGAATTGGGGCTTGCAATTGTTCCC
CATGAACGAGGAATTCCCAGTAAGCGCGAGTCATAAGCTCGCGTTGATCACGTCCCTGCCCTTTGTACACACC
GCCCGTCGCTACTACTGATTGAATGATTTAGTGAGGTCTTCGGACC-GGCGAA---AACCGGC-ACC--GG--
---CTCGTTCGG---TATCGGT-TTCGCTGGAAA-
GATGACCGAACTTGATCATTTAGAGGAAGTAAAAGTCGTAACAAGGGTTTCCAGTCA--TTGGGTG---
AAAAACCTAA-GGCGCAATGAAAGTTGTCGGG------------GGAGGGCGCCCTT------------
CGCGGGTG---CCCTTCCCGTTGGGAGGATGGGCTCCC----GTCC-TCACGG----------T--
CGGGGCCCCGCACTCCCGGGGCGTTTC--GCCGCTCGTCCCG-AG----
CAGAGACGCACCAGGAGCGCACACGCTGGTACCCGAAAGATGGTGAACTATGCCTGGCCAGGACGAAGCCAGG
GGAAACCCTGGTGGAGGTCC--GAAGCG-ATTCTG--ACGTGCAAATCG-
ATCGTCTGAGCTGGGTATAGGGGCGAAAGACCAATCGAACCATCTGT----TGGTTA--
ATATTAGGTCTGGCCTGCCCACTGATTTA-------
GTAGATTAAAGGGCCGCAGTATACTGACTGTGCAAAGGTAGCATAATCAATTGTCTTTTAATTGAAGGCTGGT
ATGAATGGTTGGATGAAATGCATGCTTTCTTTATTTAATGTTATT-----
GAATTTAATTTTTAAGTTAAAAAGCTTAAATTTTGT-TGAAGGACGAGAAGACCCTATAGAA--
TTTTATCTAACTTGGT-----------TTTAGT-ATT-TAAAGGTTGATTT---------
TATGTGAATTGAGTT-GGATTTTGTTGGGGTGATGGGGAAA-TTTATATAACTTTCTTTTTATA-AATA--
TTATTTGTGAGTATAT----TTAAAGATCTTT-----ATTTTAGGATA--
ATAAGATTAAATTACCTTAGGGATAAC
Thyreocoris_scarabaeoides
ATGCAAGTCTCAGTACGAGCTGAAT-
TAAAGTGAAACCGCAAAAGGCTCAGTAAACCGGTCATAGTTTACCGGA-TATATG-CCACTC-
ACGTGGATAACTGTGGTAATTCTAGAGCTAATACATGAACTC-AAGTCCCTACCAGCG---
ATGGAAAGGACTCTTTTATTGGATCAAAGCCATTCGCCCGGCTCGCCC-GGGC------
GCACGCTTGATGACTCT-AGATAAC-ACCACAAGATCGCACGGTCACAGTACCGGC-
GACGTATCCGTCAAATGTCTGACTTATCAGCTGTCGATGGTAGACTCTGTGTCTACCATGGCGCTCACGGGTA
ACGGGGAATCAGGGTTCGATTCCGGAGAGGGAGCCTGAGAAACGGCTACCACATCCAAGGAAGGCAGCAGGCA
CGCAAATTACCCACTCCCGGCACGGGGAGGTAGTGACAAAAAATAACGATACGGGACTCATCCGAGGCCCCGT
AATCGGAATGAGAACACCCTAAATCCTTTAACGAGGATCTATTGGAGGGCAAGTCTGGTGCCAGCAGCCGCGG
TAACTCCAGCTCCAATAGCGTATATTAAAGTTGTTGCGGTTAAAAA-
GCTCGTAGTTGGTTCTGCGTGCCACGCTGTCGGTTCACCGCCTGTCGGTGCAACTGGCATGCC-
GTGGCATGTCCTGTCGGTGGCTGACAGGC-CTCGCG-GCGCGCCCTATG-------CGTTCA-CTCGTGT---
-AGGTAGTTGC--GCCG-CGTCGTCC-TTGTAGG-CCGGCT-CAATCCTACCGCGGTGCT-
CTTTACCGAGTGTCGAGGTAGGCCGACATT-
GTTTACTTTGAACAAATTAGAGTGCTTCAAGCAGGCTCTGAAAGC-
AGCCTGAATAGTGGTGCATGGAATAATGGAACAGGACCTTGGTTCTATTTTGTT-GGTTTTCGGA-
ATCCAAGGTAATGATCAATAAGGA-CAGGCGGGGGCATTCGTATTGCGACGTTAGA-----------------
-GGTGAAATTCTTGG-
ATCGTCGCAAGACGCACTAGAGCGAAAGCATTTGCCAAGTATGTCTTAATTGATCAAGAACGAAAGTTAGAGG
TTCGAAGGCGATCAGATACCGCCCTAGTTCTAACCATAAACGATGCCAGCCAGCGATCCGCCGATGTTCCTCC
GATGACTCGGCGGGGAGCTT-
TCCCCGGGAAACCAAAGCTTTTGGGTTCCGGGGGAAGTATGGTTGCAAAGCTGAAACTTAAAGGAATTGACGG
AAGGGCACCACCAGGGGTGGAGCCTGCGGCTTAATTTGACTCAACACGCGAAAGCTCACCAGGCCCGGACATT
GGAA-GGATTGACAGAT-CGATAGCTCTTT-CTCGATT-CAGTGGGT-
AGTGGTGCATGGCCGTTCTTAGTTGGTGGATTGATTTGTCTGGTTAATTCCGATAACGAACGAGACTCTGACC
TATTAACTAGGCGCTC-CCGGTATAC-ATA-CCTACCGGCGAGAT-
TTATTCTTCTTAAGGGGACAGGCGGCTCTCAGCCGCACGAGATTGAGCAATAACAGGTCTGTAATGCCCTTAG
ATGTTCTGGGCCGCACGCGCGCTACACTGAAGGAATCAGCGTGTGC--
TCCCCTGACCGAAAGGTCCGGGCAACCCGCTGAACCTCCTTCGTGCTAGGAATTGGGGCTTGCAATTGTTCCC
CATGAACGAGGAATCCCTAGTAAGCGCGAGTCATAAGCTCGCGTTGATTAGGTCCCTGCCCTTTGTACACACC
GCCCGTCGCTACTACCGATTGAATGATTTAGTGAGGTCTTCGGACC-GGCGGTA--AC-CGGC-ACC--GGC-
----TCGCCCGG---TATCGGT-TTCGCTGGAAA-
GATGACCGAACTTGATCATTTAGAGGAAGTAAAAGTCGTAACAAGG-TTTCCAGTCA--TTGGGTGC---
AAA-CCTAAAGGCGCAATGAAAGT-GTCAG-------------TGGGGGATCCTA-------------
CGGGACC-----GCCTCCCGCTGGGAGGATGGGCTGCC----GTGCAGACACC--------TGC--
CGGCGCCCCGCACTCCCGGGGCGTCTC--GTTCTC-TTAACG-AG----
TGGAGACGCACCATGAGCGCACACGCTGGTACCCGAAAGATGGTGAACTATGCCTGGCCAGGACGAAGCCAGG
GGAAACCCTGGTGGAGGTCC--GAAGCG-ATTCTG--ACGTGCAAATCG-

ATCGTCTGAGCTGGGTATAGGGGCGAAAGACCAATCGAACCATCTTG---TTTATTT--
ATTTTAGGTCAGGCCTGCTCAATGATTAAT----
TTTTTAATTAAATGGCCGCAGTATCTTGACTGTGCAAAGGTAGCATAATCATTTGTCTTTTAATTGTAGGCTT
GTATGAATGGCTCGATGAGGTATAAACTTTCTTTATATTAAATGT------
TGAATTTAATTTTTAAGTTAAAAGGCTTAAATGTTT-TTAGAGGACGAGAAGACCCTATAGAG--TTTTACT-
--------CTACA-ATCAT-TATTCAATT-TTTGGATAATTG-----TAGTTGAATAATGGT--
TTAGGAGTTTTGTTGGGGCGACAGGGAAA-TTTTATTAACTTTCTTTATG--TATTAT-
TTATTAATTAGTATA-----TTAAAGATCCTGGA---TT---AGGATA--
ATAAGATTAAATTACCTTAGGGATAAC
Tritomegas_bicolor
ATGCAAGTCTCAGTACGAGCTTACT-
TAAAGTGAAACCGCAAAAGGCTCAGTAAACCGGTCATAGTTTACCGGA-TATATG-CCACTC-
ACGTGGATAACTGTGGTAATTCTAGAGCTAATACATGCAACT-AAGTCCCGACCAGCG---
ATGGAAAGGACGCTTTTATTGGATCAAAACCATTCGCCCGGTTCGCCC-GGGC------
GTCTGCTTGATGACTCT-AGATAAC-ACCACA-GATCGCACGGTCATTGTACCGGC-
GACGTATCCGTCAAATGTCTGACTTATCAGCTGTCGATGGTAGACTCTGTGTCTACCATGGCGCTCACGGGTA
ACGGGGAATCAGGGTTCGATTCCGGAGAGGGAGCCTGAGAAACGGCTACCACATCCAAGGAAGGCAGCAGGCA
CGCAAATTACCCACTCCCGGCACGGGGAGGTAGTGACAAAAAATAACGATACGGGACTCATCCGAGGCCCCGT
AATCGGAATGAGAACACCCTAAATCCTTTAACGAGGATCTATTGGAGGGCAAGTCTGGTGCCAGCAGCCGCGG
TAATTCCAGCTCCAATAGCGTATATTAAAGTTGTTGCGGTTAAAAA-
GCTCGTAGTTGGTTCTGCGTGCCACGCTGTCGGTTCACCGCCTGTCGGTGCAACTGGCATGCC-
GTGGCATGTCCTGTCGGTGGCCTACTGGT-TTCGCG-GCGCACCCTATG-------CGTT-CACTCGTGT---
-AGGTCGTTGC--GCTGC-GTTGTCC-TCGTAGG-CCGGCT-CAATCCTACCGCGGTGCT-
CTTTACCGAGTGTCGAGGTAGGCCGACACT-
GTTTACTTTGAACAAATTAGAGTGCTTCAAGCAGGCTCTGAAAGC-
AGCCTGAATAGTGGTGCATGGAATAATGGAACAGGACCTTGGTTCTATTTTGTT-GGTTTTCTGA-
ATCCAAGGTAATGATCAATAAGGA-CAGGCGGGGGCATTCGTATTGCGACGTTAGA-----------------
-GGTGAAATTCTTGG-
ATCGTCGCAAGACGCACTAGAGCGAAAGCATTTGCCAAGTATGTCTTAATTGATCAAGAACGAAAGTTAGAGG
TTCGAAGGCGATCAGATACCGCCCTAGTTCTAACCATAAACGATGCCAGCCAGCGATCCGCCGATGTTCCTCC
GATGACTCGGCGGGGAGCTT-
CCCCCGGGAAACCAAAGCTTTTGGGTTCCGGGGGAAGTATGGTTGCAAAGCTGAAACTTAAAGGAATTGACGG
AAGGGCACCACCAGGAGTGGAGCCTGCGGCTTAATTTGACTCAACACGGGAAACCTCACCAGGCCCGGACATT
GGAA-GGATTGACAGAT-CGATAGCTCTTTTCTCGATT-CAGTGGGT-
AGTGGTGCATGGCCGTTCTTAGTTGGTGGACTGATTTGTCTGGATAATTCCGATAACGAACGAGACTCTGACC
TATTAACTAGGCGCTC-CCGGTATAC-ATA-CCTACCGGCGAGAC-
TTATTCTTCTTAAAGGGACAGGCGGCTCTTAGCCGCACGAGATTGAGCAATAACAGGTCTGTGATGCCCTTAG
ATGTTCTGGGCCGCACGCGCGCTACACTGAAGGAATCAGCGTGTTC--
TCCCCTGTCCGAAAGGACCGGGCAACCCGCTGAACCTCCTTCGTGCTAGGAATTGGGGCTTGCAATTGTTCCC
CATGAACGAGGAATTCCCAGTAAGCGCGAGTCATAAGCTCGCGTTGATTACGTCCCTGCCCTTTGTACACACC
GCCCGTCGCTACTACCGATTGAATGATTTAGTGAGGTCTTCGGACC-GGCGGTA--AC-AGGC-ATA--
GGCT-----CGCCCTT---TATCTGT-TCCGCTGGAAA-
GATGACCGAACTTGATCATTTAGAGGAAGTAAAAGTCGTAACAAGG-TTTCCAGTCA--TTGGGTGT---
AAAACCTAAAGGCGTAATGAAAGT-GTC-------------AGGGGGTCCTCCT-------------
ACGGGAGG----ATCTCCCCGCTGGGAAGATGGGCCACC----GTACAATTATT--------TGT--
CGGAGCCCCGCACTCCCGGGGCGTCTC--GTTCTC-ATTGCG-AG----
TAGAGACGCACCAGGAGCGCACACGCTGGTACCCGAAAGATGGTGAACTATGCCTGGCCAGGACGAAGCCAGG
GGAAACCCTGGTGGAGGTCC--GAAGCG-ATTCTG--ACGTGCAAATCG-
ATCGTCTGAGCTGGGTATAGGGGCGAAAGACCAATCGAACCATCTTGA-ATTTTTG---
ATTTTAAGTCAAGCCTGCTCAATGATT----A------
TTATTAAATAGCCGCAGTATTTTGACTGTGCAAAGGTAGCATAATCATTTGTCTATTAATTGTAGGCTGGTAT
GAATGGTTAGATGAAATACTAGCTTTCTTTATTTTATGTTTTGA---
TGAATTTAATATTTGAGTTAAAAAGCTTAAATGTTT-TTGTAGGACGAGAAGACCCTATAGAA--
TTTTACTTAATT-GAT--------TATTTAATTAGATTTTTTAT-AT--TT----AT-TA-AATTTTTAGT--
--TGAGTTTTGTTGGGGTGACAGGGAAA-ATTCAGTAACTTTCTTTATTAAACA--T-
TTATTAATTGGTATAT-----TAAATATCCCT-----TTTTAGGGAA---
ATATGATTAAATTACCTTAGGGATAAC
Tritomegas_sexmaculatus

ATGCAAGTCTCAGTACGAGCTTACT-
TAAAGTGAAACCGCAAAAGGCTCAGTAAACCGGTCATAGTTTACCGGA-TATATG-CCACTC-
ACGTGGATAACTGTGGTAATTCTAGAGCTAATACATGCAACT-AAGTCCCGACCAGCG---
ATGGAAAGGACGCTTTTATTGGATCAAAACCATTCGCCCGGTTCGCCC-GGGC------
GTCTGCTTGATGACTCT-AGATAAC-ACCACA-GATCGCACGGTCATTGTACCGGC-
GACGTATCCGTCAAATGTCTGACTTATCAGCTGTCGATGGTAGACTCTGTGTCTACCATGGCGCTCACGGGTA
ACGGGGAATCAGGGTTCGATTCCGGAGAGGGAGCCTGAGAAACGGCTACCACATCCAAGGAAGGCAGCAGGCA
CGCAAATTACCCACTCCCGGCACGGGGAGGTAGTGACAAAAAATAACGATACGGGACTCATCCGAGGCCCCGT
AATCGGAATGAGAACACCCTAAATCCTTTAACGAGGATCTATTGGAGGGCAAGTCTGGTGCCAGCAGCCGCGG
TAATTCCAGCTCCAATAGCGTATATTAAAGTTGTTGCGGTTAAAAA-
GCTCGTAGTTGGTTCTGCGTGCCACGCTGTCGGTTCACCGCCTGTCGGTGCAACTGGCATGCC-
GTGGCATGTCCTGTCGGTGGCCTACTGGT-TTCGCG-GCGCACCCTATG-------CGTT-CACTCGTGT---
-AGGTCGTTGC--GCTGC-GTTGTCC-TCGTAGG-CCGGCT-CAATCCTACCGCGGTGCT-
CTTTACCGAGTGTCGAGGTAGGCCGACACT-
GTTTACTTTGAACAAATTAGAGTGCTTCAAGCAGGCTCTGAAAGC-
AGCCTGAATAGTGGTGCATGGAATAATGGAACAGGACCTTGGTTCTATTTTGTT-GGTTTTCTGA-
ATCCAAGGTAATGATCAATAAGGA-CAGGCGGGGGCATTCGTATTGCGACGTTAGA-----------------
-GGTGAAATTCTTGG-
ATCGTCGCAAGACGCACTAGAGCGAAAGCATTTGCCAAGTATGTCTTAATTGATCAAGAACGAAAGTTAGAGG
TTCGAAGGCGATCAGATACCGCCCTAGTTCTAACCATAAACGATGCCAGCCAGCGATCCGCCGATGTTCCTCC
GATGACTCGGCGGGGAGCTT-
CCCCCGGGAAACCAAAGCTTTTGGGTTCCGGGGGAAGTATGGTTGCAAAGCTGAAACTTAAAGGAATTGACGG
AAGGGCACCACCAGGAGTGGAGCCTGCGGCTTAATTTGACTCAACACGGGAAACCTCACCAGGCCCGGACATT
GGAA-GGATTGACAGAT-CGATAGCTCTTTTCTCGATT-CAGTGGGT-
AGTGGTGCATGGCCGTTCTTAGTTGGTGGACTGATTTGTCTGGATAATTCCGATAACGAACGAGACTCTGACC
TATTAACTAGGCGCTC-CCGGTATAC-ATA-CCTACCGGCGAGAC-
TTATTCTTCTTAAAGGGACAGGCGGCTCTTAGCCGCACGAGATTGAGCAATAACAGGTCTGTGATGCCCTTAG
ATGTTCTGGGCCGCACGCGCGCTACACTGAAGGAATCAGCGTGTTC--
TCCCCTGTCCGAAAGGACCGGGCAACCCGCTGAACCTCCTTCGTGCTAGGAATTGGGGCTTGCAATTGTTCCC
CATGAACGAGGAATTCCCAGTAAGCGCGAGTCATAAGCTCGCGTTGATTACGTCCCTGCCCTTTGTACACACC
GCCCGTCGCTACTACCGATTGAATGATTTAGTGAGGTCTTCGGACC-GGCGGTA--AC-AGGC-ATA--
GGCT-----CGCCCTT---TATCTGT-TCCGCTGGAAA-
GATGACCGAACTTGATCATTTAGAGGAAGTAAAAGTCGTAACAAGG-TTTCCAGTCA--TTGGGTGT---
AAAACCTAAAGGCGTAATGAAAGT-GTC-------------AGGGGGTCCTCCT-------------
CCGGGAGG----ATCTCCCCGCTGGGAAGATGGGCCACC----GTACAATTATT--------TGT--
CGGAGCCCCGCACTCCCGGGGCGTCTC--GTTCTC-ATTGCG-AG----
TAGAGACGCACCAGGAGCGCACACGCTGGTACCCGAAAGATGGTGAACTATGCCTGGCCAGGACGAAGCCAGG
GGAAACCCTGGTGGAGGTCC--GAAGCG-ATTCTG--ACGTGCAAATCG-
ATCGTCTGAGCTGGGTATAGGGGCGAAAGACCAATCGAACCATCTTGTTATTTTTT---
ATTTTAAGTCAAGCCTGCTCAATGATTC--TA------
TTATTAAATAGCCGCAGTATTTTGACTGTGCAAAGGTAGCATAATCATTTGTCTATTAATTGTAGGCTGGTAT
GAATGGTTAGATGAAATATTGACTTTCTTTATTTTAT-TATTTA---
TGAATTTAATGTTTGAGTTAAAAAGCTTAAATATTT-ATGTAGGACGAGAAGACCCTATAGAA--
TTTTACTTAATTTAAT--------TATTTGGTTAAATTTTTTAT-AT--TT----AT-TA-AATTTTTATT--
--TAAGTTTTGTTGGGGTGACAGGGAAA-ATTTAATAACTTTCTTTATTAAGTT--T-
TTATTGATTTATATAT-----TAAATATCCCT-----TTTTAGGGAA---
ATATGATTAAATTACCTTAGGGATAAC
Urochela_luteovaria
ATGCAAGTCTCAGTACGAGCTGAAT-
TAAAGTGAAACCGCAAAAGGCTCAGTAAACCGGTCATAGTTTACCGGA-TATATATCCACTC-
ACGTGGATAACTGTGGTAATTCTAGAGCTAATACATGCAACC-AAATCCCGACCAGCG---
ATGGAAAGGACGCTTTTATTGGATCAAAACCATTCGCCCGGCTCGCCC-GGGC------
GTCTGTCTGATGACTCT-ATATAAC-ACCGCA-GATCGCACGGTCTTAGTACCGGC-
GACGTATCCGTCAAATGTCTGACTTATCAGCTGTCGATGGTAGACTCTGTGTCTACCATGGCGCTCACGGGTA
ACGGGGAATCAGGGTTCGATTCCGGAGAGGGAGCCTGAGAAACGGCTACCACATCCAAGGAAGGCAGCAGGCA
CGCAAATTACCCACTCCCGGCACGGGGAGGTAGTGACAAAAAATAACGATACGGGACTCATCCGAGGCCCCGT
AATCGGAATGAGAACACCCTAAATCCTTTAACGAGGATCCATTGGAGGGCAAGTCTGGTGCCAGCAGCCGCGG
TAATTCCAGCTCCAATAGCGTATATTAAAGTTGTTGCGGTTAAAAA-

GCTCGTAGTTGGTTCTGCGTGCCACGCTGTCGGTTCACCGCCTGTCGGTGCAACTGGCATGCC-
GTGGCATGTCCTGTCGGTGGCCGACAGGC-TTCGCG-GCGCGCCCTACG-------CGCT-CACGCGTGT---
-AGGTAGTTGC--GCCGC-GTCGTCC-TTGTAGG-CCGTTT-CAATCCTACCGCGGTGCT-
CTTAAACGAGTGTCGAGGTAGGCCGACACT-
GTTTACTTTGAACAAATTAGAGTGCTTCAAGCAGGCTTTTAATGC-
AGCCTGAATAGTGGTGCATGGAATAATGGAACAGGACCTTGGTTCTATTTTGTT-GGTTTTCGGA-
ATCCAAGGTAATGATCAATAAGGA-CAGGCGGGGGCATTCGTATTGCGACGTTAGA-----------------
-GGTGAAATTCTTGG-
ATCGTCGCAAGACGCACTAGAGCGAAAGCATTTGCCAAGTATGTCTTAATTGATCAAGAACGAAAGTTAGAGG
TTCGAAGGCGATCAGATACCGCCCTAGTTCTAACCATAAACGATGCCAGCCAGCGATCCGCCGATGTTCCTCC
GATGACTCGGCGGGGAGCTT-
TCCCCGGGAAACCAAAGCTTTTGGGTTCCGGGGGAAGTATGGTTGCAAAGCTGAAACTTAAAGGAATTGACGG
AAGGGCACCACCAGGAGTGGAGCCTGCGGCTTAATTTGACTCAACACGGGAAACCTCACCAGGCCCGGACATT
GGAA-GGATTGACAGAT-CGATAGCTCTTT-CTCGATT-CAGTGGGT-
AGTGGTGCATGGCCGTTCTTAGTTGGTGGACTGATTTGTCTGGTTAATTCCGATAACGAACGAGACTCTGACC
TATTAACTAGGCGTT--CCGGTATAT-ATATACTACCGGCGAGAT-
TTATTCTTCTTAAGGGGACAGGCGGCTCTTAGCCGCACGAGATTGAGCAATAACAGGTCTGTGATGCCCTTAG
ATGTTCTGGGCCGCACGCGCGCTACACTGAAGGAATCAGCGTGTTC--
TCCCCTGTCCGAAAGGTCCGGGCAACCCGCTGAACCTCCTTCGTGCTAGGAATTGGGGCTTGCAATTGTTCCC
CATGAACGAGGAATTCCCAGTAAGCGCGAGTCATAAGCTCGCGTTGATTACGTCCCTGCCCTTTGTACACACC
GCCCGTCGCTACTACCGATTGAATGATTTAGTGAGGTCTTCGGACC-GGCGCTA--AC-CGGC-ACC--
GGCT-----CGTTCGG---TATCGGT-TTCGCTGGAAA-
GATGACCGAACTTGATCATTTAGAGGAAGTAAAAGTCGTAACAAGG-TTTCCAGTCA--TTGGGTGA---
AAAACCTAAAGGCGCAATGAAAGT-ATC-------------AGGGGGGCCTTCT-------------
ACGGAAGT-----GCCCCCTGTAGGGAGGATGGGCCACCG---GT----TTTTT--------AAC--
CGGAGCCCCGCACTCCCGGGGCGTCTC--GTTCTC-ATTGCG-AG----
TTGAGACGCACCAGGAGCGCACACGCTGGTACCCGAAAGATGGTGAACTATGCCTGGCCAGGACGAAGCCAGG
GGAAACCCTGGTGGAGGTCC--GAAGCG-ATTCTG--ACGTGCAAATCG-
ATCGTCTGAGCTGGGTATAGGGGCGAAAGACCAATCGAACCATCATGAATTAAATAT--A----
AGGTCAGGCCTGCTCGGTGATT------------
TATTTAACAGCTGCAGTATATTGACTGTGCAAAGGTAGCATAATCATTTGTCTTTTAATTGGGGACTAGTATG
AACGGTTTGATGAAATATTAACTTTCTTTT---TATATTAATTT--
TGAAATTAATTTTTGTGTTAAAAAGCATAAATTCAA-TTATAGGACGAGAAGACCCTATAGAA--
TTTTGAATATCCATAT----------TTT-TATAGTAGATT--T-ATTATC----AAGTA--
TTTAAAAAATAGTATATTTTTGTTGGGGTGACAGAGGAA-TATATCAAACTTCCATTTAAATAATT---
TTATAAATTTATATAT-----TATTGATCCTA------TTTTTAGAAT--
ATAAGATTAAATTACCTTAGGGATAAC
Urostylis_chinai
ATGCAAGTCTCAGTACGAGCTGAAT-
TAAAGTGAAACCGCAAAAGGCTCAGTAAACCGGTCATAGTTTACCGGA-TATATATCCACAC-
ACGTGGATAACTGTGGTAATTCTAGAGCTAATACATGCAACC-AAATCCTGACCAGCG---
ATGGAAAGGACGCTTTTATTAGATCAAAACCATTCGCCCGGCTCGCCC-GGGC------
GTCTGCTTGATGACTCT-ATATAAC-ACCGCA-GATCGCACGGTCTTAGTACCGGC-
GACGTATCCGTCAAATGTCTGACTTATCAGCTGTCGATGGTAGACTCTGTGTCTACCATGGCGCTCACGGGTA
ACGGGGAATCAGGGTTCGATTCCGGAGAGGGAGCCTGAGAAACGGCTACCACATCCAAGGAAGGCAGCAGGCA
CGCAAATTACCCACTCCCGGCACGGGGAGGTAGTGACAAAAAATAACGATACGGGACTCATCCGAGGCCCCGT
AATCGGAATGAGAACACCCTAAATCCTTTAACGAGGATCCATTGGAGGGCAAGTCTGGTGCCAGCAGCCGCGG
TAATTCCAGCTCCAATAGCGTATATTAAAGTTGTTGCGGTTAAAAA-
GCTCGTAGTTGGTTCTGCGTGCCACGCTGTCGGTTCACCGCCTGTCGGTGCAACTGGCATGTT-
GTGGCATGTCCTGTCGGTGGTCGACAGGC-TTCGCG-GGGCGCCCTACT-------CGCTTCGCGCTTGT---
-AGGTAGTTGC--TCCGC-GTCGTCC-TTGTAGG-CCGGCT-CAATCCTATCGCGGTGCT-
CTTAAACGAGTGTCGAGGTAGGCCGACACT-
TTTTACTTTGAACAAATTAGAGTGCTTCAAGCAGGCTTTTAAAGC-
AGCCTGAATAGTTGTGCATGGAATAATGGAACAGGACCTTGGTTCTATTTTGTT-GGTTTTCGGA-
ATCCAAGGTAATGATCAATAAGGA-CAGGCGGGGGCATTCGTATTGCGACGTTAGA-----------------
-GGTGAAATTCTTGG-
ATCGTCGCAAGACGCACTAGAGCGAAAGCATTTGCCAAGTATGTCTTAATTGATCAAGAACGAAAGTTAGAGG
TTCGAAGGCGATCAGATACCGCCCTAGTTCTAACCATAAACGATGCCAGCCAGCGATCCGCCGATGTTCCTCC

```
GATGACTCGGCGGGGAGCTT-
TCCCCGGGAAACCAAAGCTTTTGGGTTCCGGGGGAAGTATGGTTGCAAAGCTGAAACTTAAAGGAATTGACGG
AAGGGCACCACCAGGAGTGGAGCCTGCGGCTTAATTTGACTCAACACGGGAAACCTCACCAGGCCCGGACATT
GGAA-GGATTGACAGAT-CGATAGCTCTTT-CTCGATT-CAGTGGGT-
AGTGGTGCATGGCCGTTCTTAGTTGGTGGACTGATTTGTCTGGTTAATTCCGATAACGAACGAGACTCTAACC
TATTAACTAGGCGTT--CCGGTAT-T-ATTCGCTACCGGCGAGAT-
TTATTCTTCTTAAGGGGACAGGCGGCTCTTAGCCGCACGAGATTGAGCAATAACAGGTCTGTGATGCCCTTAG
ATGTTCTGGGCCGCACGCGCGCTACACTGAAGGAATCAGCGTGTTC--
TCCCCTGTCCGAAAGGTCCGGGCAACCCGCTGAACCTCCTTCGTGCTAGGAATTGGGGCTTGCAATTGTTCCC
CATGAACGAGGAATTCCCAGTAAGCGCGAGTCATAAGCTCGCGTTGATTACGTCCCTGCCCTTTGTACACACC
GCCCGTCGCTACTACCGATTGAATGATTTAGTGAGGTCTTCGGACC-GGCGTTA--AC-CGGC-ACC--
GGCT-----CGTTCGG---TATCGGT-TTCGCTGGAAA-
GATGACCGAACTTGATCATTTAGAGGAAGTAAAAGTCGTAACAAGG-TTTCCAGTCA--TTGGGTGC---
AAAACCTAAAGGCGCAATGAAAGT-ATC-------------AGGGGGGCCTTCC-------------
ACGGAAGT-----GCTCCTCGTTGGGAAGATGGGCCACCG---GC----TTTTT--------AGT--
CGGAGCCCCGCACTCCCGGGGCGTCTC--GTTCTC-ATTGCG-AG----
TTGAGACGCACCAGGAGCGCACACGCTGGTACCCGAAAGATGGTGAACTATGCCTGGCCAGGACGAAGCCAGG
GGAAACCCTGGTGGAGGTCC--GAAGCG-ATTCTG--ACGTGCAAATCG-
ATCGTCTGAGCTGGGTATAGGGGCGAAAGACCAATCGAACCATCGTG--
ATACATATTTATATTAGGTCGGGCCTGCTCAGTGATT------------
TATTTAACAGCTGCAGTAATTTGACTGTGCAAAGGTAGCATAATCATTTGTCTTTTAATTGAAGGCTGGTATG
AATGGTTTGATGAAATATTAACTTTCTTTT---TATTTAATTTT--
TGAATTTAATTTTTATGTGAAAAAGCATAGATTATG-TTGTAGGACGAGAAGACCCTATAGAA--
TTTTAATTTAGTTTAT----------TTTATATTATTTATGGAT-ATTAT-----AAATC--
TTTAATATTAATTATATTTTTGTTGGGGTGACAGGAAAA-TTTATTTAACTTTTTCTTTTTAATTT---
TTATTCTTCCATATAT-----TAATTATCCTA------ATTATAGATT--
ACAAGATTAAATTACCTTAGGGATAAC
;
end;
```
