# Supplementary material for: Sehiridae stat. nov., the Highly-Supported Monophylum Within the ‘Cydnoid’ Complex of Pentatomoid Families (Hemiptera: Heteroptera: Pentatomoidea)
Source: Insects. 2026 Aug 12;17(8):835. doi: 10.3390/insects17080835 (PMC13512964; doi:10.3390/insects17080835)
Supplement: Supplementary file 1 [file insects-17-00835-s001.zip › Table S1.pdf]

Table S1. List of Cydnidae specimens used in the phylogenetic analyses with GenBank accession numbers.

**Family/subfamily**	**Species**	**16S rDNA GenBank accession number**	**18S rDNA GenBank accession number**	**28S rDNA GenBank accession number**
**Cydnidae: Amaurocorinae**	*Amaurocoris curtus* (Brullé, 1838)	PP357100 [1]	PZ621194 [6]	PZ612346 [6]
**Cydnidae: Amnestinae**	*Amnestus ficus* Mayorga & Cervantes, 2001	PP357101 [1]	PZ621195 [6]	PZ612347 [6]
	Amnestus subferrugineus (Westwood, 1837)	PZ639145 [6]	PZ621196 [6]	PZ612348 [6]
	Amnestus zacki Mayorga & Cervantes, 2009	PP357103 [1]	PZ621197 [6]	PZ612349 [6]
	Lattinestus barrerai Mayorga & Brailovsky, 2012	PP357123 [1]	PZ621213 [6]	PZ612365 [6]
**Cydnidae: Cephalocteinae**	*Scaptocoris divergens* Froeschner, 1960	PZ639146 [6]	PZ621227 [6]	PZ612379 [6]
	Stibaropus indonesicus J.A. Lis, 1991	PP357145 [1]	PZ621228 [6]	PZ612380 [6]
**Cydnidae: Cydninae**	*Adrisa birmana* J.A. Lis, 1992	PP357095 [1]	PZ621190 [6]	PZ612342 [6]
	Adrisa magna (Uhler, 1861)	PP357096 [1]	KY911199 [4]	KY886254 [4]
	Adrisa romani J.A. Lis, 1994	PP357097 [1]	PZ621191 [6]	PZ612343 [6]
	Aethus pseudindicus J.A. Lis, 1993	PP357098 [1]	PZ621192 [6]	PZ612344 [6]
	Alonips obsoletus Signoret, 1881	PP357099 [1]	PZ621193 [6]	PZ612345 [6]
	Byrsinus pseudosyriacus (Linnavuori, 1977)	PP357105 [1]	PZ621200 [6]	PZ612351 [6]
	Byrsinus varians (Fabricius, 1803)	PP357106 [1]	PZ621199 [6]	PZ612352 [6]
	Chilocoris assmuthi Breddin, 1904	-	-	KY886256 [4]
	Chilocoris confusus Horváth, 1919	PP357109 [1]	KY911201 [4]	-
	Chilocoris laevicollis Horváth, 1919	PZ639147 [6]	PZ621202 [6]	PZ612354 [6]
	Chilocoris neozealandicus Lariviére & Froeschner, 1994	PP357110 [1]	-	-
	Chilocoris piceus Signoret, 1884	-	PZ621203 [6]	PZ612355 [6]
	Cydnus aterrimus (Forster, 1771)	PP357113 [1]	KY911202 [4]	KY886257 [4]
	Cyrtomenus emarginatus Stål, 1862	PP357114 [1]	PZ621204 [6]	PZ612356 [6]
	Fromundus biimpressus (Horváth, 1919)	PZ639148 [6]	PZ621207 [6]	PZ612358 [6]
	Fromundus difficilis (Stål, 1854)	PP357115 [1]	PZ621206 [6]	PZ612359 [6]
	Fromundus pygmaeus (Dallas, 1851)	PP357116 [1]	KJ535871 [5]	KJ535871 [5]
	Geotomus convexus Hsiao, 1977	PP357118 [1]	KY911203 [4]	KY886258 [4]
	Katakadia caliginosa (Walker, 1867)	PP357119 [1]	PZ621210 [6]	PZ612362 [6]
	Lactistes obesipes Signoret, 1879	PP357120 [1]	PZ621211 [6]	PZ612363 [6]

	Lactistes vericulatus Schiødte, 1848	PP357121 [1]	PZ621212 [6]	PZ612364 [6]
	Macroscytus annulipoides J.A. Lis, 1999	PP357125 [1]	PZ621216 [6]	PZ612368 [6]
	Macroscytus badius (Walker, 1867)	PP357126 [1]	PZ621217 [6]	PZ612369 [6]
	Macroscytus brunneus (Fabricius, 1803)	JQ029138 [2]	KY911204 [4]	KY886259 [4]
	Macroscytus fraterculus Horváth, 1919	PP357127 [1]	KJ535876 [5]	KJ535876 [5]
	Macroscytus japonensis Scott, 1874	PP357128 [1]	PZ621218 [6]	PZ612370 [6]
	Macroscytus minimus J.A. Lis, 1999	PP357129 [1]	PZ621219 [6]	PZ612371 [6]
	Microporus nigrita (Fabricius, 1794)	JQ029137 [2]	KY911205 [4]	KY886260 [4]
	Microporus pallidipennis (Reuter, 1883)	PP357130 [1]	PZ621220 [6]	PZ612372 [6]
	Pangaeus bilineatus (Say, 1825)	PP357137 [1]	KY911207 [4]	KY886262 [4]
	Pangaeus rugiceps Horváth, 1919	PP357138 [1]	PZ621222 [6]	PZ612374 [6]
	Parachilocoris minutus (Distant, 1901)	PP357139 [1]	PZ621223 [6]	PZ612375 [6]
	Pseudoscoparipes fraterculus J.A. Lis, 1994	PP357141 [1]	PZ621224 [6]	PZ612376 [6]
	Pseudoscoparipes kinabalensis J.A. Lis, 1994	PP357142 [1]	PZ621225 [6]	PZ612377 [6]
	Pseudoscoparipes vollenhoveni (Signoret, 1881)	PP357143 [1]	PZ621226 [6]	PZ612378 [6]
	Rhytidoporus indentatus Uhler, 1877	PP357144 [1]	KY911208 [4]	KY886263 [4]
**Cydnidae: Garsauriinae**	*Blaena setosa* Walker, 1868	PP357104 [1]	PZ621198 [6]	PZ612350 [6]
	Garsauria aradoides Walker, 1868	PP357117 [1]	PZ621208 [6]	PZ612360 [6]
**Cydnidae: Sehirinae**	*Adomerus biguttatus* (Linnaeus, 1758)	PP357091 [1]	KY911198 [4]	KY886253 [4]
	Adomerus variegatus (Signoret, 1884)	PP357094 [1]	PZ621189 [6]	PZ612341 [6]
	Canthophorus impressus (Horváth, 1881)	PP357107 [1]	PZ621201 [6]	PZ612353 [6]
	Canthophorus niveimarginatus Scott, 1874	PP357108 [1]	KY911200 [4]	KY886255 [4]
	Legnotus limbosus (Geoffroy, 1785)	PP357124 [1]	PZ621214 [6]	PZ612366 [6]
	Ochetostethomorpha secunda J.A. Lis & B. Lis, 2014	PP357132 [1]	KY911206 [4]	KY886261 [4]
	Ochetostethus opacus (Scholtz, 1847)	PP357136 [1]	PZ621221 [6]	PZ612373 [6]
	Sehirus luctuosus Mulsant et Rey, 1866	JQ234973 [3]	KY911209 [4]	KY886264 [4]
	Tritomegas bicolor (Linnaeus, 1758)	PP357148 [1]	PZ621229 [6]	PZ612381 [6]
	Tritomegas sexmaculatus (Rambur, 1839)	PP357149 [1]	KY911210 [4]	KY886265 [4]

References

[1] Lis, J.A.; Domagała, P.J.; Lis, B. New Molecular Phylogenetic Evidence Confirms Independent Origin of Coxal Combs in the Families of the 'Cydnoid' Complex (Hemiptera: Heteroptera: Pentatomoidea). *Insects* **2024**, *15*, 792. https://doi.org/10.3390/insects15100792

[2] Lis, J.A.; Lis, P.; Ziaja, D.J.; Kocorek, A. Systematic position of Dinidoridae within the superfamily Pentatomoidea (Hemiptera: Heteroptera) revealed by the Bayesian phylogenetic analysis of the mitochondrial 12S and 16S rDNA sequences. *Zootaxa* **2012**, *3423*, 61–68. https://doi.org/10.11646/zootaxa.3423.1.5
[3] Lis, J.A.; Lis, P.A.; Ziaja, D.J. Comparative studies on 12S and 16S mitochondrial rDNA sequences in pentatomomorphan bugs (Hemiptera: Heteroptera: Pentatomomorpha. *Nature J. (Opole)* **2011**, *44*, 73–91.
[4] Lis, J.A.; Ziaja, D.; Lis, B.; Gradowska, P.A. Non-monophyly of the "cydnoid" complex within Pentatomoidea (Hemiptera: Heteroptera) revealed by Bayesian phylogenetic analysis of nuclear rDNA sequences. *Arthropod. Syst. Phylogeny.* **2017**, *75*, 481–496.
[5] Wu, Y.Z.; Yu, S.S.; Wang, Y.H.; Wu, Y.H.; Li, X.R.; Men, X.Y.; Zhang, Y.W.; Rédei, D.; Xie, Q.; Bu, W.J. The evolutionary position of Lestoniidae revealed by molecular autapomorphies in the secondary structure of rRNA besides phylogenetic reconstruction (Insecta: Hemiptera: Heteroptera). *Zool. J. Linn. Soc.* **2016**, *177*, 750–763. https://doi.org/10.1111/zoj.12385
[6] present paper.
