# Supplementary material for: Sehiridae stat. nov., the Highly-Supported Monophylum Within the ‘Cydnoid’ Complex of Pentatomoid Families (Hemiptera: Heteroptera: Pentatomoidea)
Source: Insects. 2026 Aug 12;17(8):835. doi: 10.3390/insects17080835 (PMC13512964; doi:10.3390/insects17080835)
Supplement: Supplementary file 1 [file insects-17-00835-s001.zip › Table S2.pdf]

Table S2. List of Pentatomoidea specimens (other than Cydnidae) used in the phylogenetic analyses with GenBank accession numbers.

Family	Species	16S rDNA GenBank accession number	18S rDNA GenBank accession number	28S rDNA GenBank accession number
**Acanthosomatidae**	*Acanthosoma nigrodorsum* Hsiao & Liu, 1977	LC099172 [1]	KJ522637 [16]	KJ522637 [16]
	Elasmostethus interstinctus (Linnaeus, 1758)	JQ029152 [2]	KY911197 [17]	KY886252 [17]
	Elasmucha grisea (Linnaeus, 1758)	JQ029151 [2]	KJ559415 [18]	KJ559415 [18]
	Elasmucha laeviventris Liu, 1919	LC099199 [1]	KJ535865 [16]	KJ535865 [16]
	Lindbergicoris hochii (Yang, 1933)	LC099164 [1]	KJ535875 [16]	KJ535875 [16]
	Sastragala scutellata (Scott, 1874)	LC099181[1]		
	Sastragala sp.	-	KJ535882 [16]	KJ535882 [16]
**Dinidoridae**	*Cyclopelta obscura* (Lepeletier & Serville, 1828)	JQ029146 [2]	KJ522641 [16]	KJ522642 [16]
	Megymenum brevicorne (Fabricius, 1787)	JQ029141 [2]	-	KY886266 [17]
	Megymenum sp.	-	KJ535879 [16]	-
**Lestoniidae**	*Lestonia haustorifera* China, 1955	AY252664 [23]	KT188471 [16]	KT188472 [16]
**Parastrachiidae**	*Dismegistus sanguineus* (DeGeer, 1778)	EF641131 [3]	EF641203 [3]	EF641183 [3]
	Parastrachia japonensis (Scott, 1880)	JQ029136 [2]	EF641204 [3]	EF641184 [3]
**Pentatomidae**	*Dolycoris baccarum* (Linnaeus)	JQ029153 [2]	-	-
	Dolycoris penicillatus Horváth, 1904	-	KJ535863 [16]	KJ535863 [16]
	Erthesina fullo (Thunberg, 1783)	AY986801 [4]	KJ535866 [16]	KJ535866 [16]
	Eurydema maracandica Oshanin, 1871	NC_037042 [5]	KJ535867 [16]	KJ535867 [16]
	Gonopsis affinis (Uhler, 1860)	MG182695 [6]	-	-
	Gonopsis sp.	-	KJ535872 [16]	KJ535872 [16]
	Graphosoma italicum (O.F. Müller, 1766)	PP357151 [7]	KY911211 [17]	KY886267 [17]
	Picromerus lewisi Scott, 1874	MW355499 [8]	KJ535896 [16]	KJ535891 [16]
**Plataspidae**	*Coptosoma bifarium* Montandon, 1896	EU427334 [9]	KJ461259 [19]	KJ461239 [19]
	Coptosoma scutellatum (Geoffroy, 1785)	JQ029144 [2]	KY911212 [17]	KY886268 [17]
	Megacopta cribraria (Fabricius, 1798)	JF288758 [10]	AY627313 [20]	-
	Megacopta sp.	-	-	KJ535889 [16]
**Scutelleridae**	*Eurygaster maura* (Linnaeus, 1758)	JQ029150 [2]	PZ621205 [21]	PZ612357 [21]
	Odontoscelis fuliginosa (Linnaeus)	JQ029149 [2]	KJ535881 [16]	KJ535881 [16]

	Poecilocoris druraei (Linnaeus, 1771)	MW847246 [11]	KY495683 [22]	KY495716 [22]
**Tessaratomidae**	*Dalcantha alata* Breddin, 1904	-	KJ535862 [16]	KJ535862 [16]
	Dalcantha dilatata Amyot & Serville, 1843	JQ910981 [12]	-	-
	Eusthenes cupreus (Westwood, 1837)	JQ910983 [13]	KJ535869 [16]	KJ535869 [16]
**Thaumastellidae**	*Thaumastella elizabethae* Jacobs, 1989	EF641147 [3]	EF641221 [3]	EF641195 [3]
	Thaumastella namaquensis Schaefer & Wilcox, 1971	EF641148 [3]	EF641222 [3]	EF641196 [3]
**Thyreocoridae**	*Galgupha australis* McAtee & Malloch, 1933	PP357150 [7]	KY911213 [17]	KY886269 [17]
	Thyreocoris scarabaeoides (Linnaeus, 1758)	JQ029135 [2]	KY911214 [17]	KY886270 [17]
**Urostylididae**	*Urochela luteovaria* Distant, 1881	AB858380 [14]	KJ461205 [19]	KJ461306 [19]
	Urostylis chinai Maa, 1947	OQ325103 [15]	KJ535886 [16]	KJ535886 [16]

References

[1] Tsai, J.F.; Kudo, S.; Yoshizawa, K. Maternal Care in Acanthosomatinae (Insecta: Heteroptera: Acanthosomatidae) – Correlated Evolution with Morphological Change. *BMC Evol. Biol.* **2015**, *15*, 258. https://doi.org/10.1186/s12862-015-0537-4

[2] Lis, J.A.; Lis, P.; Ziaja, D.J.; Kocorek, A. Systematic position of Dinidoridae within the superfamily Pentatomoidea (Hemiptera: Heteroptera) revealed by the Bayesian phylogenetic analysis of the mitochondrial 12S and 16S rDNA sequences. *Zootaxa* **2012**, *3423*, 61–68. https://doi.org/10.11646/zootaxa.3423.1.5

[3] Grazia, J.; Schuh, R.T.; Wheeler, W.C. Phylogenetic Relationships of Family Groups in Pentatomoidea Based on Morphology and DNA Sequences (Insecta: Heteroptera). *Cladistics* **2008**, *24*, 932–976. https://doi.org/10.1111/j.1096-0031.2008.00224.x

[4] Li, H.-M. *Erthesina fullo* 16S Ribosomal RNA Gene, Partial Sequence; Mitochondrial. *GenBank, direct submission* **2005**, *Accession No. AY986801*. Available online: https://www.ncbi.nlm.nih.gov/nuccore/AY986801 (accessed on 11 January, 2026).

[5] Zhao, W.; Zhao, Q.; Li, M.; Wei, J.; Zhang, X.; Zhang, H. Characterization of the Complete Mitochondrial Genome and Phylogenetic Implications for *Eurydema maracandica* (Hemiptera: Pentatomidae). *Mitochondrial DNA B Resour.* **2017**, *2*, 550–551. https://doi.org/10.1080/23802359.2017.1365649

[6] Chen, C.; Wei, J.; Ji, W.; Zhao, Q. The First Complete Mitochondrial Genome from the Subfamily Phyllocephalinae (Heteroptera: Pentatomidae) and Its Phylogenetic Analysis. *Mitochondrial DNA B Resour.* **2017**, *2*, 938–939. https://doi.org/10.1080/23802359.2017.1413313

[7] Lis, J.A.; Domagała, P.J.; Lis, B. New Molecular Phylogenetic Evidence Confirms Independent Origin of Coxal Combs in the Families of the 'Cydnoid' Complex (Hemiptera: Heteroptera: Pentatomoidea). *Insects* **2024**, *15*, 792. https://doi.org/10.3390/insects15100792

[8] Mu, Y. *Picromerus lewisi* mitochondrion, complete genome. *GenBank, direct submission* **2020**, *Accession No. MW355499*. Available online: https://www.ncbi.nlm.nih.gov/nuccore/MW355499 (accessed on 11 January, 2026).

[9] Hua, J.; Li, M.; Dong, P.; Cui, Y.; Xie, Q.; Bu, W. Comparative and Phylogenomic Studies on the Mitochondrial Genomes of Pentatomomorpha (Insecta: Hemiptera: Heteroptera). *BMC Genomics* **2008**, *9*, 610. https://doi.org/10.1186/1471-2164-9-610
[10] Eaton, T.D.; Jenkins, T.M. *Megacopta cribraria* mitochondrion, complete genome. *GenBank, direct submission* **2012**, *Accession No. JF288758*. Available online: https://www.ncbi.nlm.nih.gov/nuccore/JF288758 (accessed on 11 January, 2026).
[11] Xu, S.; Wu, Y.; Liu, Y.; Zhao, P.; Chen, Z.; Song, F.; Li, H.; Cai, W. Comparative Mitogenomics and Phylogenetic Analyses of Pentatomoidea (Hemiptera: Heteroptera). *Genes* **2021**, *12*, 1306. https://doi.org/10.3390/genes12091306
[12] Li, H.; Leavengood, J.M., Jr.; Chapman, E.G.; Burkhardt, D.; Song, F.; Jiang, P.; Liu, J.; Zhou, X.; Cai, W. Mitochondrial Phylogenomics of Hemiptera Reveals Adaptive Innovations Driving the Diversification of True Bugs. *Proc. Biol. Sci.* **2017**, *284*, 20171223. https://doi.org/10.1098/rspb.2017.1223
[13] Song, W.; Li, H.; Song, F.; Liu, L.; Wang, P.; Xun, H.; Dai, X.; Chang, J.; Cai, W. The Complete Mitochondrial Genome of a Tessaratomid Bug, *Eusthenes cupreus* (Hemiptera: Heteroptera: Pentatomomorpha: Tessaratomidae). *Zootaxa* **2013**, *3620*, 260–272. https://doi.org/10.11646/zootaxa.3620.2.4
[14] Kaiwa, N.; Hosokawa, T.; Nikoh, N.; Tanahashi, M.; Moriyama, M.; Meng, X.Y.; Maeda, T.; Yamaguchi, K.; Shigenobu, S.; Ito, M.; Fukatsu, T. Symbiont-Supplemented Maternal Investment Underpinning Host's Ecological Adaptation. *Curr. Biol.* **2014**, *24*, 2465–2470. https://doi.org/10.1016/j.cub.2014.08.065
[15] Duan, Y.; Fu, S.; Ye, Z.; Bu, W. Phylogeny of Urostylididae (Heteroptera: Pentatomoidea) Reveals Rapid Radiation and Challenges Traditional Classification. *Zool. Scr.* **2023**, *52*, 264–278. https://doi.org/10.1111/zsc.12582
[16] Wu, Y.-Z.; Yu, S.-S.; Wang, Y.-H.; Wu, H.-Y.; Li, X.-R.; Men, X.-Y.; Zhang, Y.-W.; Rédei, D.; Xie, Q.; Bu, W.-J. The Evolutionary Position of Lestoniidae Revealed by Molecular Autapomorphies in the Secondary Structure of rRNA besides Phylogenetic Reconstruction (Insecta: Hemiptera: Heteroptera). *Zool. J. Linn. Soc.* **2016**, *177*, 750–763. https://doi.org/10.1111/zoj.12385
[17] Lis, J.A.; Ziaja, D.; Lis, B.; Gradowska, P.A. Non-monophyly of the "cydnoid" complex within Pentatomoidea (Hemiptera: Heteroptera) revealed by Bayesian phylogenetic analysis of nuclear rDNA sequences. *Arthropod. Syst. Phylogeny.* **2017**, *75*, 481–496.
[18] Yu, S.; Xie, Q. *Elasmucha grisea* 18S ribosomal RNA gene, partial sequence; internal transcribed spacer 1, 5.8S ribosomal RNA gene, and internal transcribed spacer 2, complete sequence; and 28S ribosomal RNA gene, partial sequence. *GenBank, direct submission* **2014**, *Accession No. KJ559415*. Available online: https://www.ncbi.nlm.nih.gov/nuccore/KJ559415 (accessed on 11 January, 2026).
[19] Wang, Y.-H.; Cui, Y.; Rédei, D.; Baňař, P.; Xie, Q.; Štys, P.; Damgaard, J.; Chen, P.-P.; Yi, W.-B.; Wang, Y.; Dang, K.; Li, C.-R.; Bu, W.-J. Phylogenetic Divergences of the True Bugs (Insecta: Hemiptera: Heteroptera), with Emphasis on the Aquatic Lineages: The Last Piece of the Aquatic Insect Jigsaw Originated in the Late Permian/Early Triassic. *Cladistics* **2016**, *32*, 390–405. https://doi.org/10.1111/cla.12137
[20] Li, H.M.; Deng, R.Q.; Wang, J.W.; Chen, Z.Y.; Jia, F.L.; Wang, X.Z. A Preliminary Phylogeny of the Pentatomomorpha (Hemiptera: Heteroptera) Based on Nuclear 18S rDNA and Mitochondrial DNA Sequences. *Mol. Phylogenet. Evol.* **2005**, *37*, 313–326. https://doi.org/10.1016/j.ympev.2005.07.013
[21] present paper.

[22] Wu, Y.-Z.; Rédei, D.; Eger, J., Jr.; Wang, Y.-H.; Wu, H.-Y.; Carapezza, A.; Kment, P.; Cai, B.; Sun, X.-Y.; Guo, P.-L.; Luo, J.-Y.; Xie, Q. Phylogeny and the Colourful History of Jewel Bugs (Insecta: Hemiptera: Scutelleridae). *Cladistics* **2018**, *34*, 502–516. https://doi.org/10.1111/cla.12224
[23] Wheeler, W.C.; Schuh, R.T. *Lestonia haustorifera* 16S ribosomal RNA gene, partial sequence; mitochondrial. *GenBank, direct submission* **2003**, *Accession No.* AY252664. Available online: www.ncbi.nlm.nih.gov/nuccore/48256021 (accessed on 11 January, 2026).
