# Supplementary material for: Sehiridae stat. nov., the Highly-Supported Monophylum Within the ‘Cydnoid’ Complex of Pentatomoid Families (Hemiptera: Heteroptera: Pentatomoidea)
Source: Insects. 2026 Aug 12;17(8):835. doi: 10.3390/insects17080835 (PMC13512964; doi:10.3390/insects17080835)
Supplement: Supplementary file 1 [file insects-17-00835-s001.zip › Table S3.pdf]

Table S3. List of outgroup specimens used in the phylogenetic analyses with GenBank accession numbers.

Infraorder/Family	Species	16S rDNA GenBank accession number	18S rDNA GenBank accession number	28S rDNA GenBank accession number
**Cimicomorpha/Miridae**	*Adelphocoris lineolatus* (Goeze, 1778)	AY252764 [1]	KJ461200 [12]	KJ461163 [12]
**Cimicomorpha/Nabidae**	*Himacerus mirmicoides* (O. G. Costa, 1834)	PP357152 [2]	PZ621209 [13]	PZ612361 [13]
**Cimicomorpha/Tingidae**	*Corythucha ciliata* (Say, 1832)	KC756280 [3]	KJ461201 [12]	KJ461175 [12]
**Pentatomomorpha/Alydidae**	*Riptortus pedestris* (Fabricius, 1775)	AY986811 [4]	AB725684 [14]	AB725684 [14]
**Pentatomomorpha/Aradidae**	*Isodermus planus* Erichson, 1842	-	MF460824 [15]	MF460813 [15]
	Libiocoris heissi Bai, Yang & Cai 2006	JQ780819 [5]	-	-
**Pentatomomorpha/Coreidae**	*Cletus punctiger* (Dallas, 1852)	AY986810 [6]	KJ461173 [12]	KJ461219 [12]
**Pentatomomorpha/Geocoridae**	*Geocoris ater* (Fabricius, 1787)	-	KJ461218 [12]	KJ461309 [12]
	Geocoris pallidipennis (A. Costa, 1843)	EU427336 [7]	-	-
**Pentatomomorpha/Largidae**	*Physopelta gutta* (Burmeister, 1834)	EU427343 [7]	AY742884 [16]	KJ461255 [12]
**Pentatomomorpha/Lygaeidae**	*Kleidocerys resedae* (Panzer, 1797)	KJ584365 [8]	-	-
	Kleidocerys sp.	-	AY252410 [17]	AY252629 [18]
	Lygaeus equestris (Linnaeus, 1758)	JQ234972 [9]	PZ621215 [13]	PZ612367 [13]
**Pentatomomorpha/Pyrrhocoridae**	*Dysdercus cingulatus* (Fabricius, 1775)	EU427335 [7]	KJ461263 [12]	KJ461235 [12]
	Pyrrhocoris apterus (Linnaeus, 1758)	KX821818 [8]	KX821833 [8]	KX821848 [8]
**Pentatomomorpha/Rhopalidae**	*Stictopleurus punctatonervosus* (Goeze, 1778)	-	KJ461217 [12]	KJ461286 [12]
	Stictopleurus subviridis Hsiao, 1977	EU826088 [9]	-	-
**Pentatomomorpha/Rhyparochromidae**	*Neolethaeus assamensis* (Distant, 1901)	KX505856 [10]	KJ461280 [12]	KJ461225 [12]
**Pentatomomorpha/Stenocephalidae**	*Dicranocephalus alticolus* (Zheng, 1981)	MW619667 [11]	KJ461228 [12]	KJ461267 [12]

References

[1] Wheeler, W.C.; Schuh, R.T. *Adelphocoris lineolatus* 16S ribosomal RNA gene, partial sequence; mitochondrial. *GenBank, direct submission* **2003**, *Accession No. AY252764*. Available online: https://www.ncbi.nlm.nih.gov/nuccore/AY252764 (accessed on 11 January, 2026).

[2] Lis, J.A.; Domagała, P.J.; Lis, B. New Molecular Phylogenetic Evidence Confirms Independent Origin of Coxal Combs in the Families of the 'Cydnoid' Complex (Hemiptera: Heteroptera: Pentatomoidea). *Insects* **2024**, *15*, 792. https://doi.org/10.3390/insects15100792

[3] Yang, W.; Yu, W.; Du, Y. The Complete Mitochondrial Genome of the Sycamore Lace Bug *Corythucha ciliata* (Hemiptera: Tingidae). *Gene* **2013**, *532*, 27–40. https://doi.org/10.1016/j.gene.2013.08.087

[4] Li, H.-M. *Riptortus pedestris* 16S ribosomal RNA gene, partial sequence; mitochondrial. *GenBank, direct submission* **2005**, *Accession No. AY986811*. Available online: https://www.ncbi.nlm.nih.gov/nuccore/AY986811 (accessed on 11 January, 2026).
[5] Song, F.; Li, H.; Shao, R.; Shi, A.; Bai, X.; Zheng, X.; Heiss, E.; Cai, W. Rearrangement of Mitochondrial tRNA Genes in Flat Bugs (Hemiptera: Aradidae). *Sci. Rep.* **2016**, *6*, 25725. https://doi.org/10.1038/srep25725
[6] Li, H.-M. *Cletus punctiger* 16S ribosomal RNA gene, partial sequence; mitochondrial. *GenBank, direct submission* **2005**, *Accession No. AY986810*. Available online: https://www.ncbi.nlm.nih.gov/nuccore/AY986810 (accessed on 11 January, 2026).
[7] Hua, J.; Li, M.; Dong, P.; Cui, Y.; Xie, Q.; Bu, W. Comparative and Phylogenomic Studies on the Mitochondrial Genomes of Pentatomomorpha (Insecta: Hemiptera: Heteroptera). *BMC Genomics* **2008**, *9*, 610. https://doi.org/10.1186/1471-2164-9-610
[8] Crumière, A.J.J.; Santos, M.E.; Sémon, M.; Armisén, D.; Moreira, F.F.F.; Khila, A. Diversity in Morphology and Locomotory Behavior Is Associated with Niche Expansion in the Semi-Aquatic Bugs. *Curr. Biol.* **2016**, *26*, 3336–3342. https://doi.org/10.1016/j.cub.2016.09.061
[9] Hua, J.; Li, M.; Dong, P.; Bu, W. *Stictopleurus subviridis* mitochondrion, complete genome. *GenBank, direct submission* **2008**, *Accession No. EU826088*. Available online: https://www.ncbi.nlm.nih.gov/nuccore/EU826088 (accessed on 11 January, 2026).
[10] Jiang, P. *Neolethaeus assamensis* mitochondrion, complete genome. *GenBank, direct submission* **2016**, *Accession No. KX505856*. Available online: https://www.ncbi.nlm.nih.gov/nuccore/KX505856 (accessed on 11 January, 2026).
[11] Ye, F.; Kment, P.; Rédei, D.; Luo, J.Y.; Wang, Y.H.; Kuechler, S.M.; Zhang, W.W.; Chen, P.P.; Wu, H.Y.; Wu, Y.Z.; Sun, X.Y.; Ding, L.; Wang, Y.R.; Xie, Q. Diversification of the Phytophagous Lineages of True Bugs (Insecta: Hemiptera: Heteroptera) Shortly after That of the Flowering Plants. *Cladistics* **2022**, *38*, 403–428. https://doi.org/10.1111/cla.12501
[12] Wang, Y.-H.; Cui, Y.; Rédei, D.; Baňař, P.; Xie, Q.; Štys, P.; Damgaard, J.; Chen, P.-P.; Yi, W.-B.; Wang, Y.; Dang, K.; Li, C.-R.; Bu, W.-J. Phylogenetic Divergences of the True Bugs (Insecta: Hemiptera: Heteroptera), with Emphasis on the Aquatic Lineages: The Last Piece of the Aquatic Insect Jigsaw Originated in the Late Permian/Early Triassic. *Cladistics* **2016**, *32*, 390–405. https://doi.org/10.1111/cla.12137
[13] present paper.
[14] Futahashi, R.; Tanaka, K.; Tanahashi, M.; Nikoh, N.; Kikuchi, Y.; Lee, B.L.; Fukatsu, T. Gene Expression in Gut Symbiotic Organ of Stinkbug Affected by Extracellular Bacterial Symbiont. *PLoS One* **2013**, *8*, e64557. https://doi.org/10.1371/journal.pone.0064557
[15] Weirauch, C.; Schuh, R.T.; Cassis, G.; Wheeler, W.C. Revisiting Habitat and Lifestyle Transitions in Heteroptera (Insecta: Hemiptera): Insights from a Combined Morphological and Molecular Phylogeny. *Cladistics* **2019**, *35*, 67–105. https://doi.org/10.1111/cla.12233
[16] Li, H.M.; Deng, R.Q.; Wang, J.W.; Chen, Z.Y.; Jia, F.L.; Wang, X.Z. A Preliminary Phylogeny of the Pentatomomorpha (Hemiptera: Heteroptera) Based on Nuclear 18S rDNA and Mitochondrial DNA Sequences. *Mol. Phylogenet. Evol.* **2005**, *37*, 313–326. https://doi.org/10.1016/j.ympev.2005.07.013
[17] Wheeler, W.C.; Schuh, R.T. *Kleidocerys* sp. WCW-2003 18S ribosomal RNA gene, partial sequence. *GenBank, direct submission* **2003**, *Accession No. AY252410*. Available online: https://www.ncbi.nlm.nih.gov/nuccore/AY252410 (accessed on 11 January, 2026).
[18] Wheeler, W.C.; Schuh, R.T. *Kleidocerys* sp. WCW-2003 28S ribosomal RNA gene, partial sequence. *GenBank, direct submission* **2003**, *Accession No. AY252629*. Available online: https://www.ncbi.nlm.nih.gov/nuccore/AY252629 (accessed on 11 January, 2026).
